# Associations of major congenital anomalies, long-term health conditions, and socioeconomic deprivation with educational attainment in Wales: A population-based data linkage study

**DOI:** 10.1371/journal.pmed.1005050

**Published:** 2026-10-16

**Authors:** Ieuan Scanlon, Anna Rawlings, David Tucker, Daniel S. Thayer, Hywel T. Evans, Joanne Given, Sam Jones, Maria Loane, Chloe Morgan, Joan K. Morris, Sue Jordan

**Affiliations:** 1 Faculty of Medicine, Health and Life Sciences, Swansea University, Swansea, United Kingdom; 2 Congenital Anomaly Registers & Information Service for Wales (CARIS), Public Health Knowledge & Research, Public Health Wales, Swansea, United Kingdom; 3 Data Solutions Division, Mathematica Policy Research, Princeton, New Jersey, United States of America; 4 Faculty of Life & Health Sciences, Ulster University, Belfast, United Kingdom; 5 School of Health and Medical Sciences, City St George’s, University of London, London, United Kingdom; London School of Hygiene and Tropical Medicine, UNITED KINGDOM OF GREAT BRITAIN AND NORTHERN IRELAND

## Abstract

**Background:**

Education outcomes are associated with life chances. However, deprivation, ill-health and disability are barriers to achievement. We aimed to investigate educational attainment of children with and without major congenital anomalies in state-funded mainstream schools at ages 11 and 16 (key stages [KS] 2 and 4), accounting for maternal and child socioeconomic deprivation, demographic factors, comorbidities, and special education needs (SEN) provision.

**Methods and findings:**

We conducted a retrospective population-based cohort study using the Secure Anonymised Information Linkage (SAIL) Databank to link routinely collected electronic health and education records of children born in Wales between January 1st, 1998 and December 31st, 2007, who were followed up until December 31st, 2019. Education outcomes were explored using logistic regression, adjusting for: major congenital anomalies, maternal and child socioeconomic deprivation, medicated co-morbidities, hospitalisation, gestation length, child sex, year of birth, and SEN provision.

Children with major congenital anomalies were less likely to achieve national academic standards, including those required for some professional courses: however, attainment was more strongly associated with socioeconomic deprivation. At age 11, 81.9% (7167/8754) with and 93.8% (232,450/247,814) without major congenital anomalies attained established standards (odds ratio [OR] 0.30; 95% confidence intervals [CI] [0.28,0.32]). At age 16, 46.8% (2070/4427) with and 56.1% (69,732/124,300) without major congenital anomalies achieved 5 General Certificates of Secondary Education (GCSEs) at grades C–A*, including English/Welsh, Maths and Science (EWMS) (OR 0.69; 95% CI [0.65,0.73]). Discrepancies narrowed in adjusted analyses, particularly when SEN provision was accounted: aOR 0.72; 95% CI [0.66,0.78] at KS2, age 11, and aOR 0.93; 95% CI [0.87,1.00] for 5 GCSEs C–A* with EWMS. These GCSEs were achieved by 29.7% (307/1034) children with major congenital anomalies and 38.4% (10,875/28,305) of unaffected children in the most deprived quintile (using maternal Welsh Index of Multiple Deprivation: in the least deprived quintile, figures were 67.6% (547/810) and 75.0% (16,978/22,644), respectively.

Children with major congenital anomalies, receiving maximum SEN support, and eligible for Free School Meals (FSM) were the least likely to achieve national academic standards: 5/192 (2.6%) obtained 5 GCSEs C–A* with EWMS, as did 37/354 (10.4%) ineligible for FSM. The strongest associations with 5 GCSEs C–A* with EWMS, were SEN statements (aOR 0.07; 95% CI [0.06,0.07]), FSM eligibility (aOR 0.39; 95% CI [0.37,0.41]), and epilepsy (aOR 0.60; 95% CI [0.45,0.80]). Study limitations included absence of data for 15%–18% of children, mainly those educated outside mainstream schools, and for some comorbidities; generalisation of findings to other settings or other countries is uncertain.

**Conclusions:**

Many children with major congenital anomalies from least deprived quintiles attained national academic standards, including 5 GCSEs C–A* with EWMS. The children living in the most deprived quintiles or eligible for FSM and with ill-health from major congenital anomalies and/or epilepsy were two to four times less likely to attain these standards. SEN provision mitigated the associations with disadvantage, but children from socioeconomically deprived quintiles with major congenital anomalies were unlikely to achieve 5 GCSEs C–A* with EWMS.

## Author summary

**Why was this study done?:**

- Major congenital anomalies affect around 267 of every 10,000 births in Europe. In Wales, 319 in 10,000 are affected.
- Most (~96%) children born with major congenital anomalies survive infancy and over 80% are educated in mainstream schools.
- Few national whole population cohorts have followed these children to ages 11 and 16 to explore educational attainment, including national qualifications.

**What did the researchers do and find?:**

- Our cohort included 256,568 children aged 11, and 128,727 aged 16, born between 1988 and 2007. We built explanatory models to report any associations between educational attainment and major congenital anomalies, deprivation, epilepsy, asthma, diabetes, hospitalisation, preterm birth, demographics, and special education needs (SEN) provision.
- Differences in educational attainment between children eligible and ineligible for free school meals were greater than differences between children with and without major congenital anomalies, when measured at each level of SEN provision.
- At age 16, 67.8% of children with major congenital anomalies in the least deprived households attained 5 general certificates of secondary education (GCSEs), including English/Welsh plus maths and science, at grades C–A*, which are the entry qualifications for many professional courses. In the most deprived households, only 38.4% of children without and 29.7% of children with major congenital anomalies achieved this standard.
- SEN provision narrowed the gap between children with and without major congenital anomalies, but did not close it.

**What do these findings mean?:**

- In Wales, a profoundly disadvantaged group of children living with disability and deprivation need greater health, education, and social support to obtain the entry qualifications for professional courses.
- Our study is limited by the inability to account for some potentially important factors, including school attendance and behavioural issues. Our findings may not apply to children educated outside mainstream education or to other countries.

## Abstract 3

Ieuan Scanlon and colleagues found that children with major congenital anomalies were less likely to meet national academic standards in Wales. However, educational attainment was more strongly associated with socioeconomic deprivation.

## Introduction

Educational attainment is associated with life chances [1], achievement [2], quality of life [3], and health [4]. Ill-health and disability are barriers to educational attainment and optimal school performance [5]. Disability and learning difficulties may emanate from congenital anomalies [6–9]. Major congenital anomalies affected 212.6 per 10,000 (95% confidence intervals [CI] [211.8, 213.4]) live or still births in Europe 2005–2023 [10].

Most (97.3%) children with major congenital anomalies survive infancy, due to improved care and surgical interventions: [11–16] 96.9% are alive at 10 years [17]. Accordingly, research is focussing on their life chances. However, evidence suggests their educational attainment is lower than their peers’ [18–28], despite provision for special educational needs (SEN) [22,29–36].

Children with certain congenital anomalies may also be more likely to experience co-morbidities, such as epilepsy, cardiovascular insufficiency [37–40]), cancer and injury [41], and require surgery, hospitalisation and other treatments [42], which may detract from educational attainment [5]. Of the three most common long-term medicated conditions requiring hospitalisation in childhood [43], epilepsy is linked with lower educational attainment [44], but the literature offers no consensus on asthma [45–48] and diabetes [49,50]. Although educational attainment may be affected by congenital anomalies or medicated long-term conditions [51,52], intergenerational social and cultural capital may not, suggesting that higher socioeconomic status may protect individuals from some adverse consequences of congenital anomalies, and time spent in hospital, away from school [53].

Educational attainment is linked with socioeconomic status (SES) [54,55], childhood ill-health and disability, which are concentrated in the most deprived communities [56–58]. The proportion of UK children with disability increased from 7.3% in 2004/5% to 11% in 2021/22 [58,59].

Increasing child poverty [60], the widening attainment gaps at age 16 between those eligible and ineligible for free school meals (FSM) [61], re-organisation of SEN provision [62], and the expanding population of children with major congenital anomalies attending mainstream schools necessitate understanding the determinants of educational attainment and targets for support. How biological and socioeconomic disadvantages combine and interact, are mitigated by additional support, as delivered in routine practice, and whether this generates a profoundly disadvantaged subgroup, is not, to our knowledge, fully explored. The literature focuses on isolated congenital anomalies [22,23]. Current evidence is limited by the paucity of large whole-population studies with objective measures of educational attainment and key covariates. We aimed to explore associations between educational attainment of children attending state-funded mainstream schools in Wales at the end of primary and secondary schooling, at ages 11 (KS [key stage]2) and 16 years (KS4) [55,56], and major congenital anomalies, accounting for: socioeconomic status (SES) (as FSM eligibility, income, environment, access to services, and maternal deprivation), clinical factors (hospitalisation, common medicated co-morbidities [asthma, epilepsy, diabetes]), sex, preterm birth, year of birth and SEN provision.

## Methods

This population-based cohort study is reported in accordance with the Reporting of Studies Conducted using Observational Routinely-Collected Data (RECORD) guidelines (RECORD Statement, S1 Checklist) [63]. The main data linkage and analytical approaches for this EUROLINKCAT study were published before we accessed data [64,65]. The analysis plan was expanded to include co-morbidities, gestation, and SEN provision by level. We removed the Welsh Index of Multiple Deprivation (WIMD) components relating to educational outcomes at KS2 and KS4 to avoid circular arguments (Text A in S1 Appendix has details). The pre-published protocol and analysis plan are appended (S1 File). Stratified analyses, sensitivity analyses, mediation, and moderation exploration were prompted by reviewers (Text B and C in S1 Appendix).

### Data sources

Routinely collected administrative data were accessed *via* the Wales national databank, the Secure Anonymised Information Linkage (SAIL) Databank [66,67]. The maternity, prescribing, hospital admission and socioeconomic data of all children born in Wales January 1st, 1998 to December 31st, 2007 (earliest and latest data available) were selected as described [64,65]. Children born to mothers resident in Wales were identified from the National Child Community Health Database (NCCHD), which supplied data on maternity and perinatal outcomes. Children’s birth dates, sex, and, where relevant, dates of death were ascertained from the Annual District Births Extract (ADBE) and the Deaths’ Register (ADDE). Records of eligible infants were linked to the Welsh Demographic Service dataset (WDS) to obtain anonymised information on geographical area of residence and facilitate linkage to the WIMD [68]. Approximately 85% of Welsh primary care practices contribute routinely collected data to SAIL, including all primary care prescriptions, from 86.2% of the population and 86.5% of children, as the Welsh Longitudinal General Practice (WLGP) dataset [69]. The study was restricted to this portion of the national population to account for medicated epilepsy, asthma and diabetes. Data on hospitalisations were obtained from the Patient Episodes Dataset for Wales (PEDW). Table 1 summarises the datasets.

**Table 1:** Data sources in this study.

Data source	Acronym	Data extracted	Number of individuals	Link to SAIL Databank metadata
*Demographics:*				
National Child Community Health Database	NCCHD	Children’s IDs, maternity and perinatal outcomes	1,526,405	https://healthdatagateway.org/en/dataset/360
Annual District Births Extract	ADBE	Week of birth and sex	907,729	https://healthdatagateway.org/en/dataset/321
Annual District Deaths Extract	ADDE	Date of death	1,153,316	https://healthdatagateway.org/en/dataset/316
Welsh Demographic Service Dataset	WDSD	Geographical area of residence, hence linkage to the Welsh Index of Multiple Deprivation (WIMD)	5,945,485	https://healthdatagateway.org/en/dataset/359
*Health:*				
Welsh Longitudinal General Practice dataset	WLGP	Prescription data	5,917,115	https://healthdatagateway.org/en/dataset/355
Patient Episode Dataset for Wales	PEDW	Hospitalisation	3,838,725	https://healthdatagateway.org/en/dataset/318
Welsh Congenital Anomaly Register and Information Service	CARIS	Major congenital anomalies	36,251	https://healthdatagateway.org/en/dataset/319
*Education:*				
Education Wales dataset	EDUW	Learners’ IDs, FSM, SEN provision, KS2 results, KS4 results	1,279,866	https://healthdatagateway.org/en/dataset/314

Notes to Table 1: ID, identity; FSM, free school meals; KS, key stage; SEN, special educational needs.

FSM eligibility was based on parents’ receipt of benefits, including income support, income-related employment and support allowance, and child tax credit, provided income was below £16,190 per year. During the study period, there was a staggered replacement of these benefits by receipt of Universal Credit, plus an earned income allowance of £7,400 [70,71].

Major congenital anomalies, as defined and classified by the European Surveillance of Congenital Anomalies (EUROCAT) network [70,72], are recorded by the Welsh Congenital Anomaly and Information Service (CARIS) register held within Public Health Wales and shared with SAIL. Major congenital anomalies are structural changes that have important medical, surgical, social, or cosmetic consequences for the affected individual, and typically require medical intervention. They account for most of the mortality, morbidity, and disability related to congenital anomalies [70]. Reports of congenital anomalies are received from clinicians, including obstetricians, paediatricians and surgeons, and families, and verified by the database manager (DT). We analysed ‘All major congenital anomalies’ as a single group defined by allocation of International Classification of Diseases version 10 (ICD10) codes listed in EUROCAT guide 1.4 (List in S1 Appendix) (binary variable al1) [72], in line with experts’ [73] and national reporting [74], to offer an overview of needs and avoid too many small categories obscuring associations [75]. We reported isolated structural major congenital anomalies, often associated with surgical intervention, and two chromosomal congenital anomalies, Turner and Down syndromes, where numbers permitted (Text D in S1 Appendix). Isolated anomalies are defined by EUROCAT as a major congenital anomaly in one organ system only or with a known sequence where multiple congenital anomalies cascade as a consequence of a single primary anomaly [65]. Children with major congenital anomalies outside these categories were analysed as a single group, ‘other major congenital anomalies’, together with children with more than one major congenital anomaly.

We linked children to their education records until December 31st, 2,019 in the Pupil Level Annual School Census and Welsh Examinations Database, held within SAIL as the Education Wales (EDUW) dataset. Within EDUW, children in mainstream state-funded schools are identified through school-derived learner identities (IDs). Data relating to children attending special schools were not available, and therefore our analysis is restricted to children considered sufficiently well, physically and mentally, not to need the expertise available only in special schools. Pupil characteristics including eligibility for FSM and records of SEN provision allocated to the pupil from the beginning of each academic year, along with attainment data for KS2 were obtained from the Pupil Level Annual School Census tables within the EDUW dataset, while attainment data for KS4 were obtained from Welsh Examinations Database tables. Within SAIL, all IDs (including learner IDs) are matched to the standard SAIL anonymised linkage field (ALF) using probability matching based on National Health Service (NHS) numbers or personal identifiers [66,67].

### Inclusion criteria

Our cohort comprised all live born children with primary care records, with and without major congenital anomalies born in Wales between 1998–2007 [76], alive on their 16th birthday and with academic results in EDUW by age 11 (KS2) or age 16 (KS4). Children alive at 11, but dying before 16 were excluded in all analyses. (In Wales, there are ~30 deaths each year in children aged 12–17; about 30% are expected deaths [77].) Children born 1998–2007 and 1998–2002 were included in the analysis of KS2 (age 11) and KS4 (age 16) results, respectively.

### Outcomes

The Welsh National Curriculum aims to provide a firm foundation in language (English or Welsh/Cymraeg), maths and science (EWMS). At KS2, age 11, the expected level of attainment in Wales is Level 4 or above in all subjects including EWMS, assessed through a mixture of standardised and moderated teacher assessments. At KS4, age 16, the end of compulsory schooling, children work towards externally assessed national qualifications in core (EWMS), foundation and optional subjects. The standard KS4 qualifications are the General Certificates of Secondary Education (GCSEs), although institutions may offer other academic or vocational qualifications, which may or may not be equivalent to whole or part GCSEs. The Welsh Examinations Database records whether a child has achieved Level 1 (G–A*) or Level 2 (C–A*) in at least 5 GCSE subjects or equivalents and whether these included EWMS. Achievement of 5 GCSEs, C–A* with EWMS governs access to some higher education and professional courses, such as nursing.

Expected levels of attainment have remained consistent over time. We selected outcomes at ages 11 and 16, consistent with UK-wide standards [78,79] (Table 2). The ‘lower-level pass’ outcome captured all children who met expectations to any extent, whilst the high-level outcome included only children meeting expectations in all three core subjects.

**Table 2:** Attainment standards.

Stage	Low-level/other pass/any of:	High- level pass
Key Stage 2, Age 11	Level 4 English/Welsh Level 4 Maths Level 4 Science Level 4 English/Welsh and Maths Level 4 English/Welsh and Science Level 4 Maths and Science Level 4 EWMS	Level 4 EWMS
Key Stage 4, Age 16	Level 1 (G–A* in any 5 GCSEs) Level 2 (C–A* in any 5 GCSEs) Level 1 (G–A* in 5 GCSEs including EWMS) Level 2 (C–A* in 5 GCSEs including EWMS)	Level 2 (C–A* in 5 GCSEs, including EWMS)

Notes to Table 2: EWMS, English/Welsh, plus Maths and Science; GCSE, General Certificate of Secondary Education.

Children who achieve level 2 are also deemed to have achieved level 1.

### Covariates

Covariates were added sequentially to explanatory analyses to assess their influence and explore modifiable risk factors. We adjusted for covariates related to: demographics (child’s sex and year of birth); preterm birth; deprivation indices of both child and mother; hospital stay; long-term conditions (as prescribed medicines); and SEN provision. We categorised gestational age data from the NCCHD as very pre-term (<32 weeks; gestation), pre-term (32–36 + 6 weeks) and term (≥37 weeks) (Table A, Text E in S1 Appendix).

We accounted for mothers’ deprivation at the time of childbirth at the local area level and children’s deprivation at both the local area and household level. We took the access to services (ATS), physical environment and income quintile scores from the 2014 version of the WIMD, as measures of the child’s local area deprivation [80], using the child’s registered address on the 1st May in the year of their key stage 2 and 4 assessments. WIMD has 8 components: income, employment, health, education, ‘Access to Services’, community safety, physical environment, housing. Each domain is compiled from a range of indicators. For example, the education outcome data comprise: KS2 ′Average Point Score’, KS4 ′Capped Point Score’, and K4 ‘Level 2 Inclusive’, repeat absenteeism, proportion of 18–19-year-olds not entering Higher Education, and proportion of 25-64-year-olds with no qualifications [80]. We used the full WIMD to account for mothers’ deprivation, but only selected components (above) for children’s deprivation. We analysed free school meals (FSM) eligibility (not receipt) during the child’s KS2 and KS4 years, as an indicator of household-level deprivation [81].

Children or young people are considered to have SEN if they have a learning difficulty or disability which requires additional educational provision, over and above routine schooling; this does not always parallel severity of physical disability. Procedures for service allocation are described in the 2002 code of practice [82], which has been revised [83]. Needs are assessed by schools and the local education authority; decisions are sometimes appealed. SEN provision recorded in the year of assessment was taken as a marker for the perceived severity of a child’s difficulties: (1) no provision, (2) school action plan (in-school support), (3) action plan plus (involving external support from, for example, psychologists), and (4) statementing (involving additional funded education, health and care plans) [82].

Medicated asthma, type 1 diabetes, epilepsy or cardiac conditions, were indicated by primary care records of > 1 prescription for any of these conditions [84] by the 1st May of a child’s key stage 2 and 4 assessment years. ATC (anatomic, therapeutic chemical) codes related to these conditions were identified, as previously [85–87], and matched with Readv3 codes (see ‘Codes’ below). We discounted single prescriptions as likely to represent nonadherence. Hospitalisation was measured as the total number of days (with overnight stays) spent in hospital by the 1st May of a child’s KS2 assessment year and between KS2 and 1st May of the KS4 assessment year. Days spent in hospital offers a proxy measure for illnesses other than those accounted; it represents a small proportion of school nonattendance.

### Statistical analyses

Record linkage was undertaken *via* Structured Query Language. All other data preparation and analyses were completed in R version 4.3.3 using packages: stats v4.3.3, dplyr v1.1.4, olsrr v0.6.0, lmtest v0.9.40.

Major congenital anomalies (MaCAs) were analysed together, and, where numbers permitted, as isolated MaCAs. We tested whether MaCAs and FSM altered the likelihood of having SEN provision, and the association between maternal deprivation and major congenital anomalies. To explore whether major congenital anomalies were associated with educational attainment, when demographic, biological and socioeconomic factors were accounted, logistic regression [88] was used to explore four education outcomes (Table 2), set as binary categorical variables. Explanatory variables were selected and categorised *a priori*. Covariates were added to models in four categories: child traits (year of birth, sex, and gestational age), deprivation (FSM, ATS quintile, physical environment quintile, income quintile, and mother’s deprivation quintile), medical interventions and co-morbidities (days in hospital, asthma, epilepsy, diabetes, and cardiac conditions), and SEN provision (S1 Data). Models were tested for collinearity. To avoid over-specification in these explanatory models (Fig 1), results were reported with and without SEN provision, Down syndrome and deprivation, and separately for boys and girls, and for each of the four levels of SEN provision. We assessed the associations between educational attainment and the combined effects of a) SEN and MaCAs/sex/hospitalisation/deprivation measures in turn, and b) MaCAs and deprivation measures in turn as interaction terms (Text F in S1 Appendix, S1 Data). Interactions were assessed using product terms (multiplicative interactions). Hospitalisation, deprivation at birth and SEN provision were examined for mediation effects [89] (Text C in S1 Appendix). A sensitivity analysis was undertaken for FSM eligibility at both key stages: all missing values were imputed as eligible, and then as ineligible [28]. There were no other data imputations.

**Fig 1:**
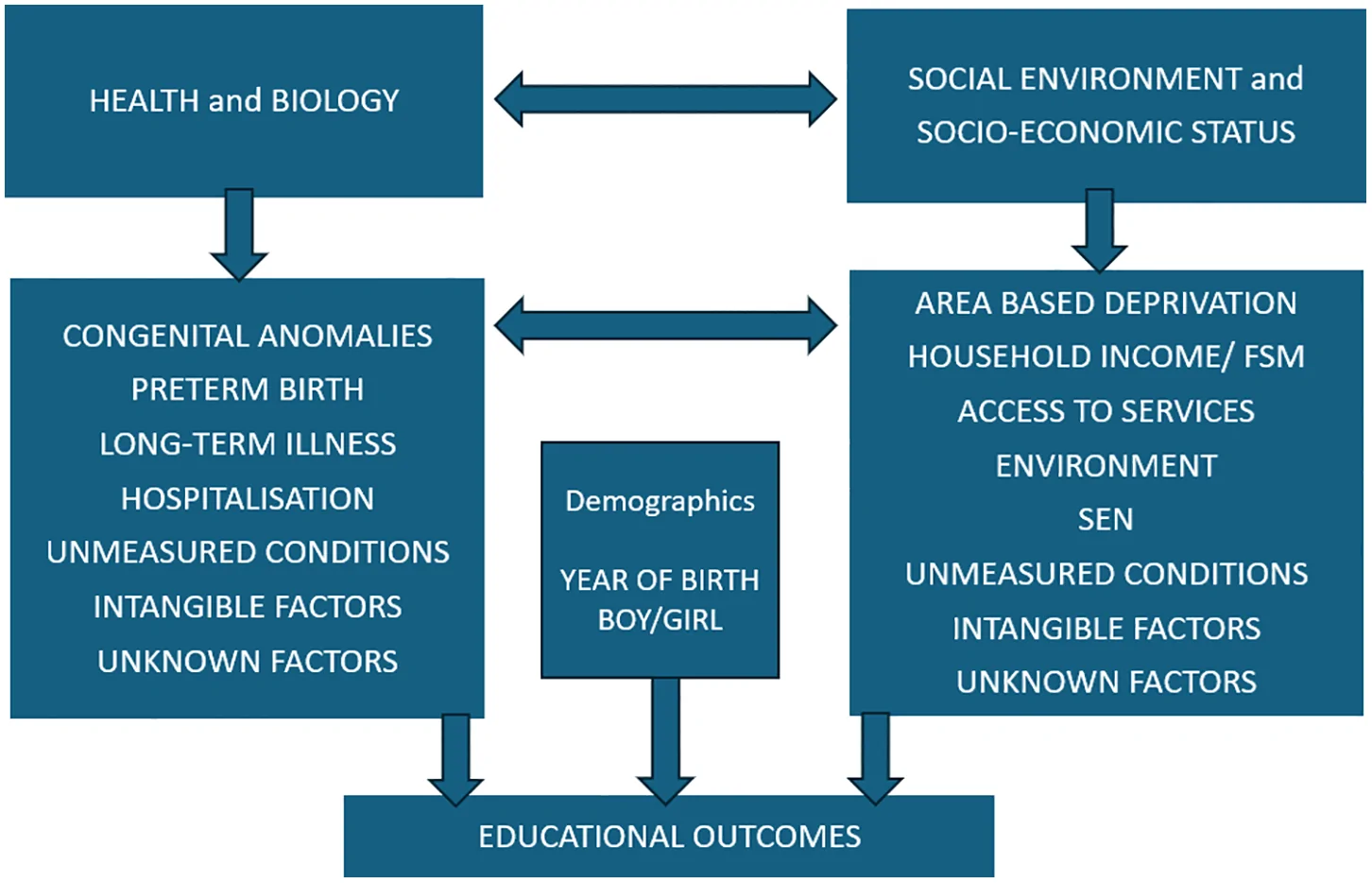
Determinants of educational outcomes. Note to Fig 1: SEN special educational needs.

### Artificial tools and technologies

Neither artificial tools nor technologies were used in this work.

### Ethical and governance approval

The EUROlinkCAT study was approved by SAIL’s Independent Governance Review Panel (IGRP), which comprises information governance experts and patient and public representatives, on January 27th, 2018 and December 20th, 2022 (number 0511). The study used only irrevocably anonymised routinely collected data held within the SAIL Databank. Project 0511 was reviewed and approved by the SAIL IGRP, which assesses whether the project is appropriate, legally compliant, and in the public interest. Separate ethical approval was not required as only anonymised data were used, within a secure trusted research environment [66]. Disclosure of small numbers (1–4) in any category is prohibited, as discussed [90].

## Results

We report on 256,568 children at age 11, KS2, and 128,727 at age 16, KS4 (Table 3). Most (83%–87%) children were linked to their education records at both key stages. A further 2%–5% of children had either no primary care data or no information on SES or uncertain gestation (Tables A, B in S1 Appendix). After exclusions for missing data, we identified 8,754 children with MaCAs with KS2 records and 4,427 with KS4 records (Table 3, Figs 2–3). Most children present in the KS4 cohort were also present in the KS2 cohort: 4,350 children with MaCAs and 122,100 children without MaCAs contributed to education records at both stages. Children (77 with MaCAs and 2,200 without) transferring into mainstream education in Wales were present at KS4, but not KS2. Transfers were from private, home or special education. We found no differences in SES between children with and without primary care data (Text G in S1 Appendix). Only children identified in all datasets were included in the analysis.

**Table 3:** Number of children in the key stage 2 and 4 cohorts.

	Available		Linked to attainment data				Linked to all data			
**With anomalies** ^**§**^	**No anomalies**	**With anomalies**		**No anomalies**		**With anomalies**		**No anomalies**	
***n***	***n***	***n***	**%**	***n***	**%**	***n***	**%**	***N***	**%**
KS2^*^	10,821	303,776	9,223	85.5%	261,827	87.0%	8,754	81.2%	247,814	82.4%
KS4^ⴕ^	5,454	146,459	4,524	82.9%	127,081	86.8%	4,427	81.2%	124,300	84.9%

Notes to Table 3: KS, key stage; ‘anomalies’ refers to ‘major congenital anomalies‘, as defined in EUROCAT guide 1.4.

* born 1998–2007; ^ⴕ^ born 1998–2002, % of total quoted.

§children with major congenital anomalies included in the al1 category of EUROCAT guide 1.4 (List in S1 Appendix)

**Fig 2:**
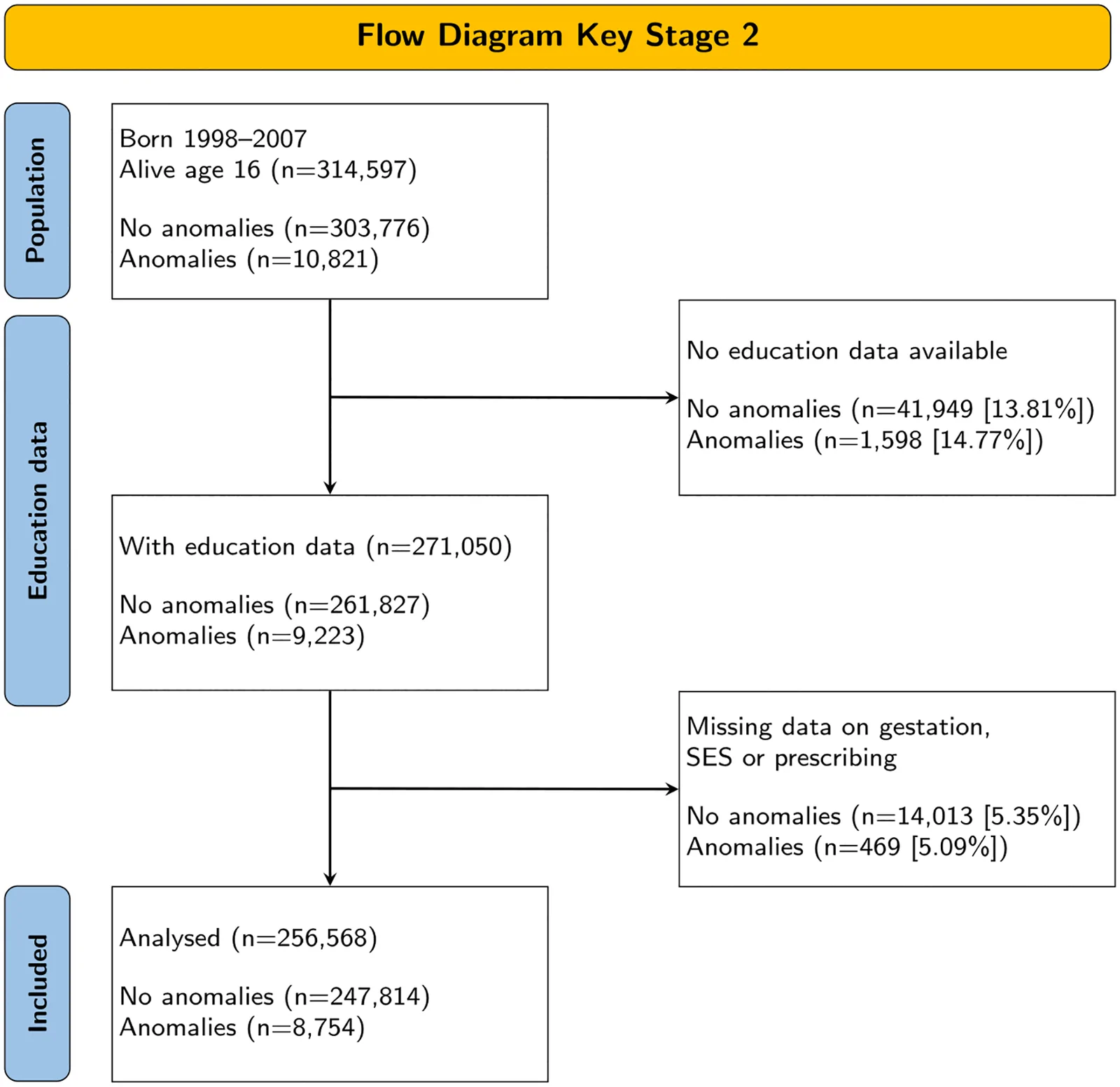
Flow diagram of participants at age 11, key stage 2. Note to Fig 2: ‘anomalies’ refers to ‘major congenital anomalies, defined in EUROCAT guide 1.4 (List in S1 Appendix).

**Fig 3:**
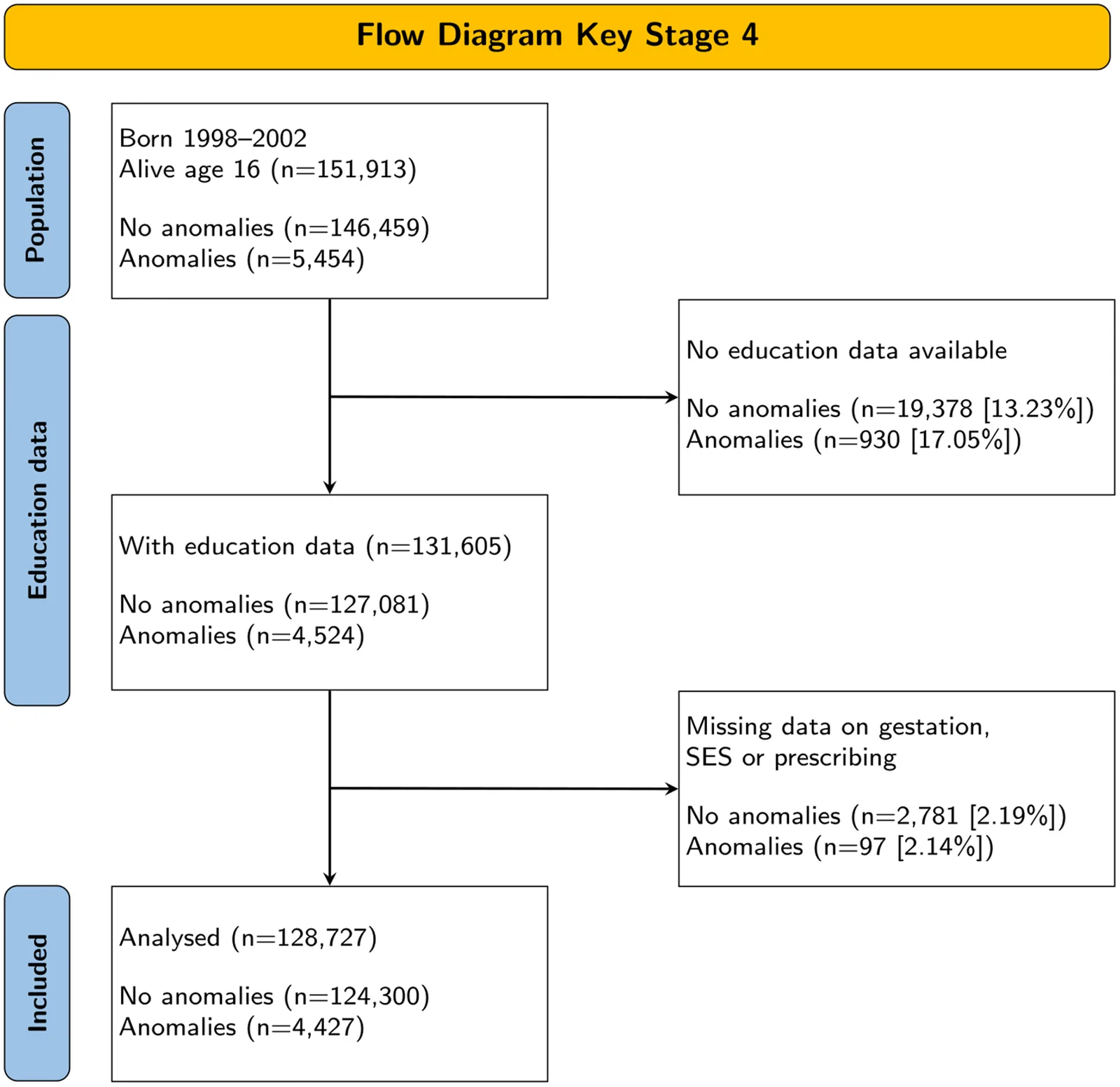
Flow diagram of participants at age 16, key stage 4. Note to Fig 3: ‘anomalies’ refers to ‘major congenital anomalies, defined in EUROCAT guide 1.4 (t List in S1 Appendix).

Just under half the children with major congenital anomalies (MaCAs) could be categorised within the ‘isolated structural major congenital anomalies’ taxonomy. The remainder had more than one MaCA or a genetic or other syndrome or a MaCA with too few children for analysis (List in S1 appendix) (Table 4).

**Table 4:** Prevalence of major congenital anomalies.

	Key Stage 2 (age 11) born 1998–2007		Key Stage 4 (age 16) born 1998–2002	
Isolated anomalies*	***n***	**% of children with anomalies**	***n***	**% of children with anomalies**
Cleft lip with or without cleft palate	177	2.0%	78	1.8%
Cleft palate	153	1.7%	74	1.7%
CHD (excluding severe CHD)	1,617	18.5%	808	18.3%
Severe CHD	383	4.4%	183	4.1%
Oesophageal atresia	14	0.2%	<10	0.2%
Ano-rectal atresia and stenosis	21	0.2%	<15	0.3%
Diaphragmatic hernia	26	0.3%	15	0.3%
Gastroschisis	119	1.4%	43	1.0%
Multicystic renal dysplasia	99	1.1%	48	1.1%
Hypospadias	691	7.9%	347	7.8%
Spina bifida	22	0.3%	13	0.3%
Limb reduction defects	70	0.8%	35	0.8%
Hydrocephalus	55	0.6%	28	0.6%
Craniosynostosis	99	1.1%	45	1.0%
Chromosomal anomalies				
Down syndrome†	202	2.3%	66	1.5%
Turner syndrome†	27	0.3%	13	0.3%
Other EUROCAT anomalies*	4,979	56.9%	2,610	59.0%
All MaCAs (EUROCAT al1) ‡	8,754	100%	4,427	100%
		**% of population**		**% of population**
No MaCA	247,814	96.6	124,300	96.6
TOTAL	256,568	100	128,727	100

Notes to Table 4: CHD, congenital heart defect; MaCA, major congenital anomaly.

* ‘anomalies’ refers to ‘major congenital anomalies’, as defined in EUROCAT guide 1.4 (List in S1 Appendix).

† Children with Down or Turner syndrome with additional major congenital anomalies are included here, as the problems have a common genetic aetiology.

‡ To delineate major congenital anomalies and achieve comparability of prevalence rates between registers across Europe, the count of cases with all major congenital anomalies (al1) includes all cases with one or more codes in the Q chapter of International Classification of Diseases version 10 (ICD10) and a limited set of conditions coded outside the Q chapter, as specified in the definition of subgroups in Chapter 3.3 (EUROCAT 2013/2021 P.81): Q-chapter, D215, D821, D1810^, P350, P351, and P371 [72]. Children with nonchromosomal genetic conditions are captured in the dataset and are included in the “ALL MAJOR CONGENITAL ANOMALIES” subgroup. This includes all genetic conditions with an ICD10 code beginning with Q and Di George syndrome (ICD10 code D821). The EUROCAT syndrome guide lists the syndromes included [91]. It is possible that some syndromes may be undiagnosed and therefore misclassified as ‘isolated’ MaCAs; EUROCAT includes all MaCAs diagnosed and reported in infancy or prenatally, including those commonly associated with structural malformations and/or found in the Q-chapter of ICD10.

Low numbers have been redacted to ensure compliance with governance requirements in subgroup analyses presented in supplementary tables.

### Cohort description

When compared with children without MaCAs, children with MaCAs were more likely to be male, born pre-term to mothers in the most and least deprived areas, eligible for FSM, living in the most deprived income quintile and most deprived environment quintile. They were less likely to live in the areas with poor access to services (ATS). The largest differences in prevalence of deprivation (of ~2%–4%), between those with and without MaCAs, related to FSM eligibility and ATS. Other prevalence differences were ~1% (Table 5).

There was no overall association between maternal WIMD at birth and MaCA (most deprived versus least deprived quintiles OR 1.03; (95% CI [0.97,1.10]. Only gastroschisis, cleft lip and multicystic renal dysplasia were more prevalent in the most deprived quintile (Table C in S1 Appendix). Educational attainment was associated with deprivation in unadjusted analyses and when the child’s sex was accounted (Table D in S1 Appendix).

**Table 5:** Children with and without major congenital anomalies.

	Key Stage 2, age 11				Key Stage 4, age 16			
	With anomalies		No anomalies		With anomalies		No anomalies	
	*n*	%	*n*	%	*n*	%	*n*	%
Total	8,754	100	247,814	100	4,427	100	124,300	100
Sex								
Male	5,112	58.4	125,890	50.8	2,540	57.4	62,678	50.4
Female	3,642	41.6	121,924	49.2	1,887	42.6	61,622	49.6
Gestation								
<32 weeks	306	3.5	1,983	0.8	153	3.5	978	0.8
32–36 + 6 weeks	1,033	11.8	14,621	5.9	523	11.8	7,295	5.9
Term ≥ 37 weeks	7,415	84.7	231,210	93.3	3,751	84.7	116,027	93.3
Mother’s deprivation quintile								
5 (most deprived)	2,416	27.6	65,423	26.4	1,152	26.0	31,410	25.3
4	1,856	21.2	52,537	21.2	919	20.8	26,093	21.0
3	1,672	19.1	48,076	19.4	864	19.5	24,328	19.6
2	1,339	15.3	41,881	16.9	692	15.6	21,495	17.3
1 (least deprived)	1,462	16.7	39,898	16.1	800	18.1	20,974	16.9
FSM								
Eligible	1,987	22.7	47,828	19.3	840	19.0	18,911	15.2
Not eligible	6,767	77.3	199,986	80.7	3,587	81.0	105,389	84.8
Child’s income quintile								
1 (most deprived)	2,241	25.6	60,467	24.4	1,034	23.4	28,305	22.8
2	1,900	21.7	52,041	21.0	965	21.8	25,677	20.7
3	1,663	19.0	49,067	19.8	844	19.1	24,803	20.0
4	1,462	16.7	44,111	17.8	774	17.5	22,871	18.4
5 (least deprived)	1,488	17.0	42,128	17.0	810	18.3	22,644	18.2
Child’s ATS quintile								
1 (most deprived)	1,453	16.6	45,846	18.5	734	16.6	23,399	18.8
2	1,908	21.8	54,023	21.8	921	20.8	26,672	21.5
3	1,917	21.9	53,032	21.4	1,039	23.5	26,628	21.4
4	1,821	20.8	49,811	20.1	906	20.5	24,900	20
5 (least deprived)	1,655	18.9	44,854	18.1	827	18.7	22,701	18.3
Child’s environment quintile								
1 (most deprived)	1,786	20.4	49,315	19.9	875	19.8	24,043	19.3
2	1,812	20.7	50,802	20.5	960	21.7	25,524	20.5
3	1,795	20.5	50,058	20.2	909	20.5	25,212	20.3
4	1,672	19.1	49,067	19.8	839	19.0	24,790	19.9
5 (least deprived)	1,681	19.2	48,572	19.6	844	19.1	24,731	19.9
**Hospitalisation** At least 1 day	7,353	84.0	120,195	48.5	3,684	83.2	60,613	48.8
1–7 days	3,709	42.4	104,045	42	1,908	43.1	51,999	41.8
8–28 days	2,330	26.6	13,361	5.4	1,165	26.3	7,187	5.8
29–168 days	1,235	14.1	2,684	1.1	585	13.2	1,359	1.1
>168 days (6 months)	79	0.9	105	0.04	26	0.6	68	0

Notes to Table 5: ATS, access to services; FSM, free school meals; WIMD, Welsh index of multiple deprivation; ‘anomalies’ refers to ‘major congenital anomalies’, as defined in EUROCAT guide 1.4 (List in S1 Appendix).

Reference categories for the components of the WIMD were the highest ranked or least deprived quintiles. For maternal deprivation, 1 is the highest rank or least deprived. For other indices, 5 is the highest rank or least deprived.

### Major congenital anomalies and the burden of illness

Proportionally fewer children were issued prescriptions for asthma, epilepsy, and cardiac conditions at age 16 than at age 11. Children with MaCA were more likely to be hospitalised (and for longer), and receive prescriptions for cardiac conditions, asthma and epilepsy, but not diabetes. Cardiac medicines were largely confined to children with congenital heart defects (CHD) and Down syndrome, and could not be analysed independently. A higher proportion of children with CHD and ‘any MaCA’ were treated for epilepsy. Most children with prolonged hospitalisation did not have MaCAs (Table 6).

**Table 6:** Co-morbidities amongst children with and without major congenital anomalies.

		Days hospitalised *	Asthma-related prescriptions	Prescriptions for epilepsy	Insulin prescriptions	Cardiac-related prescriptions
**Major congenital anomaly**	***N***	**Median** **(25**^**th**^**, 75**^**th**^ **centile)**	***N*** **Prescriptions >1 (%)**	***N* Prescriptions >1 (%)**	***N* Prescriptions >1 (%)**	***N* Prescriptions >1 (%)**
**Key Stage 2**						
ALL ANOMALIES (al1)	8,754	5 (1, 16)	1,691 (19.3)	177 (2.0)	16 (0.2)	359 (4.1)
Cleft lip ± cleft palate	177	11 (7, 18)	30 (17.0)	<10	<10	0
Cleft palate	153	7 (5,11)	29 (19.0)	<10	0	0
CHD (excluding severe CHD)	1,617	3 (1, 11)	316 (19.5)	23 (1.4)	<10	87 (5.4)
Oesophageal atresia	14	40 (17,65)	<10	0	0	0
Ano-rectal atresia and stenosis	21	14 (8, 23)	<10	0	0	0
Diaphragmatic hernia	26	18.5 (10,34)	<10	0	0	0
Gastroschisis	119	33 (22, 47)	22 (18.5)	0	0	0
Multi-cystic renal dysplasia	99	2 (1, 6.5)	20 (10.2)	<10	0	<10
Hypospadias	691	4 (2, 8)	126 (18.2)	<10	0	5
Spina bifida	22	10.5 (1, 33)	<10	<10	0	0
Limb reduction defects	70	4 (1, 9.8)	11 (15.7)	<10	0	0
Hydrocephalus	55	15 (5, 43)	<10	<10	0	<10
Craniosynostosis	99	7 (3, 12)	16 (16.2)	<10	0	0
Down syndrome	202	13 (4, 33)	49 (24.3)	<10	<10	30 (14.9)
Turner syndrome	27	5 (2, 19)	0	<10	0	<10
Severe CHD	383	21 (10, 48)	76 (19.8)	<10	0	124 (32.4)
Other anomalies	4,979	5 (1, 15)	966 (19.4)	124 (2.5)	<10	110 (2.2)
No anomaly	247,814	0 (0, 2)	39,962 (16.1)	888 (0.4)	383 (0.2)	172 (0.1)
**Key Stage 4**						
ALL ANOMALIES (al1)	4,427	5 (1, 15)	608 (13.7)	43 (1.0)	<10	104 (2.4)
Cleft lip ± cleft palate	78	10 (7, 19)	10 (12.8)	0	<10	0
Cleft palate	74	6 (4, 12)	12 (16.2)	<10	0	0
CHD (excluding severe CHD)	808	4 (1, 12)	112 (13.9)	<10	<10	30 (3.7)
Oesophageal atresia	<10	39 (15, 56)	<10	0	0	0
Ano-rectal atresia and stenosis	<15	12.5 (9, 23)	<10	0	0	0
Diaphragmatic hernia	15	18 (9, 40)	<10	0	0	0
Gastroschisis	43	30 (6, 46)	<10	0	0	0
Multi-cystic renal dysplasia	48	2 (1, 8.3)	<10	<10	0	0
Hypospadias	347	4 (2,9)	48 (13.8)	<10	0	0
Spina bifida	13	6 (0, 15)	<10	0	0	0
Limb reduction defects	35	3 (1, 7)	<10	0	0	0
Hydrocephalus	28	15.5 (5, 36)	<10	<10	0	<10
Craniosynostosis	45	7 (2, 13)	<10	0	0	0
Down syndrome	66	9.5 (3, 24)	13 (19.7)	<10	<10	<10
Turner syndrome	13	5 (3, 15)	0	<10	0	0
Severe CHD	183	21 (9, 44)	28 (15.3)	<10	0	37 (20.2)
Other anomalies	2,610	4 (1, 14)	350 (13.4)	28 (1.1)	<10	32 (1.2)
No anomaly	124,300	0 (0, 2)	14,586 (11.7)	301 (0.2)	148 (0.12)	42 (0.03)

Notes to Table 6: Lq, lower quartile (25th centile); Uq, upper quartile (75th centile); CHD, congenital heart defect; ‘anomalies’ refers to ‘major congenital anomalies, as defined in EUROCAT guide 1.4 (List in S1 Appendix). Insulin is the only medicine prescribed for type 1 diabetes in children.

*Days in hospital by 1st May in the year of the key stage assessment.

### Special Education Needs (SEN) provision

Children with major congenital anomalies were more likely to receive SEN provision than their peers, particularly the most intensive intervention, statementing (Table 7).

**Table 7:** Major congenital anomalies and type of special educational needs (SEN) provision.

	Children with major congenital anomalies *n*	%	Children without major congenital anomalies *n*	%	SEN provision rather than none OR (95% CI)
**KS2 SEN provision, age 11**					
None	4,945	56.5	181,776	73.4	1
Action plan	1,304	14.9	37,085	15	1.29 (1.21, 1.38)
Action plan plus	1,258	14.4	22,893	9.2	2.02 (1.90, 2.15)
Statement	1,247	14.2	6,060	2.5	7.56 (7.07, 8.09)
Total	8,754	100	247,814	100	
**KS4 SEN provision, age 16**					
None	2,745	62	95,564	76.9	1
Action plan	645	14.6	15,681	12.6	1.18 (1.08, 1.29)
Action plan plus	491	11.1	9,275	7.5	1.55 (1.41, 1.70)
Statement	546	12.3	3,780	3.0	4.49 (4.08, 4.93)
Total	4,427	100	124,300	100	

Notes to Table 7: OR, unadjusted odds ratio; CI, confidence interval; KS, key stage.

Children with major congenital anomalies, particularly Down or Turner syndromes, hydrocephalus, *spina bifida*, limb reduction, craniosynostosis, severe CHD (Table Ei in S1 Appendix), or eligible for FSM were more likely than those ineligible to receive SEN statements (aOR 3.17; 95% CI [3.00,3.34] at KS2, and aOR 3.97; 95% CI [3.70,4.26] at KS4) (Table Eii in S1 Appendix). Adjusting for deprivation made little difference to associations between MaCAs and statementing at KS2 (aOR 7.65; 95% CI [7.15,8.19]), and KS4 (aOR 5.06; 95% CI [4.58,5.60], Table Eii in S1 Appendix). Not all MaCAs were statistically significantly associated with statementing (oesophageal atresia, ano-rectal atresia, diaphragmatic hernia, and at KS4 only, gastroschisis, craniosynostosis, multicystic renal dysplasia), but numbers in cross-tabulations were low (Table Ei in S1 Appendix).

### Major congenital anomalies and educational attainment in mainstream state-funded schools

In this cohort, 93.4% (239,617/256,568) and 86.5% (222,011/256,568) children met academic standards at low- and high-levels at KS2; 94.3% (121364/128,727) obtained 5 GCSEs; and 55.78% (71,802/128,727) obtained 5 GCSEs grades C–A* with EWMS.

Major congenital anomalies (MaCA) were associated with significantly reduced likelihoods of attainment in unadjusted analyses, particularly in the low-level or ‘any pass’ model for KS2 (81.9% [7167/8754] with and 93% [232,450/247,814] without MaCAs [OR 0.30; 95% CI [0.28,0.32]]). Associations were less marked at KS4, particularly for 5 GCSEs grades C to A* with EWMS, which were attained by 46.8% (2070/44270) with and 56.1% (69,732/124,300) without MaCA (OR 0.69; 95% CI [0.65,0.73]). Inclusion of SEN provision in the regression models reduced, but did not eliminate, associations between MaCAs and achievement: aOR 0.72; 95% CI [0.66,0.78] at KS2 (any pass), and aOR 0.93; 95% CI [0.87,1.00] for 5 GCSEs C–A* with EWMS (Table 8, S1 Data). SEN provision mediated the association between MaCA and educational attainment (Text C in S1 appendix).

**Table 8:** Major congenital anomalies and educational attainment.

		Children with major congenital anomalies		Children without major congenital anomalies		Unadjusted	Adjusted as below*	Adjusted as below* plus SEN provision
***N***	**%**	***N***	**%**	**OR (95% CI)**	**aOR (95% CI)**	**aOR (95% CI)**
KS2	‘Pass’ (any)	7,167	81.9	232,450	93.8	0.30 (0.28, 0.32)	0.36 (0.34, 0.39)	0.72 (0.66, 0.78)
Age	no pass	1,587	18.1	15,364	6.2			
11	Total	8,754		247,814				
	‘Pass’ (high)	6,413	73.3	215,598	87.0	0.41 (0.39, 0.43)	0.48 (0.45, 0.51)	0.83 (0.77, 0.89)
	no pass	2,341	26.7	32,216	13.0			
	Total	8,754		247,814				
KS4	5 GCSEs							
Age	Attained	3,900	88.1	117,464	94.5	0.43 (0.39, 0.47)	0.48 (0.44, 0.53)	O.78 (0.70, 0.87)
16	Not attained	527	11.9	6,836	5.5			
	Total	4,427		124,300				
	5 GCSEs with EWMS grades C–A*							
	Attained	2,070	46.8	69,732	56.1	0.69 (0.65, 0.73)	0.73 (0.69, 0.78)	0.93 (0.87, 1.00)
	Not attained	2,357	53.2	54,568	43.9			
	Total	4,427		124,300				

Notes to Table 8: EWMS, English/Welsh, maths and science; GCSE, General Certificate of Secondary Education; OR, odds ratio; aOR, adjusted odds ratio; CI, confidence interval; KS, key stage; SEN, special educational needs.

*Adjusted for year of birth, sex, gestational age, FSM eligibility, access to services, physical environment, household income, maternal deprivation quintiles, hospital stay, medicated epilepsy, asthma, and type 1 diabetes, as in Table 10.

Children receiving SEN provision were less likely than their peers to attain prescribed national standards (Table 2), reflecting their additional needs. Differences in the proportion of children attaining national standards were greater between children with/without FSM eligibility than between those with/without MaCAs. For 5 GCSEs C–A* with EWMS, for children without SEN support, the ‘pass’ rate was 30% lower for children eligible for FSM, and 3.6% lower for children with MaCAs. Children with MaCAs receiving SEN action plans or ‘action plans plus’ were more likely to attain 5 GCSEs C–A* with EWMS than children without MaCAs receiving the same provision. SEN provision narrowed the gaps in attainment between children with/without MaCAs and those eligible/ineligible for FSM, only for this outcome, suggesting that SEN provision was a moderator only for the highest level of attainment (Table 9).

**Table 9:** Educational attainment and support: major congenital anomalies and free school meals (FSM) eligibility.

			No SEN		Action Plan		Action Plan plus		Statement	
			Pass/total *n*	% passed	Pass/total *n*	% passed	Pass/total *n*	% passed	Pass/total *n*	% passed
KS2 ′any pass’	MaCA	No	180,810 / 181,776	99.5	32,641 / 36,085	88.0	16,751 / 22,893	73.2	2,146 / 6,060	35.4
		Yes	4,901 / 4,945	99.1	1,121 / 1,304	86.0	876 / 1,258	69.6	271 / 1,247	21.7
		*Difference*		*0.4*		*2.1*		*3.5*		*13.7*
	FSM eligibility	No	160,041 / 160,647	99.6	24,848 / 27,415	90.6	12,707 / 16,282	78.1	1,714 / 4,618	37.1
		Yes	25,670 / 26,074	98.5	8,914 / 10,974	81.2	4,920 / 7,869	62.5	703 / 2,689	26.1
		*Difference*		*1.2*		*9.4*		*15.5*		*11.0*
KS2 ′high pass’	MaCA	No	177,085 / 181,776	97.4	25,658 / 37,085	69.2	11,609 / 22,893	50.7	1,241 / 6,060	20.5
		Yes	4,766 / 4,945	96.4	859 / 1,304	65.9	617 / 1,258	49.1	177 / 1,247	14.2
		*Difference*		*1.0*		*3.3*		*1.7*		*6.3*
	FSM eligibility	No	157,399 / 160,647	98.0	20,059 / 27,415	73.2	9,110 / 16,282	56.0	1,031 / 4,618	22.3
		Yes	24,452 / 26,074	93.8	6,458 / 10,974	58.9	3,116 / 7,869	39.6	387 / 2,689	14.5
		*Difference*		*4.2*		*14.3*		*16.4*		*7.9*
KS4 5 GCSEs	MaCA	No	93,780 / 95,564	98.1	14,475 / 15,681	92.3	7,010 / 9,275	75.6	2,161 / 3,780	57.2
		Yes	2,690 / 2,745	98.0	594 / 649	92.1	386 / 491	78.6	234 / 546	42.9
		*Difference*		*0.1*		*0.2*		*−3.1*		*14.3*
	FSM eligibility	No	86,111 / 87,267	98.7	11,728 / 12,446	94.2	5,259 / 6,515	80.7	1,721 / 2,748	62.6
		Yes	10,359 / 11,042	93.8	3,341 / 3,880	86.1	2,137 / 3,251	65.7	674 / 1,578	42.7
		*Difference*		*4.9*		*8.1*		*15.0*		*19.9*
KS4 5 GCSEs C–A* (EWMS)	MaCA	No	64,151 / 95,564	67.1	3,654 / 15,681	23.3	1,529 / 9,274	16.5	388 / 3,780	10.3
		Yes	1,743 / 2,745	63.5	171 / 645	26.5	114 / 493	23.2	42 / 546	7.7
		*Difference*		*3.6*		*−3.2*		*−6.7*		*2.6*
	FSM eligibility	No	61,479 / 87,267	70.5	3,434 / 12,446	27.6	1,367 / 6,515	21.0	357 / 2,748	13.0
		Yes	4,415 / 11,042	40.0	391 / 3,880	10.1	276 / 3,251	8.5	73 / 1,578	4.6
		*Difference*		*30.5*		*17.5*		*12.5*		*8.4*

Notes to Table 9: EWMS, English/Welsh, maths and science; GCSE, General Certificate of Secondary Education; FSM, free school meals; KS, key stage; MaCA, major congenital anomaly, as defined in EUROCAT guide 1.4 (List in S1 Appendix); SEN, special educational needs. All figures, including calculated differences, have been corrected to one decimal place. The denominator for each category is the sum of the number passing plus the number not passing.

Of the children with MaCAs and FSM eligibility, 2.60% (5/192) receiving SEN statements, and 34.64% (115/332) without SEN provision attained 5 GCSEs C–A* with EWMS. Of children with MaCAs ineligible for FSM, 10.45% (37/354) of those receiving statements and 67.45% (1628/2413) of those not receiving SEN provision achieved these GCSEs (Fig 4, Table Fi in S1 Appendix): a two to 4-fold difference.

**Fig 4:**
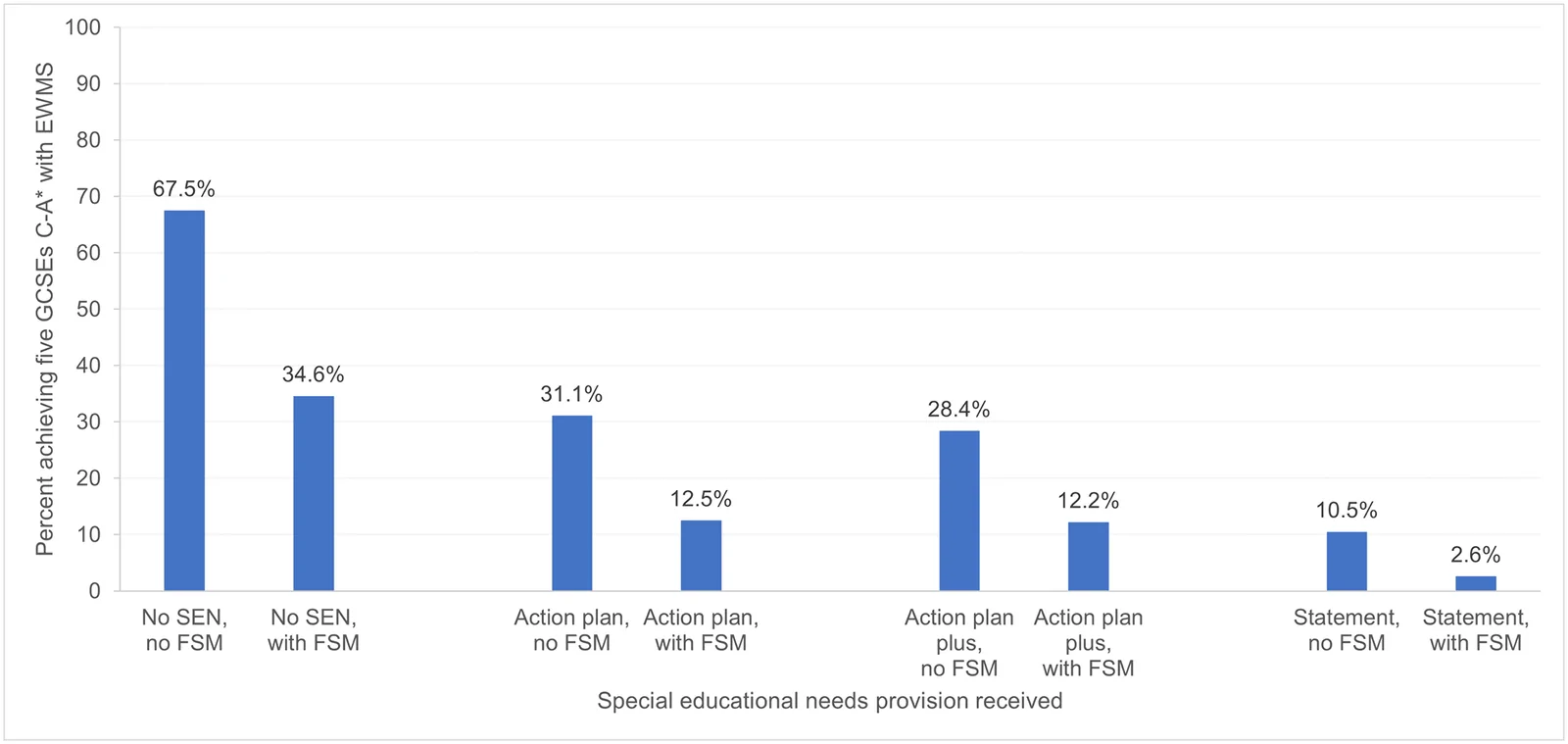
Children with major congenital anomalies, with and without FSM eligibility at each level of special educational needs provision, % achieving 5 GCSEs, C–A* with EWMS (*n* = 4,427). Note to Fig 4: FSM, free school meals; KS, key stage; SEN, special educational needs.

Differences between children eligible and ineligible for FSM were similar for children without MaCAs: amongst statemented children, 68/1318 (4.9%) of those eligible for FSM attained 5 GCSEs C–A* with EWMS, as did 320/2074 (13.4%) of those ineligible. Slightly (2%–6%) more children without than with MaCAs attained prescribed standards at each SEN level (Table 2, Fig 5, Table Fii in S1 Appendix).

**Fig 5:**
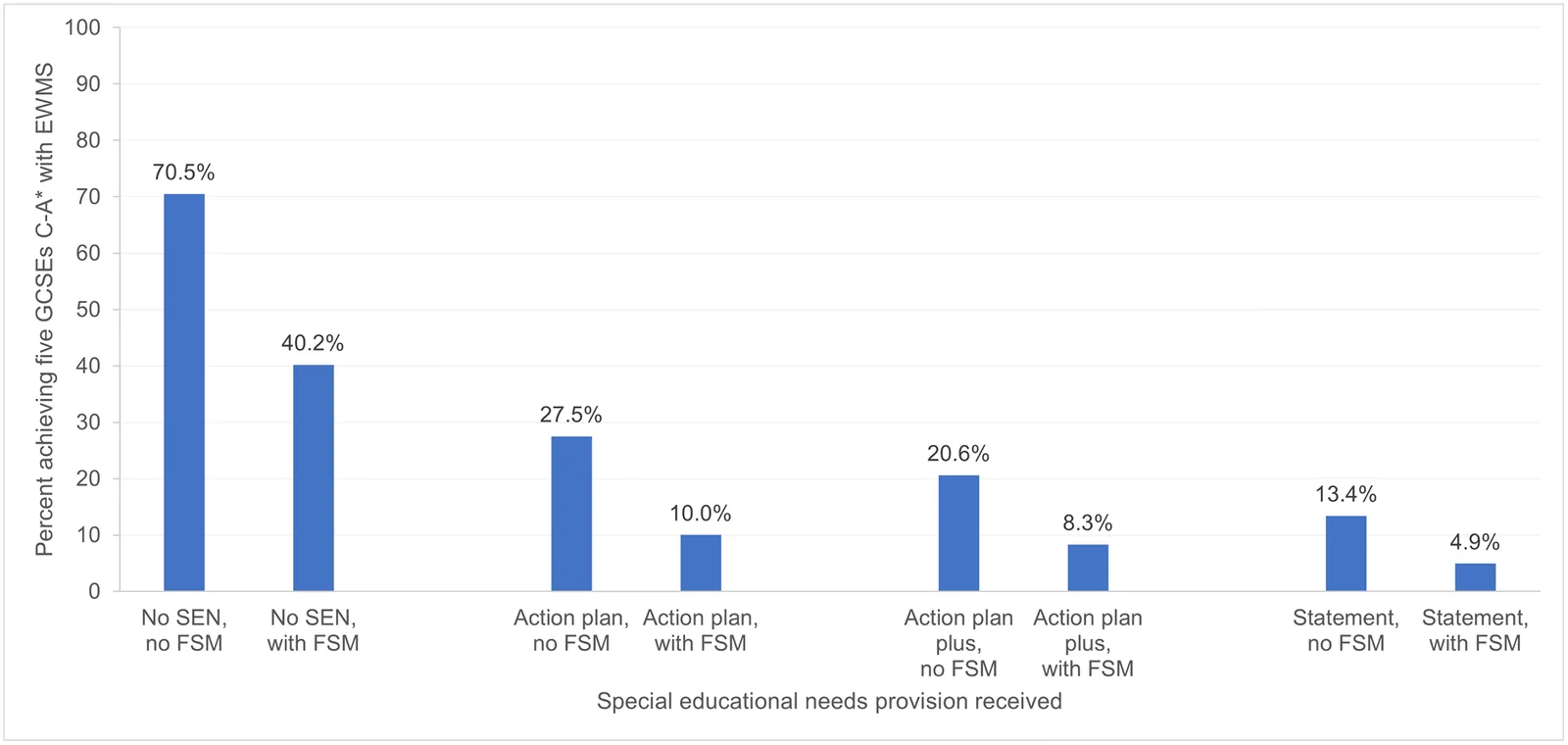
Children without major congenital anomalies, with and without FSM eligibility at each level of special educational needs provision, % achieving 5 GCSEs, C–A* with EWMS (*n* = 4,427). Note to Fig 5: EWMS, English/Welsh, maths and science; GCSE, General Certificate of Secondary Education; FSM, free school meals; KS, key stage; SEN, special educational needs.

### Educational attainment: Adjusted analyses

Adjusting for year of birth, preterm birth, sex, deprivation, hospitalisation, co-morbidities (as prescribed medicines), and SEN provision reduced the association between MaCAs and attainment: MaCAs were associated with statistically nonsignificant reduced attainment of 5 GCSEs C–A* with EWMS (aOR 0.93; 95% CI [0.87,1.00]), but associations remained statistically significant for other educational outcomes (Tables 8, 10). Results were similar when children with Down syndrome were removed from the analysis; the association between ‘any MaCA’ and 5 GCSEs C–A* with EWMS was statistically nonsignificant (aOR 0.94; 95% CI [0.88,1.01]) (Table G in S1 Appendix). Removal of SEN provision from the model strengthened the associations between educational attainment and MaCAs or epilepsy, but made little difference to associations with SES (Table H in S1 Appendix).

**Table 10:** Associations with educational attainment at KS2, age 11 (*n* = 256,568) and KS4, age 16 (*n* = 128,727).

		KS2, age 11		KS4, age 16	
		Lower-level pass	Higher level pass	Any 5 GCSEs	5 GCSEs C–A* with EWMS
		aOR (95% CI)	aOR (95% CI)	aOR (95% CI)	aOR (95% CI)
	Any major congenital anomaly	0.72 (0.66,0.78)	0.83 (0.77,0.89)	0.78 (0.70,0.87)	0.93 (0.87,1.00)
Birth year	1999	0.87 (0.81,0.94)	0.94 (0.88,0.99)	1.08 (1.00,1.18)	1.16 (1.12,1.21)
Reference 1998	2000	1.03 (0.95,1.11)	1.21 (1.14,1.28)	1.09 (1.00,1.19)	1.18 (1.14,1.23)
	2001	1.28 (1.18,1.38)	1.56 (1.47,1.66)	0.90 (0.83,0.98)	0.98 (0.94,1.02)
	2002	1.39 (1.28,1.50)	1.95 (1.83,2.07)	0.84 (0.78,0.92)	0.93 (0.89,0.97)
	2003	1.58 (1.46,1.72)	2.42 (2.27,2.57)		
	2004	1.75 (1.61,1.91)	2.77 (2.60,2.95)		
	2005	1.82 (1.67,1.98)	3.11 (2.91,3.31)		
	2006	1.89 (1.73,2.05)	3.36 (3.15,3.58)		
	2007	1.63 (1.49,1.79)	2.86 (2.66,3.07)		
Sex	Male	1.15 (1.10,1.19)	1.11 (1.07,1.14)	0.87 (0.82,0.92)	0.81 (0.79,0.83)
Gestational age	Pre-term 32–36 + 6 weeks	1.01 (0.86,1.18)	0.84 (0.74,0.96)	1.56 (1.22,2.01)	0.94 (0.81,1.08)
Reference: term	Very pre-term <32 weeks	0.88 (0.82,0.95)	0.91 (0.86,0.97)	1.07 (0.96,1.18)	1.02 (0.97,1.08)
	FSM eligibility	0.53 (0.51,0.55)	0.54 (0.52,0.55)	0.43 (0.40,0.45)	0.39 (0.38,0.41)
Access to services	ATS1 – most deprived quintile	1.03 (0.97,1.11)	0.98 (0.94,1.03)	0.92 (0.83,1.01)	0.89 (0.85,0.93)
	ATS2	0.91 (0.86,0.97)	0.94 (0.90,0.98)	0.90 (0.82,0.98)	0.86 (0.83,0.90)
	ATS3	0.89 (0.84,0.95)	0.93 (0.89,0.98)	0.85 (0.78,0.93)	0.87 (0.84,0.91)
	ATS4	0.98 (0.92,1.05)	0.97 (0.93,1.02)	0.88 (0.81,0.96)	0.89 (0.86,0.93)
	Access to services – least deprived quintile	1	1	1	1
Physical	ENV1 environment – most deprived quintile	1.12 (1.05,1.18)	1.02 (0.97,1.06)	0.92 (0.85,1.01)	1.03 (0.99,1.07)
Environment	ENV2	1.13 (1.06,1.20)	1.06 (1.02,1.11)	0.94 (0.87,1.03)	0.98 (0.94,1.02)
	ENV3	1.10 (1.03,1.17)	1.03 (0.99,1.08)	0.95 (0.87,1.03)	0.98 (0.94,1.02)
	ENV4	1.11 (1.05,1.18)	1.07 (1.02,1.12)	1.02 (0.93,1.11)	1.01 (0.97,1.05)
	Environment 5 – least deprived quintile	1	1	1	1
Child’s household’s	INC1 income – most deprived quintile	0.65 (0.60,0.70)	0.67 (0.64,0.72)	0.54 (0.48,0.60)	0.40 (0.38,0.42)
income at	INC2	0.70 (0.65,0.76)	0.75 (0.70,0.79)	0.61 (0.55,0.69)	0.48 (0.46,0.50)
assessment	INC3	0.80 (0.74,0.87)	0.81 (0.76,0.86)	0.74 (0.66,0.83)	0.59 (0.56,0.62)
	INC4	0.94 (0.86,1.02)	0.90 (0.85,0.96)	0.86 (0.77,0.97)	0.74 (0.70,0.77)
	5 Household income – least deprived quintile	1	1	1	1
Maternal	1 – least deprived quintile	1	1	1	1
Deprivation	2	0.99 (0.91,1.08)	0.98 (0.92,1.04)	0.90 (0.79,1.01)	0.85 (0.81,0.89)
At birth	3 – Central quintile	0.85 (0.78,0.92)	0.85 (0.80,0.90)	0.82 (0.73,0.92)	0.72 (0.69,0.76)
	4	0.77 (0.71,0.84)	0.81 (0.76,0.86)	0.76 (0.67,0.85)	0.65 (0.62,0.68)
	5 – most deprived quintile	0.73 (0.68,0.79)	0.75 (0.70,0.79)	0.63 (0.56,0.70)	0.52 (0.50,0.55)
Co-morbidities	Asthma >1 prescription	1.01 (0.96,1.06)	1.00 (0.96,1.04)	1.04 (0.96,1.13)	0.99 (0.95,1.03)
	Epilepsy >1 prescription	0.48 (0.41,0.57)	0.56 (0.47,0.66)	0.78 (0.58,1.05)	0.60 (0.45,0.80)
	Insulin >1 prescription	1.78 (1.18,2.80)	2.23 (1.59,3.18)	1.10 (0.59,2.24)	1.11 (0.76,1.61)
SEN	A action plan	0.05 (0.04,0.05)	0.06 (0.06,0.07)	0.29 (0.27,0.31)	0.17 (0.16,0.18)
	P action plan plus	0.02 (0.02,0.02)	0.03 (0.03,0.03)	0.08 (0.08,0.09)	0.13 (0.12,0.14)
	S statement	0.00 (0.00,0.00)	0.01 (0.01,0.01)	0.03 (0.03,0.03)	0.07 (0.06,0.07)
Hospitalisation	Each day hospitalised	1.00 (1.00,1.00)	1.00 (1.00,1.00)	1.00 (1.00,1.00)	1.00 (1.00,1.00)
Model	-2LL (log likelihood)	62,016	100,839	28,188	88,293
Parameters*	Nagelkerke R^2^	40.2	37.9	26	18.8
	Goodness of fit† χ^2^ *†*	0.4	0.38	0.26	0.19

Notes to Table 10: ATS, access to services; ENV, physical environment; EWMS, English/Welsh plus maths and science; FSM, free school meals eligibility; GCSE, General Certificate of Secondary Education; KS, key stage; INC, household income levels; SEN, special educational needs; aOR, adjusted odds ratio; CI, 95% confidence intervals; VIF, variance inflation factor.

Odds ratios were adjusted for: sex, gestational age, year of birth, deprivation, hospitalisation, medicated co-morbidities, and SEN provision. Including interaction terms in the models widened confidence intervals, but did not change the findings (Text F in S1 Appendix, S1 Data has the models).

Hospitalisation for each day is the only continuous variable in the analysis. All other variables are categorical, comparing attainment for children with and without each variable.

Reference categories for the components of the WIMD 2014 were the least deprived quintile (corresponding with highest rank) [80]. For maternal deprivation, 1 is the least deprived quintile. For other indices, 5 is the least deprived quintile.

VIF was <3 for all models. Income and area-based measures of deprivation were associated, but there was no collinearity between FSM eligibility or gestation age and area-based measures of deprivation. By including all parameters, these models identify the strongest associations with educational attainment.

There were no data at age 16 for children born 2003–2007

*Reported for model comparison.

† as described in [92]. Values for χ^2^ were statistically nonsignificant.

Birth year was associated with educational attainment, mainly for teacher-assessed KS2 (Table 10). Removing year of birth from the analyses and treating year of birth as a continuous variable did not change the findings. Over time, more children attained prescribed standards at age 11, but fewer attained GCSEs (Tables I, J in S1 Appendix).

For all children, the most marked associations with attainment at KS2 (age 11) were SEN provision, epilepsy, and FSM eligibility; the association with any MaCA was weaker, particularly at the higher level. At KS4 (age 16), the strongest associations with 5 GCSEs C–A* with EWMS, were SEN statements (aOR 0.07; 95% CI [0.06,0.07]), FSM eligibility (aOR 0.39; 95% CI [0.37,0.41]), and epilepsy (aOR 0.60; 95% CI [0.45,0.80]); the association with MaCAs was weaker (aOR 0.93; 95% CI [0.87,1.00]). Area-based markers for deprivation, including ‘access to services’, were associated with lower attainment, particularly for 5 GCSEs C–A* with EWMS (Tables 10, H in S1 Appendix).

Educational attainment followed WIMD income deprivation quintiles: 29.7% (307/1034) children with and 38,4% (10,875/28,305) without MaCAs from the most deprived income quintile attained 5 GCSEs C–A* with EWMS; in the least deprived quintile, figures were 67.6% (547/810) and 75.0% (16,978/22,644). Children with MaCAs in the least deprived income quintiles were at least as likely to attain 5 GCSEs C–A* with EWMS as unaffected children in the more deprived quintiles. Children without MaCAs from the most deprived quintile were less likely to attain these GCSEs than children with MaCAs from quintiles 1–3. Differences in attainment rates between children with and without MaCAs ranged from 7.4% to 10.2%, with the largest difference in the central quintile (Fig 6) (Table K in S1 Appendix).

**Fig 6:**
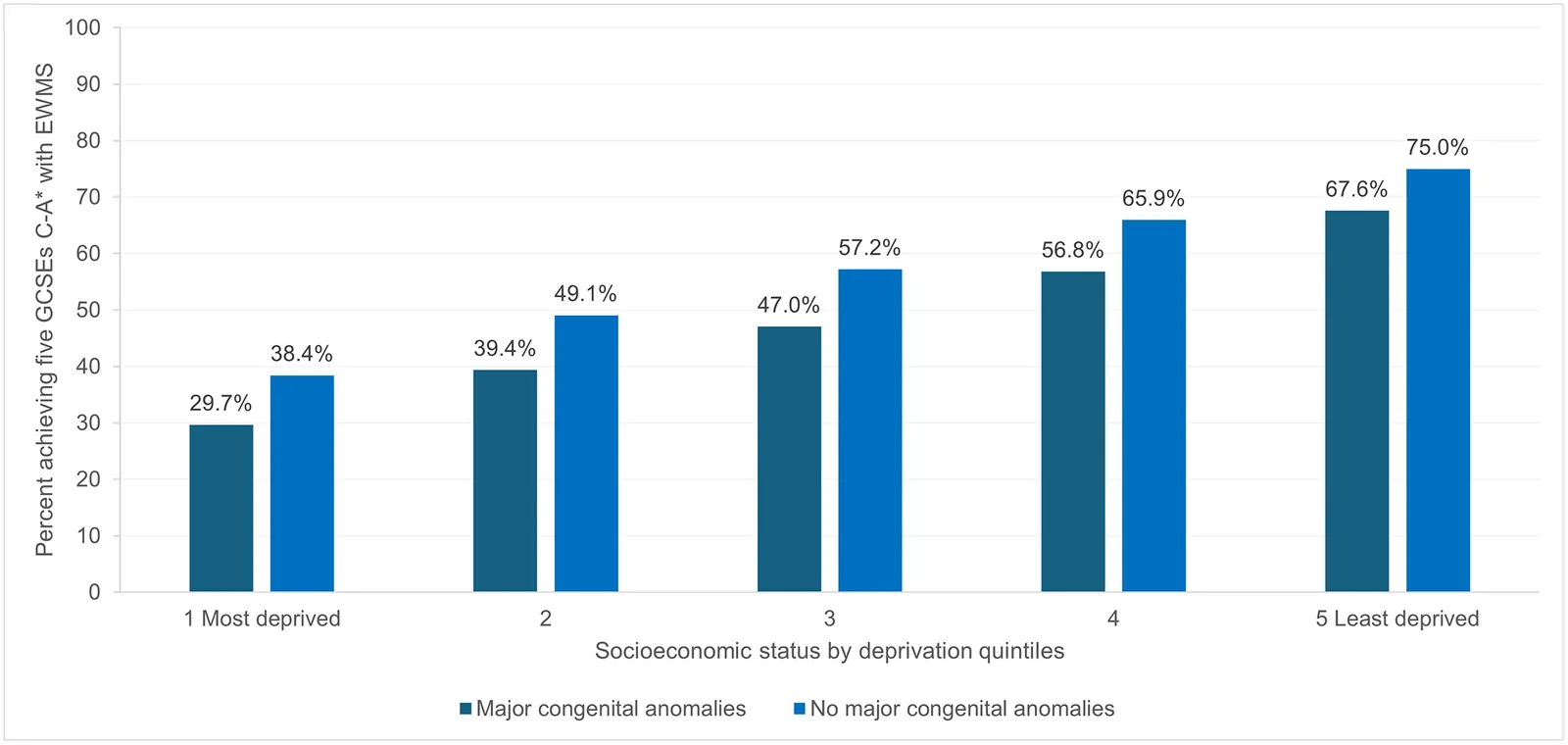
Achieving 5 GCSEs at grades C–A* with EWMS: income deprivation and major congenital anomalies. Notes to Fig 6: Deprivation recorded from the WIMD income domain 2014. WIMD, Welsh Index of Multiple Deprivation.

Preterm birth was not associated with attainment at KS4, when SEN was accounted, but an association was seen at KS2. Hospitalisation (as each day admitted), medicated asthma and type 1 diabetes did not significantly affect outcomes in fully adjusted models. Boys were more likely than girls to achieve national standards (Table 2) at KS2, but only when SEN was accounted. Girls were more likely to obtain GCSEs (Tables 10, H in S1 Appendix); differences were larger for some MaCAs (Tables Li, Lii in S1 Appendix). Over time, associations between educational attainment and SEN or FSM changed little at KS4, but intensified at KS2 (Tables M, N in S1 Appendix). Negative associations between attainment and FSM eligibility increased: for KS2 ′high-level pass’ from OR 0.33; 95% CI [0.30,0.35] in birth year 1998 to OR 0.28; (95% CI [0.25,0.31] in 2007; for ‘low-level pass’ from OR 0.30; 95% CI [0.28, 0.33] for birth year 1998 to OR 0.24; 95% CI [0.21,0.28] for 2007.

Inclusion of interaction terms left findings essentially unchanged; confidence intervals widened slightly, but the sample size precluded further interpretation (S1 Data). Most interaction terms, including MaCAs were not statistically significant. Exceptions included: the positive association between 5 GCSEs C–A* with EWMS and ‘SEN school-support combined with MaCAs’; and the negative association between any 5 GCSEs and ‘statementing combined with MaCAs’. The interaction term ‘SEN with FSM’ was statistically significant at all assessments except 5 GCSEs C–A* with EWMS; ‘SEN combined with income’ was statistically significant for some outcomes. In the main, ‘SEN combined with hospitalisation’ was not significant. Of the WIMD components, only income interacted with SEN, and none interacted with MaCAs (S1 Data). Findings were not materially altered in sensitivity analyses exploring missing FSM eligibility data (Table O in S1 Appendix).

### Isolated major congenital anomalies and educational attainment

Children with some, not all, MaCAs were less likely to attain prescribed standards. In unadjusted analyses, educational attainment was associated with Down syndrome, hydrocephalus, CHD, hypospadias or severe CHD, and with gastroschisis and craniosynostosis for most outcomes (Table P in S1 Appendix). After adjusting for SEN provision, FSM eligibility, co-morbidity and hospitalisation, at KS2 (age 11), associations with CHD, multicystic renal dysplasia, Down syndrome and ‘other MaCAs’ remained statistically significant. Down syndrome, gastroschisis and hypospadias were associated with lower rates of attainment of 5 GCSEs C–A* with EWMS, but associations were lost when boys and girls were analysed separately (Table Li, Lii in S1 Appendix). Association with asthma was only apparent at age 16 (KS4). Each day’s hospitalisation was negatively associated with educational outcomes at age 11, but this was attenuated by age 16. Association with FSM eligibility was particularly pronounced for attainment of 5 GCSEs C–A* with EWMS; only Down syndrome and SEN provision were stronger predictors. Type 1 diabetes (as insulin use), Turner syndrome, limb reduction, diaphragmatic hernia, *spina bifida,* hydrocephalus, craniosynostosis*,* and ano-rectal atresia did not adversely affect outcomes at age 16, and *spina bifida had some positive associations at age 11,* but numbers with each MaCA in mainstream schools were low. At both stages, epilepsy was more strongly associated with unattained standards than most MaCAs (Table 11). Removing FSM from models reduced most associations, particularly with asthma, and one day in hospital was no longer statistically significant (Table Q in S1 Appendix).

**Table 11:** Education outcomes and major congenital anomalies at KS2, age 11 (*n* = 256,568) and KS4, age 16 (*n* = 128,727), adjusted analysis.

	KS2, age 11		KS4, age 16	
	Lower level pass	Higher level pass	Any 5 GCSEs	5 GCSEs C–A* with EWMS
	aOR (95% CI)	aOR (95% CI)	aOR (95% CI)	aOR (95% CI)
No major congenital anomalies (reference)	1	1	1	1
Cleft lip ± cleft palate	1.43 (0.84,2.57)	1.12 (0.73,1.73)	1.78 (0.72,5.47)	0.75 (0.45,1.27)
Cleft palate	1.43 (0.77,2.84)	1.17 (0.71,1.97)	1.78 (0.65,6.31)	0.71 (0.42,1.20)
CHD (excluding severe CHD)	0.77 (0.63,0.94)	0.75 (0.64,0.89)	0.88 (0.67,1.18)	0.98 (0.84,1.15)
Oesophageal atresia	0.34 (0.04,4.27)	0.40 (0.08,3.04)		
Ano-rectal atresia and stenosis	1.21 (0.19,11.16)	0.74 (0.18,3.78)		1.32 (0.42,4.74)
Diaphragmatic hernia	2.07 (0.40,16.23)	1.58 (0.41,7.30)		1.41 (0.45,4.85)
Gastroschisis	0.56 (0.31,1.06)	0.75 (0.44,1.29)	0.85 (0.30,3.14)	0.49 (0.24,0.96)
Multi-cystic renal dysplasia	0.40 (0.19,0.92)	0.48 (0.26,0.93)	1.40 (0.45,6.21)	0.75 (0.40,1.44)
Hypospadias	0.86 (0.64,1.16)	0.87 (0.69,1.11)	1.01 (0.66,1.58)	0.77 (0.60,0.98)
Hypospadias with boys only	0.82 (0.62,1.11)	0.85 (0.67,1.08)	1.05 (0.69,1.65)	0.84 (0.66,1.07)
Spina bifida	4.80 (1.11,33.28)	1.62 (0.46,5.75)	0.36 (0.07,2.14)	0.58 (0.15,2.15)
Limb reduction defects	1.70 (0.73,4.35)	1.99 (0.96,4.30)	1.87 (0.57,8.50)	1.80 (0.80,4.11)
Hydrocephalus	1.09 (0.53,2.32)	1.45 (0.72,2.89)	0.96 (0.36,3.05)	0.43 (0.13,1.20)
Craniosynostosis	0.66 (0.32,1.41)	0.65 (0.34,1.25)	0.44 (0.17,1.35)	1.20 (0.61,2.41)
Down syndrome	0.03 (0.01,0.06)	0.02 (0.00,0.07)	0.06 (0.02,0.13)	0.09 (0.01,0.44)
Turner syndrome	1.81 (0.58,5.89)	4.11 (1.37,13.07)		1.00 (0.27,3.72)
Severe CHD	1.61 (1.10,2.40)	1.20 (0.88,1.65)	0.79 (0.45,1.43)	0.77 (0.55,1.07)
Other major congenital anomalies	0.71 (0.65,0.79)	0.85 (0.78,0.93)	0.86 (0.75,0.99)	1.02 (0.93,1.12)
FSM eligibility	0.45 (0.43,0.46)	0.46 (0.45,0.48)	0.35 (0.33,0.37)	0.29 (0.28,0.30)
Asthma >1 prescription	1.14 (1.09,1.20)	1.25 (1.20,1.30)	0.97 (0.89,1.04)	0.92 (0.88,0.95)
Epilepsy>1 prescription	0.52 (0.44,0.61)	0.65 (0.55,0.77)	0.74 (0.55,1.01)	0.56 (0.42,0.75)
Insulin >1 prescription	2.25 (1.46,3.63)	2.81 (2.00,4.05)	1.30 (0.68,2.77)	1.25 (0.87,1.80)
SEN A action plus	0.05 (0.04,0.05)	0.07 (0.06,0.07)	0.27 (0.25,0.29)	0.16 (0.16,0.17)
SEN P action plan plus	0.02 (0.02,0.02)	0.03 (0.03,0.03)	0.08 (0.07,0.08)	0.12 (0.11,0.12)
SEN S statemented	0.00 (0.00,0.00)	0.01 (0.01,0.01)	0.03 (0.03,0.03)	0.07 (0.06,0.07)
Hospitalisation, each day	1.00 (1.00,1.00)	1.00 (1.00,1.00)	1.000 (1.00,1.00)	1.00 (1.00,1.00)
Model parameters*				
−2LL (log likelihood)	61,634	100,544	28,066	880,243
Nagelkerke R^2^	39.2	35.6	25	15.6
Goodness of fit† c^2^ *†*	0.39	0.36	0.25	0.16

Notes to Table 11: CHD, congenital heart defect; NA, not applicable; SEN, special educational needs; EWMS, English/Welsh plus maths and science; FSM, free school meals eligibility; GCSE, General Certificate of Secondary Education; KS, key stage; SEN, special educational needs; aOR, adjusted odds ratio; CI, 95% confidence intervals; VIF, variance inflation factor; WIMD, Welsh Index of Multiple Deprivation.

Adjusted for: SEN, co-morbidities, hospitalisation, income as FSM. Not adjusted for (due to low numbers in categories): year of birth, pre-term birth, sex, components of WIMD.

Hospitalisation for each day is the only continuous variable in the analysis. All other variables are categorical, comparing attainment for children with and without each variable.

Low numbers for some major congenital anomalies (Table 4) precluded inclusion in the table. These children were included in the ‘other major congenital anomalies’ category. Low numbers in some remaining major congenital anomalies resulted in wide confidence intervals, and affected the stability of the model. Down syndrome had a stronger association with outcomes than other major congenital anomalies, and might be considered an ‘outlier’. Between 5–10 children with Down syndrome attained any level, and <5 attained the higher levels in both key stages.

Data were re-analysed excluding girls, and associations with hypospadias were no longer statistically significant (reported above) (Table Li in S1 Appendix).

VIF was <2 for all models, indicating minimal collinearity.

*Reported for model comparison.

† as described in [92]. Values for χ^2^ statistically nonsignificant.

The low number of children with oesophageal atresia preclude reporting of results.

At KS4 (age 16), children with diaphragmatic hernia, Down syndrome, hydrocephalus, Turner syndrome, oesophageal atresia, *spina bifida*, limb reduction defects and severe CHD were the most likely to receive SEN statements (Table Ei in S1 Appendix), but the children least likely to achieve 5 GCSEs C–A* with EWMS were those with Down syndrome, gastroschisis and hypospadias (Table 11).

When boys and girls were modelled separately with fewer covariates, only Down syndrome in boys made a statistically significant negative difference at KS4 5 GCSEs C–A* with EWMS. FSM remained the strongest predictor, followed by epilepsy and asthma. Girls with limb reductions were likely to attain these GCSEs (Tables Li, Lii in S1 Appendix).

In analyses stratified by each category of SEN provision, only when no SEN support was allocated were asthma, gastroschisis, hypospadias, and CHD associated with reduced chances of 5 GCSEs C–A* with EWMS. For all outcomes, the FSM association with attainment was stronger amongst children not receiving SEN provision. Epilepsy was strongly associated with attainment amongst children with statements (Table R in S1 Appendix).

## Discussion

The children least likely to attain prescribed educational standards (Table 2) lived with deprivation [80] and major congenital anomalies (MaCAs) (Figs 4,6). Educational attainment at age 16 was more strongly associated with deprivation (as measured by WIMD quintiles and FSM) than MaCAs or comorbidities, when hospitalisation, sex, preterm birth, year of birth, and SEN provision were included in analyses. Accounting for SEN provision in adjusted analyses narrowed, but did not close, the attainment gap between children with and without MaCAs (Tables 8, 9, H in S1 Appendix). In England, the differences in attainment between children with and without SEN are decreasing [93], but, in Wales, the association between SEN provision and attainment intensified over time (Table M in S1 Appendix). Meanwhile, the disparity between those eligible and ineligible for FSM is increasing (Table N in S1 Appendix) [61,94].

FSM eligibility, as a measure of individual-level deprivation, had a stronger, if atheoretical [95], association with educational attainment than area-based indices [81,95]. Children with poor access to services (ATS) were less likely to obtain GCSEs at age 16, highlighting the impact of rurality on educational attainment [96], not reflected in standard deprivation indices [97] and FSM [98,99].

Not all MaCAs statistically significantly detracted from performance in adjusted models (Table 11). As elsewhere, at various younger ages, MaCAs [28,100] or CHD [22,33,34] were negatively associated with educational attainment. Associations between *spina bifida*, craniosynostosis, or diaphragmatic hernia and lower educational attainment reported previously were not confirmed using Wales’ teacher-assessed criteria [18,20,22,24,26,29,31,32,101,102]. Associations with orofacial clefts [14,20,22,26,27] were confirmed in unadjusted, but not adjusted models (Tables 11, P in S1 Appendix).

At age 16, observations for children with *spina bifida,* diaphragmatic hernia, and CHD were consistent with earlier reports [22,103–106], with the *caveat* that, whilst many children with *spina bifida* do not complete mainstream schooling, those that do achieve the same standards as their contemporaries [101]. Findings regarding limb reduction defects, hydrocephalus, oesophageal atresia, and Turner syndrome resonate with earlier work [100,107,108], whilst findings on ano-rectal atresia and stenosis, and multicystic renal dysplasia contrast [100,109,110]. Disparities in attainment had narrowed for most children with MaCAs by the end of secondary schooling, age 16 [111,112], as for other conditions in Wales [113]. However, disparities across the socioeconomic spectrum increased, as elsewhere [114].

Epilepsy was associated with lower attainment, as in Scotland [44]. Adjusted models (including SEN provision) found no associations between medicated asthma [45–48] or diabetes [49,50] and educational attainment. When some deprivation parameters, including environmental conditions, were not accounted, asthma was associated with attainment at age 16 (Table 11), particularly for children without SEN provision (Table R in S1 Appendix). We were unable to separate illnesses, including seizures and any underlying brain injury, from their *sequelae,* including school absence and medicines, whether sedative or stimulant. Associations between preterm birth and educational attainment [115] were not apparent when SEN provision was accounted; preterm birth frequently coincides with MaCAs [116] (Tables 5, 10, H in S1 Appendix).

The cumulative risks associated with deprivation and MaCAs markedly reduced children’s chances of achieving the 5 GCSEs needed to access many higher education courses, despite SEN support (Figs 4,6). Childhood ill-health compounded the effects of deprivation [56]; children with MaCAs spent more time in hospital [42], and were more likely to be prescribed medicines for epilepsy, asthma and heart conditions (Tables 5,6). Accounting for both SEN provision and deprivation removed the statistical significance of the association between time in hospital and educational attainment reported elsewhere [32,117]. The detrimental effects of hospitalisation [110] may be mitigated if social and cultural capital are available to support learning [55,118].

Maternal ill-health, obesity, stress, alcohol or illicit drug use are all associated with both deprivation [119] and congenital anomalies [120,121], intensifying disadvantage. Long-term childhood deprivation alone depletes social capital, with profound inter-generational consequences. Deprivation and low socioeconomic status are associated with stress, inadequate stimulation and poor nutrition, all of which adversely affect brain development [122,123]: abnormalities in frontal and temporal lobes and the hippocampus may account for 15%–20% of educational attainment deficits between ages 4 and 22 [124]. Deficits may be exacerbated by: hypoxia induced by cardiac or respiratory congenital anomalies or surgical anaesthesia [7,125]; anxiety and depression [8]; autism [9], particularly for children with CHD [8,126]; or corticosteroid therapy [127]. Autism and intellectual delay are associated with CHD [128], long-term conditions [129] or metabolic problems secondary to parenteral nutrition (for gastro-intestinal congenital anomalies) or formula feeding (for children with cleft palate or prolonged hospitalisation) [130,131]. The absence of overall association between WIMD at birth and major congenital anomalies, as previously reported [132], runs counter to reports elsewhere [133–137]; this may be attributed to associations between maternal age and SES in Wales [138].

Children with the least favourable educational outcomes were not always the most likely to receive the highest level of in-school support (Tables Ei, Eii in S1 Appendix). For some visible MaCAs, such as Turner syndrome [107] or limb reduction defects, additional support may have been instrumental in raising achievement to the level of contemporaries by age 16. We do not know whether the same level of provision might be equally effective for children with MaCAs that are less visible after surgery, such as gastroschisis or hypospadias. FSM eligibility did not attract the same investment in SEN as major congenital anomalies, despite being more strongly associated with nonattainment of 5 GCSEs C–A* with EWMS (Tables 9–11, Eii in S1 Appendix).

To our knowledge, this is one of the largest population cohorts examining educational attainment, whilst accounting for holistic life experience: educational support, MaCAs, preterm birth, sex, long-term health (epilepsy, diabetes, and asthma), hospitalisation, deprivation at individual and area level for mother and child, access to services, and the environment. Analyses of a composite variable of ‘all EUROCAT major congenital anomalies’ [70,72] minimises uncertainty or obfuscation over coding and classification of disability and perceptions of disability [139]. Analyses of individual MaCAs suggest insights into contrasts between visible and less visible MaCAs, but low numbers introduce uncertainty [75]. We used individual components of the WIMD score: WIMD has elements relating to education at KS2 and KS4, whose inclusion would have introduced a circular argument [68,140]. Whole-population data ensures absence of volunteer, and, therefore, collider bias. As with all observational research, no causation can be inferred.

Despite the strength of associations between educational attainment, deprivation and MaCAs, generalisation to unobserved populations or to post-pandemic years, is predicated on logical, not statistical, inference [141,142]. Education assessments were disrupted during the COVID-19 pandemic, and, currently, there are no more recent education data available. The KS2 teacher assessment has been reviewed, but there are no reasons to question its internal validity [143]; publication of KS2 results ceased in 2018/19 [144].

This paper describes one European country, where child poverty rates, at 27%–36%, are higher than elsewhere in the UK [60] and most OECD countries [145]. However, funding for schools in deprived areas is proportionately lower than in England [146], and expenditure varies between £1,000 and £2,000 per child *per annum* between the 22 local education authorities [147]. The reported prevalence of major congenital anomalies is higher than elsewhere in the UK: for example in 2021, England reported major congenital anomalies in 235 per 10,000 (95% CI [231,239]), and Wessex in 246 per 10,000 (95% CI [229,265]) total births [133], whereas Wales reported 319 per 10,000 (95% CI [299,341]) [148]. We do not know whether this discrepancy is due to better case finding or worse air quality [149] in the more deprived communities [150–152], or more poverty [60,145] or greater use of medicines in pregnancy [132,153].

Over 80% of children were linked to their education records. The Pupil Level Annual School Census does not hold data on most independent and special schools; therefore, many children educated privately, at home, outside ‘mainstream’ schools or outside Wales were lost to the study. During primary school, some 2% of children are educated privately and 4.3% in special schools [117]. Incomplete data on special schools disproportionately excludes children with severe disabilities and behaviour difficulties. Provision for some children needing specialist input (for example, for blindness or profound deafness) is in England, and data are lost to Wales. However, the MaCAs included represent a range of disability, and indicated conditions that might be targeted for additional support (gastroschisis, hypospadias). Including prescription data reduced the size of the cohort, but not its representativeness (Text G in S1 Appendix) [85]. Missing data were largely confined to the outcome variable and missing data for other variables were <5% [154]. Since imputation may underestimate variability whilst never fully compensating for missing data in cohort studies [155], only exploratory imputations were undertaken. This complete case analysis is not transferable to children outside mainstream education.

Data on school attendance, social care interventions [156], ethnicity, breastfeeding [157], multiple births, autism, ADHD, mental health, cystic fibrosis, perinatal complications, maternal ill-health, medication use or illicit drug use [158] were not included [65]. The SAIL Databank does not hold information on fathers, behaviour disturbance or perceptions of bullying, stress or isolation. Intellectual disability is poorly recorded in electronic records, and associations with major congenital anomalies, and multiple major congenital anomalies [159], could not be explored independently of SEN provision. Primary care records do not document medication adherence; where repeat prescriptions are issued, nonadherence is less likely [160]. To mitigate the difficulties of identifying all illness in electronic health records, and take some account of school attendance, we accounted for time spent in hospital. School attendance may be a mediating covariate [106], and time in hospital accounts for a small proportion of absence. We adjusted for the three most common long-term childhood physical health problems [43,161]. Rarer conditions were unaccounted, including cerebral palsy or injuries. By using proxies for unmeasured covariates, such as attendance and ill-health outside the three named conditions, we risked overadjustment by controlling for intermediate variables or mediators [162]. Our explanatory analyses aimed to identify modifiable risk factors, where interventions might be targeted; propensity score matching might have obscured risk factors or left variables excluded from the propensity score unbalanced, confounding estimates [163]. EUROCAT does not assess severity of lesions, but this may not be the only determinant of need [128]. The heterogeneous categories ‘all major congenital anomalies’ and ‘other major congenital anomalies’ include children with different degrees of disability, which we were unable to explore.

EUROCAT coding gives ‘cleft lip +/− cleft palate’ and ‘cleft palate’, with no ‘cleft lip only’ category. We are, therefore, unable to report on isolated cleft lip, a condition for which there is uncertainty regarding school performance [22]. EUROCAT excludes minor congenital anomalies; however, most do not cause long-term disability, and their effects may be less pervasive.

Achieving the goals of ‘no child left behind [164], no community left behind’ will entail identifying and ***triaging*** children at risk from compounded socioeconomic and biological disadvantage, ensuring they are not overlooked in service re-organisation [62], and expediting intervention [165,166]. Differences between the most and least deprived [80] are unsurprising [146,167]: it is the 2- to 4-fold magnitude of the differences that should stimulate change. Allocated support, whilst beneficial, is insufficient for children with MaCAs from deprived backgrounds to match their contemporaries and for children from deprived quintiles [80] without MaCAs to match children with MaCAs from less deprived households. Routes to educational attainment are needed to counter the increasing impact of poverty [168,169], low SES [165], and disability, but only intensive programmes have been successful [170,171].

Children with physical ill-health need holistic specialist care to minimise pain, sensory impairment, seizures or hypoxic episodes and compensate for learning difficulties, time spent in hospital [129,172,173], and the stress of living with deprivation with poor access to services (ATS) [124]. However, the help needed [35] is not always available or accessible due to time and access (ATS) constraints [174]. Multi-agency provision from education, social and health services, has not prevented deprivation, isolation and disability generating a profoundly disadvantaged subgroup. Exploration of a unified, equitable, expert service throughout school to employment is warranted [44,158,172,175–177].

## Supporting information

S1 Checklist RECORD Statement. Benchimol EI, Smeeth L, Guttmann A, Harron K, Moher D, Petersen I, Sørensen HT, von Elm E, Langan SM, the RECORD Working Committee. The REporting of studies Conducted using Observational Routinely-collected health Data (RECORD) Statement. PLoS Medicine 2015; in press. *Checklist is protected under Creative Commons Attribution (CC BY) license.
(DOCX)

S1 Appendix Supporting information: additional text and tables. **Table A.** Gestational age categories. **Table B.** Missing data. **Table C.** Maternal deprivation and major congenital anomalies. **Table Di.** Education results and deprivation, unadjusted data. **Table Dii.** Education results and deprivation, adjusted for child sex. **Table Ei.** Associations between isolated major congenital anomalies and SEN provision at statement level (unadjusted analysis). **Table Eii.** Associations between isolated major congenital anomalies and SEN provision at statement level, adjusted for deprivation. **Table Fi.** Data underlying Fig 4. Achieving 5 GCSEs at grades C–A* with EWMS, children with major congenital anomalies, with and without FSM. **Table Fii.** Data underlying Fig 5. Achieving 5 GCSEs at grades C–A* with EWMS, children without major congenital anomalies, with and without FSM. **Table G.** Re-analysis with Down syndrome excluded. **Table H.** Associations with attainment at ages 11 and 16, modelled without SEN provision. **Table I.** Re-analysis of educational outcomes without year of birth. **Table J.** Re-analysis of educational outcomes with year of birth as a continuous variable. **Table K.** Data underlying Fig 6: achieving 5 GCSEs at grades C–A* with EWMS at age 16, maternal deprivation and major congenital anomalies. **Table Li.** Re-analysis including boys only. **Table Lii.** Re-analysis including girls only. **Table M.** Educational outcomes and SEN for birth years 1998–2007. **Table N.** Educational outcomes and deprivation for birth years 1998–2007. **Table O.** Sensitivity analysis, for deprivation data. **Table P.** Individual major congenital anomalies and educational attainment, unadjusted data. **Table Q.** Educational outcomes and major congenital anomalies, adjusted for co-morbidities and SEN, modelled without deprivation. **Table R.** Education attainment stratified by SEN provision. **List.** EUROCAT major congenital anomalies in this study as in EUROCAT Guide 1.4.
(DOCX)

S1 File EUROLINKCAT work package 5. Study protocol and Statistical analysis plan.
(DOCX)

S1 Data Analyses including interaction terms. (XLSX)

S1 Fig Deprivation and educational outcomes. (TIF)

S2 Fig Overview of possible mediators. Note to S2 Fig: SEN special educational needs.
(TIF)

## Abbreviations

ADBE: Annual District Births Extract
ALF: anonymised linkage field
ATS: access to services
CARIS: Congenital Anomaly Registers & Information Service for Wales
CHD: congenital heart defects
CI: confidence intervals
EWMS: English or Welsh/Cymraeg), maths and science
FSM: FSM free school meals
GCSEs: General Certificates of Secondary Education
ICD10: International Classification of Diseases version 10
IGRP: Information Governance Review Panel
KS: key stages
MaCA: Major congenital anomalies
NCCHD: National Child Community Health Database
NHS: National Health Service
OR: odds ratio
PEDW: Patient Episodes Dataset for Wales
SAIL: Secure Anonymised Information Linkage
SEN: special education needs
SES: socioeconomic status
WDS: Welsh Demographic Service dataset
WIMD: Welsh Index of Multiple Deprivation
WLGP: Welsh Longitudinal General Practice.
