# Supplementary material for: Associations of major congenital anomalies, long-term health conditions, and socioeconomic deprivation with educational attainment in Wales: A population-based data linkage study
Source: PLoS Med. 2026 Oct 16;23(10):e1005050. doi: 10.1371/journal.pmed.1005050 (PMC13705610; doi:10.1371/journal.pmed.1005050)
Supplement: Supplementary file 2 [file pmed.1005050.s002.docx]

# Associations of major congenital anomalies, long-term health conditions and socioeconomic deprivation with educational attainment in Wales: a population-based data linkage study

# S1 appendix

# Supporting information: additional text and tables

Contents

[Associations of major congenital anomalies, long-term health conditions and socioeconomic deprivation with educational attainment in Wales: a population-based data linkage study	1](#_Toc242187403)

[S1 appendix	1](#_Toc242187404)

[Supporting information: additional text and tables	1](#_Toc242187405)

[S1 Appendix. Additional details of methods	3](#_Toc242187406)

[Text A. Pre-published protocol and analysis plan	3](#_Toc242187407)

[Text B. Moderators	3](#_Toc242187408)

[Text C. Mediation	4](#_Toc242187409)

[Text D Major congenital anomalies	4](#_Toc242187410)

[Text E Gestational age	4](#_Toc242187411)

[Table A. Gestational age categories	5](#_Toc242187412)

[Table B. Missing data by category	5](#_Toc242187413)

[Text F. Interaction terms	5](#_Toc242187414)

[Text G. Prescription data	5](#_Toc242187415)

[References	6](#_Toc242187416)

[Additional Data Tables	7](#_Toc242187417)

[Table C. Maternal deprivation and major congenital anomalies, with reference to the least deprived quintile	7](#_Toc242187418)

[Table Di. Education results and deprivation	8](#_Toc242187419)

[Table Dii. Education results and deprivation, adjusted for child sex	9](#_Toc242187420)

[Table Ei. Associations between isolated major congenital anomalies and SEN provision at statement level (unadjusted analysis) at KS2 (n=256,568) and KS4 (n=128,727), unadjusted data	10](#_Toc242187421)

[Table Eii. Associations between major congenital anomalies and SEN provision at statement level, adjusted for deprivation at KS2 (n=256,568) and KS4 (n=128,727)	11](#_Toc242187422)

[Table Fi. Data underlying figure 4. Achieving 5 GCSEs at grades C-A* with EWMS at age 16, children with major congenital anomalies, with and without FSM (n=4427), unadjusted data	12](#_Toc242187423)

[Table Fii. Data underlying figure 5. Achieving 5 GCSEs at grades C-A* with EWMS at age 16, children without major congenital anomalies, with and without FSM (n=124300)	12](#_Toc242187424)

[Table G. Associations with attainment at KS2 and KS4, ages 11 and 16, Re-analysis with children with Down syndrome excluded KS2 (n=256,366) and KS4 (n=128,661)	13](#_Toc242187425)

[Table H. Associations with attainment at KS2 and KS4, ages 11 and 16, modelled without SEN provision at KS2 (n=256,568) and KS4 (n=128,727)	15](#_Toc242187426)

[Table I. Re-analysis of educational outcomes and isolated major congenital anomalies without year of birth	17](#_Toc242187427)

[Table J. Re-analysis of educational outcomes with year of birth as a continuous variable	18](#_Toc242187428)

[Table K. Data underlying figure 6: achieving 5 GCSEs at grades C-A* with EWMS at age 16, income deprivation and major congenital anomalies, unadjusted data	20](#_Toc242187429)

[Table L. Analysis of educational attatinment stratified by child’s sex	21](#_Toc242187430)

[Table Li. Re-analysis with boys only	21](#_Toc242187431)

[Table Lii. Re-analysis with girls only	22](#_Toc242187432)

[Table M. Education outcomes and SEN for birth years 1998- 2007, unadjusted data	24](#_Toc242187433)

[Table N. Education outcomes and deprivation (as FSM eligibility) for birth years 1998-2007	25](#_Toc242187434)

[Table O. Sensitivity analysis, based on FSM eligibility	26](#_Toc242187435)

[Table P. Individual major congenital anomalies and educational outcomes, unadjusted data at KS2 (n=256,568) and KS4 (n=128,727) (ages 11 and 16)	27](#_Toc242187436)

[Table Q. Education outcomes and major congenital anomalies, adjusted for co-morbidities and SEN, modelled without deprivation	28](#_Toc242187437)

[Table R. Educational attainment stratified by SEN provision	29](#_Toc242187438)

[Table Ri. Educational attainment stratified by SEN provision (KS2 low level pass)	30](#_Toc242187439)

[Table Rii. education attainment stratified by SEN provision (KS2 high)	31](#_Toc242187440)

[Table Riii. Educational attainment stratified by SEN provision (KS4 – any 5 GCSEs)	32](#_Toc242187441)

[Table Riv, Educational attainment stratified by SEN provision (KS4 5 GCSEs C-A* with EWMS)	33](#_Toc242187442)

[S1 Appendix List. EUROCAT major congenital anomalies in this study as in EUROCAT Guide 1.4	35](#_Toc242187443)

[Abbreviations	37](#_Toc242187444)

## S1 Appendix. Additional details of methods

### Text A. Pre-published protocol and analysis plan

The protocol for this EUROLINKCAT sub-study is available at: [eurolinkcat.eu/wp5-education/wp5-educationstudyprotocol](https://eur03.safelinks.protection.outlook.com/?url=https%3A%2F%2Fwww.eurolinkcat.eu%2Fwp5-education%2Fwp5-educationstudyprotocol&data=05%7C02%7CS.E.Jordan%40Swansea.ac.uk%7C7fc6c2f843934aa3484408ded2d0a963%7Cbbcab52e9fbe43d6a2f39f66c43df268%7C0%7C0%7C639179991114153124%7CUnknown%7CTWFpbGZsb3d8eyJFbXB0eU1hcGkiOnRydWUsIlYiOiIwLjAuMDAwMCIsIlAiOiJXaW4zMiIsIkFOIjoiTWFpbCIsIldUIjoyfQ%3D%3D%7C0%7C%7C%7C&sdata=sMalNhy3DOg4RGEo1j9%2BNtwvkIVMZveUpUmMIcO3Uwc%3D&reserved=0), and an analysis plan at: [eurolinkcat.eu/wp5-education/wp5analysisplans](https://eur03.safelinks.protection.outlook.com/?url=https%3A%2F%2Fwww.eurolinkcat.eu%2Fwp5-education%2Fwp5analysisplans&data=05%7C02%7CS.E.Jordan%40Swansea.ac.uk%7C7fc6c2f843934aa3484408ded2d0a963%7Cbbcab52e9fbe43d6a2f39f66c43df268%7C0%7C0%7C639179991114176329%7CUnknown%7CTWFpbGZsb3d8eyJFbXB0eU1hcGkiOnRydWUsIlYiOiIwLjAuMDAwMCIsIlAiOiJXaW4zMiIsIkFOIjoiTWFpbCIsIldUIjoyfQ%3D%3D%7C0%7C%7C%7C&sdata=R8529xlPZ6TVWPTkOpJXl7KhoTmcPQTth8c2VjdNxxk%3D&reserved=0).

The protocol envisaged combining data from Wales, four English regions, Odense, Finland, Italy and Norway. Some countries were unable to supply data. The English regions did not supply population controls. Variables for socio-economic status differed in Finland. Only Wales was able to include co-morbidities, hospitalisation, gestational age, SEN provision by level, and components of the socio-economic status measure in quintiles. Due to substantial heterogeneity in the available data, no common data model could be built, and no meta-analysis was possible [1]. Decisions to include gestational age and co-morbidities reduced the numbers of records available for analysis. We analysed the CHD category excluding children in the severe CHD category: we did not analyse individual CHDs to avoid low numbers. We did not analyse academic subjects separately to focus on the key educational outcomes needed for entry into professional courses (5 GCSEs grades C-A* with EWMS). Subject specific analyses generated low numbers in some outcomes, particularly for individual major congenital anomalies, and where children passed in some subjects, but not others. School absence data was considered unduly sensitive, and not obtained.

We offer no predictions regarding the number of children likely to need SEN provision in the future. Provision has been in transition, and categories revised [62]. Currently, the additional learning needs provision is encountering increased demands for assessment and support of children with neurodivergence [2].

In response to suggestions of expert reviewers, after submission to PLOS Medicine, we undertook:

- stratified analyses by
  - FSM and major congenital anomalies (Table 9)
  - SEN provision (Tables Fi, Fii, Ri-iv)
  - maternal deprivation (Table K),
  - child sex (Tables Li, Lii),
  - birth years (Tables M, N),
- sensitivity analyses excluding
  - Down syndrome (Table G),
  - SEN (Table H),
  - year of birth (Table I)
  - FSM excluding missing data (Table O),
  - deprivation (Table Q),
- Exploration of mediation (Tables C, Di)
- Exploration of moderation (Table 9) and interaction terms (S1 data file).

These analyses were requested by reviewers, and not specified *a priori.*

### Text B. Moderators

We explored whether the association between major congenital anomalies and poor outcome was stronger amongst children not receiving SEN support than those receiving SEN support. The numbers in Table 9 indicate that differences between those with and without major congenital anomalies increased with higher levels of SEN provision for all outcomes except 5 GCSEs C-A* with EWMS. SEN provision reduced the differences in attainment rates only for 5GCSEs C-A* with EWMS, suggesting that it was only a moderator for the highest level of attainment.

### Text C. Mediation

A variable functions as a mediator when: a) variations in levels of the predictor variable significantly account for variations in the presumed mediator, b) variations in the mediator significantly account for variations in the outcome variable, and c) when these are both controlled, a previously significant relation between the predictor and outcome variables is no longer significant [3, 89].

Adopting this definition, we tested for mediation for:

Deprivation at birth

Deprivation was associated with educational outcomes (Table Di). We found no association between deprivation at birth and major congenital anomalies (Table C). Although deprivation is the most significant predictor of educational outcomes, adding it to the models makes little difference to the association between major congenital anomalies and educational outcomes (S4 file). The interaction between ill-health, deprivation and educational attainment is complex and bidirectional. Deprivation engenders both ill-health and poor educational attainment: the most deprived families have little time to devote to their children and their health. Any illness in the family reduces employment and increases deprivation (Fig S1). In Wales 1998-2007, deprivation was associated with educational attainment, but did not, to any appreciable extent, mediate the association with major congenital anomalies. It is outside the scope of this paper to speculate on the reasons for this, but we note that mothers from less deprived quintiles were older.

Hospitalisation

Children with major congenital anomalies spent more time in hospital than those without (Table 5). However, in all analyses, ‘days in hospital’ did not influence educational attainment when corrected to 2 decimal places.

SEN provision

We checked the associations between major congenital anomalies and SEN (Table 7), and SEN and educational outcomes (Table M). We then modelled the data with and without SEN (Tables 10, H). Additions of SEN reduced the association between major congenital anomalies and educational outcomes. SEN provision is allocated on the basis of perceived reduced ability to learn, which is often associated with illness (S2 fig). It is likely that SEN provision was a mediator in our analysis of educational outcomes, and may reduce the association between major congenital anomalies and educational attainment [4].

### Text D Major congenital anomalies

For some isolated major congenital anomalies, the sample was sufficiently large for some inferential analysis (Table 4). Where inferential analyses divided the sample so that at least one outcome category contained <5 individuals, results were redacted or the major congenital anomaly was not analysed separately. Major congenital anomalies that could not be analysed separately were combined into an ‘other major congenital anomalies’ category. The major congenital anomalies in the EUROCAT classification where there were too few individuals for separate analysis are appended to this supplement (List 1).

### Text E Gestational age

We used NCCHD-recorded birth weight data to ensure realistic categorisation of gestational age data (Table A) and set all other records to missing. Values above 44 weeks were considered implausible [5,6]. Before classifying these infants as ‘term births’, gestations were checked against birth weights. Given that all children were live births, surviving to age 16, we considered a birth weight below the 0.4th centile [7] was unlikely to be associated with term birth, and the gestational age was set to missing. Pre-term birth categories were also checked against birth weight (Table A).

### Table A. Gestational age categories

|  | Gestational age | Birth weights not accepted |
| --- | --- | --- |
| Very pre-term | < 32 weeks | Missing  **or**  < 0.4^th^ centile for 23 weeks  > 99.6^th^ centile for 32 weeks |
| Pre-term | 32 – 36 weeks | Missing  **or**  < 0.4^th^ centile for 32 weeks  > 99.6^th^ centile for 37 weeks |
| Term | ≥ 37 weeks  ≥ 44 weeks | Missing  **or**  > 99.6^th^ centile for 37 weeks |

Missing data are summarised in Table B.

### Table B. Missing data by category

|  |  |  | For children with education data | | | |  |
| --- | --- | --- | --- | --- | --- | --- | --- |
|  | Total in birth years | Education data n (%) | No  gestation age n (%) | No SES  n (%) | No primary care /prescription data n (%) | Total missing n (%) | Included |
| KS2, age 11 | 314597 | 271,050 (86.15) | 3658 (1.35) | 7865 (2.90) | 2959 (1.09) | 14482 (5.34) | 256568 |
| KS4, age 16 | 151913 | 131,605 (86.63) | 1649 (1.25) | 460 (0.35) | 769 (0.58) | 2878 (2.19) | 128727 |

*Note to Table: KS key stage, SES socio-economic status*

### Text F. Interaction terms

We investigated the associations between child’s sex, deprivation, SEN provision, hospitalisation and co-morbidities by testing pairwise interaction effects between a) major congenital anomalies and SEN, and b) major congenital anomalies and SES, in the final model, Table 10. Statistically significant interaction terms included: SEN and sex, SEN and FSM eligibility, (except for 5 GCSEs C-A* with EWMS); and SEN and income, mainly at KS2. Adjusting for interaction terms made little difference to key associations, but widened confidence intervals: for example, attaining 5 GCSEs C-A* with EWMS remained statistically non-significantly associated with major congenital anomalies, and FSM remained the strongest association, other than SEN provision (data available in S4 File).

### Text G. Prescription data

Only 86% primary care practices in Wales contribute data to SAIL. Data submission is voluntary and not remunerated. As reported (Divin et al 2022) [85] in this cohort, the population with prescription data had a similar mean deprivation (Townsend) score (Townsend 1987) [8] to the population without prescription data (mean 0.603 [SD 3.678] vs 0.612 [3.580]), which was statistically insignificant, P 0.38, t 0.87, df-t 281,962.8, with a very small effect size (r=0.002).

### References

*Reference numbers are those of the manuscript, where possible. If references are not cited in the manuscript, they are numbered according to their appearance in this text.*

1. Rankin J. et al 2026 Deliverable 5.1: Report on education achievements and needs of children born with a congenital anomaly in selected European countries with predictions of the number of children with special education needs. EUROLINKCAT website [loadFile.aspx](https://www.eurolinkcat.eu/loadFile.aspx?filename=EUROlinkCAT%20WP5%20Deliverable%20D5.1_final4_submitted(2).pdf)

2. Welsh Government (2025) Estimates of the number of neurodivergent people in Wales using multiple data sources, including estimates by type of neurodivergence, age and region. <https://www.gov.wales/estimated-prevalence-neurodivergent-people-wales-science-research-evidence-html>

3. Jordan S, Bromley R, Damase-Michel C, Given J, Komninou S, Loane M, Marfell N, Dolk H. Breastfeeding, pregnancy, medicines, neurodevelopment, and population databases: the information desert. Int Breastfeed J. 2022 Aug 2;17(1):55. doi: 10.1186/s13006-022-00494-5. PMID: 35915474.

4. Morrow EL, Duff MC, Mayberry LS. Mediators, Moderators, and Covariates: Matching Analysis Approach for Improved Precision in Cognitive-Communication Rehabilitation Research. J Speech Lang Hear Res. 2022 Nov 17;65(11):4159-4171. doi: 10.1044/2022_JSLHR-21-00551. Epub 2022 Oct 28. PMID: 36306506; PMCID: PMC9940892.

5. Cole TJ, Williams AF, Wright CM; RCPCH Growth Chart Expert Group. Revised birth centiles for weight, length and head circumference in the UK-WHO growth charts. Ann Hum Biol. 2011 Jan;38(1):7-11. doi: 10.3109/03014460.2011.544139. Erratum in: Ann Hum Biol. 2011 Mar;38(2):241. Growth Chart Expert Group [added]. PMID: 21175302.

6. Hilder L, Costeloe K, Thilaganathan B. Prolonged pregnancy: evaluating gestation-specific risks of fetal and infant mortality. Br J Obstet Gynaecol. 1998 Feb;105(2):169-73. doi: 10.1111/j.1471-0528.1998.tb10047.x. PMID: 9501781.

7. Royal College of Paediatrics and Child Health (RCPCH)/ Department of Health 2009: BOYS UK–WHO Neonatal and Infant Close Monitoring Growth Chart 23 weeks gestation to 2 years corrected age Available: <https://www.rcpch.ac.uk/sites/default/files/Boys_neonatal_and_infant_close_monitoring_growth_chart.pdf> GIRLS UK–WHO Neonatal and Infant Close Monitoring Growth Chart 23 weeks gestation to 2 years corrected age Available: <https://www.rcpch.ac.uk/sites/default/files/Girls_neonatal_and_infant_close_monitoring_growth_chart.pdf>

8. Townsend P. Deprivation. *Journal of Social Policy*. 1987;16(2):125-146. doi:10.1017/S0047279400020341

62. Welsh Parliament (2024). Implementation of education reforms: Interim Report. Children, Young People and Education Committee. Welsh Parliament, 2024.

85. Divin N, Given JE, Tan J, Astolfi G, Ballardini E, Barrachina-Bonet L, Cavero-Carbonell C, Coi A, Garne E, Gissler M, Heino A, Jordan S, Pierini A, Scanlon I, Urhøj SK, Morris JK, Loane M. Antiasthmatic prescriptions in children with and without congenital anomalies: a population-based study. BMJ Open. 2023 Oct 13;13(10):e068885. doi: 10.1136/bmjopen-2022-068885. PMID: 37832979.

89. Baron RM, Kenny DA: The moderator–mediator variable distinction in social psychological research: Conceptual, strategic, and statistical considerations. Journal of Personality and Social Psychology 1986, 51(6):1173–1182. 10.1037/0022-3514.51.6.1173

### Additional Data Tables

### Table C. Maternal deprivation and major congenital anomalies, with reference to the least deprived quintile

|  |  | Fourth most deprived quintile | Central quintile for maternal deprivation | Second most deprived quintile | Most deprived quintile |
| --- | --- | --- | --- | --- | --- |
|  |  | OR (95% CI) | OR (95% CI) | OR (95% CI) | OR (95% CI) |
| Major congenital anomaly | All anomalies (al1) | 0.87 (0.81,0.94) | 0.95 (0.89,1.02) | 0.96 (0.90,1.03) | 1.03 (0.97,1.10) |
|  | Cleft lip | 1.80 (0.88,3.69) | 2.22 (1.12,4.39) | 3.15 (1.65,6.02) | 2.81 (1.48,5.35) |
|  | Cleft palate | 1.30 (0.72,2.34) | 1.36 (0.77,2.40) | 1.62 (0.94,2.80) | 1.44 (0.84,2.46) |
|  | CHD (excluding severe CHD) | 0.87 (0.74,1.03) | 0.96 (0.82,1.13) | 0.96 (0.82,1.12) | 1.08 (0.93,1.25) |
|  | Gastroschisis | 1.40 (0.57,3.41) | 2.16 (0.96,4.86) | 4.14 (1.96,8.75) | 2.81 (1.31,6.02) |
|  | Multicystic renal dysplasia | 2.79 (1.00,7.74) | 2.28 (0.81,6.40) | 2.69 (0.99,7.33) | 4.81 (1.88,12.31) |
|  | Hypospadias | 1.05 (0.82,1.34) | 0.87 (0.68,1.12) | 0.85 (0.67,1.09) | 1.16 (0.92,1.45) |
|  | Limb reduction defects | 0.59 (0.24,1.49) | 0.89 (0.40,1.98) | 0.87 (0.39,1.91) | 1.01 (0.49,2.11) |
|  | Hydrocephalus | 1.67 (0.49,5.71) | 0.07 (0.00,2.62) | 4.03 (1.38,11.74) | 2.45 (0.82,7.38) |
|  | Craniosynostosis | 0.26 (0.11,0.66) | 0.74 (0.40,1.38) | 0.31 (0.14,0.69) | 0.95 (0.55,1.65) |
|  | Down syndrome | 0.61 (0.39,0.96) | 0.91 (0.62,1.35) | 0.58 (0.37,0.89) | 0.54 (0.36,0.82) |
|  | Turner syndrome | 1.77 (0.36,8.83) | 0.87 (0.14,5.37) | 0.45 (0.05,3.87) | 0.00 (0.00,0.00) |
|  | Severe CHD | 1.13 (0.79,1.61) | 1.32 (0.94,1.85) | 1.27 (0.91,1.78) | 1.37 (0.99,1.88) |
|  | Other major congenital anomalies | 0.89 (0.81,0.98) | 0.97 (0.88,1.06) | 0.97 (0.88,1.06) | 1.01 (0.92,1.10) |
| Notes to Table: CHD major congenital heart defect, CI confidence interval, OR odds ratio.  The least deprived quintile was set as 1.  There were too few children with oesophageal atresia, ano-rectal atresia and stenosis, diaphragmatic hernia, and *spina bifida* to report unadjusted odds ratios. | | | | | |

### Table Di. Education results and deprivation

| Key stage (KS) | Maternal deprivation quintile | Pass | % | Unadjusted OR (95% CI) | High pass | % | Unadjusted OR (95% CI) | TOTAL |
| --- | --- | --- | --- | --- | --- | --- | --- | --- |
| KS2 | 1 (Least deprived) | 41,859 | 96.80 | 1.00 | 40,058 | 92.63 | 1.00 | 43,243 |
| KS2 | 2 | 42,307 | 95.63 | 0.72 (0.67,0.78) | 39,879 | 90.14 | 0.73 (0.69,0.76) | 44,242 |
| KS2 | 3 | 46,980 | 93.98 | 0.52 (0.48,0.55) | 43,648 | 87.32 | 0.55 (0.52,0.57) | 49,987 |
| KS2 | 4 | 49,825 | 92.44 | 0.40 (0.38,0.43) | 45,863 | 85.09 | 0.45 (0.43,0.47) | 53,898 |
| KS2 | 5 (Most deprived) | 58,546 | 89.80 | 0.29 (0.27,0.31) | 52,564 | 80.62 | 0.33 (0.32,0.34) | 65,198 |
|  | All quintiles | 239,517 | 93.35 |  | 222,012 | 86.53 |  | 256,568 |
| KS4 | 1 (Least deprived) | 21,234 | 97.52 | 1.00 | 16,033 | 73.63 | 1.00 | 21,774 |
| KS4 | 2 | 21,409 | 96.49 | 0.70 (0.63,0.78) | 14,506 | 65.38 | 0.68 (0.65,0.70) | 22,187 |
| KS4 | 3 | 23,983 | 95.20 | 0.50 (0.46,0.56) | 14,407 | 57.19 | 0.48 (0.46,0.50) | 25,192 |
| KS4 | 4 | 25,320 | 93.74 | 0.38 (0.34,0.42) | 13,817 | 51.15 | 0.37 (0.36,0.39) | 27,012 |
| KS4 | 5 (Most deprived) | 29,384 | 90.24 | 0.24 (0.21,0.26) | 13,029 | 40.01 | 0.24 (0.23,0.25) | 32,562 |
|  | All quintiles | 121,330 | 94.25 |  | 71,792 | 55.77 |  | 128,727 |

Based on maternal deprivation at birth. OR odds ratio, CI confidence interval.

| Table Dii. Education results and deprivation, adjusted for child sex | | | | |
| --- | --- | --- | --- | --- |
|  |  | Any pass | High pass |  |
| Key stage (KS) | Maternal deprivation quintile | aOR (95% CI) | aOR (95% CI) | Total *n* |
| KS2 | 1 (least deprived) | 1 | 1 | 43,243 |
|  | 2 | 0.72 (0.68,0.78) | 0.73 (0.69,0.76) | 44,242 |
|  | 3 | 0.52 (0.48,0.55) | 0.55 (0.52,0.57) | 49,987 |
|  | 4 | 0.40 (0.38,0.43) | 0.45 (0.43,0.47) | 53,898 |
|  | 5 (most deprived) | 0.29 (0.27,0.31) | 0.33 (0.32,0.34) | 65,198 |
|  | Sex (male) | 0.59 (0.57,0.60) | 0.63 (0.61,0.64) | 25,6568 |
| Model parameters* | -2LL (log likelihood) | 121620 | 197212 |  |
|  | Nagelkerke R^2^ | 0.03 | 0.03 |  |
|  | Goodness of fit† c^2^ | 11.17 | 4.41 |  |
|  |  | Any 5 GCSEs | 5 GCSEs C-A* with EWMS | |
| Key stage | Maternal deprivation quintile | aOR (95% CI) | aOR (95% CI) | Total *n* |
| KS4 | 1 (least deprived) | 1 | 1 | 21,774 |
|  | 2 | 0.70 (0.63,0.79) | 0.68 (0.65,0.71) | 22,187 |
|  | 3 | 0.51 (0.46,0.56) | 0.48 (0.46,0.50) | 25,192 |
|  | 4 | 0.38 (0.34,0.42) | 0.37 (0.36,0.39) | 27,012 |
|  | 5 (most deprived) | 0.23 (0.21,0.26) | 0.24 (0.23,0.24) | 32,562 |
|  | Sex (male) | 0.59 (0.56,0.62) | 0.69 (0.68,0.71) | 128,727 |
| Model parameters | -2LL (log likelihood) | 54518 | 168400 |  |
|  | Nagelkerke R^2^ | 0.04 | 0.05 |  |
|  | Goodness of fit† c^2^ | 1.4 | 3.24 |  |

Notes to Table: aOR adjusted odds ratio, CI confidence interval, EWMS English/Welsh, Maths and Science, GCSE General Certificate of Secondary Education, OR odds ratio.

### Table Ei. Associations between isolated major congenital anomalies and SEN provision at statement level (unadjusted analysis) at KS2 (n=256,568) and KS4 (n=128,727), unadjusted data

|  | KS2 age 11 | KS4 age 16 |
| --- | --- | --- |
|  | OR (95% CI) | OR (95% CI) |
| Cleft lip | 2.64 (1.28,5.45) | 2.81 (1.11,7.08) |
| Cleft palate | 2.90 (1.46,5.75) | 2.53 (1.01,6.34) |
| CHD (excluding severe CHD) | 2.75 (2.23,3.38) | 2.69 (2.05,3.53) |
| Oesophageal atresia | 2.73 (0.35,21.10) |  |
| Ano-rectal atresia and stenosis | 4.00 (0.91,17.50) | 2.11 (0.27,16.20) |
| Diaphragmatic hernia | 3.53 (0.82,15.30) | 2.11 (0.27,16.20) |
| Gastroschisis | 3.86 (1.92,7.72) | 2.53 (0.77,8.28) |
| Multicystic renal dysplasia | 2.53 (1.10,5.83) | 2.17 (0.67,7.05) |
| Hypospadias | 3.31 (2.45,4.46) | 2.97 (1.98,4.47) |
| Spina bifida | 16.40 (6.06,44.40) | 9.50 (2.52,35.80) |
| Limb reduction defects | 6.46 (3.01,13.90) | 8.42 (3.34,21.20) |
| Hydrocephalus | 30.03 (15.33,58.80) | 22.48 (8.67,58.30) |
| Craniosynostosis | 6.19 (3.40,11.20) | 2.44 (0.75,8.00) |
| Down syndrome | >1 (>1,>1) | >1 (>1,>1) |
| Turner syndrome | 25.38 (11.36,56.70) | 10.84 (2.80,41.90) |
| Severe CHD | 6.09 (4.38,8.46) | 3.13 (1.83,5.36) |
| Other major congenital anomalies | 8.58 (7.89,9.33) | 5.42 (4.80,6.12) |

Notes to table: CHD congenital heart defect, KS key stage, OR odds ratio, CI confidence interval.

Almost all children with Down syndrome received a statement. Numbers for some major congenital anomalies are low and confidence intervals are wide. All major congenital anomalies considered were associated with statementing at KS2. There was no statistically significant association with oesophageal atresia, diaphragmatic hernia, gastroschisis, multicystic renal dysplasia, ano-rectal atresia and stenosis, and craniosynostosis at KS4.

There were too few children with oesophageal atresia at KS4 to report results.

### Table Eii. Associations between major congenital anomalies and SEN provision at statement level, adjusted for deprivation at KS2 (n=256,568) and KS4 (n=128,727)

|  |  | KS2 age 11 | KS4 age 16 |
| --- | --- | --- | --- |
|  |  | aOR (95% CI) | aOR (95% CI) |
|  | Any major congenital anomaly | 7.65 (7.15,8.19) | 5.06 (4.58,5.60) |
|  | FSM eligibility | 3.17 (3.00,3.34) | 3.97 (3.70,4.26) |
| Access to services (ATS) | ATS1 most deprived quintile | 1.18 (1.09,1.28) | 1.26 (1.13,1.40) |
|  | ATS2 | 0.98 (0.91,1.05) | 1.10 (0.99,1.21) |
|  | ATS3 | 0.97 (0.90,1.04) | 1.11 (1.00,1.23) |
|  | ATS4 | 0.97 (0.90,1.04) | 1.08 (0.97,1.19) |
|  | ATS5 least deprived quintile | 1.00 | 1.00 |
| Physical environment (ENV) | ENV1 most deprived quintile | 1.27 (1.18,1.38) | 1.28 (1.16,1.41) |
|  | ENV2 | 1.10 (1.02,1.20) | 1.13 (1.02,1.25) |
|  | ENV3 | 1.08 (1.00,1.17) | 1.12 (1.01,1.24) |
|  | ENV4 | 1.16 (1.07,1.25) | 1.14 (1.03,1.27) |
|  | ENV5 least deprived quintile | 1.00 | 1.00 |
| Child’s household income (INC) | INC1 most deprived quintile | 1.12 (1.02,1.23) | 1.06 (0.94,1.20) |
|  | INC2 | 1.04 (0.95,1.15) | 0.97 (0.86,1.09) |
|  | INC3 | 1.06 (0.96,1.16) | 1.05 (0.94,1.18) |
|  | INC4 | 1.02 (0.93,1.12) | 0.95 (0.84,1.07) |
|  | INC5 least deprived quintile | 1.00 | 1.00 |
| Maternal deprivation at birth | 1 least deprived quintile | 1.00 | 1.00 |
|  | MATDPR_IND2 | 1.12 (1.02,1.24) | 1.08 (0.95,1.23) |
|  | MATDPR_IND3 | 1.22 (1.10,1.34) | 1.26 (1.11,1.43) |
|  | MATDPR_IND4 | 1.38 (1.26,1.52) | 1.44 (1.27,1.63) |
|  | MATDPR_IND5 most deprived quintile | 1.58 (1.44,1.74) | 1.66 (1.47,1.87) |

*Notes to table:* *ATS access to services, ENV physical environment, KS key stage, INC household income levels, SEN special educational needs, aOR adjusted odds ratio, CI confidence interval, MATDPR_IND maternal deprivation indicator.*

*Adjusted for components of WIMD: ATS, ENV, INC, MATDR. Reference categories for the components of the WIMD 2014 were the least deprived quintile (corresponding with highest rank) [79]. For maternal deprivation, 1 is the least deprived quintile. For other indices, 5 is the least deprived quintile.*

Deprivation measures were as of year of assessment, with the exception of maternal deprivation, which as measured at birth.

Statements were more closely associated with major congenital anomalies than any measure of deprivation, including FSM. Income was the only measure of deprivation not associated with statementing. For other measures, a trend was observable.

### Table Fi. Data underlying figure 4. Achieving 5 GCSEs at grades C-A* with EWMS at age 16, children with major congenital anomalies, with and without FSM (n=4427), unadjusted data

|  |  | With FSM | | | No FSM | | |
| --- | --- | --- | --- | --- | --- | --- | --- |
|  |  | N | % | Unadjusted OR (95% CI) | N | % | Unadjusted OR (95% CI) |
| No SEN | Passed | 115 | 34.64 | 0.26 (0.20,0.33) | 1628 | 67.47 | 1.00 |
|  | Did not pass | 217 | 65.36 |  | 785 | 32.53 |  |
|  | Total | 332 |  |  | 2413 |  |  |
| SEN action plan | Passed | 20 | 12.50 | 0.32 (0.19,0.52) | 151 | 31.13 | 1.00 |
|  | Did not pass | 140 | 87.50 |  | 334 | 68.87 |  |
|  | Total | 160 |  |  | 485 |  |  |
| SEN action plan plus | Passed | 19 | 12.18 | 0.35 (0.21,0.60) | 95 | 28.36 | 1.00 |
|  | Did not pass | 137 | 87.82 |  | 240 | 71.64 |  |
|  | Total | 156 |  |  | 335 |  |  |
| Statemented | Passed | 5 | 2.60 | 0.23 (0.09,0.59) | 37 | 10.45 | 1.00 |
|  | Did not pass | 187 | 97.40 |  | 317 | 89.55 |  |
|  | Total | 192 |  |  | 354 |  |  |

Notes to table, EWMS English/ Welsh Maths and Science, FSM free school meals eligibility, GCSE General Certificate of Secondary Education, SEN special educational needs, OR odds ratio, CI confidence interval.

### Table Fii. Data underlying figure 5. Achieving 5 GCSEs at grades C-A* with EWMS at age 16, children without major congenital anomalies, with and without FSM (n=124300)

|  |  | With FSM | | | No FSM | | |
| --- | --- | --- | --- | --- | --- | --- | --- |
|  |  | N | % | Unadjusted OR (95% CI) | N | % | Unadjusted OR (95% CI) |
| No SEN | Passed | 4300 | 40.15 | 0.28 (0.27,0.29) | 59851 | 70.53 | 1.00 |
|  | Did not pass | 6410 | 59.85 |  | 25003 | 29.47 |  |
|  | Total | 10710 |  |  | 84854 |  |  |
| SEN action plan | Passed | 371 | 9.97 | 0.29 (0.26,0.33) | 3283 | 27.45 | 1.00 |
|  | Did not pass | 3349 | 90.03 |  | 8678 | 72.55 |  |
|  | Total | 3720 |  |  | 11961 |  |  |
| SEN action plan plus | Passed | 257 | 8.30 | 0.35 (0.30,0.40) | 1272 | 20.58 | 1.00 |
|  | Did not pass | 2838 | 91.70 |  | 4908 | 79.42 |  |
|  | Total | 3095 |  |  | 6180 |  |  |
| Statemented | Passed | 68 | 4.91 | 0.33 (0.26,0.44) | 320 | 13.37 | 1.00 |
|  | Did not pass | 1318 | 95.09 |  | 2074 | 86.63 |  |
|  | Total | 1386 |  |  | 2394 |  |  |
| Total |  | 18911 |  |  | 105389 |  |  |

Notes to table, EWMS English/ Welsh Maths and Science, FSM free school meals eligibility, GCSE General Certificate of Secondary Education, SEN special educational needs, OR odds ratio, CI confidence interval.

### Table G. Associations with attainment at KS2 and KS4, ages 11 and 16, Re-analysis with children with Down syndrome excluded KS2 (n=256,366) and KS4 (n=128,661)

|  |  | **KS2** | **KS2** | **KS4** | **KS4** |
| --- | --- | --- | --- | --- | --- |
|  |  | **Lower level pass** | **High Level (4) pass** | **Any 5 GCSEs** | **5 GCSEs C-A* with EWMS** |
|  |  | aOR (95% CI) | aOR (95% CI) | aOR (95% CI) | aOR (95% CI) |
| Birth year  (Reference  1998) | Any major congenital anomaly | 0.79 (0.72,0.85) | 0.87 (0.81,0.93) | 0.86 (0.77,0.97) | 0.94 (0.88,1.01) |
|  | 1999 | 0.87 (0.81,0.94) | 0.94 (0.88,0.99) | 1.09 (1.00,1.19) | 1.16 (1.12,1.21) |
|  | 2000 | 1.03 (0.95,1.11) | 1.21 (1.14,1.28) | 1.10 (1.01,1.20) | 1.18 (1.13,1.23) |
|  | 2001 | 1.28 (1.18,1.39) | 1.56 (1.47,1.66) | 0.91 (0.83,0.99) | 0.98 (0.94,1.02) |
|  | 2002 | 1.39 (1.28,1.50) | 1.95 (1.83,2.07) | 0.84 (0.78,0.92) | 0.93 (0.89,0.97) |
|  | 2003 | 1.58 (1.45,1.71) | 2.42 (2.27,2.57) |  |  |
|  | 2004 | 1.77 (1.62,1.92) | 2.77 (2.60,2.95) |  |  |
|  | 2005 | 1.83 (1.68,1.99) | 3.11 (2.92,3.32) |  |  |
|  | 2006 | 1.89 (1.74,2.06) | 3.36 (3.15,3.59) |  |  |
|  | 2007 | 1.63 (1.49,1.79) | 2.86 (2.66,3.07) |  |  |
| Sex | Male | 1.14 (1.10,1.19) | 1.10 (1.07,1.14) | 0.86 (0.82,0.91) | 0.81 (0.79,0.83) |
| Gestational age | Pre-term 32-36+6 weeks | 0.99 (0.84,1.16) | 0.83 (0.73,0.95) | 1.51 (1.18,1.96) | 0.94 (0.81,1.08) |
|  | Very pre-term <32 weeks | 0.88 (0.83,0.95) | 0.91 (0.86,0.97) | 1.07 (0.96,1.19) | 1.02 (0.97,1.08) |
| FSM | FSM1 eligibility | 0.53 (0.51,0.55) | 0.54 (0.52,0.55) | 0.42 (0.40,0.45) | 0.39 (0.38,0.41) |
| Access to services | ATS1 – most deprived quintile | 1.04 (0.97,1.11) | 0.98 (0.94,1.03) | 0.92 (0.83,1.01) | 0.89 (0.85,0.93) |
|  | ATS2 | 0.91 (0.86,0.97) | 0.94 (0.90,0.98) | 0.90 (0.82,0.98) | 0.86 (0.82,0.90) |
|  | ATS3 | 0.89 (0.84,0.95) | 0.93 (0.89,0.98) | 0.85 (0.78,0.92) | 0.87 (0.84,0.91) |
|  | ATS4 | 0.99 (0.93,1.05) | 0.97 (0.93,1.02) | 0.88 (0.81,0.96) | 0.89 (0.86,0.93) |
|  | Access to services – least deprived quintile | 1.00 | 1.00 | 1.00 | 1.00 |
| Physical  Environment | ENV1 | 1.12 (1.05,1.19) | 1.02 (0.97,1.06) | 0.92 (0.85,1.00) | 1.03 (0.98,1.07) |
|  | ENV2 | 1.13 (1.06,1.20) | 1.06 (1.02,1.11) | 0.94 (0.86,1.02) | 0.97 (0.94,1.01) |
|  | ENV3 | 1.10 (1.03,1.16) | 1.03 (0.99,1.08) | 0.94 (0.87,1.03) | 0.98 (0.94,1.02) |
|  | ENV4 | 1.11 (1.05,1.18) | 1.07 (1.02,1.12) | 1.02 (0.93,1.11) | 1.01 (0.97,1.05) |
|  | Environment 5 – least deprived quintile | 1.00 | 1.00 | 1.00 | 1.00 |
| Child’s household’s  income at  Assessment | INC1 | 0.64 (0.59,0.70) | 0.67 (0.63,0.71) | 0.53 (0.47,0.60) | 0.40 (0.38,0.42) |
|  | INC2 | 0.70 (0.64,0.76) | 0.74 (0.70,0.79) | 0.61 (0.54,0.68) | 0.48 (0.46,0.50) |
|  | INC3 | 0.79 (0.73,0.86) | 0.80 (0.76,0.85) | 0.74 (0.66,0.83) | 0.59 (0.56,0.62) |
|  | INC4 | 0.93 (0.85,1.01) | 0.90 (0.85,0.96) | 0.86 (0.76,0.97) | 0.74 (0.70,0.77) |
|  | 5 Household income – least deprived quintile | 1.00 | 1.00 | 1.00 | 1.00 |
| Maternal  Deprivation  At birth | 1 – least deprived quintile | 1.00 | 1.00 | 1.00 | 1.00 |
|  | 2 – 4^th^ most deprived quintile | 0.99 (0.90,1.07) | 0.98 (0.92,1.04) | 0.89 (0.79,1.01) | 0.85 (0.81,0.89) |
|  | 3 – Central quintile for maternal deprivation | 0.85 (0.78,0.92) | 0.85 (0.80,0.90) | 0.81 (0.72,0.92) | 0.72 (0.69,0.76) |
|  | 4 – 2^nd^ most deprived quintile | 0.77 (0.71,0.84) | 0.81 (0.76,0.86) | 0.75 (0.67,0.84) | 0.65 (0.62,0.68) |
|  | 5 – most deprived quintile | 0.73 (0.67,0.79) | 0.75 (0.70,0.79) | 0.62 (0.55,0.70) | 0.52 (0.50,0.55) |
| Co-morbidities | Asthma >1 prescription | 1.01 (0.96,1.06) | 1.00 (0.96,1.04) | 1.04 (0.96,1.13) | 0.99 (0.95,1.03) |
|  | Epilepsy >1 prescription | 0.47 (0.39,0.55) | 0.55 (0.46,0.65) | 0.76 (0.57,1.04) | 0.60 (0.45,0.80) |
|  | Insulin >1 prescription | 2.04 (1.31,3.32) | 2.38 (1.68,3.45) | 1.27 (0.65,2.79) | 1.12 (0.77,1.63) |
| SEN | A action plan | 0.05 (0.04,0.05) | 0.06 (0.06,0.07) | 0.29 (0.27,0.31) | 0.17 (0.16,0.17) |
|  | P action plan plus | 0.02 (0.02,0.02) | 0.03 (0.03,0.03) | 0.08 (0.08,0.09) | 0.13 (0.12,0.14) |
|  | S statement | 0.00 (0.00,0.00) | 0.01 (0.01,0.01) | 0.03 (0.03,0.03) | 0.07 (0.06,0.08) |
| Hospitalisation | Each day hospitalised | 1.00 (1.00,1.00) | 1.00 (1.00,1.00) | 1.00 (1.00,1.00) | 1.00 (1.00,1.00) |
| Model  Parameters | -2LL (log likelihood) | 74806 | 125788 | 41752 | 143520 |
|  | Nagelkerke R^2^ | 0.4 | 0.38 | 0.26 | 0.19 |
|  | Goodness of fit χ^2^ | 101.56 | 306.08 | 58.49 | 14.82 |

**Notes to table** aOR *adjusted odds ratio, ATS access to services, ENV physical environment, EWMS English/ Welsh plus maths + science, FSM free school meals eligibility, INC household income levels, KS key stage, SEN special educational needs*, CI 95% confidence intervals, *WIMD Welsh Index of Multiple Deprivation (Jones 1017)*

*Odds ratios were adjusted for: Child trait (sex, gestational age, and year of birth), deprivation, medical interventions, co-morbidities, and SEN provision.*

*Reference categories for the components of the WIMD were the highest ranked or least deprived quintiles. For maternal deprivation, 1 is the highest rank. For other indices, 5 is the highest rank.*

*There were no data at age 16 for children born 2003-2007.*

JONES, N. 2017. Welsh Index of Multiple Deprivation 2014: A guide to analysing indicator data. Statistics Wales.

### Table H. Associations with attainment at KS2 and KS4, ages 11 and 16, modelled without SEN provision at KS2 (n=256,568) and KS4 (n=128,727)

|  |  | KS2 Lower level pass | KS2 Higher level pass | KS4 any5GCSEs | KS4 5GCSEs C-A* (EWMS) |
| --- | --- | --- | --- | --- | --- |
|  |  | aOR (95% CI) | aOR (95% CI) | aOR (95% CI) | aOR (95% CI) |
|  | Any major congenital anomaly | 0.36 (0.34,0.38) | 0.48 (0.45,0.51) | 0.48 (0.44,0.53) | 0.73 (0.69,0.78) |
|  | Year of birth 1999 | 0.88 (0.83,0.94) | 0.93 (0.89,0.97) | 1.05 (0.97,1.13) | 1.13 (1.09,1.17) |
|  | Year of birth 2000 | 1.02 (0.95,1.09) | 1.11 (1.06,1.16) | 1.08 (1.00,1.17) | 1.14 (1.10,1.19) |
|  | Year of birth 2001 | 1.15 (1.07,1.23) | 1.27 (1.21,1.33) | 0.89 (0.82,0.96) | 0.96 (0.93,1.00) |
|  | Year of birth 2002 | 1.22 (1.14,1.30) | 1.47 (1.40,1.55) | 0.84 (0.78,0.91) | 0.92 (0.88,0.95) |
|  | Year of birth 2003 | 1.36 (1.27,1.46) | 1.74 (1.66,1.84) |  |  |
|  | Year of birth 2004 | 1.53 (1.43,1.64) | 1.97 (1.87,2.08) |  |  |
|  | Year of birth 2005 | 1.60 (1.49,1.72) | 2.18 (2.07,2.30) |  |  |
|  | Year of birth 2006 | 1.64 (1.52,1.76) | 2.32 (2.20,2.44) |  |  |
|  | Year of birth 2007 | 1.46 (1.35,1.58) | 2.04 (1.92,2.16) |  |  |
|  | SEX: Male | 0.58 (0.56,0.60) | 0.62 (0.61,0.63) | 0.59 (0.56,0.62) | 0.68 (0.67,0.70) |
|  | Gestation 32-36+6 weeks | 0.63 (0.55,0.72) | 0.61 (0.55,0.68) | 0.95 (0.76,1.20) | 0.79 (0.69,0.90) |
|  | Gestation <32 weeks | 0.78 (0.74,0.83) | 0.82 (0.79,0.86) | 0.97 (0.89,1.07) | 0.97 (0.93,1.02) |
|  | FSM eligibility | 0.34 (0.32,0.35) | 0.37 (0.36,0.38) | 0.27 (0.26,0.29) | 0.31 (0.30,0.32) |
| Access to services (ATS) | ATS1 most deprived quintile | 0.86 (0.82,0.92) | 0.82 (0.79,0.86) | 0.84 (0.77,0.92) | 0.84 (0.81,0.87) |
|  | ATS2 | 0.87 (0.82,0.92) | 0.88 (0.85,0.91) | 0.86 (0.80,0.93) | 0.85 (0.81,0.88) |
|  | ATS3 | 0.90 (0.85,0.95) | 0.91 (0.88,0.95) | 0.83 (0.76,0.89) | 0.86 (0.83,0.89) |
|  | ATS4 | 0.97 (0.92,1.02) | 0.96 (0.92,0.99) | 0.86 (0.79,0.93) | 0.88 (0.85,0.92) |
|  | ATS5 least deprived quintile | 1 | 1 | 1 | 1 |
| Physical environment (ENV) | ENV1 most deprived quintile | 1.07 (1.02,1.12) | 1.03 (0.99,1.07) | 0.88 (0.81,0.95) | 0.99 (0.95,1.02) |
|  | ENV2 | 1.10 (1.05,1.16) | 1.07 (1.03,1.11) | 0.92 (0.85,1.00) | 0.97 (0.93,1.00) |
|  | ENV3 | 1.09 (1.03,1.15) | 1.05 (1.01,1.09) | 0.94 (0.87,1.02) | 0.98 (0.94,1.01) |
|  | ENV4 | 1.05 (1.00,1.11) | 1.04 (1.00,1.08) | 0.98 (0.90,1.06) | 1.00 (0.96,1.04) |
|  | ENV5 least deprived quintile | 1 | 1 | 1 | 1 |
| Child’s household income (INC) | INC1 most deprived quintile | 0.58 (0.54,0.62) | 0.58 (0.55,0.61) | 0.49 (0.44,0.55) | 0.39 (0.38,0.41) |
|  | INC2 | 0.64 (0.59,0.68) | 0.65 (0.62,0.68) | 0.59 (0.53,0.66) | 0.48 (0.46,0.50) |
|  | INC3 | 0.71 (0.66,0.76) | 0.71 (0.68,0.75) | 0.69 (0.62,0.77) | 0.58 (0.56,0.61) |
|  | INC4 | 0.85 (0.79,0.92) | 0.82 (0.78,0.86) | 0.86 (0.77,0.97) | 0.74 (0.71,0.78) |
|  | INC5 least deprived quintile | 1 | 1 | 1 | 1 |
| Maternal deprivation (MATDPR) | MATDPR_1 least deprived quintile | 1 | 1 | 1 | 1 |
|  | MATDPR_2 | 0.87 (0.81,0.94) | 0.87 (0.82,0.91) | 0.85 (0.76,0.95) | 0.83 (0.80,0.87) |
|  | MATDPR_3 | 0.74 (0.69,0.80) | 0.76 (0.72,0.79) | 0.75 (0.67,0.84) | 0.70 (0.67,0.73) |
|  | MATDPR_4 | 0.66 (0.61,0.71) | 0.70 (0.66,0.73) | 0.67 (0.6,0.75) | 0.63 (0.60,0.66) |
|  | MATDPR_5 most deprived quintile | 0.59 (0.55,0.63) | 0.61 (0.58,0.65) | 0.53 (0.48,0.59) | 0.49 (0.47,0.51) |
|  | Hospital stay in days | 1.00 (1.00,1.00) | 1.00 (1.00,1.00) | 1.00 (1.00,1.00) | 1.00 (1.00,1.00) |
|  | Asthma >1 prescription | 0.98 (0.94,1.03) | 0.98 (0.94,1.01) | 1.01 (0.94,1.09) | 0.97 (0.93,1.00) |
|  | Epilepsy >1 prescription | 0.12 (0.10,0.13) | 0.16 (0.14,0.18) | 0.26 (0.20,0.34) | 0.34 (0.26,0.44) |
|  | Diabetes >1 insulin prescription | 0.94 (0.64,1.43) | 1.07 (0.79,1.49) | 0.76 (0.42,1.52) | 0.76 (0.54,1.07) |

**Notes to table: *aOR*** *adjusted odds ratio, ATS access to services, ENV physical environment, EWMS English/ Welsh plus maths + science, FSM free school meals eligibility, FSM free school meals, INC household income levels, KS key stage, MATDPR maternal deprivation, SEN special educational needs*, CI confidence intervals,

*Odds ratios were adjusted for: Child trait (sex, gestational age, and year of birth), deprivation, hospitalisation, co-morbidities, and SEN provision. Including interaction terms in the models widened confidence intervals, but did not change the findings (S4 file).*

*Reference categories for the components of the WIMD were the highest ranked or least deprived quintiles. For maternal deprivation, 1 is the highest rank. For other indices, 5 is the highest rank.*

*VIF <3 for all models. Income and area-based measures of deprivation were associated, but there was no collinearity between FSM eligibility or gestation age and area-based measures of deprivation. By including all parameters, these models identify the most powerful predictors of educational achievement*

*There were no data at age 16 for children born 2003-2007.*

### Table I. Re-analysis of educational outcomes and isolated major congenital anomalies without year of birth

|  |  | **KS2** | **KS2** | **KS4** | **KS4** |
| --- | --- | --- | --- | --- | --- |
|  |  | **Lower level pass** | **High Level (4) pass** | **Any 5 GCSEs** | **5 GCSEs C-A* with EWMS** |
|  |  | aOR (95% CI) | aOR (95% CI) | aOR (95% CI) | aOR (95% CI) |
| Major congenital anomaly | Cleft lip ± cleft palate | 1.43 (0.84,2.57) | 1.12 (0.73,1.73) | 1.78 (0.72,5.47) | 0.75 (0.45,1.27) |
|  | Cleft palate | 1.43 (0.77,2.84) | 1.17 (0.71,1.97) | 1.78 (0.65,6.31) | 0.71 (0.42,1.20) |
|  | CHD (excluding severe CHD) | 0.77 (0.63,0.94) | 0.75 (0.64,0.88) | 0.88 (0.67,1.18) | 0.98 (0.84,1.15) |
|  | Oesophageal atresia | 0.33 (0.04,4.27) | 0.40 (0.08,3.04) | a | a |
|  | Ano-rectal atresia and stenosis | 1.21 (0.19,11.16) | 0.74 (0.18,3.78) | b | 1.32 (0.42,4.74) |
|  | Diaphragmatic hernia | 2.07 (0.40,16.23) | 1.58 (0.41,7.30) | b | 1.41 (0.45,4.85) |
|  | Gastroschisis | 0.56 (0.30,1.06) | 0.75 (0.44,1.29) | 0.85 (0.30,3.14) | 0.48 (0.24,0.96) |
|  | Multi-cystic renal dysplasia | 0.40 (0.19,0.92) | 0.48 (0.26,0.93) | 1.40 (0.45,6.21) | 0.75 (0.40,1.44) |
|  | Hypospadias | 0.86 (0.64,1.16) | 0.87 (0.69,1.11) | 1.01 (0.66,1.58) | 0.77 (0.60,0.98) |
|  | Spina bifida | 4.80 (1.11,33.28) | 1.62 (0.46,5.75) | 0.36 (0.07,2.14) | 0.58 (0.15,2.15) |
|  | Limb reduction defects | 1.70 (0.73,4.35) | 1.99 (0.96,4.30) | 1.87 (0.57,8.50) | 1.80 (0.80,4.11) |
|  | Hydrocephalus | 1.09 (0.53,2.32) | 1.44 (0.72,2.89) | 0.96 (0.36,3.05) | 0.43 (0.13,1.19) |
|  | Craniosynostosis | 0.66 (0.32,1.41) | 0.65 (0.34,1.25) | 0.44 (0.17,1.35) | 1.20 (0.61,2.41) |
|  | Down syndrome | 0.03 (0.01,0.06) | 0.02 (0.00,0.07) | 0.06 (0.02,0.13) | 0.09 (0.01,0.44) |
|  | Turner syndrome | 1.81 (0.58,5.89) | 4.11 (1.37,13.07) | b | 1.00 (0.27,3.72) |
|  | Severe CHD | 1.61 (1.10,2.40) | 1.20 (0.88,1.65) | 0.79 (0.45,1.43) | 0.77 (0.55,1.07) |
|  | Other major congenital anomalies | 0.71 (0.65,0.79) | 0.85 (0.78,0.93) | 0.86 (0.75,0.99) | 1.02 (0.93,1.12) |
| FSM | FSM eligibility | 0.45 (0.43,0.46) | 0.46 (0.45,0.48) | 0.35 (0.33,0.36) | 0.29 (0.28,0.30) |
| Co-morbidities | Asthma > 1 prescription | 1.14 (1.08,1.20) | 1.25 (1.20,1.30) | 0.96 (0.89,1.04) | 0.92 (0.88,0.95) |
|  | Epilepsy > 1 prescription | 0.51 (0.44,0.61) | 0.65 (0.55,0.77) | 0.74 (0.55,1.00) | 0.56 (0.42,0.75) |
|  | Insulin > 1 prescription | 2.25 (1.46,3.63) | 2.81 (2.00,4.05) | 1.30 (0.68,2.77) | 1.25 (0.87,1.80) |
| SEN | SEN A action plus | 0.04 (0.04,0.05) | 0.07 (0.06,0.07) | 0.27 (0.25,0.29) | 0.16 (0.16,0.17) |
|  | SEN P action plan plus | 0.02 (0.02,0.02) | 0.03 (0.03,0.03) | 0.08 (0.07,0.08) | 0.12 (0.11,0.12) |
|  | SEN S statemented | 0.00 (0.00,0.00) | 0.01 (0.01,0.01) | 0.03 (0.03,0.03) | 0.07 (0.06,0.07) |
| Hospitalisation | Each day hospitalised | 1.00 (1.00,1.00) | 1.00 (1.00,1.00) | 1.00 (1.00,1.00) | 1.00 (1.00,1.00) |
| Model  Parameters | -2LL (log likelihood) | 76262 | 130550 | 42442 | 149230 |
|  | Nagelkerke R^2^ | 0.39 | 0.36 | 0.25 | 0.16 |
|  | Goodness of fit χ^2^ | 75.01 | 148.57 | 120.66 | 178.45 |

a The low number of children with this major congenital anomaly preclude reporting results.

b There were very few children with these major congenital anomalies in the data at KS4. Most of these children obtained 5 GCSEs.

*ATS access to services, ENV physical environment, EWMS English/ Welsh plus maths + science, FSM free school meals eligibility, INC household income levels, SEN special educational needs*, aOR adjusted odds ratio, CI confidence interval.

*Odds ratios were adjusted for: Child trait (sex, gestational age), deprivation, hospitalisation, co-morbidities, and SEN provision*

### Table J. Re-analysis of educational outcomes with year of birth as a continuous variable

|  |  | **KS2** | **KS2** | **KS4** | **KS4** |  |
| --- | --- | --- | --- | --- | --- | --- |
|  |  | **Lower level pass** | **High Level (4) pass** | **Any 5 GCSEs** | **5 GCSEs C-A* with EWMS** |  |
|  |  | aOR (95% CI) | aOR (95% CI) | aOR (95% CI) | aOR (95% CI) |  |
|  | Any major congenital anomaly | 0.72 (0.66,0.78) | 0.83 (0.77,0.89) | 0.78 (0.70,0.87) | 0.93 (0.87,1.00) |  |
|  | Year | 1.09 (1.08,1.10) | 1.17 (1.17,1.18) | 0.95 (0.93,0.97) | 0.97 (0.96,0.98) |  |
| Sex | Male | 1.14 (1.10,1.19) | 1.10 (1.07,1.14) | 0.87 (0.82,0.92) | 0.81 (0.79,0.83) |  |
| Gestational age | Pre-term 32-36+6 weeks | 1.00 (0.86,1.18) | 0.84 (0.74,0.96) | 1.55 (1.21,2.00) | 0.94 (0.81,1.08) |  |
|  | Very pre-term <32 weeks | 0.88 (0.82,0.95) | 0.91 (0.86,0.97) | 1.07 (0.96,1.18) | 1.02 (0.97,1.08) |  |
| FSM | FSM1 eligibility | 0.53 (0.51,0.55) | 0.54 (0.52,0.56) | 0.42 (0.40,0.45) | 0.39 (0.38,0.41) |  |
| Access to services | ATS1 – most deprived quintile | 1.03 (0.97,1.10) | 0.98 (0.93,1.03) | 0.92 (0.83,1.01) | 0.89 (0.85,0.93) |  |
|  | ATS2 | 0.91 (0.86,0.97) | 0.94 (0.90,0.98) | 0.90 (0.82,0.98) | 0.86 (0.83,0.90) |  |
|  | ATS3 | 0.89 (0.84,0.95) | 0.93 (0.89,0.97) | 0.85 (0.78,0.93) | 0.87 (0.84,0.91) |  |
|  | ATS4 | 0.98 (0.92,1.05) | 0.97 (0.93,1.02) | 0.88 (0.81,0.96) | 0.89 (0.86,0.93) |  |
|  | Access to services – least deprived quintile | 1.00 | 1.00 | 1.00 | 1.00 |  |
| Physical  Environment | ENV1 | 1.12 (1.05,1.18) | 1.02 (0.97,1.06) | 0.92 (0.85,1.00) | 1.03 (0.98,1.07) |  |
|  | ENV2 | 1.13 (1.06,1.19) | 1.06 (1.02,1.11) | 0.94 (0.87,1.03) | 0.97 (0.94,1.01) |  |
|  | ENV3 | 1.10 (1.03,1.16) | 1.03 (0.99,1.08) | 0.95 (0.87,1.03) | 0.98 (0.94,1.02) |  |
|  | ENV4 | 1.11 (1.05,1.18) | 1.07 (1.02,1.12) | 1.02 (0.93,1.11) | 1.01 (0.97,1.05) |  |
|  | Environment 5 – least deprived quintile | 1.00 | 1.00 | 1.00 | 1.00 |  |
| Child’s household’s  income at  Assessment | INC1 | 0.65 (0.60,0.70) | 0.67 (0.63,0.72) | 0.54 (0.48,0.60) | 0.40 (0.38,0.42) |  |
|  | INC2 | 0.70 (0.65,0.76) | 0.75 (0.70,0.79) | 0.61 (0.55,0.69) | 0.48 (0.46,0.50) |  |
|  | INC3 | 0.80 (0.74,0.87) | 0.81 (0.76,0.86) | 0.74 (0.66,0.83) | 0.59 (0.56,0.62) |  |
|  | INC4 | 0.94 (0.86,1.02) | 0.90 (0.85,0.96) | 0.86 (0.76,0.97) | 0.74 (0.70,0.77) |  |
|  | 5 Household income – least deprived quintile | 1.00 | 1.00 | 1.00 | 1.00 |  |
| Maternal  Deprivation  At birth | 1 – least deprived quintile | 1.00 | 1.00 | 1.00 | 1.00 |  |
|  | 2 – 4^th^ most deprived quintile | 0.99 (0.91,1.08) | 0.98 (0.92,1.04) | 0.90 (0.79,1.02) | 0.85 (0.81,0.89) |  |
|  | 3 – Central quintile for maternal deprivation | 0.85 (0.78,0.92) | 0.85 (0.80,0.90) | 0.82 (0.73,0.92) | 0.72 (0.69,0.76) |  |
|  | 4 – 2^nd^ most deprived quintile | 0.77 (0.71,0.84) | 0.81 (0.76,0.86) | 0.76 (0.67,0.85) | 0.65 (0.62,0.68) |  |
|  | 5 – most deprived quintile | 0.73 (0.67,0.79) | 0.75 (0.70,0.79) | 0.63 (0.56,0.71) | 0.52 (0.50,0.55) |  |
| Co-morbidities | Asthma >1 prescription | 1.02 (0.97,1.08) | 1.02 (0.99,1.07) | 1.05 (0.97,1.14) | 1.00 (0.96,1.04) |  |
|  | Epilepsy >1 prescription | 0.48 (0.41,0.57) | 0.56 (0.48,0.67) | 0.78 (0.58,1.06) | 0.61 (0.45,0.81) |  |
|  | Insulin >1 prescription | 1.81 (1.20,2.84) | 2.26 (1.61,3.23) | 1.10 (0.59,2.25) | 1.11 (0.76,1.61) |  |
| SEN | A action plan | 0.05 (0.04,0.05) | 0.06 (0.06,0.07) | 0.29 (0.27,0.31) | 0.17 (0.16,0.18) |  |
|  | P action plan plus | 0.02 (0.02,0.02) | 0.03 (0.03,0.03) | 0.08 (0.08,0.09) | 0.13 (0.12,0.14) |  |
|  | S statement | 0.00 (0.00,0.00) | 0.01 (0.01,0.01) | 0.03 (0.03,0.03) | 0.07 (0.06,0.07) |  |
| Hospitalisation | Each day hospitalised | 1.00 | 1.00 | 1.00 | 1.00 |  |
| Model  Parameters | -2LL (log likelihood) | 75102 | 126186 | 41900 | 143720 |  |
|  | Nagelkerke R^2^ | 0.4 | 0.38 | 0.26 | 0.19 |  |
|  | Goodness of fit χ^2^ | 100.19 | 324.06 | 65.28 | 22.35 |  |

*aOR adjusted odds ratio, ATS access to services, CI confidence interval, ENV physical environment, EWMS English/ Welsh plus maths + science, FSM free school meals eligibility, INC household income levels, KS key stage, SEN special educational needs*.

*Odds ratios were adjusted for: Child trait (sex, gestational age, and year of birth), deprivation, hospitalisation, co-morbidities, and SEN provision*

### Table K. Data underlying figure 6: achieving 5 GCSEs at grades C-A* with EWMS at age 16, income deprivation and major congenital anomalies, unadjusted data

| WIMD quintile |  | % Achieving | Number achieving | Unadjusted OR (95% CI) | Total in category | Difference between children with  and without major congenital anomalies |
| --- | --- | --- | --- | --- | --- | --- |
| 1 (Most deprived) | Anomalies | 29.65 | 307 | 0.68 (0.59,0.78) | 1034 | 8.77% |
|  | No anomalies | 38.42 | 10,875 | 1.00 | 28,305 |  |
| 2 | Anomalies | 39.36 | 380 | 0.67 (0.59,0.77) | 965 | 9.70% |
|  | No anomalies | 49.06 | 12,597 | 1.00 | 25,677 |  |
| 3 | Anomalies | 47.04 | 397 | 0.66 (0.58,0.76) | 844 | 10.17% |
|  | No anomalies | 57.21 | 14,190 | 1.00 | 24,803 |  |
| 4 | Anomalies | 56.79 | 440 | 0.68 (0.59,0.79) | 774 | 9.15% |
|  | No anomalies | 65.94 | 15,081 | 1.00 | 22,871 |  |
| 5 (Least deprived) | Anomalies | 67.57 | 547 | 0.69 (0.60,0.81) | 810 | 7.41% |
|  | No anomalies | 74.98 | 16,978 | 1.00 | 22,644 |  |
| Total |  | 55.78 | 71,792 |  | 128,727 |  |

Notes to table: EWMS English/ Welsh Maths Science, GCSE General Certificate of Secondary Education, OR odds ratio, CI confidence interval. Deprivation was recorded from the WIMD income domain. WIMD, Welsh Index of Multiple Deprivation.

### Table L. Analysis of educational attainment stratified by child’s sex

Stratification reduced numbers of children with some major congenital anomalies. For some major congenital anomalies results have been obfuscated to comply with governance restrictions on reporting of small numbers and analyses that might reveal small numbers when tables are compared. Low numbers in cells of the regression models resulted in some models not converging, and only an overall summary of these results is presented.

### Table Li. Re-analysis with boys only

|  |  | **KS2** | **KS2** | **KS4** | **KS4** |
| --- | --- | --- | --- | --- | --- |
|  |  | **Lower level pass** | **High Level (4) pass** | **Any 5 GCSEs** | **5 GCSEs C-A* with EWMS** |
|  |  | aOR (95% CI) | aOR (95% CI) | aOR (95% CI) | aOR (95% CI) |
| Major congenital anomaly | Cleft lip ± cleft palate | 1.92 (1.00,4.01) | 1.07 (0.66,1.77) | 1.86 (0.69,6.59) | 0.64 (0.34,1.18) |
|  | Cleft palate | 1.99 (0.83,5.63) | 1.15 (0.59,2.33) | 1.00 (0.34,3.78) | 0.87 (0.42,1.80) |
|  | CHD (excluding severe CHD) | 0.86 (0.65,1.13) | 0.99 (0.78,1.24) | 1.07 (0.72,1.63) | 1.02 (0.80,1.29) |
|  | Oesophageal atresia | NS^a^ | NS^a^ | a | a |
|  | Ano-rectal atresia and stenosis | NS^a^ | NS^a^ | NS^a^ | NS^a^ |
|  | Diaphragmatic hernia | 3.34 (0.43,72.53) | 1.38 (0.30,8.28) | NS^a^ | NS^a^ |
|  | Gastroschisis | 0.47 (0.21,1.10) | 0.55 (0.28,1.12) | 0.30 (0.09,1.26) | 0.49 (0.17,1.31) |
|  | Multi-cystic renal dysplasia | 0.30 (0.12,0.84) | 0.37 (0.16,0.85) | 1.50 (0.23,29.98) | 0.59 (0.20,1.68) |
|  | Hypospadias | 0.82 (0.62,1.11) | 0.85 (0.67,1.08) | 1.05 (0.69,1.65) | 0.84 (0.66,1.07) |
|  | Spina bifida | NS^a^ | NS^a^ | NS^a^ | NS^a^ |
|  | Limb reduction defects | 0.99 (0.39,2.67) | 1.21 (0.51,2.89) | 1.28 (0.35,6.22) | 1.08 (0.36,3.23) |
|  | Hydrocephalus | 0.76 (0.31,1.89) | 1.29 (0.54,3.07) | 0.98 (0.32,3.69) | 0.66 (0.16,2.18) |
|  | Craniosynostosis | 0.85 (0.35,2.12) | 0.94 (0.42,2.12) | 0.45 (0.16,1.57) | 1.11 (0.52,2.44) |
|  | Down syndrome | 0.02 (0.00,0.07) | 0.02 (0.00,0.11) | 0.03 (0.00,0.10) | b |
|  | Turner syndrome | NA |  |  |  |
|  | Severe CHD | 1.60 (1.02,2.56) | 1.53 (1.05,2.25) | 0.75 (0.40,1.48) | 0.89 (0.59,1.34) |
|  | Other major congenital anomalies | 0.69 (0.61,0.79) | 0.82 (0.73,0.92) | 0.82 (0.69,0.99) | 0.96 (0.85,1.09) |
| FSM | FSM eligibility | 0.46 (0.44,0.49) | 0.47 (0.46,0.49) | 0.35 (0.33,0.38) | 0.30 (0.28,0.31) |
| Co-morbidities | Asthma > 1 prescription | 1.11 (1.04,1.18) | 1.23 (1.18,1.29) | 0.95 (0.86,1.04) | 0.92 (0.88,0.97) |
|  | Epilepsy > 1 prescription | 0.51 (0.41,0.63) | 0.68 (0.55,0.85) | 0.74 (0.50,1.09) | 0.55 (0.36,0.82) |
|  | Insulin > 1 prescription | 1.64 (0.97,2.95) | 2.26 (1.45,3.63) | 1.10 (0.48,2.93) | 1.13 (0.65,1.94) |
| SEN | SEN A action plus | 0.05 (0.04,0.05) | 0.07 (0.07,0.07) | 0.26 (0.23,0.29) | 0.16 (0.15,0.17) |
|  | SEN P action plan plus | 0.02 (0.02,0.02) | 0.03 (0.03,0.03) | 0.07 (0.06,0.07) | 0.10 (0.10,0.11) |
|  | SEN S statemented | 0.00 (0.00,0.00) | 0.01 (0.01,0.01) | 0.03 (0.03,0.03) | 0.08 (0.07,0.09) |
| Hospitalisation | Each day hospitalised | 1.00 (1.00,1.00) | 1.00 (1.00,1.00) | 1.00 (1.00,1.00) | 1.00 (1.00,1.00) |
| Model  Parameters | -2LL (log likelihood) | 46100 | 73808 | 24382 | 75040 |
|  | Nagelkerke R^2^ | 0.38 | 0.36 | 0.27 | 0.17 |
|  | Goodness of fit χ^2^ | 32.46 | 89.94 | 37.24 | 70.58 |

Notes to table: Turner syndrome does not occur in boys

*aOR adjusted odds ratio, ATS access to services, CI confidence interval, ENV physical environment, EWMS English/ Welsh plus maths + science, FSM free school meals eligibility, INC household income levels, KS key stage, SEN special educational needs*.

*Odds ratios were adjusted for: FSM, hospitalisation, co-morbidities, and SEN provision.*

### Table Lii. Re-analysis with girls only

|  |  | **KS2** | **KS2** | **KS4** | **KS4** |
| --- | --- | --- | --- | --- | --- |
|  |  | **Lower level pass** | **High Level (4) pass** | **Any 5 GCSEs** | **5 GCSEs C-A* with EWMS** |
|  |  | aOR (95% CI) | aOR (95% CI) | aOR (95% CI) | aOR (95% CI) |
| Major congenital anomaly | Cleft lip ± cleft palate | 0.62 (0.24,1.77) | 1.21 (0.51,3.06) | 1.85 (0.27,39.23) | 1.39 (0.51,4.07) |
|  | Cleft palate | 1.06 (0.44,2.84) | 1.22 (0.57,2.71) | NS^a^ | 0.58 (0.27,1.23) |
|  | CHD (excluding severe CHD) | 0.69 (0.52,0.94) | 0.57 (0.46,0.72) | 0.70 (0.48,1.06) | 0.93 (0.75,1.15) |
|  | Oesophageal atresia | NS^a^ | NS^a^ | a | a |
|  | Ano-rectal atresia and stenosis | NS^a^ | NS^a^ | NS^a^ | NS^a^ |
|  | Diaphragmatic hernia | 1.28 (0.06,46.37) | 3.16 (0.19,99.58) | NS^a^ | NS^a^ |
|  | Gastroschisis | 0.74 (0.29,2.03) | 1.16 (0.50,2.80) | NS^a^ | 0.48 (0.18,1.24) |
|  | Hypospadias | NA |  |  |  |
|  | Spina bifida | NS^a^ | 6.51 (0.75,139.97) | NS^a^ | NS^a^ |
|  | Limb reduction defects | NS^a^ | 11.60 (1.96,223.07) | NS^a^ | 3.82 (1.06,16.27) |
|  | Hydrocephalus | 2.56 (0.69,12.31) | 1.82 (0.57,6.11) | 0.93 (0.14,18.58) | 0.18 (0.01,1.14) |
|  | Craniosynostosis | 0.37 (0.11,1.46) | 0.34 (0.12,0.99) | 0.42 (0.06,9.44) | 1.89 (0.45,10.63) |
|  | Down syndrome | 0.04 (0.01,0.14) | 0.02 (0.00,0.13) | 0.12 (0.04,0.31) | 0.22 (0.01,1.23) |
|  | Turner syndrome | 2.32 (0.73,7.62) | 4.87 (1.60,15.65) | NS^a^ | 0.92 (0.25,3.46) |
|  | Severe CHD | 1.64 (0.78,3.72) | 0.70 (0.41,1.22) | 0.93 (0.32,3.98) | 0.63 (0.36,1.09) |
|  | Other major congenital anomalies | 0.78 (0.66,0.92) | 0.91 (0.79,1.05) | 0.92 (0.73,1.17) | 1.11 (0.97,1.27) |
| FSM | FSM eligibility | 0.42 (0.40,0.45) | 0.45 (0.43,0.47) | 0.33 (0.30,0.35) | 0.27 (0.26,0.29) |
| Co-morbidities | Asthma > 1 prescription | 1.17 (1.08,1.28) | 1.26 (1.18,1.34) | 1.01 (0.88,1.15) | 0.94 (0.88,0.99) |
|  | Epilepsy > 1 prescription | 0.57 (0.44,0.74) | 0.63 (0.49,0.81) | 0.73 (0.45,1.20) | 0.55 (0.37,0.81) |
|  | Insulin > 1 prescription | 4.04 (1.87,10.61) | 3.89 (2.26,7.15) | 1.66 (0.59,6.93) | 1.31 (0.80,2.17) |
| SEN | SEN A action plus | 0.04 (0.04,0.05) | 0.06 (0.06,0.06) | 0.29 (0.26,0.32) | 0.17 (0.16,0.18) |
|  | SEN P action plan plus | 0.01 (0.01,0.02) | 0.03 (0.03,0.03) | 0.09 (0.08,0.10) | 0.14 (0.13,0.15) |
|  | SEN S statemented | 0.00 (0.00,0.00) | 0.01 (0.01,0.01) | 0.03 (0.02,0.03) | 0.05 (0.04,0.06) |
| Hospitalisation | Each day hospitalised | 1.00 (1.00,1.00) | 1.00 (1.00,1.00) | 1.00 (1.00,1.00) | 1.00 (1.00,1.00) |
| Model  Parameters | -2LL (log likelihood) | 30054 | 56630 | 17970 | 73886 |
|  | Nagelkerke R^2^ | 0.4 | 0.35 | 0.21 | 0.14 |
|  | Goodness of fit χ^2^ | 46.96 | 91.21 | 67.12 | 72.34 |

Notes to table:

a The low number of children with this major congenital anomaly precludes reporting any results.

b no or few children with this major congenital anomaly passed at this level

NS not statistically significant. Models did not converge.

NS^a^ not statistically significant. Models did not converge and low numbers of children with these major congenital anomalies.

Multi-cystic renal dysplasia data in girls deleted in error.

Hypospadias only occurs in boys.

*aOR adjusted odds ratio, ATS access to services, CI confidence interval, ENV physical environment, EWMS English/ Welsh plus maths + science, FSM free school meals, GCSE General Certificate of Secondary Education, INC household income levels, KS key stage, SEN special educational needs*.

*Odds ratios were adjusted for: FSM, hospitalisation, co-morbidities, and SEN provision.*

### Table M. Educational outcomes and SEN for birth years 1998- 2007, unadjusted data

|  |  | **KS2** | **KS2** | | **KS4** | **KS4** |
| --- | --- | --- | --- | --- | --- | --- |
|  |  | **Lower level pass** | **High Level (4) pass** | | **Any 5 GCSEs** | **5 GCSEs C-A* with EWMS** |
|  |  | OR (95% CI) | OR (95% CI) | | OR (95% CI) | OR (95% CI) |
| 1998 | SEN A | 0.05 (0.04,0.06) | 0.07 (0.06,0.07) | | 0.27 (0.23,0.32) | 0.15 (0.13,0.16) |
|  | SEN P | 0.02 (0.02,0.03) | 0.03 (0.03,0.04) | | 0.06 (0.06,0.07) | 0.10 (0.09,0.11) |
|  | SEN S | 0.01 (0.00,0.01) | 0.01 (0.01,0.01) | | 0.03 (0.02,0.03) | 0.05 (0.04,0.07) |
| 1999 | SEN A | 0.04 (0.04,0.05) | 0.07 (0.06,0.07) | | 0.28 (0.23,0.33) | 0.16 (0.14,0.17) |
|  | SEN P | 0.02 (0.02,0.02) | 0.04 (0.03,0.04) | | 0.06 (0.05,0.07) | 0.10 (0.09,0.12) |
|  | SEN S | 0.00 (0.00,0.01) | 0.01 (0.01,0.01) | | 0.02 (0.02,0.03) | 0.05 (0.04,0.06) |
| 2000 | SEN A | 0.04 (0.04,0.05) | 0.06 (0.06,0.07) | | 0.23 (0.19,0.27) | 0.16 (0.15,0.17) |
|  | SEN P | 0.02 (0.02,0.02) | 0.03 (0.03,0.04) | | 0.06 (0.05,0.07) | 0.10 (0.09,0.12) |
|  | SEN S | 0.00 (0.00,0.00) | 0.01 (0.01,0.01) | | 0.02 (0.02,0.02) | 0.06 (0.04,0.07) |
| 2001 | SEN A | 0.03 (0.03,0.04) | 0.06 (0.05,0.06) | | 0.20 (0.17,0.24) | 0.15 (0.13,0.16) |
|  | SEN P | 0.01 (0.01,0.02) | 0.03 (0.02,0.03) | | 0.05 (0.05,0.06) | 0.09 (0.08,0.10) |
|  | SEN S | 0.00 (0.00,0.00) | 0.01 (0.01,0.01) | | 0.02 (0.02,0.03) | 0.06 (0.05,0.08) |
| 2002 | SEN A | 0.04 (0.03,0.05) | 0.05 (0.05,0.06) | | 0.19 (0.16,0.22) | 0.14 (0.13,0.15) |
|  | SEN P | 0.01 (0.01,0.02) | 0.02 (0.02,0.03) | | 0.06 (0.05,0.07) | 0.10 (0.09,0.12) |
|  | SEN S | 0.00 (0.00,0.00) | 0.00 (0.00,0.01) | | 0.02 (0.02,0.03) | 0.05 (0.04,0.06) |
| 2003 | SEN A | 0.03 (0.03,0.04) | 0.05 (0.04,0.06) | |  |  |
|  | SEN P | 0.01 (0.01,0.01) | 0.02 (0.02,0.02) | |  |  |
|  | SEN S | 0.00 (0.00,0.00) | 0.00 (0.00,0.01) | |  |  |
| 2004 | SEN A | 0.03 (0.03,0.04) | 0.04 (0.04,0.05) | |  |  |
|  | SEN P | 0.01 (0.01,0.01) | 0.02 (0.01,0.02) | |  |  |
|  | SEN S | 0.00 (0.00,0.00) | 0.00 (0.00,0.00) | |  |  |
| 2005 | SEN A | 0.03 (0.02,0.04) | 0.03 (0.03,0.04) | |  |  |
|  | SEN P | 0.01 (0.01,0.01) | 0.01 (0.01,0.02) | |  |  |
|  | SEN S | 0.00 (0.00,0.00) | 0.00 (0.00,0.00) | |  |  |
| 2006 | SEN A | 0.02 (0.02,0.03) | 0.04 (0.03,0.04) | |  |  |
|  | SEN P | 0.01 (0.00,0.01) | 0.01 (0.01,0.02) | |  |  |
|  | SEN S | 0.00 (0.00,0.00) | 0.00 (0.00,0.00) | |  |  |
| 2007 | SEN A | 0.03 (0.02,0.05) | 0.04 (0.03,0.04) | |  |  |
|  | SEN P | 0.01 (0.01,0.01) | 0.01 (0.01,0.02) | |  |  |
|  | SEN S | 0.00 (0.00,0.00) | 0.00 (0.00,0.00) | |  |  |

Notes to table: SEN special educational needs, classified as:

| SEN A | Action plan, in school support |
| --- | --- |
| SEN P | Action plan plus, some external advice and support |
| SEN S | Statement completed, specifying internal and external support |

CI confidence intervals, KS key stage, OR odds ration

### Table N. Educational outcomes and deprivation (as FSM eligibility) for birth years 1998-2007

|  | **KS2** | **KS2** | **KS4** | **KS4** |
| --- | --- | --- | --- | --- |
|  | **Lower level pass** | **High Level (4) pass** | **Any 5 GCSEs** | **5 GCSEs C-A* with EWMS** |
|  | OR (95% CI) | OR (95% CI) | OR (95% CI) | OR (95% CI) |
| 1998 | 0.30 (0.28,0.33) | 0.32 (0.30,0.35) | 0.18 (0.17,0.21) | 0.22 (0.20,0.24) |
| 1999 | 0.27 (0.25,0.30) | 0.32 (0.30,0.34) | 0.21 (0.19,0.23) | 0.24 (0.22,0.26) |
| 2001 | 0.26 (0.24,0.29) | 0.29 (0.27,0.31) | 0.21 (0.19,0.24) | 0.22 (0.20,0.23) |
| 2001 | 0.26 (0.24,0.29) | 0.30 (0.28,0.33) | 0.20 (0.18,0.22) | 0.22 (0.21,0.24) |
| 2002 | 0.25 (0.23,0.28) | 0.29 (0.27,0.31) | 0.21 (0.19,0.23) | 0.22 (0.20,0.24) |
| 2003 | 0.24 (0.22,0.27) | 0.27 (0.25,0.29) |  |  |
| 2004 | 0.28 (0.25,0.31) | 0.31 (0.28,0.33) |  |  |
| 2005 | 0.26 (0.23,0.29) | 0.29 (0.26,0.31) |  |  |
| 2006 | 0.26 (0.23,0.29) | 0.28 (0.25,0.30) |  |  |
| 2007 | 0.24 (0.21,0.28) | 0.28 (0.25,0.31) |  |  |

Notes to table: CI confidence intervals, KS key stage, OR odds ratio.

### Table O. Sensitivity analysis, based on FSM eligibility

(unadjusted analysis) FSM remains the most powerful predictor of educational attainment.

|  | KS2 | KS2 pass | KS2 high pass | KS4 | KS4 Any 5 GCSEs | KS4 5 GCSEs C-A* with EWMS |
| --- | --- | --- | --- | --- | --- | --- |
|  | *n* | OR (95% CI) | OR (95% CI) | *n* | OR (95% CI) | OR (95% CI) |
| As reported in data,  Children with data for FSM excluded | 256568 |  |  | 128727 |  |  |
| FSM eligible |  | 0.26 (0.25,0.27) | 0.30 (0.29,0.30) |  | 0.20 (0.19,0.21) | 0.22 (0.22,0.23) |
| All missing values for FSM set to eligible | 264433 |  |  | 129187 |  |  |
| FSM eligible |  | 0.42 (0.41,0.44) | 0.38 (0.38,0.39) |  | 0.37 (0.36,0.39) | 0.48 (0.47,0.50) |
| All missing values for FSM set to ineligible | 264433 |  |  | 129187 |  |  |
| FSM eligible |  | 0.43 (0.41,0.44) | 0.39 (0.38,0.39) |  | 0.32 (0.31,0.34) | 0.26 (0.25,0.27) |

Notes *OR odds ratio, CI confidence intervals, EWMS English/ Welsh plus maths + science, FSM free school meals, GCSE General Certificate of Secondary Education.*

### Table P. Individual major congenital anomalies and educational outcomes, unadjusted data at KS2 (n=256,568) and KS4 (n=128,727) (ages 11 and 16)

|  | **Key Stage 2 – pass** | **Key Stage 2 - high pass** | **Key Stage 4 – any 5 GCSEs** | **Key Stage 4 – 5 GCSEs C-A* with EWMS** |
| --- | --- | --- | --- | --- |
|  | **OR (95% CI)** | **OR (95% CI)** | **OR (95% CI)** | **OR (95% CI)** |
| Cleft lip | 0.59 (0.37,0.99) | 0.51 (0.36,0.74) | 0.86 (0.38,2.44) | 0.55 (0.34,0.85) |
| Cleft palate | 0.66 (0.40,1.20) | 0.64 (0.43,0.98) | 1.02 (0.43,3.37) | 0.63 (0.40,1.00) |
| CHD (excluding severe CHD) | 0.54 (0.46,0.63) | 0.61 (0.54,0.69) | 0.61 (0.48,0.78) | 0.80 (0.70,0.92) |
| Oesophageal atresia | 0.40 (0.11,2.57) | 0.55 (0.17,2.42) |  |  |
| Ano-rectal atresia and stenosis | 0.63 (0.18,3.97) | 0.64 (0.24,2.21) |  | 1.41 (0.49,4.59) |
| Diaphragmatic hernia | 0.80 (0.24,4.97) | 0.82 (0.32,2.81) |  | 1.17 (0.42,3.50) |
| Gastroschisis | 0.35 (0.22,0.59) | 0.46 (0.31,0.72) | 0.57 (0.23,1.90) | 0.42 (0.22,0.77) |
| Multicystic renal dysplasia | 0.44 (0.26,0.83) | 0.56 (0.35,0.92) | 0.88 (0.32,3.62) | 0.66 (0.37,1.17) |
| Hypospadias | 0.52 (0.42,0.67) | 0.60 (0.50,0.73) | 0.64 (0.45,0.96) | 0.63 (0.51,0.78) |
| Spina bifida | 0.67 (0.19,4.17) | 0.40 (0.16,1.11) | 0.20 (0.06,0.87) | 0.49 (0.15,1.47) |
| Limb reduction defects | 0.52 (0.26,1.17) | 0.66 (0.37,1.25) | 0.62 (0.22,2.60) | 0.93 (0.48,1.83) |
| Hydrocephalus | 0.18 (0.10,0.33) | 0.26 (0.15,0.46) | 0.27 (0.11,0.80) | 0.17 (0.06,0.41) |
| Craniosynostosis | 0.35 (0.21,0.61) | 0.44 (0.29,0.71) | 0.38 (0.17,1.00) | 0.98 (0.55,1.78) |
| Down syndrome | 0.00 (0.00, 0.00) | 0.00 (0.00,0.01) | 0.01 (0.00,0.01) | 0.01 (0.00,0.05) |
| Turner syndrome | 0.23 (0.10,0.64) | 0.52 (0.22,1.43) |  | 0.67 (0.22,2.02) |
| Severe CHD | 0.51 (0.38,0.71) | 0.51 (0.40,0.65) | 0.54 (0.34,0.90) | 0.66 (0.50,0.89) |
| Other major congenital anomalies | 0.28 (0.26,0.30) | 0.39 (0.36,0.41) | 0.43 (0.38,0.49) | 0.71 (0.66,0.77) |

*Notes to Table.* *OR odds ratio, CI confidence intervals, CHD congenital heart defect, EWMS English/ Welsh plus maths + science, FSM free school meals, GCSE General Certificate of Secondary Education, KS key stage.*

*There are low numbers of children with many of the isolated* major congenital *anomalies (Table 4), affecting the stability of the models and confidence intervals. Where low numbers would be disclosed, data are not reported. For 5 GCSEs C-A* with EWMS, associations with cleft palate, oesophageal or ano-rectal atresia, diaphragmatic hernia, multicystic renal dysplasia, spina bifida, limb reduction defects, craniosynostosis and Turner syndrome were not statistically significant. Down syndrome had a stronger association with outcomes than other anomalies, and might be considered an ‘outlier’. This may have implications for generalisation of the findings.*

### Table Q. Education outcomes and major congenital anomalies, adjusted for co-morbidities and SEN, modelled without deprivation

|  | **Key Stage 2 – pass** | **Key Stage 2 - high pass** | **Key Stage 4 – 5 GCSEs** | **Key Stage 4 5 GCSEs with EWMS** |
| --- | --- | --- | --- | --- |
|  | **aOR (95% CI)** | **aOR (95% CI)** | **aOR (95% CI)** | **aOR (95% CI)** |
| Cleft lip | 1.31 (0.77,2.33) | 1.04 (0.69,1.60) | 1.68 (0.70,5.03) | 0.75 (0.45,1.25) |
| Cleft palate | 1.33 (0.72,2.62) | 1.10 (0.67,1.84) | 1.68 (0.63,5.87) | 0.73 (0.44,1.22) |
| CHD (excluding severe CHD) | 0.77 (0.63,0.94) | 0.76 (0.65,0.89) | 0.87 (0.67,1.16) | 0.97 (0.83,1.14) |
| Severe CHD | 1.58 (1.08,2.34) | 1.20 (0.88,1.64) | 0.84 (0.50,1.51) | 0.82 (0.59,1.14) |
| Oesophageal atresia | 0.28 (0.04,3.11) | 0.37 (0.08,2.57) |  |  |
| Ano-rectal atresia and stenosis | 1.12 (0.20,9.07) | 0.79 (0.19,3.74) |  | 1.27 (0.41,4.58) |
| Diaphragmatic hernia | 2.40 (0.49,17.99) | 1.90 (0.50,8.55) |  | 1.39 (0.45,4.72) |
| Gastroschisis | 0.56 (0.31,1.06) | 0.73 (0.43,1.26) | 0.82 (0.28,3.03) | 0.44 (0.22,0.86) |
| Multicystic renal dysplasia | 0.42 (0.20,0.94) | 0.49 (0.26,0.95) | 1.25 (0.40,5.58) | 0.69 (0.37,1.30) |
| Hypospadias | 0.89 (0.67,1.20) | 0.91 (0.72,1.15) | 1.00 (0.67,1.56) | 0.79 (0.62,1.00) |
| Spina bifida | 5.71 (1.34,39.26) | 1.96 (0.56,6.87) | 0.39 (0.08,2.27) | 0.64 (0.17,2.32) |
| Limb reduction defects | 1.54 (0.67,3.91) | 1.80 (0.88,3.87) | 1.88 (0.60,8.34) | 1.94 (0.88,4.30) |
| Hydrocephalus | 1.18 (0.56,2.53) | 1.50 (0.75,3.02) | 1.08 (0.39,3.53) | 0.36 (0.11,1.00) |
| Craniosynostosis | 0.67 (0.33,1.41) | 0.67 (0.36,1.28) | 0.44 (0.17,1.32) | 1.25 (0.65,2.48) |
| Down syndrome | 0.03 (0.01,0.07) | 0.03 (0.00,0.08) | 0.08 (0.03,0.16) | 0.11 (0.01,0.52) |
| Turner syndrome | 2.02 (0.64,6.51) | 4.43 (1.47,14.02) |  | 1.23 (0.33,4.68) |
| Other major congenital anomalies | 0.73 (0.66,0.80) | 0.86 (0.79,0.94) | 0.87 (0.76,1.01) | 1.01 (0.92,1.11) |
| Hospitalisation (days) | 1.00 (1.00,1.00) | 1.00 (1.00,1.00) | 1.00 (1.00,1.00) | 1.00 (1.00,1.00) |
| Asthma >1 prescription | 1.10 (1.05,1.16) | 1.21 (1.17,1.26) | 0.94 (0.87,1.01) | 0.90 (0.86,0.93) |
| Epilepsy >1 prescription | 0.51 (0.43,0.60) | 0.64 (0.54,0.75) | 0.72 (0.54,0.96) | 0.57 (0.43,0.75) |
| Insulin >1 prescription | 2.25 (1.46,3.61) | 2.78 (1.98,3.98) | 1.46 (0.77,3.09) | 1.28 (0.89,1.84) |
| SEN A action plan | 0.04 (0.04,0.04) | 0.06 (0.06,0.06) | 0.23 (0.21,0.25) | 0.15 (0.15,0.16) |
| SEN P action plan plus | 0.02 (0.01,0.02) | 0.03 (0.03,0.03) | 0.06 (0.06,0.06) | 0.10 (0.10,0.11) |
| SEN S statemented | 0.00 (0.00,0.00) | 0.01 (0.01,0.01) | 0.03 (0.02,0.03) | 0.06 (0.05,0.06) |

Notes to Table Q. SEN special educational needs, a*OR adjusted odds ratio, CI confidence intervals, CHD congenital heart defect, EWMS English/ Welsh plus maths + science, FSM free school meals, GCSE General Certificate of Secondary Education, KS key stage.*

*Odds ratios were adjusted for: hospitalisation, co-morbidities, and SEN provision.*

Where numbers were low, results were redacted. Achievement was more closely associated with SEN provision than major congenital anomalies. The major congenital anomalies associated with not achieving 5 GCSEs C-A* with EWMS were gastroschisis, and Down syndrome. When FSM was included in the model, hypospadias was also statistically significant. Odds ratios were very similar to those in models including FSM (Table 10).

### Table R. Educational attainment stratified by SEN provision

Stratification reduced numbers of children with some major congenital anomalies. These results have been obfuscated to comply with governance restrictions on reporting of small numbers and analyses that might reveal small numbers when tables are compared. Low numbers in cells of the regression models resulted in some models not converging, and only an overall summary of these results is presented.

| Table Ri. Educational attainment stratified by SEN provision (KS2 low level pass) | | | | | |
| --- | --- | --- | --- | --- | --- |
|  |  | No SEN | Action in school | Action plus | Statement |
|  |  | aOR (95% CI) | aOR (95% CI) | aOR (95% CI) | aOR (95% CI) |
| Major congenital anomaly | Cleft lip ± cleft palate | NS | 0.91 (0.38,2.68) | 1.41 (0.71,3.06) | 3.02 (0.73,14.89) |
|  | Cleft palate | NS | 1.66 (0.48,10.38) | 1.94 (0.78,5.87) | 0.52 (0.08,2.19) |
|  | CHD (excluding severe CHD) | 0.84 (0.43,1.96) | 0.89 (0.62,1.31) | 0.63 (0.46,0.85) | 0.89 (0.56,1.37) |
|  | Oesophageal atresia | a | a | a | a |
|  | Ano-rectal atresia and stenosis | NS^a^ | 0.11 (0.00,2.73) | 0.29 (0.01,7.25) | NS^a^ |
|  | Diaphragmatic hernia | NS^a^ | NS^a^ | 2.37 (0.21,69.85) | 1.96 (0.08,50.48) |
|  | Gastroschisis | 0.25 (0.08,1.51) | 0.81 (0.30,2.80) | 0.82 (0.28,2.69) | 0.20 (0.01,1.09) |
|  | Multi-cystic renal dysplasia | 0.39 (0.09,6.99) | 0.46 (0.14,2.05) | 0.58 (0.14,2.85) | NS^a^ |
|  | Hypospadias | 0.54 (0.23,1.75) | 0.85 (0.51,1.53) | 1.05 (0.67,1.72) | 0.68 (0.35,1.28) |
|  | Spina bifida | NS^a^ | b | c | 3.72 (0.71,27.37) |
|  | Limb reduction defects | NS^a^ | 0.77 (0.20,5.05) | 2.48 (0.64,16.38) | 1.79 (0.42,7.68) |
|  | Hydrocephalus | NS | c | 1.27 (0.37,5.82) | 0.74 (0.23,2.05) |
|  | Craniosynostosis | 0.34 (0.07,5.99) | 1.34 (0.26,24.48) | 0.30 (0.07,1.06) | 1.05 (0.31,3.19) |
|  | Down syndrome | NS | NS | 0.04 (0.00,0.20) | 0.02 (0.00,0.05) |
|  | Turner syndrome | NS^a^ | NS^a^ | NS^a^ | 2.33 (0.68,8.43) |
|  | Severe CHD | 0.58 (0.19,3.54) | 1.68 (0.74,4.85) | 1.59 (0.88,3.05) | 1.71 (0.91,3.18) |
|  | Other major congenital anomalies | 0.55 (0.38,0.83) | 0.76 (0.62,0.93) | 0.93 (0.79,1.10) | 0.53 (0.44,0.63) |
| FSM | FSM eligibility | 0.24 (0.21,0.27) | 0.44 (0.42,0.47) | 0.47 (0.44,0.50) | 0.57 (0.51,0.63) |
| Co-morbidities | Asthma > 1 prescription | 1.43 (1.18,1.73) | 1.18 (1.08,1.28) | 1.06 (0.99,1.14) | 1.14 (1.00,1.29) |
|  | Epilepsy > 1 prescription | c | 1.02 (0.65,1.71) | 0.53 (0.41,0.70) | 0.37 (0.28,0.49) |
|  | Insulin > 1 prescription | 1.12 (0.25,19.65) | 1.54 (0.72,3.99) | 4.03 (2.09,8.89) | 0.70 (0.15,2.45) |
| Hospitalisation | Each day hospitalised | 1.00 (1.00,1.00) | 1.00 (1.00,1.00) | 1.00 (1.00,1.00) | 1.00 (1.00,1.00) |
| Model  Parameters* | -2LL (log likelihood) | 12104 | 27606 | 27446 | 8886 |
|  | Nagelkerke R^2^ | 0.04 | 0.02 | 0.03 | 0.04 |
|  | Goodness of fit χ^2^ | 28.93 | 9.41 | 15.18 | 7.09 |

| Table Rii. education attainment stratified by SEN provision (KS2 high) | | | | | |
| --- | --- | --- | --- | --- | --- |
|  |  | No SEN | Action | Action plus | Statement |
|  |  | aOR (95% CI) | aOR (95% CI) | aOR (95% CI) | aOR (95% CI) |
| Major congenital anomaly | Cleft lip ± cleft palate | 0.81 (0.30,3.30) | 0.94 (0.48,1.96) | 1.22 (0.66,2.28) | 2.30 (0.47,9.50) |
|  | Cleft palate | 2.41 (0.53,42.51) | 1.20 (0.52,3.10) | 0.98 (0.44,2.15) | 1.15 (0.17,4.86) |
|  | CHD (excluding severe CHD) | 0.74 (0.54,1.04) | 0.79 (0.61,1.03) | 0.70 (0.52,0.95) | 0.84 (0.47,1.42) |
|  | Oesophageal atresia | a | a | a | a |
|  | Ano-rectal atresia and stenosis | 0.38 (0.07,6.93) | NS^a^ | 0.82 (0.03,20.72) | NS^a^ |
|  | Diaphragmatic hernia | NS^a^ | 0.66 (0.06,14.25) | 1.23 (0.13,12.24) | 4.25 (0.16,109.51) |
|  | Gastroschisis | 0.56 (0.23,1.87) | 0.88 (0.39,2.10) | 0.92 (0.32,2.61) | 0.44 (0.02,2.40) |
|  | Multi-cystic renal dysplasia | 0.30 (0.14,0.79) | 1.05 (0.35,3.87) | 0.55 (0.11,2.28) | NS^a^ |
|  | Hypospadias | 0.98 (0.56,1.90) | 0.78 (0.53,1.17) | 1.05 (0.70,1.58) | 0.57 (0.21,1.24) |
|  | Spina bifida | NS^a^ | 2.57 (0.68,16.71) | 3.22 (0.48,63.13) | 0.78 (0.04,4.93) |
|  | Limb reduction defects | c | b | 2.51 (0.78,9.55) | 0.54 (0.03,3.05) |
|  | Hydrocephalus | NS^a^ | 0.91 (0.24,4.37) | 1.51 (0.47,5.20) | 1.60 (0.50,4.40) |
|  | Craniosynostosis | 1.74 (0.38,31.02) | 0.62 (0.20,2.04) | 0.13 (0.01,0.69) | 1.10 (0.25,3.65) |
|  | Down syndrome | NS | NS | 0.08 (0.00,0.48) | b |
|  | Turner syndrome | NS^a^ | NS^a^ | NS^a^ | 4.84 (1.42,17.37) |
|  | Severe CHD | 0.81 (0.41,1.92) | 1.08 (0.63,1.90) | 1.33 (0.81,2.22) | 1.47 (0.70,2.89) |
|  | Other major congenital anomalies | 0.66 (0.55,0.81) | 0.86 (0.74,1.01) | 1.03 (0.89,1.20) | 0.73 (0.59,0.90) |
| FSM | FSM eligibility | 0.31 (0.29,0.33) | 0.52 (0.50,0.55) | 0.51 (0.49,0.54) | 0.57 (0.50,0.64) |
| Co-morbidities | Asthma > 1 prescription | 1.43 (1.31,1.57) | 1.26 (1.19,1.34) | 1.18 (1.10,1.26) | 1.00 (0.85,1.16) |
|  | Epilepsy > 1 prescription | 0.87 (0.48,1.84) | 1.09 (0.77,1.55) | 0.59 (0.45,0.78) | 0.44 (0.30,0.61) |
|  | Insulin > 1 prescription | 1.10 (0.50,3.10) | 1.49 (0.88,2.65) | 6.73 (3.83,12.85) | 0.87 (0.13,3.42) |
| Hospitalisation | Each day hospitalised | 1.00 (1.00,1.00) | 1.00 (1.00,1.00) | 1.00 (1.00,1.00) | 1.00 (1.00,1.00) |
| Model  Parameters* | -2LL (log likelihood) | 43808 | 46666 | 32780 | 6966 |
|  | Nagelkerke R^2^ | 0.03 | 0.02 | 0.02 | 0.03 |
|  | Goodness of fit χ^2^ | 47.46 | 13.86 | 14.12 | 6.46 |

| Table Riii. Educational attainment stratified by SEN provision (KS4 – any 5 GCSEs) | | | | | | |
| --- | --- | --- | --- | --- | --- | --- |
|  |  | No SEN | Action | Action plus | Statement |  |
|  |  | aOR (95% CI) | aOR (95% CI) | aOR (95% CI) | aOR (95% CI) |  |
| Major congenital anomaly | Cleft lip ± cleft palate | 0.89 (0.19,15.87) | NS | 1.81 (0.48,11.76) | 1.38 (0.22,10.73) |  |
|  | Cleft palate | c | 0.92 (0.17,17.14) | NS | 0.50 (0.06,3.10) |  |
|  | CHD (excluding severe CHD) | 0.65 (0.41,1.12) | 0.82 (0.46,1.64) | 0.99 (0.59,1.75) | 1.04 (0.61,1.80) |  |
|  | Oesophageal atresia | a | b | a | a |  |
|  | Ano-rectal atresia and stenosis | NS^a^ | NS^a^ | b | NS^a^ |  |
|  | Diaphragmatic hernia | NS^a^ | b | NS^a^ | NS^a^ |  |
|  | Gastroschisis | 0.35 (0.10,2.22) | NS^a^ | 1.02 (0.15,20.19) | 1.53 (0.14,33.88) |  |
|  | Multi-cystic renal dysplasia | c | 0.38 (0.05,7.65) | 1.79 (0.26,35.26) | 1.09 (0.10,23.38) |  |
|  | Hypospadias | 2.03 (0.65,12.28) | 0.80 (0.37,2.09) | 0.87 (0.42,1.98) | 0.97 (0.44,2.18) |  |
|  | Spina bifida | NS^a^ | NS^a^ | NS^a^ | 0.36 (0.02,3.91) |  |
|  | Limb reduction defects | NS^a^ | NS^a^ | 1.54 (0.24,30.09) | 1.47 (0.28,10.86) |  |
|  | Hydrocephalus | NS | 0.66 (0.11,12.71) | 1.10 (0.14,22.70) | 1.00 (0.24,4.93) |  |
|  | Craniosynostosis | 0.24 (0.07,1.51) | NS^a^ | 0.29 (0.03,2.45) | 0.49 (0.02,5.35) |  |
|  | Down syndrome | NS^a^ | b | 0.22 (0.01,5.52) | 0.05 (0.02,0.12) |  |
|  | Turner syndrome | NS^a^ | NS^a^ | NS^a^ | NS^a^ |  |
|  | Severe CHD | 2.14 (0.47,37.79) | 0.77 (0.22,4.84) | 0.80 (0.30,2.50) | 0.43 (0.13,1.23) |  |
|  | Other major congenital anomalies | 1.00 (0.71,1.47) | 0.97 (0.66,1.46) | 1.20 (0.90,1.62) | 0.61 (0.48,0.77) |  |
| FSM | FSM eligibility | 0.20 (0.19,0.22) | 0.38 (0.34,0.43) | 0.46 (0.42,0.50) | 0.43 (0.38,0.49) |  |
| Co-morbidities | Asthma > 1 prescription | 0.99 (0.86,1.15) | 0.93 (0.79,1.10) | 0.93 (0.81,1.06) | 1.03 (0.86,1.24) |  |
|  | Epilepsy > 1 prescription | 0.33 (0.16,0.80) | 2.47 (0.76,15.20) | 1.27 (0.63,2.82) | 0.61 (0.41,0.91) |  |
|  | Insulin > 1 prescription | 0.87 (0.27,5.33) | 0.66 (0.23,2.79) | 2.08 (0.81,7.08) | 2.42 (0.19,34.30) |  |
| Hospitalisation | Each day hospitalised | 1.00 (1.00,1.00) | 1.00 (1.00,1.01) | 1.00 (1.00,1.01) | 1.00 (1.00,1.00) |  |
| Model  Parameters* | -2LL (log likelihood) | 17398 | 8606 | 10540 | 5670 |  |
|  | Nagelkerke R^2^ | 0.05 | 0.03 | 0.03 | 0.05 |  |
|  | Goodness of fit χ^2^ | 9.61 | 2.03 | 7.86 | 5.27 |  |

| Table Riv, Educational attainment stratified by SEN provision (KS4 5 GCSEs C-A* with EWMS) | | | | | |
| --- | --- | --- | --- | --- | --- |
|  |  | No SEN | Action | Action plus | Statement |
|  |  | aOR (95% CI) | aOR (95% CI) | aOR (95% CI) | aOR (95% CI) |
| Major congenital anomaly | Cleft lip ± cleft palate | 0.69 (0.38,1.28) | 1.29 (0.35,3.95) | 0.82 (0.13,3.06) | 0.00 (NA,1.45E+41) |
|  | Cleft palate | 0.60 (0.34,1.06) | 0.35 (0.02,1.91) | 3.48 (0.69,14.86) | 2.33 (0.12,16.81) |
|  | CHD (excluding severe CHD) | 0.84 (0.70,1.01) | 1.51 (1.02,2.18) | 2.08 (1.21,3.44) | 0.53 (0.13,1.48) |
|  | Oesophageal atresia | NS^a^ | a | a | a |
|  | Ano-rectal atresia and stenosis | 1.09 (0.33,4.21) | NS^a^ | a | a |
|  | Diaphragmatic hernia | 1.28 (0.38,5.03) | b | NS^a^ | a |
|  | Gastroschisis | 0.43 (0.20,0.91) | 1.21 (0.06,8.86) | 1.15 (0.06,8.06) | NS^a^ |
|  | Multi-cystic renal dysplasia | 0.71 (0.36,1.44) | 0.97 (0.05,7.02) | 2.15 (0.11,15.25) | NS^a^ |
|  | Hypospadias | 0.68 (0.52,0.90) | 1.02 (0.55,1.80) | 1.12 (0.45,2.44) | 1.28 (0.30,3.78) |
|  | Spina bifida | 0.42 (0.10,1.78) | a | a | 4.44 (0.20,50.74) |
|  | Limb reduction defects | 1.08 (0.41,3.35) | c | 1.04 (0.05,6.74) | 1.67 (0.09,10.84) |
|  | Hydrocephalus | 0.52 (0.12,2.06) | b | 2.05 (0.10,17.13) | NS^a^ |
|  | Craniosynostosis | 1.69 (0.76,4.32) | 0.56 (0.03,3.39) | NS^a^ | NS^a^ |
|  | Down syndrome | NS^a^ | b | NS | b |
|  | Turner syndrome | 1.04 (0.22,7.25) | NS^a^ | NS^a^ | 4.94 (0.21,64.10) |
|  | Severe CHD | 0.71 (0.49,1.03) | 1.15 (0.47,2.56) | 0.86 (0.20,2.60) | 0.74 (0.04,3.88) |
|  | Other major congenital anomalies | 0.95 (0.86,1.06) | 1.14 (0.89,1.44) | 1.63 (1.23,2.13) | 0.89 (0.59,1.30) |
| FSM | FSM eligibility | 0.28 (0.27,0.29) | 0.29 (0.26,0.33) | 0.35 (0.30,0.40) | 0.33 (0.25,0.42) |
| Co-morbidities | Asthma > 1 prescription | 0.90 (0.86,0.94) | 1.01 (0.90,1.12) | 0.97 (0.83,1.13) | 0.91 (0.66,1.23) |
|  | Epilepsy > 1 prescription | 0.61 (0.43,0.87) | 0.54 (0.22,1.13) | 0.73 (0.29,1.55) | 0.17 (0.03,0.55) |
|  | Insulin > 1 prescription | 0.83 (0.54,1.29) | 2.00 (0.90,4.30) | 2.51 (1.15,5.19) | 6.26 (0.25,158.61) |
| Hospitalisation | Each day hospitalised | 1.00 (1.00,1.00) | 1.00 (1.00,1.00) | 1.00 (1.00,1.00) | 1.00 (1.00,1.00) |
| Model  Parameters* | -2LL (log likelihood) | 120728 | 17170 | 8552 | 2674 |
|  | Nagelkerke R^2^ | 0.03 | 0.03 | 0.03 | 0.05 |
|  | Goodness of fit χ^2^ | 157.57 | 9.76 | 7.04 | 3.42 |

Notes to table Ri-iv:

a The low number of children with this major congenital anomaly preclude reporting results.

b no or few children with this major congenital anomaly passed at this level

c all or almost all children in this category passed

NS not statistically significant. Models did not converge.

NS^a^ not statistically significant. Models did not converge and low numbers of children with these major congenital anomalies.

*aOR adjusted odds ratio, CHD congenital heart defect, CI confidence interval, EWMS English/ Welsh plus maths + science, FSM free school meals, GCSE General Certificate of Secondary Education, KS key stage, SEN special educational needs*.

*Odds ratios were adjusted for: FSM, hospitalisation, co-morbidities, and SEN provision.*

### S1 Appendix List. EUROCAT major congenital anomalies in this study as in EUROCAT Guide 1.4

This list presents the EUROCAT classification used to determine the ‘all major congenital anomalies’ and ‘other major congenital anomalies’ categories. Subgroups are as listed in section 3.3 of the EUROCAT guide 1.4. Exclusions are as described in sections 3.2 & 3.3.

The anomalies not analysed separately were analysed in the ‘other anomalies’ group.

| **EUROCAT Subgroups** | **ICD10-BPA** | **Analysed separately** |
| --- | --- | --- |
| **All isolated structural major congenital anomalies*** | Q-chapter, D215, D821, D1810^a^, P350, P351, P371 | Yes |
| **Nervous System** |  |  |
| Encephalocele | Q01 | No |
| Spina Bifida | Q05 | Yes |
| Hydrocephalus | Q03 | Yes |
| Severe microcephaly | Q02 | No |
| Arhinencephaly/holoprosencephaly | Q041, Q042 | No |
| **Eye** |  |  |
| Anophthalmos / microphthalmos | Q110, Q111, Q112 | No |
| Anophthalmos | Q110, Q111 | No |
| Congenital cataract | Q120 | No |
| Congenital glaucoma | Q150 | No |
| **Ear, Face and Neck** |  |  |
| Anotia | Q160 | No |
| **Congenital Heart Defects (CHD)** |  |  |
| All CHD | Q20-Q26 | Yes |
| Severe CHD | Q200, Q201, Q203, Q204, Q212, Q213, Q220, Q224, Q225, Q226, Q230, Q232, Q233, Q234, Q251, Q252, Q262 | Yes |
| Common arterial truncus^b^ | Q200 | As severe CHD |
| Double outlet right ventricle^b^ | Q201 | As severe CHD |
| Transposition of great vessels^b^ | Q203 | As severe CHD |
| Single ventricle^b^ | Q204 | As severe CHD |
| Ventricular septal defect | Q210 | As CHD |
| Atrial septal defect | Q211 | As CHD |
| Atrioventricular septal defect^b^ | Q212 | As severe CHD |
| Tetralogy of Fallot^b^ | Q213 | As severe CHD |
| Triscuspid atresia and stenosis^b^ | Q224 | As severe CHD |
| Ebstein’s anomaly^b^ | Q225 | As severe CHD |
| Pulmonary valve stenosis | Q221 | As CHD |
| Pulmonary valve atresia^b^ | Q220 | As severe CHD |
| Aortic valve atresia/stenosis^b^ | Q230 | As severe CHD |
| Mitral valve anomalies^b^ | Q232, Q233 | As severe CHD |
| Hypoplastic left heart^b^ | Q234 | As severe CHD |
| Hypoplastic right heart^b^ | Q226 | As severe CHD |
| Coarctation of aorta^b^ | Q251 | As severe CHD |
| Aortic atresia/interrupted aortic arch^b^ | Q252 | As severe CHD |
| Total anomalous pulmonary venous return^b^ | Q262 | As severe CHD |
| PDA as only CHD in term infants (GA +37 weeks) | Q250 | As CHD |
| **Respiratory** |  |  |
| Choanal atresia | Q300 | No |
| Cystic adenomatous malf of lung | Q3380 | No |
| **Oro-facial clefts** |  |  |
| Cleft lip with or without cleft palate | Q36, Q37 | Yes |
| Cleft palate | Q35~ | Yes |
| **Digestive System** |  |  |
| Oesophageal atresia with/ without trachea-oesophageal fistula | Q390-Q391 | Only at age 11 |
| Duodenal atresia or stenosis | Q410 | No |
| Atresia or stenosis of other parts of small intestine | Q411-Q418 | No |
| Ano-rectal atresia and stenosis | Q420-Q423 | Yes |
| Hirschsprung’s disease | Q431 | No |
| Atresia of bile ducts | Q442 | No |
| Diaphragmatic hernia | Q790 | Yes |
| **Abdominal wall defects** |  |  |
| Gastroschisis | Q793 | Yes |
| Omphalocele | Q792 | No |
| **Urinary** |  |  |
| Multicystic renal dysplasia | Q6140, Q6141 | Yes |
| Congenital hydronephrosis | Q620 | No |
| *Bilateral* renal agenesis, including Potter syndrome | Q601, Q606 | No |
| Bladder exstrophy and / or epispadias | Q640, Q641 | No |
| Posterior urethral valve and / or prune belly | Q6420, Q794 | No |
| **Genital** |  |  |
| Hypospadias | Q54~ | Yes |
| Indeterminate sex | Q56 | No |
| **Limb** |  |  |
| Limb reduction defects | Q71-Q73 | Yes |
| Club foot – talipes equinovarus | Q660 | No |
| Hip dislocation and/or dysplasia | Q650-Q652, Q6580, Q6581 | No |
| Polydactyly | Q69 | No |
| Syndactyly | Q70 | No |
| **Other major congenital anomalies** |  |  |
| Skeletal dysplasias | Q7402, Q77, Q7800, Q782-Q788 | No |
| Craniosynostosis | Q750 | Yes |
| Constriction bands | Q7980 | No |
| Situs inversus | Q893 | No |
| Skin disorders | Q80-Q82 | No |
| Vascular disruptions | Q0435, Q411, Q412, Q418, Q710, Q712, Q713, Q720, Q722, Q723, Q730, Q793, Q795, Q7980, Q7982, Q8706 | No |
| Laterality disruptions | Q206, Q240, Q3381, Q890, Q893 | No |
| Teratogenic syndromes with malformations | Q86, P350, P351, P371 | No |
| Genetic syndromes and microdeletions | Q4471, Q6190, Q7484, Q751, Q754, Q7581, Q87, Q936, D821 |  |
| **Chromosomal anomalies** |  |  |
| Down syndrome | Q90 | Yes |
| Turner syndrome | Q96 | Yes |

* All major congenital anomalies = all cases of major congenital anomaly, excluding cases with only minor congenital anomalies as defined in Section 3.2 in Guide 1.4 for cases born post-2005. Cases with more than one major congenital anomaly are only counted once in the “All major congenital anomalies” subgroup.

^a^ ICD10 code D1810 is the code for cystic hygroma

^b^ Included in Severe CHD subgroup

~As per the EUROCAT classification of congenital anomalies Q357 (cleft uvula) and Q544 chordee were excluded from the analysis as both these conditions are categorized as minor congenital anomalies.

CHD, congenital heart defect; GA, gestational age; ICD10-BPA, international classification of diseases (in collaboration with the British Paediatric Association). PDA, patent ductus arteriosus

Based on EUROCAT 2013. Chapter 3.3: EUROCAT Subgroups of Congenital Anomalies (version 23.09.2016). *EUROCAT Guide 1.4: Instruction for the registration of congenital anomalies (Last update version 20/12/2016).* Newtownabbey, UK: EUROCAT Central Registry, University of Ulster. <https://eu-rd-platform.jrc.ec.europa.eu/system/files/public/JRC-EUROCAT-Full%20Guide%201%204%20version%2022-Nov-2021.pdf>

And GLINIANAIA, S. V., MCLEAN, A., MOFFAT, M., SHENFINE, R., ARMAROLI, A. & RANKIN, J. 2021. Academic achievements and needs of school-aged children with selected congenital anomalies: a systematic review and meta-analysis. *Birth Defects Research,* 113**,** 1431-1462. supplementary material

## Abbreviations

| aOR | Adjusted odds ratio |
| --- | --- |
| ATS | Access to services |
| CHD | Congenital heart defect |
| CI | Confidence intervals / limits |
| EDUW | Education data for Wales |
| EWMS | English or Welsh/ Cymraeg (as first language), maths and science |
| FSM | Free school meals |
| GCSE | General Certificate of Secondary Education |
| ICD10 | International Classification of Diseases version 10 |
| KS | Key stage |
| MaCA | Major congenital anomaly |
| OR | Odds ratio |
| SAIL | Secure Anonymised Information Linkage |
| SEN | Special educational needs |
| SES | Socio-economic status |
| WIMD | Welsh Index of Multiple Deprivation |
