# Supplementary material for: Associations of major congenital anomalies, long-term health conditions, and socioeconomic deprivation with educational attainment in Wales: A population-based data linkage study
Source: PLoS Med. 2026 Oct 16;23(10):e1005050. doi: 10.1371/journal.pmed.1005050 (PMC13705610; doi:10.1371/journal.pmed.1005050)
Supplement: Supplementary file 3 [file pmed.1005050.s003.docx]

**EUROlinkCAT Work package (WP) 5**

**Educational achievements and needs of children with congenital anomalies Study Protocol**

**Institutions responsible:**

**WP5 leads:** Judith Rankin (UNEW), Amanda Neville (UNIFE)

**Researchers:** Svetlana Glinianaia (UNEW)

# 1. Background and aim

Survival beyond infancy (the first year of life) is improving for many children born with congenital anomalies due to advances in neonatal care and operative interventions.^1-4^ Therefore, it is becoming increasingly important to study children’s school performance and their educational needs as there may be a growing population of children and young people requiring additional support and resources in the future. However, apart from the more common genetic syndromes (e.g. Down syndrome) and structural anomalies (e.g. congenital heart defects, CHDs, oral clefts), the evidence of educational achievements and needs for children with other specific congenital anomalies is lacking. The existing published evidence indicates that children born with specific types of congenital anomalies are at a higher risk of lower academic achievements and may require additional support at school. For example, a recent American study reported that a significantly lower percentage of children born with a severe CHD and requiring a CHD surgery in infancy achieved grade 3 and 4 proficiency in literacy and mathematics compared with grade-matched state students.^5^ Compared with all public school children, children with severe and/or complex CHD were significantly more likely to receive special education services^5,6^ or extra support at school.^7^ Few studies, however, used population-based registry data linked to the educational records to explore academic outcomes in children with CHDs of different severity.^8^ Most studies included only specific subsets of children who underwent a CHD surgery in infancy and thereby were limited to children with more severe and complex CHD types or to a specific CHD type.^5,6,9-11^

For children with other congenital anomalies, for example, with isolated oro-facial clefts, there is further international evidence that these children/adolescents were also at a higher risk of lower academic achievements at school and special education needs than the background school population.^12-14^ Thus in a longitudinal American study, these children were at a greater risk of persistent low achievement from elementary school through high school than their classmates (by 45%, 63% and 73%, for maths, reading and language respectively after adjusting for socio-economic differences).^13^ Another recent population-based study in England assessing early academic achievement in children with isolated clefts found that academic achievement at age 5 years was lower than the national average for all six assessed areas, differed by cleft type (i.e. was worse for those with cleft palate with/without cleft lip than for those with cleft lip only) and that special education needs were high varying from 13.2% for cleft lip to 47.6% for bilateral cleft lip and palate.^14^ A recent Danish study exploring the association between anaesthesia-related neurotoxicity, timing of surgery and type of oral clefts, and academic performance in adolescents (15-16 year-olds) measured by test scores and nonattainment at ninth-grade final exams, reported that the type of oral cleft was more important for academic achievements than timing and number of exposures to surgery and anaesthesia.^15^ However, this evidence needs to be confirmed in other settings.

Previously, the majority of children with chromosomal abnormalities associated with intellectual disability, such as Down syndrome, attended school in special education settings, however this attitude has changed over the last two decades resulting in a growing percentage of children with Down syndrome being integrated in the mainstream schools.^16-18^ The more inclusive approach demonstrated great benefits for the academic development of these children,^19-21^ however, for other chromosomal syndromes these issues are much less studied. A study using Wales’ registry (CARIS) data reported that a fifth of girls with Turner syndrome required a significant amount of special education needs and 35% some additional support at school.^22^ This needs to be explored in other geographical locations and confirmed for other conditions.

Combined data from European registries of congenital anomalies linked to educational data within the WP5 of the EUROlinkCAT study will provide an opportunity to analyse population-based data on educational achievements and needs of children born with different types of congenital anomalies up to and including the school year when the child turns 16 years.

**The aim of WP5** is to expand the knowledge on the educational achievements and needs of children with specific congenital anomalies and to provide predictions of future need.

The first **specific task** of WP5 was to identify the data available on education across countries of Europe and address issues in combining it. A survey of all 21 participating registries was conducted and the results summarised in a survey report published on the EUROlinkCAT website in September

2017 (UNEW) [(https://www.eurolinkcat.eu/wp5-](https://www.eurolinkcat.eu/wp5-education/educationalachievementsandneedsofchildrenwithcongenitalanomalies)

[education/educationalachievementsandneedsofchildrenwithcongenitalanomalies](https://www.eurolinkcat.eu/wp5-education/educationalachievementsandneedsofchildrenwithcongenitalanomalies) ). This survey confirmed that nine registries initially proposed in the EUROlinkCAT application as potential WP5 participants would have access to individual-level education data and an additional registry (Italy:

Tuscany) was identified as a further participant.

There are **three studies included in the** WP5 with the following aims:

1. To determine the educational achievements and needs of children born with a congenital anomaly by congenital anomaly subgroup, including those with multiple anomalies (UNEW)
2. To evaluate if educational achievements and needs are associated with clinical (the use of anaesthesia, surgery, days spent in hospital) and sociodemographic factors (gender, maternal age, socioeconomic status) (UNEW)
3. To undertake statistical modelling of data to provide predictions of the number of children with congenital anomalies across Europe up to the child’s 10^th^ birthday who may have specific educational needs (UNEW)

# 2. Description of data sources and requested data

## 2.1. Inclusion criteria

The WP5 survey confirmed that the following ten EUROCAT registries would be able to participate in WP5 studies: England (five BINOCAR registries: Thames Valley, Wessex, East Midlands, South West and North), UK: Wales (CARIS), Denmark: Odense (RSD), Finland (THL), Italy: Emilia Romagna (UNIFE), Italy: Tuscany (CNR-IFC). Norway registry joined WP5 in December 2017 after obtaining ethics approval to use an existing linked dataset with restricted information on educational outcomes for 1999-2014 births.

Study cases are all live born infants with a major congenital anomaly as defined in EUROCAT (Guide 1.4) born between 1995 and 2011 (so that they reach the appropriate school age by the end of 2015) in six participating registries (England: Thames Valley, England: Wessex , Denmark: Odense, Finland, Italy: Emilia Romagna, Italy: Tuscany) or from the first year of EUROCAT data collection in other registries (1998: UK: East Midlands, UK: Wales; 1999: Norway; 2000: UK: North; 2005: UK: South West) to 31^st^ Dec 2011, and confirmed to be alive at start of school in the WP3 linkage with mortality data.

Where available, controls will be all or a selection of live born infants born without congenital anomalies during the same time period and in the same geographical area (Table 4).

The WP2 team will develop some data quality indicators to determine inclusion of a registry’s data in the studies analysing the effect of the risk factors (e.g. sociodemographic) on education outcomes.

## 2.2. Data file from the EUROCAT registry

Each registry will use a file of all live born congenital anomaly cases extracted from their most recent version of EUROCAT Data Management Program (EDMP version 6.10, January 2016) that are confirmed to be alive on their 10^th^ birthday by the end of 2015 based on the WP3 linkage with mortality data (i.e. all cases resulting in deaths should be excluded). Coding and classification of the congenital anomalies will be based on the coding of anomalies and subgroups in the EUROCAT registry (see Guide 1.4). EUROCAT variables to include are listed in Table 1.

These live born congenital anomaly cases will then be linked to data in education databases or any education data sources available locally for use in this study. The registry or the education data provider will be asked to produce a short report outlining the number of cases that were linked/unlinked and the reasons for non-linkage based on a standard template. This report should be sent to Ulster University.

## 2.3. Education databases and education data variables

The list of the Education data sources in the participating registries is given in Appendix 1.

We are going to request education data up to and including the school year when the child turns 16 years. Education data variables vary between the five BINOCAR registries and the other six registries by their types, names, the coding scheme used and the period of availability and will be standardised by the Standardisation Committee and a common data model specified.

The extensive lists of the education variables available for five BINOCAR registries from the National

Pupil Database (NPD) and for the Wales registry from the Secure Anonymised Information Linkage

(SAIL) database are available for members on the EUROlinkCAT website: [http://www.EUROlinkCAT.eu/wp2-buildingresultsrepository/variables.](http://www.eurolinkcat.eu/wp2-buildingresultsrepository/variables) The full lists of the NPD and SAIL variables will be provided in the WP5 BINOCAR- and CARIS-specific protocols for Ethics approval submissions and the NPD and SAIL data requests. The examples of the NPD and SAIL education variables are shown in Table 2. The comprehensive list of the standardised variables will be provided at a later stage of the study.

Education data for Norway are limited to the year when the following educational levels were first attained: basic schooling, lower secondary, upper secondary.

Lists of the education variables are not available for Finland and Italy: Emilia Romagna (as of April 2018). The Finland registry cannot provide the exact education variables before the submission of the data request to the Statistics Finland, Register on Education. They expect to obtain education data covering pre-primary education, primary education and the type of school the students attended (comprehensive, special schools at the basic level of education); there are no obligatory official exams during the compulsory school in Finland (7-16 years) but the grades at the compulsory school graduation may be available.

## 2.4. Data on risk factors

***EUROCAT variables***

Data for the following risk factors are requested form the EUROCAT registries (see Table 1 below):

Child’s sex (EUROCAT core variable 4 – SEX),

Plurality (number of babies delivered – singleton or multiple) (EUROCAT core variable 5 – NBRBABY)

Birth weight (grams) (EUROCAT core variable 10 – WEIGHT)

Gestational age (completed weeks) (EUROCAT core variable 11 – GESTLENGTH)

Maternal age (years) (EUROCAT core variable 15 – AGEMO (age of mother at delivery)

***Data from WP3 and WP4 (national health care databases)*** - see Table 3. ***Non-EUROCAT variables:***

***SES variables:***

Deprivation index at maternal residence (L_MATDEPR_IND – five BINOCAR registries; Italy: Emilia Romagna)

Maternal Education at time of birth (L_MAT_EDUC – Finland; Norway; Italy: Tuscany; Italy: Emilia Romagna)

Maternal country of birth (L_MAT_CTRY_B - Denmark: Odense; Finland; Italy: Tuscany; Italy: Emilia Romagna; UK: Wales)

Marital status (L_MATMAR_STA – Italy: Tuscany)

***WP4 morbidity variables (Table 3)***

The morbidity variables are important to consider in the analysis of the academic achievement. Among these, anaesthesia during surgery is one of the major factors, in particular during the first 18-24 months after birth. We will be using the health database variables listed in Table 3 that can be considered as proxy measures of condition severity (e.g. number of days in hospital/intensive care unit, number of day on ventilator), use of anaesthesia (surgery and surgery type) and associated morbidity, and may be associated with children’s academic performance and special education needs. As the information on the number or duration of general anaesthesia episodes for each case will not be available, the accuracy of the data on surgery type is very important.

## 2.5. Controls

Where possible, as in WP4 study, controls will be all children in the population born in the same geographical area and within the same time period that were not recorded in the EUROCAT registry.

In England, only data from a sample of these control children is available (Table 4).

## 2.6. The linked data file

The linked data file will be stored securely, either within the local registry or within the organisation doing the linkage. The registries will be provided with a set of instructions (syntax script, see section 3 below) to create pre-specified tables and perform analysis of the data and the aggregated tables and analytic results will be transmitted to the Central Results Repository (CRR) at Ulster University. No individual case data will be transmitted to the CRR.

# 3. Local analyses

A detailed analysis plan will be written by WP5 team with support from statisticians from QMUL (month 27). Ulster University and QMUL will produce common syntax scripts, which will conform to a common data model to ensure that all variables/proxy variables are standardised across all registries (month 30). Registries will run the provided registry-specific syntax scripts on their linked dataset to generate the tables/results outlined in the analysis plan. A data "dictionary" of every variable in the linked data with its name, description/definition, coding instructions/values (in English) will be created and uploaded to the website. The quality of the data linkage will be investigated and data quality checks will be conducted for unlikely results and outliers across registries (Ulster University) and a report will be produced.

Analyses will include all cases with congenital anomalies (EUROCAT subgroup al1: all anomalies) and on all relevant EUROCAT subgroups specified in this protocol (Appendix 2) and provided on the EUROlinkCAT website for members

[(https://www.eurolinkcat.eu/wp2-buildingresultsrepository/wp3andwp4subgroups)](https://www.eurolinkcat.eu/wp2-buildingresultsrepository/wp3andwp4subgroups). Cases will be classified as isolated or genetic according to the multiple anomaly algorithm programmed in EDMP (Garne 2011)^23^ and analyses will be performed in groups of cases of all structural anomalies, isolated anomalies and chromosomal/genetic anomalies. The analyses will also be performed in two separate groups – cases with and without cerebral anomalies. For rare congenital anomalies with limited or no published evidence in the literature, educational outcomes will be analysed if there is a sufficient number of cases for each anomaly in the registries to be safely transferred to the CRR in aggregate tables; for those with small numbers, analytical results [e.g. means with the standard errors (SE)] will be provided. Power calculations will be performed by QMUL to ensure that there is sufficient statistical power for the selected anomaly subgroups to derive meaningful conclusions.

Education data will be analysed in all long-term survivors from the participating registries who reached the school age (varies in different registries)*.* WP3 mortality data will be used to identify survivors up to 10 years of age and WP4 morbidity data will be used to analyse the association between clinical factors (e.g. number of days spent in hospital and in intensive care units, surgery) and educational outcomes. We will not have mortality or morbidity data for children beyond 10 years of age for the WP5 participants, but given that the mortality rates at this age are extremely low, this will only marginally affect our results. For the five English registries and the Welsh registry, the education outcome data for children in a compulsory school will include exam results for different subjects (Reading, Writing, Maths) at age 6-7 and 10-11 years, and data on special education needs (SEN) will be available for the group of children who need SEN support. For Denmark, Finland and Norway registries, less detailed data will be available, probably limited to the SEN information (yes/no) and to the data indicating graduation from compulsory school with or without grades. Deaths between 10 and 16 years will be known to these registries, as their congenital anomaly data are already linked to the population data in their health statistics databases.

The first study will determine the educational achievements and special education needs of children born with a congenital anomaly in different European regions up to age 16 years, we will also describe educational outcomes for individual subgroups of congenital anomalies including those with multiple anomalies. Data on terminations of pregnancy for fetal anomaly (TOPFA) already available in the EUROCAT registries will be used for facilitating the interpretation of the survival analysis and the number of children with special education need in each cohort.

The second study will evaluate the association between clinical (the use of anaesthesia, surgery, days spent in hospital) and sociodemographic factors (infant sex, maternal age, socioeconomic status) and educational achievements and needs by using multilevel models to allow for differences between types of congenital anomalies These analyses will be run separately by each registry and the results submitted to the CRR using a secure data transfer procedure and data suppression if required.

# 4. Data transmission to the CRR and to WP5

The tables and results created by each registry using the supplied syntax scripts will be submitted in Excel, SPSS or STATA file formats, or other commercially available packages, to the Ulster University via the secure project portal (members’ area on the EUROlinkCAT website) (month 33). All data submitted will be aggregated - no individual case data will be sent.

Ulster University will then:

1. Compile the data from each participating registry to create the CRR;
2. Perform data quality checks together with QMUL;
3. Generate extracts of the data from the CRR required for this WP5 study;
4. Provide clean, checked aggregate data and analytical results from the CRR to the WP5 project leaders via the secure project portal (members’ area on the EUROlinkCAT website) (month 36).

Only combined data and the summarised results will be sent to the WP5 research team. The WP5 team will perform pooled analyses based on the data received from the CRR with the statistical advice from QMUL/UU.

# WP5 analyses and milestones

UNEW:

The UNEW team will perform meta-analyses on education outcomes (educational achievements and needs) based on the data from eleven registries received from the CRR. Results will be discussed at a subgroup meeting with the involved registries (month 38) and a first draft of the paper/report will be circulated for comments (month 40). The paper will be submitted before the final report to the EU (month 42).

We will also perform meta-analyses of the results of the association between clinical and/or sociodemographic factors and educational achievements and needs. Results will be discussed at a subgroup meeting with the involved registries (month 38) and a first draft of the paper will be circulated for comments in month 48 and submitted for publication (month 50).

The modelling predicting the number of children with congenital anomalies under 16 years across Europe who will have specific educational needs will be done at UNEW as well. Results will be discussed at a subgroup meeting with the involved registries (month 38) and a first draft of the paper will be circulated for comments in month 46. The paper will be submitted before the final report to the EU (month 50).

# 5. Publication of results

All three studies will be published in high-impact peer-review journals with open access and with authorship according to EUROlinkCAT criteria.

The studies have two deliverables in the Horizon 2020 contract:

D5.1: Report on educational achievements and needs of children born with a congenital anomaly and geographical variation in Europe [month 42]

D5.2: Report on predictions of the number of children with congenital anomalies across Europe up to the child’s 10^th^ birthday who will have specific educational needs [month 50]

# 6. Data archiving and destruction

a. All results generated from EUROlinkCAT will be archived at the Ulster University. These will include the pooled analyses from each WP.
b. Destruction of CRR data will occur 20 years after the completion of the EUROlinkCAT project, at which point it is believed such data will no longer be of use.
c. Each WP leader/institution is responsible for ensuring the destruction of any data five years after the completion of the EUROlinkCAT project.
d. Any duplicate datasets held at QMUL will be destroyed five years after the completion of the EUROlinkCAT project.

# References

1. Rankin J, Tennant PW, Bythell M, Pearce MS. Predictors of survival in children born with Down syndrome: a registry-based study. *Pediatrics* 2012;129:e1373-1381.
2. Erikssen G, Liestol K, Seem E, Birkeland S, Saatvedt KJ, Hoel TN, et al. Achievements in congenital heart defect surgery: a prospective, 40-year study of 7038 patients. *Circulation* 2015;131:337-346; discussion 346.
3. Cassina M, Ruol M, Pertile R, Midrio P, Piffer S, Vicenzi V, et al. Prevalence, characteristics, and survival of children with esophageal atresia: A 32-year population-based study including 1,417,724 consecutive newborns. *Birth Defects Res A Clin Mol Teratol* 2016;106:542-548.
4. Shin M, Kucik JE, Siffel C, Lu C, Shaw GM, Canfield MA, et al. Improved survival among children with spina bifida in the United States. *J Pediatr* 2012;161:1132-1137.
5. Mulkey SB, Bai S, Luo C, Cleavenger JE, Gibson N, Holland G, et al. School-Age Test

Proficiency and Special Education After Congenital Heart Disease Surgery in Infancy. *J Pediatr* 2016;178:47-54 e41.

6. Mulkey SB, Swearingen CJ, Melguizo MS, Reeves RN, Rowell JA, Gibson N, et al. Academic proficiency in children after early congenital heart disease surgery. *Pediatr Cardiol* 2014;35:344-352.
7. Shillingford AJ, Glanzman MM, Ittenbach RF, Clancy RR, Gaynor JW, Wernovsky G. Inattention, hyperactivity, and school performance in a population of school-age children with complex congenital heart disease. *Pediatrics* 2008;121:e759-767.
8. Oster ME, Watkins S, Hill KD, Knight JH, Meyer RE. Academic Outcomes in Children With Congenital Heart Defects: A Population-Based Cohort Study. *Circ Cardiovasc Qual Outcomes* 2017;10.
9. Gerstle M, Beebe DW, Drotar D, Cassedy A, Marino BS. Executive Functioning and School Performance among Pediatric Survivors of Complex Congenital Heart Disease. *J Pediatr* 2016;173:154-159.
10. Cassidy AR, White MT, DeMaso DR, Newburger JW, Bellinger DC. Processing speed, executive function, and academic achievement in children with dextro-transposition of the great arteries: Testing a longitudinal developmental cascade model. *Neuropsychology* 2016;30:874-885.
11. Wernovsky G, Stiles KM, Gauvreau K, Gentles TL, duPlessis AJ, Bellinger DC, et al. Cognitive development after the Fontan operation. *Circulation* 2000;102:883-889.
12. Persson M, Becker M, Svensson H. Academic achievement in individuals with cleft: a population-based register study. *Cleft Palate Craniofac J* 2012;49:153-159.
13. Wehby GL, Collett BR, Barron S, Romitti P, Ansley T. Children with oral clefts are at greater risk for persistent low achievement in school than classmates. *Arch Dis Child* 2015;100:11481154.
14. Fitzsimons KJ, Copley LP, Setakis E, Charman SC, Deacon SA, Dearden L, et al. Early academic achievement in children with isolated clefts: a population-based study in England. *Arch Dis Child* 2017.
15. Clausen NG, Pedersen DA, Pedersen JK, Moller SE, Grosen D, Wehby GL, et al. Oral Clefts and Academic Performance in Adolescence: The Impact of Anesthesia-Related Neurotoxicity, Timing of Surgery, and Type of Oral Clefts. *Cleft Palate Craniofac J* 2017;54:371-380.
16. De Graaf G, Van Hove G, Haveman M. A quantitative assessment of educational integration of students with Down syndrome in the Netherlands. *J Intellect Disabil Res* 2014;58:625-636.
17. van Wouwe JP, van Gameren-Oosterom HB, Verkerk PH, van Dommelen P, Fekkes M. Mainstream and special school attendance among a Dutch cohort of children with Down Syndrome. *PloS one* 2014;9:e91737.
18. Hughes J. Inclusive education for individuals with Down syndrome. *Down Syndrome News and Update* 2006;6:1-3.
19. Buckley S, Bird G, Sacks B, Archer T. A comparison of mainstream and special education for teenagers with Down syndrome: implications for parents and teachers. *Downs Syndr Res Pract* 2006;9:54-67.
20. de Graaf G, van Hove G, Haveman M. More academics in regular schools? The effect of regular versus special school placement on academic skills in Dutch primary school students with Down syndrome. *J Intellect Disabil Res* 2013;57:21-38.
21. Laws G, Burne A, Buckley S. Language and Memory Development in Children with Down Syndrome at Mainstream Schools and Special Schools: a comparison. *Educational Psychology* 2000;20:447-457.
22. Iyer NP, Tucker DF, Roberts SH, Moselhi M, Morgan M, Matthes JW. Outcome of fetuses with Turner syndrome: a 10-year congenital anomaly register based study. *J Matern Fetal Neonatal Med* 2012;25:68-73.
23. Garne E, Dolk H, Loane M, Wellesley D, Barisic I, Calzolari E, et al. Paper 5: Surveillance of multiple congenital anomalies: implementation of a computer algorithm in European registers for classification of cases. *Birth Defects Res A Clin Mol Teratol* 2011;91 Suppl 1:S4450.

# Table 1 Variables to extract from the EUROCAT database (EDMP)

|  | **EUROCAT core variables, one row of data per case (see also** [**http://www.eurocat-**](http://www.eurocat-network.eu/aboutus/datacollection/guidelinesforregistration/guide1_4)  [**network.eu/aboutus/datacollection/guidelinesforregistration/guide1_4)**](http://www.eurocat-network.eu/aboutus/datacollection/guidelinesforregistration/guide1_4) | |
| --- | --- | --- |
|  | **EDMP Core variables (shaded in blue)** | |
| **Baby and Mother – Variables 1 to 18** | | |
| 1 | CENTRE | Centre Number |
| 2 | NUMLOC | Local ID of case |
| 3 | BIRTH_DATE | Date of Birth |
| 4 | SEX | Sex |
| 5** | NBRBABY | Number of babies delivered |
| 6 | SP_TWIN | Specify twin type of birth, like or unlike, zygosity |
| 7 | NBRMALF | Number of malformed in multiple set |
| 8 | TYPE | Type of birth |
| 9 | CIVREG | Civil registration status |
| 10 | WEIGHT | Birth weight |
| 11 | GESTLENGTH | Length of gestation in completed weeks |
| 12 | SURVIVAL | Survival beyond one week of age |
| 13 | DEATH_DATE | Date of death |
| 14 | DATEMO | Date of birth of mother |
| 15 | AGEMO | Age of mother at delivery |
| 16* | BMI | Maternal Body Mass Index |
| 17 | RESIDMO | Mother’s residence code |
| **Diagnosis – Variables 19 to 57** | | |
| 19** | WHENDISC | When discovered |
| 20 | CONDISC | Condition at discovery |
| 21 | AGEDISC | If prenatally diagnosed, gestational age at discovery |
| 22 | FIRST PRE | First positive prenatal test |
| 24 | KARYO | Karyotype of infant/fetus |
| 25 | SP_KARYO | Specify karyotype |
| 26* | GENTEST | Genetic Test |
| 27* | SP_GENTEST | Specify genetic test |
| 28 | PM | Post mortem examination |
| 29** | SURGERY | First surgery for malformation performed or planned |
| 30 | SYNDROME | Syndrome |
| 31 | SP_SYNDROME | Specify Syndrome |
| 32 | MALFO1 | malformation |
| 33 | SP_MALFO1 | Specify malformation |
| 34 | MALFO2 | As MALFO1 |
| 35 | SP_MALFO2 | Specify malformation |
| 36 | MALFO3 | As MALFO1 |
| 37 | SP_MALFO3 | Specify malformation |
| 38 | MALFO4 | As MALFO1 |
| 39 | SP_MALFO4 | Specify malformation |
| 40 | MALFO5 | As MALFO1 |
| 41 | SP_MALFO5 | Specify malformation |
| 42 | MALFO6 | As MALFO1 |
| 43 | SP_MALFO6 | Specify malformation |
| 44 | MALFO7 | As MALFO1 |
| 45 | SP_MALFO7 | Specify malformation |
| 46 | MALFO8 | As MALFO1 |
| 47 | SP_MALFO8 | Specify malformation |
| 57# | OMIM | OMIM code / Type of Mendelian Inheritance |
| **Exposure – variables 58 to 78** | | |
| 58** | ASSCONCEPT | Assisted conception (where available) |
| 59## | OCCUPMO | Mother’s occupation at time of conception |
| 60 | ILLBEF1 | Maternal illness before pregnancy 1 |
| 61 | ILLBEF2 | Maternal illness before pregnancy 2 |
| 64 | ILLDUR1 | Maternal illness during pregnancy 1 |
| 65 | ILLDUR2 | Maternal illness during pregnancy 2 |
| 79 | CONSANG | Consanguinity |
| 81 | SIBANOM | Sibs with congenital anomalies |
| 87 | MOANOM | Mothers family with anomalies |
| 89 | FAANOM | Fathers family with anomalies |
| **Sociodemographic – Variables 91 to 94** | | |
| 91 | MATEDU | Maternal education |
| 92 | SOCM | Socioeconomic status of mother |
| 93 | SOCF | Socioeconomic status of father |
| 94 | MIGRANT | Migrant status |
| **EDMP-derived variables** | |  |
|  | Byear | Year of birth |
|  | birth_type | Definitions of stillbirths and spontaneous abortions vary between regions. This variable recodes birth type according to EUROCAT’s specifications: cases with gestational age ≥ 20 weeks are re-coded as “stillbirths” (irrespective of the local definition of stillbirth/spontaneous abortion). |
|  | casestatus | Only cases with casestatus = 1 |
|  | al1-al114 | EUROCAT subgroups: (0 = No, 1 = Yes). Based on EUROCAT coding in Guide 1.4 |
|  | mult_malf | Algorithm for case classification into isolated and multiples |

*	New variable In Guide 1.4 from 2013

**	Variable compatible over time, but coding has been extended/modified

\#	Variable name change only

\##	Guide 1.4 use ISCO-08 classifications

# Table 2 Education outcome variables to be linked to the congenital anomaly cases listed separately for each participating registry

| **Variables in education databases by registry to be linked to congenital anomaly cases** | | | |
| --- | --- | --- | --- |
| BINOCAR – National Pupil database (NPD) –examples covering different types of outcomes and fields are given (see [http://www.EUROlinkCAT.eu/wp2-buildingresultsrepository/variables](http://www.eurolinkcat.eu/wp2-buildingresultsrepository/variables) for the full list) | | | |
| **Variable name** | **Description** | **Years populated** | **Table name in NPD** |
| AcademicYear_[term][yy] | Academic year | 2001/02 - | PLASC_Census 01-02 to 16-17 SPR |
| SENprovision_[term][yy] | Provision types under the special educational need (SEN) Code of Practice. | 2001/02 - | PLASC_Census 01-02 to 16-17 SPR |
| SENprovisionMajor_[term  ][yy] | Pupil's major SEN provision group based on SEN provision code. | 2008/09- | PLASC_Census 01-02 to 16-17 SPR |
| PrimarySENtype_[term][y y] | Nature of pupil's primary special educational need. | 2003/04 - | PLASC_Census 01-02 to 16-17 SPR |
| SecondarySENtype_[term  ][yy] | Nature of pupil's secondary special educational need | 2003/04 - | PLASC_Census 01-02 to 16-17 SPR |
| SENUnitIndicator_[term][ yy] | Indicates if a pupil with SEN in a mainstream school is a member of a SEN Unit (sometimes called special class) | 2006/07- | PLASC_Census 01-02 to 16-17 SPR |
| ResourcedProvisionIndica tor_[term][yy] | Indicates if a pupil with SEN in a mainstream school is a member of a resourced provision | 2006/07- | PLASC_Census 01-02 to 16-17 SPR |
| AcademicYear_ab[yy] | Academic year – pupil absence | 2005/06 - | Absence_05-  06_to_15-16 |
| AP_PupilMatchingRefAno nymous | Pupil matching reference -  Anonymous | 2007/2008 - | Alt_Provision_0708_to_16-17 |
| AP_UPN | Unique Pupil Number | 2007/2008 - | Alt_Provision_0708_to_16-17 |
| AuthorisedAbsence_2Ter m_ab[yy] | Number of sessions missed due to authorised absence in Autumn and Spring terms. This will be blank for special schools. | 2005/06 - | Absence_05-  06_to_15-16 |
| CIN_PrimaryNeedCode | The main need which the episode is taking care of. | 2008/2009 - | Children in Need -  CIN_08-09_to_15-16 |
| CIN_PUPILID | Pupil matching reference. | 2008/2009 - | CIN_08-09_to_15-16 |
| CIN_PupilMatchingRefAn onymous | Pupil matching reference - Anonymous. | 2008/2009 - | CIN_08-09_to_15-16 |
| CIN_PupilMatchingRefNo nAnonymous | Pupil matching reference - Non Anonymous. | 2008/2009 - | CIN_08-09_to_15-16 |
| CIN_DOB | Date Of Birth | 2008/2009 - | CIN_08-09_to_15-16 |

| CIN_Ethnicity | Code identifying the child's ethnic group | 2009/2010 - | CIN_08-09_to_15-16 |
| --- | --- | --- | --- |
| CIN_Disability | Holds a record of the type of disability(s) a child may suffer from. NONE by itself is used for no disability. | 2008/2009 - | CIN_08-09_to_15-16 |
| cla_CHILD_ID | DfE unique child identifier | 2005/2006 - | CLA_05-06_to_15-16 |
| cla_CHILD_LA_CODE | Local Authority child identifier | 2005/2006 - | CLA_05-06_to_15-16 |
| cla_DOB | Date of birth | 2005/2006 - | CLA_05-06_to_15-16 |
| cla_ETHNIC | Ethnic origin. This corresponds to EthnicGroupMinor in Census data. | 2005/2006 - | CLA_05-06_to_15-16 |
| cla_SEX | Gender | 2005/2006 - | CLA_05-06_to_15-16 |
| KS1_APS | Average attainment point score (including Reading, Writing, Maths and Overall Science only). | 2006/07-  2014/15 | KS1_97-98_to_15-16 |
| KS1_APSRWM | Average attainment point score (including Reading, Writing and Maths only). | 2010/11 -  2014/15 | KS1_97-98_to_15-16 |
| KS1_DOB | Date of birth. | 1997/98 - | KS1_97-98_to_15-16 |
| KS1_ENGCOMPTST | English Comprehension Test. | 1997/98 -  2003/04 | KS1_97-98_to_15-16 |
| KS1_ENGLEV | English Average Level  (derived from Reading and  Writing) | 2007/08 only | KS1_97-98_to_15-16 |
| KS1_ENGREADTSK | English Reading Task. | 1997/98 -  2003/04 | KS1_97-98_to_15-16 |
| KS1_ENGSPELLTST | English Spelling Test. | 1997/98 -  2001/02 | KS1_97-98_to_15-16 |
| KS1_ENGSUBTA | Overall English Teacher Assessment Level. | 1997/98 -  2003/04 | KS1_97-98_to_15-16 |
| KS1_ENGWRITTST | English Writing Test. | 1997/98 -  2003/04 | KS1_97-98_to_15-16 |
| KS1_ERELIG | Eligible result for Reading | 2003/04 only | KS1_97-98_to_15-16 |
| KS1_EWELIG | Eligible result for Writing. | 2003/04 only | KS1_97-98_to_15-16 |
| KS1_MAELIG | Eligible result for Maths. | 2003/04 only | KS1_97-98_to_15-16 |
| KS1_MATH_OUTCOME | Mathematics outcome | 2015/16 - | KS1_97-98_to_15-16 |
| KS1_MATHNOALG | Maths Number and  Algebra. (Teacher Assessment) | 1997/98 -  2003/04 | KS1_97-98_to_15-16 |
| KS1_MATHS | National Curriculum level awarded for Maths. (Teacher Assessment) | 1997/98 -  2014/15 | KS1_97-98_to_15-16 |
| KS1_READING | National Curriculum level awarded for reading. (Teacher Assessment) | 1997/98 -  2014/15 | KS1_97-98_to_15-16 |

| KS1_WRITING | National Curriculum level awarded for writing. (Teacher Assessment) | 1997/98 -  2014/15 | KS1_97-98_to_15-16 |
| --- | --- | --- | --- |
| KS2_MATLEV | National Curriculum level awarded for Maths test. | 1995/96 -  2014/15 | KS2_95-96_to_15-16 |
| KS2_MATMAINLEV | Maths Main Test Level | 1995/96 -  2004/05 | KS2_95-96_to_15-16 |
| KS2_MATLEVTA | National Curriculum level awarded for Maths  Teacher Assessment. | 2009/10 -  2014/15 | KS2_95-96_to_15-16 |
| KS2_READLEV | National Curriculum level awarded for English reading test. | 1998/99 -  2014/15 | KS2_95-96_to_15-16 |
| KS2_WRITLEVTA | National Curriculum level awarded for English writing Teacher Assessment. | 2011/12 -  2014/15 | KS2_95-96_to_15-16 |
| KS2_WRITLEV | Writing level | 2006/07 -  2014/15 | KS2_95-96_to_15-16 |
| KS2_WRITMARK | Writing test mark. | 2006/07 -  2014/15 | KS2_95-96_to_15-16 |
| Denmark: Odense | |  |  |
| **Variable name** | **Description** | **Years populated** | **Table name** |
| ALM_VFRA | Date for primary education | 1981- | Statistics Denmark |
| ALMAUDD | Primary education | 1981- | Statistics Denmark |
| ERH_VFRA | Date for vocational education | 1981- | Statistics Denmark |
| ERHAUDD | Vocational education | 1981- | Statistics Denmark |
| Date for Highest attained education | Date for Highest attained education | 1981- | Statistics Denmark |
| HFAUD | Highest attained education | 1981- | Statistics Denmark |
| IG_VFRA | Start date for ongoing education | 1981- | Statistics Denmark |
| IGUDD | Ongoing education | 1981- | Statistics Denmark |
| PNR | Personal Identifier | 1981- | Statistics Denmark |
| DANSK_2_SP | Teaching in Danish as a second language | 2011- | UDSP |
| KLASSETRIN | Code indicating class level, i.e. 0 = 0. grade, 1 = 1st grade, 10 = 10th grade, 11 = 11th grade, 2 = 2nd grade, 3 = 3rd grade, 4 = 4th grade, 5 = 5th grade, 6 = 6th grade, 7 = 7th grade,  8 = 8th grade, 9 = 9th grade | 2011- | UDSP |
| KL_TYPE | Class type: 40=Normal class, full-time divided, 41=Normal class, not fully graded, 50=special class, 55=Class for older bilingual students, 99=undisclosed | 2011- | UDSP |

| SKL_VFRA | Start time of education (UDD). | 2011- | UDSP |
| --- | --- | --- | --- |
| SPC_ART | Type of special needs education (H00= not SEN; H1-H20 – reason for referral to special education; H99=undisclosed) | 2011- | UDSP |
| SPC_OMFANG | Extent of SEN (specifies the average number of special tuition hours per week for each SEN program) | 2011- | UDSP |
| SPC_SLUT | End of special education | 2012- | UDSP |
| SPC_START | Start of special education | 2012- | UDSP |
| UDD | Code of education  (included over 3500 codes) | 2011- | UDSP |
| UDEL | Part of educational program | 2011- | UDSP |
| Italy: Tuscany | |  |  |
| **Variable name** | **Description** | **Years populated** | **Table name** |
| achievements of educational objectives | achievements of educational objectives |  |  |
| attending school class | attending school class |  |  |
| certification date | certification date (if available) |  | Regional Scholastic Office |
| early school leaving | early school leaving |  |  |
| educational support period | educational support period |  |  |
| hours of educational support | hours of educational support |  |  |
| personalised educational plan | personalised educational plan |  |  |
| possible temporary school interruption | possible temporary school interruption |  |  |
| severity of disability | severity of disability |  |  |
| type of support | type of support |  |  |
| Wales – examples covering different educational outcomes are given here, while all the variables are listed on [http://www.EUROlinkCAT.eu/wp2-buildingresultsrepository/variables,](http://www.eurolinkcat.eu/wp2-buildingresultsrepository/variables) | | | |
| **Variable name** | **Description** | **Years populated** | **Table name** |
| CATEGORYOFPROVISION | Categories of provision | 2010-2016 | EOTAS_PROVISION |
| BOARDER | Whether the pupil is a boarder | 2009-2016 | PUPIL |
| CSI_TA | Core Subject Indicator– Teaching Assessment | 2009-2016 | NDC_PUPIL |
| CYM_TA | Welsh First Language – Teaching Assessment | 2009-2016 | NDC_PUPIL |

| The progress of pupil gaining English as an Additional Language | The progress of pupil gaining English as an Additional Language | 2009-2016 | PUPIL |
| --- | --- | --- | --- |
| ENG_TA | English First Language – Teaching Assessment | 2009-2016 | NDC_PUPIL |
| ENROLSTATUS | Enrolment Status of the pupil | 2009-2016 | EOTAS_PUPIL |
| ENROLSTATUS | The pupil's enrolment status | 2009-2016 | PUPIL |
| ENTRYDATE | The pupil's entry date to the current school | 2009-2016 | PUPIL |
| ESTAB | Establishment Reference Number | 2009-2016 | PLASC_SEN |
| ESTAB | Establishment Reference Number | 2009-2016 | ATTENDANCE |
| ETHNICITY | The pupil's ethnicity origin | 2009-2016 | PUPIL |
| ETHNICITYSOURCE | The source of provider on pupil's ethnicity origin | 2009-2016 | PUPIL |
| FSMELIGIBLE | The pupil's eligibility for Free School Meal Scheme | 2009-2016 | PUPIL |
| KEYSTAGE | The level of Key Stage | 2009-2016 | NDC_PUPIL |
| LEA | Local Education Authority code | 2009-2016 | ATTENDANCE |
| MAT_TA | Mathematics subject – Teaching Assessment | 2009-2016 | NDC_PUPIL |
| NATIONALIDENTITY | The pupil's national identity | 2009-2016 | PUPIL |
| NCYEARACTUAL | The year group in which the pupil is taught for the majority of their time, regardless of their age. | 2009-2016 | PUPIL |
| NCYEARACTUAL | The National Curriculum Year Group (Year taught in) is the year group in which the pupil for the majority of time regardless of their chronological age. | 2009-2016 | EOTAS_PUPIL |
| PARTTIME | Indicator whether pupil attends school on a parttime basis | 2009-2016 | PUPIL |
| SAIL_ID_E | Anonymised Unique Pupil  Identifier | 2010-2016 | EOTAS_PROVISION |
| SAIL_ID_E | Anonymised Unique Pupil  Identifier | 2009-2016 | EOTAS_PUPIL |
| SAIL_ID_E | Anonymised Unique Pupil  Identifier | 2009-2016 | PLASC_SEN |
| SAIL_ID_E | Anonymised Unique Pupil  Identifier | 2009-2016 | ATTENDANCE |
| SAIL_ID_E | Anonymised Unique Pupil  Identifier | 2009-2016 | PUPIL |
| SAIL_ID_E | Anonymised Unique Pupil  Identifier | 2009-2016 | NDC_PUPIL |
| SAIL_ID_E | Anonymised Unique Pupil  Identifier | 2009-2016 | EOTAS_SEN |
| SCI_TA | Science subject – Teaching Assessment | 2009-2016 | NDC_PUPIL |
| SENADVICEANDASSESSM  ENT | The level of SEN advice and assessment | 2009-2016 | PUPIL |
| SENPROVISION | The pupil's provision for SEN | 2009-2016 | EOTAS_PUPIL |
| SENPROVISION | The levels of Special  Education Needs Provision | 2009-2016 | PUPIL |
| SENSPECIALISEDRESOURC  ES | The level of SEN specialised resource | 2009-2016 | PUPIL |
| SENTYPE | Types of SEN | 2009-2016 | PLASC_SEN |
| SENTYPE | Types of SEN | 2010-2016 | EOTAS_SEN |
| SENTYPERANK | Ranking order by SEN type | 2009-2016 | PLASC_SEN |
| SESSIONSOVERALL | This field records how many sessions a pupil missed in a school year | 2009-2016 | ATTENDANCE |
| SESSIONSAUTHORISED | This field records how many of the pupil’s absence were “Authorised”. | 2009-2016 | ATTENDANCE |
| SESSIONSPOSSIBLE | The overall sessions possible in a school year. A session is half a day, | 2009-2016 | ATTENDANCE |
| SESSIONSUNAUTHORISED | This field records how many of the pupil’s absence were  “Unauthorised”. | 2009-2016 | ATTENDANCE |
| SPEAKWELSH | The pupil's Welsh capacity | 2009-2016 | PUPIL |
| WEL | Welsh Second Language | 2009-2016 | NDC_PUPIL |
| YEAR | Academic year | 2009-2016 | All tables |

# Table 3 Variables from the WP3 and WP4 morbidity databases (core variables shaded grey)

| **Variable number** | **Variable Name** | **Variable Definition and Instructions** | **Variable Values/ format** | **Variable released to researcher** |
| --- | --- | --- | --- | --- |
| 1 | L_CH_ID | Local ID number used to identify child | Unique identifier | No |
| 2 | L_CH_DATE_B | Child’s date of birth  Year of birth must be known |  | No |
| 3 | L_CH_YEAR_B | Child’s year of birth | Four digits required  e.g. 2005 | Yes |
| 4 | L_CH_SEX | Infant’s sex | As recorded in the vital statistics or health care database | Yes |
| 5 | L_DATE_LOST | Date lost to follow-up/ linkage (i.e. due to emigration, adoption or other reason) |  | No |
| 6 | L_YEAR_LOST | Year child lost to follow-up/ linkage (i.e. due to emigration, adoption or other reason) | Four digits required  e.g. 2005 | Yes |
| 7 | L_AGEL_D | Age at lost to follow-up in complete days | Numeric | Yes |
| 8 | L_MORT_MATCH | Match with vital statistics database (or, if not available, hospital episodes data) | 1= Matched  2= Not matched | Yes |
| 9 | L_CONFIDENCE | Strength of match with hospital episodes data | Please use your local data provider’s codes.    If no local code available, a guideline on how to assess confidence in matching is found below* | Yes |
| 10 | L_MAT_CTRY_B | Maternal country of birth/ place of birth/ country of origin | As recorded in the vital statistics or health care database | Yes |
| 11 | L_MAT_DOB | Maternal date of birth |  | No |
| 12 | L_MAT_YEAR_B | Maternal year of birth | Four digits required  e.g. 1980 | Yes |
| 13 | L_MATAGE_B | Maternal age at infant's birth  Calculate maternal age (completed years) at infant’s date of birth | Numeric | Yes |
| 14 | L_MAT_EDUC | Maternal education at infant’s birth | As recorded in the vital statistics or health care database | Yes |

| 15 | L_MAT_EMPL | Mother’s employment status at infant’s birth | As recorded in the vital statistics or health care database | Yes |
| --- | --- | --- | --- | --- |
| 16 | L_MAT_OCC | Maternal occupation at infant’s birth | As recorded in the vital statistics or health care database | Yes |
| 17 | L_MATDEPR_IND | Quintile of Deprivation index of maternal residence at infant’s birth | As recorded in the vital statistics or health care database | Yes |
| 18 | L_MATMAR_STA | Maternal marital status at infant’s birth | As recorded in the vital statistics or health care database | Yes |
| 19 | L_CH_DATE_D | Child’s date of death on the death  certificate or in the mortality database |  | No |
| 20 | L_CH_YEAR_D | Child’s year of death | Four digits required  e.g. 2006 | Yes |
| 21 | L_CH_AGED_H | Age at death in complete hours for day 0 (first 24 hours) = applies to infants who died within the first 24 hours | 0 = Died <1 hour after birth 1 = Died 1 complete hour after birth 2 = Died 2 complete hours after birth   Etc  23 = Died 23 complete hours after birth  88 = Alive at 24 hours  99 = Died within first 24 hours, but exact time unknown | Yes |
| 22 | L_CH_AGED_D | Age at death in complete days (up to 10^th^ birthday).  Should be provided in days for infants who died after the first 24 hours.  Subtract child’s date of birth from child’s date of death.  If age at death in complete days is not available, please complete age at death in complete months (variable L_CH_AGED_M) and/ or age at death  in complete years (variable L_CH_AGED_Y). | 0 = Died <1 complete day after birth 1 = Died 1 complete day after birth 2 = Died 2 complete days after birth  Etc    8888 = Alive on 10th birthday  9999 = Died before 10^th^ birthday, but exact date unknown | Yes |
| 23 | L_CH_AGED_M | Age at death in complete months  Only complete, if age at death in complete days (variable L_CH_AGED_D) is not available | 0 = Died <1 complete month after birth 1 = Died 1 complete months after birth 2 = Died 2 complete months after birth  Etc | No |

|  |  |  | 888 = Alive on 10th birthday  999 = Died before 10^th^ birthday, but exact date unknown |  |
| --- | --- | --- | --- | --- |
| 24 | L_CH_AGED_Y | Age at death in complete years  Only complete, if age at death in complete days (variable L_CH_AGED_D) is not available | 0 = Died <1 complete year after birth 1 = Died 1 complete year after birth 2 = Died 2 complete years after birth  Etc    88 = Alive on 10th birthday  99 = Died before 10^th^  birthday, but exact date unknown | No |
| 25 | L_CH_GA_B | Child’s gestational age at birth (in completed weeks) | As recorded in the vital statistics or health care database | Yes |
| 26 | L_CH_BW | Child’s birth weight (in grams) | As recorded in the vital statistics or health care database | Yes |
| 27 | L_MULT_BIRTH | Singleton or multiple birth | As recorded in the vital statistics or health care database | Yes |
| 28 | L_PARITY | Number of previous pregnancies | Numeric | Yes |
| 29 | L_DATE_ADM | Date of admission to hospital* | Date | No |
| 30 | L_YEAR_ADM | Year of admission to hospital | Four digits required  e.g. 2005 | Yes |
| 31 | L_DATE_DIS | Date of discharge from hospital | Date | No |
| 32 | L_HOSP_DAYS | Length of stay in hospital (i.e.  number of days) | Numeric | Yes |
| 33 | L_CH_AGE_ADM_D | Child’s age at hospital admission in complete days (up to 10^th^ birthday).  Subtract child’s date of birth from date of admission to hospital | Numeric | Yes |
| 34 | L_DIAG_DIS | Main diagnosis in ICD9 or ICD10 for the hospital stay | As recorded in the vital statistics or health care database | Yes |
| 35 | L_DIAG_SEC1 | Other diagnoses for the hospital stay (ICD9 or ICD10) | As recorded in the vital statistics or health care database | Yes |
| 36 | L_DIAG_SEC2 | Other diagnoses for the hospital stay (ICD9 or ICD10) | As recorded in the vital statistics or health care database | Yes |
| 37 | L_DIAG_SEC3 | Other diagnoses for the hospital stay (ICD9 or ICD10) | As recorded in the vital statistics or health care database | Yes |
| 38 | L_DIAG_SEC4 | Other diagnoses for the hospital stay (ICD9 or ICD10) | As recorded in the vital statistics or health care database | Yes |
| 39 | L_DIAG_SEC5 | Other diagnoses for the hospital stay (ICD9 or ICD10) | As recorded in the vital statistics or health care database | Yes |
| 40 | L_DAYS_ICU | Number of days in intensive care unit during hospital stay  (Include Neonatal Intensive Care  Unit, Paediatric Intensive Care  Unit, or Intensive Care Unit) | Numeric | Yes |
| 41 | L_DAYS_VENT | Number of days on ventilator during hospital stay | Numeric | Yes |
| 42 | L_SURG_CODE1 | Codes for surgery performed during hospital stay | As recorded in the vital statistics or health care database | Yes |
| 43 | L_SURG_CODE2 | Codes for surgery performed during hospital stay | As recorded in the vital statistics or health care database | Yes |

*If there are multiple admissions, please provide a record for each hospital admission. The number of admissions per year and total days spent in hospital per year can be calculated from

L_CH_AGE_ADM_D and L_HOSP_DAYS (or the difference between the date of discharge - date of admission)

# Table 4 Type of controls planned for the WP5 participating registries

| **Registry name** | **Type of controls** |
| --- | --- |
| Denmark: Odense | Population |
| England (5 registries) | Controls |
| Italy: Emilia Romagna | Population |
| Italy :Tuscany | Population |
| UK: Wales | Population |
| Finland | Population |
| Norway | Population |

# Appendix 1

## Sources of Education data for Linkage (as reported by the EUROCAT registries in the WP5 survey)

- England:	five	BINOCAR	registries:	the	National	Pupil	Database	(NPD)	-

[https://www.gov.uk/government/publications/national-pupil-database-application-formdeclaration-and-agreement](https://www.gov.uk/government/publications/national-pupil-database-application-form-declaration-and-agreement)

- Denmark: Odense: Ministry of education, Statistics Denmark

Address: Frederiksholms kanal 21, 1220 Copenhagen K

Tel.: (0045) 33 92 50 00 [http://www.uvm.dk/statistik/grundskolen/karakterer-og-test/nationale-test/nationalpraestationsprofil](http://www.uvm.dk/statistik/grundskolen/karakterer-og-test/nationale-test/national-praestationsprofil)

Some data are available from the database UDSP (special need for teaching) – link with the list of the variables from 2011 below:

<http://dst.dk/extranet/ForskningVariabellister/UDSP%20-%20Specialundervisning.html>

- Emilia Romagna: the Ferrara Territorial Schools Office responsible for services for disabled children *(Referente per la formazione, Ufficio VI - Ambito Territoriale di Ferrara)*
- Finland: Statistics Finland: Register on Education - <http://www.tilastokeskus.fi/til/vkour/index_en.html>
- Tuscany: Ministry of Education, University and Research (MIUR), the Regional Scolastic Office
- Wales:	the	Secure	Anonymised	Information	Linkage	(SAIL)	database <https://saildatabank.com/>
- Norway:	Statistisk	sentralbyrå	(Statistics	Norway	-	Education)	https://www.ssb.no/en/utdanning

# Appendix 2

## Subgroups for the WP5 EUROlinkCAT studies (based on EUROCAT Subgroups of Congenital Anomalies (August 2016) with exclusions mentioned in doc 3.2 and doc 3.3 in Guide 1.4)

| **EUROCAT Subgroups** | **ICD10-BPA** | **ICD9-BPA** | **Comments** | **Subgroup binary variable number (al)** |
| --- | --- | --- | --- | --- |
| **All anomalies *** | Q-chapter,  D215, D821,  D1810^^^, P350,  P351, P371 | 74, 75, 27910,  2281^^^, 76076,  76280, 7710,  7711, 77121 |  | al1 |
| **Structural anomalies** |  |  |  |  |
| Spina Bifida | Q05 | 741 | Exclude if associated with anencephalus or encephalocele subgroups | al6 |
| Hydrocephalus | Q03 | 7423 | Exclude hydranencephaly 74232. Exclude association with NTD subgroup | al7 |
| Severe microcephaly | Q02 | 7421 | Exclude association with NTD subgroup | al8 |
| Congenital cataract | Q120 | 74332 |  | al13 |
| **Congenital Heart Defects** | Q20-Q26 | 745, 746, 7470-7474 | Exclude PDA with GA <37 weeks  Exclude peripheral pulmonary artery stenosis with GA <  37 weeks | al17 |
| Severe CHD | Q200, Q201,  Q203, Q204,  Q212, Q213,  Q220, Q224,  Q225, Q226,  Q230, Q232,  Q233, Q234,  Q251, Q252,  Q262 | 74500, 74510,  7452, 7453,  7456, 7461,  7462, 74600,  7463, 7465, 7466, 7467,  7471, 74720,  74742 | ICD9-BPA has no code for HRH and double outlet right ventricle | al97 |
| Transposition of great vessels | Q203 | 74510 |  | al19 |
| VSD | Q210 | 7454 |  | al21 |
| ASD | Q211 | 7455 |  | al22 |
| AVSD | Q212 | 7456 |  | al23 |
| Tetralogy of Fallot | Q213 | 7452 |  | al24 |
| Pulmonary valve stenosis | Q221 | 74601 |  | al27 |
| Aortic valve atresia/stenosis | Q230 | 7463 | ICD9-BPA has no code for atresia | al29 |
| Mitral valve anomalies | Q232, Q233 | 7465, 7466 |  | al110 |
| Hypoplastic left heart | Q234 | 7467 |  | al30 |
| Coarctation of aorta | Q251 | 7471 |  | al32 |

| PDA as **only** CHD in term infants (GA +37 weeks) | Q250 | 7470 | Livebirths only | al100 |
| --- | --- | --- | --- | --- |
| Cystic adenomatous malf of lung | Q3380 | No code |  | al36 |
| Cleft lip with or without cleft palate | Q36, Q37 | 7491, 7492 |  | al102 |
| Cleft palate | Q35 | 7490 |  | al103 |
| Oesophageal atresia with/ without trachea-oesophageal fistula | Q390-Q391 | 75030-75031 |  | al41 |
| Duodenal atresia or stenosis | Q410 | 75110 |  | al42 |
| Atresia or stenosis of other parts of small intestine | Q411-Q418 | 75111-75112 |  | al43 |
| Ano-rectal atresia and stenosis | Q420-Q423 | 75121-75124 |  | al44 |
| Diaphragmatic hernia | Q790 | 75661 |  | al48 |
| Gastroschisis | Q793 | 75671 |  | al50 |
| Omphalocele | Q792 | 75670 |  | al51 |
| Multicystic renal dysplasia | Q6140, Q6141 | 75316 |  | al54 |
| Cong hydronephrosis | Q620 | 75320 |  | al55 |
| Hypospadias | Q54 | 75260 |  | al59 |
| Limb reduction defects | Q71-Q73 | 7552-7554 |  | al62 |
| Club foot – talipes equinovarus | Q660 | 75450 |  | al66 |
| Hip dislocation and/or dyspasia | Q650-Q652, Q6580, Q6581 | 75430 |  | al67 |
| Polydactyly | Q69 | 7550 |  | al68 |
| Syndactyly | Q70 | 7551 |  | al69 |
| Craniosynostosis | Q750 | 75600 |  | al75 |
|  |  |  |  |  |
| **Chromosomal anomalies** |  |  |  |  |
| Down syndrome | Q90 | 7580 | With or without al17 and al40 | Al89 |
|  |  |  |  |  |
| **All subgroups below analysed as rare** |  |  |  |  |
| **Teratogenic syndromes** |  |  |  |  |
| Fetal alcohol syndrome | Q860 | 76076 |  | al83 |
| Valproate syndrome | Q8680 | No code |  | al84 |
| Maternal infections resulting in malformations | P350, P351, P371 | 7710, 7711,  77121 |  | al86 |
| **Chromosomal anomalies** |  |  |  |  |
| Turner syndrome | Q96 | 75860, 75861,  75862, 75869 |  | Al92 |
| Klinefelter syndrome | Q980-Q984 | 7587 |  | Al93 |
|  |  |  |  |  |
| **Rare structural anomalies with a EUROCAT subgroup** |  |  |  |  |
| Encephalocele | Q01 | 7420 | Exclude if associated with anencephalus subgroup | al5 |
| Arhinencephaly / holoprosencephaly | Q041, Q042 | 74226 |  | al9 |

| Anophthalmos / microphthalmos | Q110, Q111, Q112 | 7430, 7431 |  | al11 |
| --- | --- | --- | --- | --- |
| Anophthalmos | Q110, Q111 | 7430 |  | al12 |
| Congenital glaucoma | Q150 | 74320 |  | al14 |
| Anotia | Q160 | 74401 |  | al16 |
| Common arterial truncus | Q200 | 74500 |  | al18 |
| Double outlet right ventricle | Q201 | No code |  | al109 |
| Single ventricle | Q204 | 7453 |  | al20 |
| Triscuspid atresia and stenosis | Q224 | 7461 |  | al25 |
| Ebstein’s anomaly | Q225 | 7462 |  | al26 |
| Pulmonary valve atresia | Q220 | 74600 |  | al28 |
| Hypoplastic right heart | Q226 | No code |  | al31 |
| Aortic atresia / interrupte aortic arch | Q252 | 74720 |  | al111 |
| Total anom pulm venous return | Q262 | 74742 |  | al33 |
| Choanal atresia | Q300 | 7480 |  | al35 |
| Hirschsprung’s disease | Q431 | 75130-75133 |  | al45 |
| Atresia of bile ducts | Q442 | 75165 |  | al46 |
| Annular pancreas | Q451 | 75172 |  | al47 |
| Indeterminate sex | Q56 | 7527 |  | al60 |
| Situs inversus | Q893 | 7593 |  | al79 |
| VATER/VACTERL | Q8726 | 759895 |  | al112 |
|  |  |  |  |  |
| **New subgroups for EUROlinkCAT** |  |  |  |  |
|  |  |  |  |  |
| **Structural anomalies** |  |  |  |  |
| Anomalies of corpus callosum | Q040 | 74221 |  | aud1 |
| Megalencephaly | Q045 | No code |  | aud2 |
| Anomalies of intestinal fixation | Q433 | 7514 |  | aud3 |
| Unilateral renal agenesis | Q600 | No code |  | aud4 |
| Accessory kidney | Q630 | 75330 |  | aud5 |
| Bladder exstrophy | Q641 | 7535 |  | aud6 |
| Epispadia | Q640 | 75261 |  | aud7 |
| Posterior urethral valves | Q6420 | 75360 |  | aud8 |
| Prune Belly | Q794 | 75672 |  | aud9 |
| Arthrogryposis multiplex congenita | Q743 | 75580 |  | aud10 |
| Ectodermal dysplasia | Q824 | No code |  | aud11 |
|  |  |  |  |  |
| **Sequences** |  |  |  |  |
| Caudal regression sequence | Q8980 | No code |  | aud26 |
| Pierre-Robin sequence | Q8708 | 75603 |  | aud27 |
|  |  |  |  |  |
| **Genetic syndromes** |  |  |  |  |
| Alagille syndrome | Q4471 | No code |  | aud12 |
| Meckel-Gruber syndrome | Q6190 | No code |  | aud13 |
| Di George syndrome | D821 | 27910 |  | aud14 |
| Goldenhar syndrome | Q8704 | 75606 |  | aud15 |
| Cornelia de Lange syndrome | Q8712 | 759821 |  | aud16 |
| Noonan syndrome | Q8714 | 759896 |  | aud17 |
| Prader-Willi | Q8715 | 759872 |  | aud18 |
| Holt-Oram syndrome | Q8720 | 759842 |  | aud19 |
| Beckwith Wiedeman syndrome | Q8730 | 759874 |  | aud20 |
| Williams syndrome | Q8784 | No code |  | aud21 |
| Angelman syndrome | Q8785 | No code |  | aud22 |
|  |  |  |  |  |
| **Chromosomal anomalies** |  |  |  |  |
| Wolff-Hirschorn syndrome | Q933 | 75832 |  | aud23 |
| Cri-du chat syndrome | Q934 | 75831 |  | aud24 |
| Karytype XXX | Q970 | 75885 |  | aud25 |

**EUROlinkCAT WP5 - Educational achievements and needs of children with congenital anomalies**

**Statistical analysis plan for the contributing BINOCAR registries (EMSYCAR, CAROBB, WANDA and NorCAS) and for the Wales registry**

**WP5 leads:** Judith Rankin (UNEW), Amanda Neville (UNIFE)

**Researchers:** Svetlana Glinianaia (UNEW)

**Wales registry team:** Anna Rawlings. Sue Jordan, Daniel Thayer (PHW, SU)

**Statistical advice**: Joan Morris, Joachim Tan (SGUL)

**The aim** of this analysis plan is to guide the registries in their local analyses and to detail the methods of analysis to be applied to all outcomes in WP5.

UNEW will summarise the analytic results from the three BINOCAR registries (analysed collectively), Wales registry and Finland registry (for 16-year-olds only) into coherent summary tables, write the WP5 deliverable report (D5.1) and a paper for publication on the educational outcomes (on Key stage (KS) 2 and KS4 results in 11-year-old and 16-year-old children respectively using BINOCAR and Wales data) and special education needs (SEN) in children with selected congenital anomalies (CAs) compared with the reference group.

In WP5, each contributing registry is responsible for writing their scripts and running their analysis.

The team agreed that the Wales registry team (AR and DT) will write the analysis scripts in R for Wales. These will include descriptive data, unadjusted and adjusted inferential analyses. The adjusted analyses will be binary logistic regression with pre-specified covariates.

# 7. Inclusion / Exclusion Criteria

For Wales, all live births (i.e. singletons and multiples) reported in the national congenital anomaly registry (CARIS) from 1998 to 2014, and still alive by their 12^th^ & 17^th^ birthdays, will be included in the study and will be linked to education data in the Secure Anonymised Information Linkage (SAIL) databank, where possible. The reference population identified before the linkage is all live born children without CAs (without EUROCAT codes for a major CA) born during the same time period as the cases and in the same geographical area covered by CARIS, linkable to SAIL education data, and still alive by their 12^th^ & 17^th^ birthdays.

For the BINOCAR registries, live born children with a major CA registered in the three registries (Thames Valley, Wessex and East Midlands) and known to be alive at age 11 (for KS2) and 16 years (for KS4) (cases) will be linked to the English National Pupil Database (NPD) education data. Referents will be a random sample from the background population who are not CA cases that are recorded in the NPD.

# 8. Categorisation of Congenital Anomalies

Children with CAs from 20 subgroups of structural CAs, and from four subgroups of chromosomal anomalies, will be included in the analyses.

For structural anomalies, only those defined as an isolated anomaly (coded as N, A, R and I in 'mult_malf' EDMP derived variable: N: NTD isolated; A: isolated cardiac; R: isolated renal; I: isolated other) (see chapter 3.4 of the EUROCAT Guide 1.4^1^ and Garne et al, 2011^2^) will be included. For chromosomal anomalies (coded as C in 'mult_malf' variable), the following chromosomal syndromes will be included: Down syndrome (al89), Down syndrome with a congenital heart defect (CHD) (al89+al17), Down syndrome without CHD (al89 excluding al17) and Turner syndrome (al92) (see Table 1).

# 9. Tables 0-1 – evaluating quality of linkage between the EUROCAT registry data and education data

**Table 0. Quality of linkage between EUROCAT registries and education data by CA subgroup and year of birth –** using the number of referent children and children born with CAs and alive by age a) 12 years & b) 17 years (expected number) and those linked to education data, by CA subgroup and year of birth (starting from 1995 for English registries and from 1998 for Wales).

Linkage quality will be investigated locally by providing the expected number of children alive by school age (Table 0a) age 12 years & Table 0b) 17 years) and the number of children linked to education data by birth year (1998- 2007 for Wales and 1995-2007 for BINOCAR as the last birth year for 11-year-old children in 2017/18 school year is 2007, last birth year for 16-year olds - 2002) and CA subgroup for cases and for referent children and by calculating the percentage linked.

Data quality criteria for inclusion in the full analysis will be agreed for Wales and the BINOCAR registries as soon as the access to the BINOCAR data is obtained and the data examined.

**Table 1. Quality of linkage between EUROCAT registries and education data CA subgroup and educational stage –** using the number of referent children and children born with CAs, and assumed alive at the end of each educational stage (expected number) and those linked with education data, by CA subgroup.

Children known to have died (based on Office for National Statistics (ONS) mortality data from NHS Digital) at the end of each educational stage included (to an average of 11 years for Key Stage 2 and 16 years for Key Stage 4) will be excluded before the linkage for each Key stage.

The WP5 team have recently agreed that for the WP5 report due on 30^th^ November 2021, the results of the analysis of educational outcomes at KS2 and KS4 only, as primary outcomes, will be reported. Therefore, the tables for other Key stages have been removed from this analysis plan. The analysis for younger children (Early Years Foundation Stage Profile (EYFSP) and KS1) and KS3 can be performed as a supplementary analysis at a later stage if time allows.

# 10. Key Stage 2 (KS2) –11-year-old children

**KS2 – Table A.** Sociodemographic status of linked children (typically aged 11 years) with Key Stage 2 (KS2) data (referents and cases) by specific congenital anomaly (CA) subgroup for children with CAs.

The descriptive table for child’s sex, gestational age (<32, 32-36, 37+ weeks and missing - for referents in Wales data only), major language, deprivation index (specified below), ‘free school meal eligibility’ and school absence by CA subgroup will be prepared for subgroups where numbers permit.

**KS2 – Table B**. KS2 test levels of attainment - number and percentage of children achieving level 4 and above (expected level) for children with congenital anomalies and referents by school subject (English/Welsh), Maths and Science).

**KS2 – Table C**. KS2 results - 1) Unadjusted and 2), 3) and 4) Adjusted ORs of achieving level 4 and above (expected level) by school subject (measured by test) - for children with congenital anomalies versus referents - logistic regression results.

1) Unadjusted
2) Adjusted for Free school meal eligibility (FSME – yes/no)
3) Adjusted for deprivation index (NPD Income Deprivation Affecting Children Indices (IDACI) tertiles or SAIL WIMD quintiles and Welsh lower super output area (LSOA)

Townsend deprivation scores and ranks,^3,4^ birth year and gestational age (GA) group (birth year and GA group for Wales only)

4) Adjusted for school absence

The first analysis (1) will show if the unadjusted odds ratio of achieving level 4 and above (expected level) by school subject (English, Maths and Science) (measured by test) differ between children with a CA (by CA subgroup) and referents.

The second (2), third (3) and fourth (4) analyses will show if the results in Table 1 change when adjusted for deprivation indicators (FSME and IDACI for England or WIMD/Townsend score for Wales), school absence and other variables specified for Wales.

The analyses below should be performed on children with CAs only to investigate the effect of risk factors on the association between a specific CA and KS2 results for an individual school subject, i.e. 1) English/Welsh (KS2- Table D), 2) Mathematics (KS2 – Table E) and 3) Science (KS2 – Table F). Both the crude ORs (in the univariate analysis) and adjusted ORs (from multivariate models) will be presented for each factor. Either FSME or IMD (depending on the results of the univariate analysis) should be included in the multivariable model.

Due to expected small numbers for some specific CA subgroups, this analysis may be possible for the most common CA subgroups only (e.g. all CHD, severe CHD, orofacial clefts, Down syndrome (any)).

**KS2 – Table D.** Logistic regression results on the association between sociodemographic factors and KS2 results (achieving level 4 or above (expected level)) for *English or Welsh* for linked children with congenital anomalies

**KS2 – Table E**. Logistic regression results on the association between sociodemographic factors and KS2 results (achieving level 4 or above (expected level)) for *Maths* for linked children with congenital anomalies

**KS2 – Table F**. Logistic regression results on the association between sociodemographic factors and KS2 results (achieving level 4 or above (expected level)) for *Science* for linked children with congenital anomalies.

Last KS2 table (KS2 – Table G) compares the percentage of children receiving special education support at KS2 tests between children with a specific CA and referent children (as given in KS2_TG_1).

In NPD, for those with documented Special Education Needs (SEN), KS2_TG_2 provides the number and the percentage of children with specific SEN type for cases and referent children.

**KS2 – Table G**. Special Education Needs (SEN) at KS2 tests (KS2_TG_1) and by SEN type if SEN identified (KS2_TG_2) for linked children with congenital anomalies by CA subgroup compared with referent children

In NPD data, until 2014, SEN type included pupils at school action plus and those pupils with a statement of SEN, but not those pupils at school action. From 2015, this includes pupils on SEN support or with a statement of SEN or EHC plan.

We (UNEW) are going to then test the differences between cases and referents by calculating relative risks for each CA subgroup compared to the referent children.

# 11. Key Stage 4 (KS4) –16-year-old children

**KS4 - Table A.** Sociodemographic status of linked children (typically aged 16 years) with Key Stage 4 (KS4) data (referent children and cases) by specific congenital anomaly (CA) subgroup for children with CAs

The descriptive table for child’s sex, gestational age (<32, 32-36, 37+ weeks and missing - for referents in Wales data only), major language, Deprivation index and Free school meal eligibility by CA subgroup is included (for subgroups where numbers permit).

**KS4 – Table B.** KS4 - Table B. GCSE or equivalents - number and percentage of children achieving 5 or more GCSE/GNVQs at grades A*-C (level 2) or A*-G (level 1) - for children with congenital anomalies and referent children

This descriptive analysis will produce the percentage of children with CAs and referent children (from the number of eligible children for each group) who achieved level 2 (5 or more GCSE/equivalents at grades A*-C), level 1 (5 or more GCSE/equivalents at grades A*-G) or any pass in GCSE or equivalent.

For English NPD data, the variables describing the highest examination category achieved

(KS4_EXAMCAT (2004/05 - 2012/13), KS4_EXAMCAT_PTQ (2013/14) and

KS4_EXAMCAT_PTQ_EE (from 2014/15)) can also be used to describe children’s KS4 achievement.

**KS4 - Table C.** KS4 results - 1) Unadjusted and 2), 3) and 4) Adjusted ORs of achieving 5 or more GCSE/GNVQs at grades A*-C or A*-G - for children with congenital anomalies versus referents - logistic regression results

1) Unadjusted
2) Adjusted for Free school meal eligibility (FSME – yes/no)
3) Adjusted for deprivation index (NPD IDACI tertiles or SAIL WIMD quintiles and Welsh LSOA Townsend deprivation scores and ranks, birth year and gestational age group (the latter two for Wales only)).
4) Adjusted for school absence

The first analysis (1) will show if the odds of achieving 5 or more GCSE/GNVQs at grades A*C or A*-G differ between children with a CA (by CA subgroup and any CA) and referents.

The second (2), third (3) and fourth (4) adjusted analyses will show if the results in Table 1 change when adjusted for deprivation indicators (FSME and IDACI for England or

WIMD/Townsend score for Wales), school absence and other variables specified for Wales

The analyses below will be performed on children with CAs only to investigate the effect of risk factors on the association between a specific CA and KS4 results.

Due to expected small numbers for some specific CA subgroups, this analysis may be possible for the most common CA subgroups only (e.g. all CHD, severe CHD, orofacial clefts, Down syndrome (any)).

The primary outcomes are: 1) achieving 5 or more GCSE/GNVQs at grades A*-C (Level 2) and 3) achieving 5 or more GCSE and equivalents at grades A*-G (Level 1).

The secondary outcomes are: 2) achieving 5 or more GCSE and equivalents at grades A*-C (Level 2) including English/Welsh and Maths as fundamental school subjects; 4) 5 or more GCSE and equivalents at grades A*-G (Level 1) including GCSE English/Welsh and Maths.

**KS4 - Table D.** Logistic regression results on the association between sociodemographic factors and KS4 results (achieving 5 or more GCSE/GNVQs at grades A*-C) - for children with congenital anomalies only

1) Achieved 5 or more GCSE/GNVQs at grades A*-C (Level 2) (yes/no)

**KS4 - Table E.** Logistic regression results on the association between sociodemographic factors and KS4 results (achieving 5 or more GCSE and equivalents at grades A*-C including

English/Welsh and Maths) - for children with congenital anomalies only

2) Achieved 5 or more GCSE and equivalents at grades A*-C (Level 2) including English/Welsh and Maths) (yes/no)

**KS4 - Table F.** Logistic regression results on the association between sociodemographic factors and KS4 results (achieving 5 or more GCSE and equivalents at grades A*-G) - for children with congenital anomalies only

3) Achieved 5 or more GCSE and equivalents at grades A*-G (Level 1) (yes/no)

**KS4 -Table G.** Logistic regression results on the association between sociodemographic factors and KS4 results (achieving 5 or more GCSE and equivalents at grades A*-G including

English/Welsh and Maths) - for children with congenital anomalies only

4) Achieved 5 or more GCSE and equivalents at grades A*-G (Level 1) including GCSE English/Welsh and Maths (yes/no)

Last KS4 table (KS4 – Table H) compares the percentage of children receiving special education support at KS4 tests between children with a specific CA and referent children (as given in KS4_TH_1).

For those with documented Special Education Needs (SEN), KS4_TH_2 provides the number and the percentage of children with specific SEN type for cases and referent children

**KS4 – Table H.** Special Education Needs (SEN) at KS4 tests and by SEN type if SEN identified for linked children with congenital anomalies by anomaly subgroup compared to referent children.

In NPD data, until 2014, SEN type included pupils at school action plus and those pupils with a statement of SEN, but not those pupils at school action. From 2015, this includes pupils on SEN support or with a statement of SEN or EHC plan.

We (UNEW) are going then to test the differences between cases and referents by calculating relative risks by a CA subgroup.

## 11.1. References

1. EUROCAT. Chapter 3.4: Multiple Congenital Anomaly Algorithm (version 19.11.2014). In: *EUROCAT Guide 1.4: Instruction for the registration of congenital anomalies (Last update version 20/12/2016).* Newtownabbey, UK: EUROCAT Central Registry, University of Ulster; 2013.
2. Garne E, Dolk H, Loane M, et al. Paper 5: Surveillance of multiple congenital anomalies:

implementation of a computer algorithm in European registers for classification of cases. *Birth defects research Part A, Clinical and molecular teratology.* 2011;91 Suppl 1:S44-50. doi:

10.1002/bdra.20777

3. Gartner A, Lester N. Briefing paper on LSOA Townsend deprivation scores calculated from unadjusted Census data. 2008. [http://www2.nphs.wales.nhs.uk:8080/hiatdocs.nsf/85c50756737f79ac80256f2700534ea3/1 7fdbca9920051368025772f003b5a35/$FILE/TownsendBriefing.pdf.](http://www2.nphs.wales.nhs.uk:8080/hiatdocs.nsf/85c50756737f79ac80256f2700534ea3/17fdbca9920051368025772f003b5a35/$FILE/TownsendBriefing.pdf) Accessed 09/03/2021.
4. Townsend P, Phillimore P, Beattie A. *Health and Deprivation: Inequality and the North.* London: Routledge; 1988.

# 12. Table 1. Congenital anomaly subgroups to use in the WP5 Education analysis

**(based on EUROCAT Subgroups of Congenital Anomalies (August 2016) with exclusions mentioned in chapters 3.2 and 3.3 in Guide 1.4)**

| **EUROCAT Subgroups** | **ICD10-BPA** | **ICD9-BPA†** | **Comments** | **Subgroup binary variable number (al)** |
| --- | --- | --- | --- | --- |
| **All anomalies *** | Q-chapter,  D215, D821,  D1810^^^, P350,  P351, P371 | 74, 75, 27910,  2281^^^, 76076,  76280, 7710,  7711, 77121 |  | al1 |
| **Structural anomalies** |  |  |  |  |
| Spina Bifida | Q05 | 741 | Exclude if associated with anencephalus or encephalocele subgroups | al6 |
| Hydrocephalus | Q03 | 7423 | Exclude hydranencephaly 74232. Exclude association with NTD subgroup | al7 |
| Congenital Heart Defects | Q20-Q26 | 745, 746, 7470-7474 | Exclude PDA with  GA <37 weeks Exclude peripheral pulmonary artery stenosis with GA <  37 weeks | al17 |
| Severe CHD | Q200, Q201,  Q203, Q204,  Q212, Q213,  Q220, Q224,  Q225, Q226,  Q230, Q232,  Q233, Q234,  Q251, Q252,  Q262 | 74500, 74510,  7452, 7453,  7456, 7461,  7462, 74600,  7463, 7465,  7466, 7467,  7471, 74720,  74742 | ICD9-BPA has no code for HRH and double outlet right ventricle | al97 |
| Transposition of great vessels | Q203 | 74510 |  | al19 |
| VSD | Q210 | 7454 |  | al21 |
| ASD | Q211 | 7455 |  | al22 |
| Tetralogy of Fallot | Q213 | 7452 |  | al24 |
| Coarctation of aorta | Q251 | 7471 |  | al32 |
| Cleft lip with or without cleft palate | Q36, Q37 | 7491, 7492 |  | al102 |
| Cleft palate | Q35 | 7490 |  | al103 |
| Oesophageal atresia with/ without trachea-oesophageal fistula | Q390-Q391 | 75030-75031 |  | al41 |
| Ano-rectal atresia and stenosis | Q420-Q423 | 75121-75124 |  | al44 |
| Diaphragmatic hernia | Q790 | 75661 |  | al48 |
| Gastroschisis | Q793 | 75671 |  | al50 |
| Multicystic renal dysplasia | Q6140, Q6141 | 75316 |  | al54 |
| Hypospadias | Q54 | 75260 |  | al59 |
| Limb reduction defects | Q71-Q73 | 7552-7554 |  | al62 |
| Craniosynostosis | Q750 | 75600 |  | al75 |
|  |  |  |  |  |
| **Chromosomal anomalies** |  |  |  |  |
| Down syndrome | Q90 | 7580 | With or without al17 and al40 | Al89 |
| Down syndrome with CHD | Q90 | 7580 |  | al89+al17 |
| Down syndrome without CHD | Q90 | 7580 |  | al89 excluding al17 |
| Turner syndrome | Q96 | 75860, 75861, |  | Al92 |
|  |  | 75862, 75869 |  |  |

**Footnote:** *All Anomalies = ALL cases of congenital anomaly, excluding cases with only minor anomalies as defined in Section 3.2 in Guide 1.4 for cases born post-2005. Cases with more than one anomaly are only counted once in the “All Anomalies” subgroup.

†EUROCAT ICD-9 codes are used with the British Paediatric Association (BPA) extension code:

<https://eu-rd-platform.jrc.ec.europa.eu/sites/default/files/EUROCAT-ICD9-with-BPA-Extension.pdf>
