# Supplementary material for: Associations of major congenital anomalies, long-term health conditions, and socioeconomic deprivation with educational attainment in Wales: A population-based data linkage study
Source: PLoS Med. 2026 Oct 16;23(10):e1005050. doi: 10.1371/journal.pmed.1005050 (PMC13705610; doi:10.1371/journal.pmed.1005050)
Supplement: Supplementary file 5 [file pmed.1005050.s008.docx]

Suzanne De Bruijn, Associate Editor

PLOS Medicine, [sbruijn@plos.org](mailto:sbruijn@plos.org)

Dear Dr. De Bruijn and Reviewers,

We appreciate the opportunity to revise and strengthen our paper.

Thank you very much for your valuable time, expertise, and constructive suggestions, which have been immensely helpful in improving our manuscript and strengthening the exploration of the findings. We were encouraged by reviewers’ support for the manuscript.

We have returned to our data to follow the advice of the expert reviewers to ensure that our data exploration is as complete as possible. This has, inevitably, taken time.

Currently, all school governors in Wales are being consulted on a revised funding formula for schools. The best way to inform this consultation is to offer a unified analysis of the relative contributions of identifiable risk factors. Our priority is to identify the children in greatest need, so that services and funding can be targeted.

We have tabulated our responses to all the comments and suggestions for further analyses.

Thank you for your help and support.

Yours

Sue

## Reviewers' comments:

| 1. | Reviewer #1: This is an extremely important study using a unique dataset from SAIL. While the findings are important, i have several technical concerns: | Thank you, these expert comments and publication in Plos Medicine will help us to change provision. |
| --- | --- | --- |
| 2. | 1. A range variables were adjusted in the analysis on the association between anomalies and education outcomes. However, some of these adjusted variables (e.g. hospitalisation) look like mediator / mechanism to me. If that's the case, they should not be adjusted because of overadjustment bias. I'd strongly recommend the authors to consider whether the included covariates are indeed confounders, and they should have some justification / explanation in the Methods. | Thank you for this helpful comment. We have included further analyses.  We believe that the impact of our paper derives from the raw data, as illustrated in figs 2 & 3. Data were modelled with and without SEN provision (now Table S8). We have now appended the sequential models, as S2 appendix. We now acknowledge the risk of overadjustment bias in our full models; however, we believe all variables to be informed by substantive knowledge of the underlying sociology or biology. Line 599.  Our models are explanatory, and not predictive; however, by using proxies for unmeasured confounders, such as attendance and ill-health outside the 3 named conditions, we risk overadjustment by controlling for intermediate variables (Schisterman et al 2009).  We have now included a justification for inclusion of ‘days in hospital’ Line 596:  We accounted for the 3 most common long-term childhood physical health problems: asthma, diabetes, epilepsy (NHS digital 2022). However, there are other conditions requiring hospitalisation that are unaccounted, such as cerebral palsy or injuries. To mitigate the difficulties of identifying all illness in electronic health records, and take some account of school attendance, we adjusted for time spent in hospital.  We acknowledge that hospitalisation is not equivalent to attendance (Fleming et al 2024), and further exploration is warranted. Our supplementary S2 file presents analyses with and without hospitalisation and co-morbidities: we saw little difference in key associations.  Most children with and without anomalies do not have SEN provision. But, as in table 8, SEN and anomalies are associated. However, there are other reasons for SEN, such as autism or ADHD, which are not accounted, making SEN a proxy for perceived educational difficulties.  To address concerns, we have now included a supplementary xl file detailing sequential development of the explanatory logistic regression models at each attainment level:  Anomalies only, unadjusted  Anomalies, year of birth, sex, preterm birth  Anomalies, year of birth, sex, preterm birth, deprivation  Anomalies, year of birth, sex, preterm birth, deprivation, co-morbidities  Anomalies, year of birth, sex, preterm birth, deprivation, co-morbidities, SEN  Anomalies, year of birth, sex, preterm birth, deprivation, co-morbidities, SEN, interaction terms |
| 3. | 2. It seems that at least one education outcome was measured during / after the Covid pandemic (PHW 2022, line 108). Were there noticeable difference between the findings before and after Covid? While statistical comparison might not be possible, even descriptive findings would be useful. | We apologise, this reference was retained in error. Data collection ended 31.12.2019. No education data were generated during the pandemic, and data collection is yet to restart. (previously line 442-3). Now line 561 |
| 4. | 3. Table 9 is particularly worrying as to overadjustment bias. Some variables, e.g. year, could be better controlled for as a spline rather as categorical variable.  The ORs of the adjusted variables (e.g. WIMD) should also not be reported as you're adjusting their downstream mechanisms (e.g. anomalies). As a result you cannot compare ORs in that table. The OR for WIMD would be underestimated.  If you do want to examine the association of WIMD with education outcome, you should include a separate model including WIMD and some very basic prognostic variables, e.g. age, sex. This affects some interpretations as well. | We agree that analyses of non-linear relationships often involve polynomial functions; however, it is unclear as to where nodes/ knots should be placed for a spine-based model (Jennings et al 2025). The impact of year of birth in this study appears uniform for ks2, but not ks4, where there are only 5 years of data. We have re-analysed with year of birth as a continuous variable, without year of birth in supplementary analyses (Tables S9, S10). To address the question of the provenance of changes over time, we now cite public domain commentary on the KS2 assessments line 563:  The KS2 teacher assessment has been reviewed, but there are no reasons to question their internal validity (Donaldson 2015 p.74).  We found no overall association between maternal WIMD and congenital anomalies (most deprived vs. least deprived OR 1.03, 0.97-1.10.), and the raw data in table S3 indicates little difference in proportions. We now state this in the paper. (Only gastroschisis, cleft lip and multicystic renal dysplasia were more prevalent in the most deprived quintile.)  We acknowledge that this runs counter to the literature in the area (Yang et al 2008, Miao et al 2021, Hall et al 2024, NHS Digital 2024). Line 534, under limitations  The absence of association between WIMD and congenital anomalies runs counter to the literature in the area (Yang et al 2008, Miao et al 2021, Hall et al 2024) NHS digital 2024); this may be explained by the association between maternal age and SES in Wales (Joshi & Hawkes 2006).  Table S4 presents the data for WIMD and educational attainment. The trends are clear. |
| 5. | 4. There are some interesting analysis that could be done with the data but not explored at the moment. e.g. whether SEN provision interaction with anomalies? i.e. could/would SEN reduce some of the adverse association of anomalies? Another is to decompose the association of WIMD on education outcome by anomalies, answering the question that to which extent anomalies is a mechanism for socioeconomic difference in education outcome. These are relevant to the studies but could be considered outside the scope - so feel free to leave this suggestion. | We agree that further analyses would offer additional insights. The relationship between SEN and anomalies was included in supplementary material. (S2a, S2b).  Interaction terms  Taking this expert advice, we have now reported these in the main text. The data file has been added to supplementary material, as it is too large for the manuscript.    We investigated the associations between child’s sex, deprivation, SEN provision, hospitalisation and co-morbidities by testing pairwise interaction effects between a) anomalies and SEN, and b) anomalies and SES, in the final model, supplementary xl file. Statistically significant interaction terms included: SEN and sex, SEN and FSM eligibility, (except for 5 GCSEs C-A* with EWMS); and SEN and income, mainly at KS2. Adjusting for interaction terms made little difference to key associations, but widened confidence intervals: for example, attaining 5 GCSEs C-A* with EWMS remained statistically non-significantly associated with congenital anomalies, and FSM remained the strongest association, other than SEN provision.  We have now expanded  Methods  We assessed the associations between education attainment and the combined effects of SEN and anomalies/ sex/ FSM/ATS/ ENV / income / hospitalisation in turn and of anomalies and ATS/ FSM/ ENV / income / maternal deprivation in turn as interaction terms. In this exploratory analysis, we did not account for multiple testing (Supplementary S2 File)  Results of this analysis have been moved from supplementary material to manuscript line 418  Inclusion of interaction terms left key findings essentially unchanged; confidence intervals were slightly widened. Most interaction terms including anomalies were not significant, other than the combined effect of SEN school-support with anomalies for KS4 with 5 GCSEs C-A* with EWMS, and statementing combined with anomalies for KS4 any 5 GCSEs. The combined effect of SEN with FSM was significant at all assessments except KS4 with 5 GCSEs C-A* with EWMS; SEN combined with income was significant for some outcomes. In the main, SEN combined with hospitalisation was not significant. Of the WIMD components, only income interacted with SEN, and none interacted with anomalies (S2 file). |
| 6. | Reviewer #2: This manuscript is a study using linked congenital anomaly register and education data to examine the education outcomes for children with congenital anomalies at ages 11 and 16, with particular attention to receiving services for special education needs and socioeconomic factors. This paper is quite difficult to follow with too many acronyms, and too much details of some methods and little about other aspects of the study. There are also some questions about methodology. | Thank you for this suggestion. We have removed some acronyms. There is always a balance to be struck, so please let us know if we should remove more. Some acronyms are widely used e.g. OR.  Specific responses are below. |
|  | Specific methodological questions: |  |
| 7. | -Combining 'all anomalies' is not useful, the groups are too different. Particularly the anomalies that impact cognition (e.g Down Syndrome) these should be separated out and possibly excluded.  To combine Down syndrome and a less severe anomaly, such as cleft palate, that has been shown not to impact education outcomes, is misleading.  However when reported separately, in group where there are few anomalies, this is also is not much use as there will be very large confidence intervals in models. And a different result may be caused by one child.  I would suggest making larger broad groups, perhaps classify on severity or functional/structural anomalies, or classify by severity.  What is in 'other anomalies' group? A description of main anomalies in this group would be helpful as this group includes over half of all anomalies. Could this group at least be divided by severity or by body system the anomaly impacts. | Thank you. We used the established EUROCAT ‘al1’ binary variable to designate ‘any major anomaly’. Congenital anomalies are grouped according to shared clinical characteristics and aetiologies. EUROCAT’s definitions and groupings have been refined over four decades. EUROCAT’s chromosomal groups include all children with the chromosomal anomaly whether or not they have other anomalies, because there is a common genetic aetiology. The “isolated anomalies” groups exclude children with chromosomal anomalies e.g. a child with Down syndrome and a cleft would be analysed only under ‘Down syndrome’ (line 262). We added the definition of isolated anomalies to the methods, line 136 ref [65]:  Isolated anomalies are defined by EUROCAT as a congenital anomaly in one organ system only or with a known sequence where multiple congenital anomalies cascade as a consequence of a single primary anomaly (Morris et al., 2021).  *† children with Down or Turner syndrome with additional anomalies are included here, as the problems have a common genetic aetiology.*  Severity of handicap varies within each condition; EUROCAT are unable to record severity of the lesion, as it often unknown when cases are reported.  As suggested, we excluded the children with Down syndrome in a sensitivity analysis (Table S7): the overall associations remained. We reported on the pass rate amongst children with Down syndrome, but low numbers precluded inferential analyses (note to table 11).  We analysed the data by anomaly (table 11), but low numbers precluded analysis for some anomalies. If categories generated contained <5 children, results of analyses were not reported. We report on these where numbers are sufficient. We acknowledged that there are insufficient data for analysis of individual anomalies, and “low numbers risk obscuring associations”. Was line 436, now 554.  We have clarified the EUROCAT definition in ‘methods’ line 127.  Major congenital anomalies are structural changes that have significant medical, surgical, social or cosmetic consequences for the affected individual, and typically require medical intervention. (…) They account for most of the mortality, morbidity, and disability related to congenital anomalies (WHO 2014 p.4, EUROCAT 2025, Kinsner-Ovaskainen et al 2025 p.6).  A strength of this paper is the EUROCAT taxonomy and standardisation.  Thank you for prompting clarification. We have listed the anomalies included, with ICD10 codes in supplementary material (S19 List1) and added a footnote to table 4: “To delineate congenital anomalies and achieve comparability of prevalence rates between registers, the count of cases with all anomalies (al1) includes all cases with one or more codes in the Q chapter of ICD10 and a very limited set of conditions coded outside the Q chapter, as specified in the definition of subgroups given in Chapter 3.3 (EUROCAT 2013 P.81) and: Q-chapter, D215, D821, D1810^ , P350, P351, P371 (EUROCAT 2013 p.91).”  Reclassifying anomalies would entail revisiting the whole of EUROCAT’s work. We note limitations of the taxonomy regarding facial clefts.  The ‘other anomalies’ are delineated in supplementary material, S19 List 1.  Thank you for prompting clarification. Was line 195 line, now line 139.  Just under half the children with major anomalies could be categorised within the ‘isolated structural anomalies’ taxonomy. The remainder had more than one anomaly or a genetic or other syndrome or a major anomaly with too few children for analysis (S9 List 1).  None of the groups that were combined as ‘other’ was large enough for analysis. |
| 8. | -Why choose epilepsy, asthma & diabetes as long-term conditions that may impact education? Is the hypotheses for these conditions that children will miss school due to the conditions or that they are cognitively impacted? | Thank you for prompting clarification. We have added a reference to support the selection of the most common childhood illnesses. This analysis sought to explore markers for educational attainment, to assist targeting of support. Currently, there is uncertainty regarding the impact of common long-term conditions. We added, line 79:  Of the three most common long-term conditions requiring hospitalisation in childhood (NHS digital 2022), epilepsy is linked with lower academic attainment (Fleming et al., 2019), but the literature offers no consensus over asthma (Milton et al., 2004, Sturdy et al., 2012, Pretorius, 2004, Lundholm et al., 2020) and diabetes (Liu et al., 2022, Oakley et al., 2020).  We adjusted for the long-term conditions that dominate UK primary care records of childhood ill-health: asthma, epilepsy and insulin-dependent diabetes (NHS digital 2022, Valabhji et al 2024).  We stated in the discussion.  “We are unable to separate the impact of seizures and any underlying brain injury from the sedative effects of anti-seizure medicines.”  And, following advice, have modified, now line 509:  We are unable to separate the impact of illness, including seizures and any underlying brain injury, from effects of school absence or medicines, whether sedative or stimulant. |
| 9. | - Although the authors have included flowcharts for results, a flow chart of all of the datasets, dates, outcomes and numbers would be very helpful.  The paragraphs in 'data sources' with all of the acronyms is confusing. Rather than acronyms, could you call datasets a name based on the data they provide (eg education, prescription etc)?  How many children have outcomes in both years?  Can you examine their trajectory to see impact of SEN and SES over years? | Thank you. We have clarified as a footnote to table 2:  Only children identified in all data sets are included in this analysis.  Thank you for prompting additional clarification. We have tabulated the data sources used as an additional table with links to information on the data sources (now Table 1).  To maintain consistency with other SAIL work, it is not possible to rename the databases.  Line 182 stated: Most children present in the key stage 4 cohort were also present in the key stage 2 cohort: 4,350 children with anomalies and 122,100 children without anomalies contributed to education records at both stages  Thank you for suggesting these new analyses. The changes in the impact on KS2 success of SEN and FSM over the years are informative. There were no observable trends at KS4. The results are in the supplementary file, Table S13 & S14. |
| 10. | -The increasing use of SEN I'd suggest is a proxy for severity of anomaly. Do children that receive SEN perform as well as those without in the rest of the population?  Just adjusting for SEN in the model does not make sense as it will be on the causal pathway between the anomaly and the education outcome (table 6). The models should be stratified by SEN (as presented in Table 8) –  There is a big difference in results between males & females, particularly between the different years - it would be interesting to stratify models by sex  General comments: | Thank you. SEN provision aims to target educational need, rather than severity of disability. we have added to results. “Not all anomalies were associated with statementing (oesophageal atresia, ano-rectal atresia, diaphragmatic hernia, and at KS4 only, gastroschisis, craniosynostosis, multicystic renal dysplasia) (was S2a table now S5a).” Line 329.  We made no assumptions, and reported on the moderating effect of SEN: SEN provision narrowed the gaps in pass rates between children with/without anomalies and those eligible/ineligible for FSM, but only for the KS4 higher level outcome (Table 9). Analyses without SEN were reported in the supplementary material: (Table S4 now S8). The impact of SEN is important to us: we need to know how effective it has been.  Thank you for this insightful suggestion. Adjusted analyses have now been undertaken within each of the SEN strata (Table S15a-d), to complement table 9. It is interesting to observe that children without SEN provision are more vulnerable to the effects of hospitalisation.  A note on the moderating effect of SEN was included in the supplementary material, now in main text line340  Moderators  We explored whether the association between anomalies and poor outcome was stronger amongst children not receiving SEN support than those receiving SEN support. SEN provision reduced the differences in attainment rates for 5GCSEs C-A* with EWMS, but not for other outcomes, suggesting that it was only a moderator for the highest level of attainment (Table 8).  Thank you. We have analysed boys and girls separately (Table S12a,b) |
| 11. | The background is very confusing, it jumps around from anomalies, disability, SES, poverty and co-morbidities. | Thank you. The background text has been re-organised. We agree that many factors potentially affect educational attainment, but all should be explored. |
| 12. | Page 2, line 28: Disability and learning difficulties may emanate from congenital anomalies. Use of the word 'emanate' seems strange? | We think ‘emanate’ is the most apposite verb here. ‘Cause’ would be incorrect. |
| 13. | Line 37: Congenital anomalies affected 2.04% - is this severe anomalies? | Thank you. We have clarified that these data relate to major anomalies, as referenced by EC definitions. |
| 14. | Line 59: add (Compulsory schooling ends at age 16) to methods, not in aims, it is confusing. | This background information has been moved. |
|  | Methods: |  |
| 15. | -Congenital anomalies - who reports them to the Welsh Congenital Anomaly and Information Service (CARIS) register? | Thank you for prompting clarification. We have added: Reports of suspected congenital anomalies are received from clinicians, including obstetricians, paediatricians and surgeons, and families.  This is explained in the CARIS website, which was referenced.  Full details are available on the reporting page of that website.  <https://phw.nhs.wales/services-and-teams/caris/about-caris/reporting-to-caris/> ADD to supp info. |
| 16. | -Inclusion criteria "Our cohort comprised all live born children with prescription records." So, children who had not had a prescription were excluded? | We apologise for this is misunderstanding. Line 103 indicated: Approximately 85% of Welsh primary care practices contribute routinely collected data to SAIL, including all primary care prescriptions, from 86.2% of the population and 86.5% of children, as the Welsh Longitudinal General Practice (WLGP) dataset (SAIL, 2023). Table 6 indicates that most children in the analysis had no prescriptions recorded. We have stated: “children with primary care records” (was line 137). We explained in the supplementary material:  Prescription data  Only 86% primary care practices in Wales contribute data to SAIL. Data submission is voluntary and not remunerated. In this cohort, the population with prescription data had a similar mean deprivation (Townsend) score (Townsend 1987) to the population without prescription data (mean 0.603 [SD 3.678] vs 0.612 [3.580]), which was statistically insignificant, P 0.38, t 0.87, df-t 281,962.8, with a very small effect size (r=0.002). |
| 17. | -"Children or young people are considered to have SEN if they have a learning difficulty or disability which requires additional educational provision, over and above routine schooling." Who decides this? | Thank you. We have added, line 200: “Needs are assessed by schools and the local education authority; decisions are sometimes appealed.” with a reference.  It is beyond the scope of this paper to explore this: further details are available chrome-extension://efaidnbmnnnibpcajpcglclefindmkaj/https://educationtribunal.gov.wales/sites/educationtribunal/files/2025-01/etw-annual-report-23-24.pdf . |
|  | Results: |  |
| 18. | Start by saying how many children included in study sample. | Thank you; we have clarified in the first line of the results: We report on 256,568 children at age 11, KS2, and 128,727 at age 16, KS4. |
| 19. | Table 5 - why have median UQ and LQ for asthma prescriptions? | Median and quartiles offer a measure of central tendency. We are happy to take the reviewer’s expert advice and remove the data. |
| 20. | Why the large difference in KS2 pass results by year of birth? | This is an interesting and controversial point. We commented: Birth year also affected educational attainment, mainly for teacher-assessed KS2. This is in line with official statistics (Stats Wales 2018 Key Stage 2 results: <https://statswales.gov.wales/Catalogue/Education-and-Skills/Schools-and-Teachers/Examinations-and-Assessments/Key-Stage-2/results-by-localauthority-level>  We have added to discussion: Teacher assessment for KS2 has been reviewed (Donaldson 2015); publication of results ceased in 2018 (Stats Wales 2018). |
|  |  |  |
| 21. | Reviewer #3: In this study, the authors use data from SAIL for live births in Wales from 1998-2007 with educational outcome at 11 and 16 years of age, to examine whether educational attainment is associated with birth defects, chronic conditions or poverty. The methods and results are generally well-described, but both the introduction and discussion could be improved,  A major concern for this reviewer is what is the value add of this manuscript, compared to a recent publication (Glinianaia SV, Tan J, Morris JK, et al. Academic achievement at ages 11 and 16 in children born with congenital anomalies in England: A multi-registry linked cohort study. Paediatr Perinat Epidemiol. 2024;38(5):411-425. doi:10.1111/ppe.13049)? The authors could bring this up in the introduction, and indicate what's new in this study that adds to the knowledge base. | Thank you  The introduction and discussion have been reworked.  This is an interesting point. The education systems and GDP *per capita* are very different in the four countries of the UK.  Glinianaia et al 2024 is referenced in para 2 of the introduction, alongside papers from other countries. This study covers an earlier time period, does not offer whole population coverage, and does not account for co-morbidity, hospitalisation, access to services, the environment, sex, or preterm birth, and does not explore SEN provision. The comparator groups were formed by matching, using an area-based measure of SES, which includes an education domain: this risks a circular argument. We opted to report on the overall outcomes rather than the 3 subject areas to limit the number of comparisons and concentrate on the outcomes that matter for common career options.  We have added to the penultimate paragraph of the introduction:  Current evidence is limited by the paucity of large whole-population studies with objective measures of academic achievement, including key covariates, such as socio-economic status (SES) as income, access to services, environment, maternal deprivation, child’s sex, preterm birth (Cocomello et al., 2021), hospitalisation, comorbidities, and the impact of SEN provision. |
|  | The following are both general and specific comments for the authors to consider: |  |
| 22. | The authors should review their references with care, and ensure that the selected references are the best choices to support statements in the text. Just as one example, for the text on line 28, reference 6 is actually a study of outcomes in hydrocephaly, a very specific condition that is sometimes acquired rather than congenital in nature. There are more comprehensive sources the authors could choose. | Thank you: we agree, and have reduced and re-arranged references accordingly. As you say, difficulties emanate from many or most anomalies; however, the literature is dominated by CHD. We should welcome suggestions for further references, based on your expertise. |
| 23. | Birth defects prevalence are generally reported per 1000, or 10000 births, not as percents to two decimal places. This should be corrected throughout the manuscript, as well as in any tables or figures. | Thank you, we have followed your advice. Child poverty rates are given as %s, so uniform presentation seemed easier. |
| 24. | The paragraph beginning in line 44 needs more substance, particularly for the association of chronic health conditions with academic performance. Many other conditions could be mentioned here. | Thank you. PubMed was checked for updated references. We justified the selection of the 3 most common long-term childhood illnesses, and referenced reports assessing their impact on education, now line 79: Of the three most common long-term conditions requiring hospitalisation in childhood (NHS digital 2022), epilepsy is linked with lower academic attainment (Fleming et al., 2019), but the literature offers no consensus over asthma (Milton et al., 2004, Sturdy et al., 2012, Pretorius, 2004, Lundholm et al., 2020) and diabetes (Liu et al., 2022, Oakley et al., 2020).    We clarified: (see point 8)  We adjusted for the long-term conditions that dominate UK primary care records of childhood ill-health: asthma, epilepsy and insulin-dependent diabetes (NHS digital 2022, Valabhji et al 2024).  We stated in the discussion.  “We are unable to separate the impact of seizures and any underlying brain injury from the sedative effects of anti-seizure medicines.”  And, following advice, have modified, now line 509:  We are unable to separate the impact of illness, including seizures and any underlying brain injury, from effects of school absence or medicines, whether sedative or stimulant.  We acknowledge under ‘limitations’ that other conditions should have been considered, but other reviewers raised the issue of overadjustment.  Data on school attendance, social care interventions (Lowthian et al., 2024), ethnicity, breastfeeding (Adams et al., 2023), multiple births, autism, ADHD, maternal ill-health, medication use or substance misuse were not included (Morris et al., 2021). |
| 25. | The authors make generally good use of active voice, but the manuscript should be read in entirety to identify sentences that might be improved. An example, the sentence beginning on line 50 which includes the phrase 'it is important to note . . .' | Thank you. We have scrutinised the grammar and syntax.  Line 50 has been re-written. We agree that the demonstrative clause was superfluous. |
| 26. | Check the manuscript to ensure that all abbreviations are spelled out prior to first use, as well as listed on line 491. One that is missed is EHC on line 152. | Thank you for the attention to detail. We wrote: education, health and care [EHC] plans). We removed the acronym, as it does not appear elsewhere.  We have reduced the number of acronyms, but would welcome further advice on this point. |
| 27. | In the methods, provide more detail on the analysis plan. A) Were DAGs or other conceptual models used to drive the development of statistical models?  B) Were any sensitivity analyses performed?  c) On line 162 there's a mention that no imputation was done, so tell the reader more about the nature of missingness and how that might have affected the results?  d) Was any modeling to evaluation interaction or effect modification done?  e) Did the authors consider propensity score matching, given the rarity of births with birth defects? | a) We appreciate the suggestion for a diagram, and agree that visualisation is important. We have opted for a simple schema (Fig. 1), acknowledging the complexity of the situation. In practice, the interactions between measurable and unmeasurable risk factors are too complex (and often untested) for a readable DAG. We have expanded the text on ‘Analysis’. Modelling reflected the pre-published protocol (Morris et al 2021) and lived experience. We have added a supplementary file detailing the construction of the models and interaction terms. b) Following this advice, we have undertaken a sensitivity analysis around FSM eligibility, as in Glinianaia et al 2024. FSM remained a strong predictor of success. Table S16. c) We agree this is important, and have added further details in Table S2. If advised, we could move this table into the manuscript.   Under ‘limitations’ we wrote:  … many children educated privately, at home, outside ‘mainstream’ schools or outside Wales were lost to the study. It is estimated that, during primary school years, some 2% children are educated privately and 4.3% in special schools (Evans et al., 2019). Incomplete data on special schools disproportionately excludes children with severe disabilities.  We have added the reasons behind absent data (Table S2). We explored the relationship between prescribing data and SES in supplementary material: we found no association.  We have added to limitations line 583: Missing data were largely confined to the outcome variable and missing data for other variables were no more than 5% (Dettori et al 2018). Since imputation may underestimate variability whilst never fully compensating for missing data in cohort studies (Li et al 2024), no imputation analyses were undertaken. This complete case analysis is not transferable to children outside mainstream education.   d) We apologise for not presenting interactions more prominently, as discussed in comments 2 & 5. We have expanded the text in the supplementary material and added a supplementary xl file (S2 file) with these analyses. e) Our analysis is not designed to establish causation: our aim was to identify modifiable risk factors. We have added, line 600:   These explanatory analyses aim to identify modifiable risk factors, where interventions might be targeted. Propensity score matching might obscure risk factors or leave variables excluded in the propensity score unbalanced, confounding effect estimates (Seeger et al 2005). |
| 28. | In Table 2, reformat to avoid line wrap. | Thank you, we apologise for this formatting error. |
| 29. | In Table 3, round percents to a single decimal place = all are 0 in the second decimal anyway except at bottom of table. | Thank you, we apologise for this formatting error. |
| 30. | Table 5, why were these conditions selected, and not ADHD, ASD, cystic fibrosis, etc? | Thank you. We now explain In limitations: (see point 2)  We accounted for the 3 most common long-term childhood physical health problems: asthma, diabetes, epilepsy (NHS digital 2022).  We have updated: line 588  Data on school attendance, social care interventions (Lowthian et al., 2024), ethnicity, breastfeeding (Adams et al., 2023), multiple births, autism, ADHD, cystic fibrosis, maternal ill-health, medication use or substance misuse were not included (Morris et al., 2021).  The number of children with cystic fibrosis is very small: in our experience, these children do well at school and university. Cystic fibrosis is not a EUROCAT anomaly, and we were restricted by EUROCAT definitions. |
| 31. | In Table 7, but check on all tables, spell out any acronyms used in each table. What is SEN? Round percents to one decimal place. Be clearer about the odds ratios, are these crude or adjusted in Table 7. A better way to present is OR (X.XX-X.XX) rather than in three separate columns. These comments apply to subsequent tables as well. | Thank you for this advice, which has been followed. To explain SEN we have added to the previous text (was line 146), now line 196:  Children or young people are considered to have SEN if they have a learning difficulty or disability which requires additional educational provision, over and above routine schooling; this does not always parallel severity of physical disability. Procedures for service allocation are described in the 2002 code of practice (Welsh Assembly Government, 2004), which has been revised (Welsh Government 2021). Needs are assessed by schools and the local education authority; decisions are sometimes appealed.  Table 7 now 8 has been modified, as suggested, and a note added to clarify that unadjusted odds ratios were calculated. |
| 32. | Line 318, how were cases of 'isolated' birth defects determined? For example, Down syndrome, a sizable proportion of these cases also have cardiac anomalies or other defects. | We agree, but we used EUROCAT coding, which identifies chromosomal anomalies as the primary anomaly. The multiple anomalies experienced by children with Down syndrome are not coded separately in EUROCAT.  Line 213 *et seq* has been updated, line 256: Just under half the children with major anomalies could be categorised within the ‘isolated structural anomalies’ taxonomy. The remainder had more than one anomaly or a genetic or other syndrome; examples include multiple renal anomalies, talipes in addition to brain malformations or foetal alcohol syndrome (Table 3).  We expanded a footnote to Table 3 to clarify:  *† children with Down or Turner syndrome with additional congenital anomalies are included here, as the chromosomal anomaly determines appearance.* |
| 33. | In Table 10, there are some glaring findings that call out for more attention. These include spina bifida K2 pass, not reflected in any of the other educational outcomes, as well as Turner syndrome.  The hospitalization risk by day seems to be the only estimate for a linear outcome, maybe place at bottom and include a note. | Thank you for these insights. The numbers of children with some anomalies in mainstream schools were too low to draw conclusions. We have now added: line 438  Insulin use, Turner syndrome, limb reduction, diaphragmatic hernia, *spina bifida, hydrocephalus, craniosynostosis,* and ano-rectal atresia did not adversely affect outcomes at age 16. There were some positive outcomes for *spina bifida* at age 11*,* but numbers in mainstream schools were low.  Thank you.  We have re-ordered tables 9 & 10 and added a note: *Hospitalisation for each day is the only continuous variable in the analysis.* |
| 34. | In the discussion, avoid making claims far beyond the results. For example, the first sentence on line 353. Yes, the study shows associations for poor educational attainment, but there are likely more children 'left behind' who don't have birth defects or chronic conditions - the role of poverty is quite important here. A more nuanced discussion seems in order. | Fig 3 illustrates the impact of deprivation + anomalies.  We have redrafted line 476:  The children least likely to succeed lived in poverty with major congenital anomalies (Fig.3). Educational attainment at age 16 was more strongly associated with deprivation than anomalies or co-morbidities, when hospitalisation, sex, preterm birth, and SEN provision were accounted.  The discussion has been redrafted and updated to incorporate the findings of the additional analyses. |
| 35. | Line 414, a study linking birth defects to developmental outcomes in early childhood might be a useful reference: Delgado C, Ullery MA, Zeng G, et al. Elevated risk for developmental disabilities in children with congenital heart defects. Birth Defects Res. 2023;115(18):1708-1722. doi:10.1002/bdr2.2246 | Thank you, this is a great paper; it is now included in the discussion. |
| 36. | In the limitations section comment on potentially reduced generalizability due to the refined population included in the study. Also on potential issues with missingness. | This is a helpful suggestion. We added line 583:  Missing data were largely confined to the outcome variable and missing data for other variables were no more than 5% (Dettori et al 2018). Since imputation may underestimate variability whilst never fully compensating for missing data in cohort studies (Li et al 2024), no imputation analyses were undertaken. This complete case analysis is not transferable to children outside mainstream education.  See comment 27. |
| 37. | In the final paragraph, perhaps comment on how public health and social welfare programs might ameliorate some of the observed effects of poverty. | We agree this is important. I serve on our local council and governor to a primary school, in one of the poorest wards in Europe, where the problem of poverty sometimes seems insuperable. We suggest unification of the 3 support services and triaging poor children with disabilities. Plos Medicine support will bring these suggestions to the attention of the politicians of all parties. |
| 38. | Let's leave this to the editors' discretion, but this reviewer would not call this a retrospective cohort study (line 61) as by definition all cohort studies are prospective. Better terms are nonconcurrent cohort study or historical cohort study. | The adjective ‘retrospective’ has been removed. We are happy to take the editor’s advice here. |
| 39. | All in all, its a nice paper, but not quite ready for publication in its present form. | Thank you, the guidance is very helpful. |
|  |  |  |
| 40. | Reviewer #4: This is a very important paper covering a wide range of questions all dealing with "the cumulative impact of congenital anomalies, long-term conditions and poverty, using health data linked to educational data for children in Wales. | Thank you |
|  | I have a number of major comments. |  |
| 41. | First, the scope of this paper is so vast that essential detail was not sufficiently developed and as a result conclusions are too wide-ranging. For example, children / young people with congenital anomalies form a very heterogeneous group. The impact that some anomalies on educational attainment will be vast whereas for other (hypospadias or cleft lip) the impact will be small. Conclusion should always be stratified according to the specific anomaly. | Thank you. This analysis has allowed us to identify gastroschisis and hypospadias as anomalies requiring additional attention. We have now emphasised this in the discussion. For our work to have impact, we shall need to more beyond the low numbers of children with individual anomalies. We found that poverty was a more powerful predictor of educational outcome than any anomaly, other than Down syndrome. |
| 42. | Second, for more than 50%, the type of congenital anomaly was not available which undermines the wider applicability of this paper. | Unfortunately, many children have more than one anomaly.  To our knowledge, this is the only population-based cohort study to assess educational attainment in children with congenital anomalies accounting for poverty and access to services. While we were unable to analyse all subgroups of congenital anomalies, due to small numbers, we believe that these results are generalisable as poverty and access to services were stronger determinants of educational attainment than type of anomaly.  The emergence of poverty and access to services as a stronger association than ‘any anomaly’ reduces the need to individualise major anomalies.  We clarified. Was line 195, now line 256:  Just under half the children with major anomalies could be categorised within the ‘isolated structural anomalies’ taxonomy. The remainder had multiple anomalies or a genetic or other syndrome or an anomaly with too few children to analyse; examples include multiple renal anomalies, talipes in addition to brain malformations or foetal alcohol syndrome (Table 3). |
| 43. | Third, given the importance of the data, the paper gives little information about how the information about comorbidities was derived from primary care data for this group of children / young people. | Thank you for prompting clarification. We stated:  Medicated asthma, type 1 diabetes, epilepsy or cardiac conditions, were indicated by primary care records of > 1 prescription for any of these conditions (UK, 2025) by the 1^st^ May of a child’s key stage 2 and 4 assessment years; we discounted single prescriptions as likely to represent non-adherence.  We have now added line 209: ATC (anatomic, therapeutic chemical) codes related to these conditions were identified, as previously (Divin et al 2023, Given et al 2023, Damkjaer et al 2022), and matched with Readv2 codes (lists are available in ‘Codes’ below).  The code lists used in the study are listed at the end of the text as public domain documents, available to readers. Coding of medicines used in this study is available in the public domain, in the phenotype library  [https://conceptlibrary.saildatabank.com/phenotypes/PH1909/version/3996/detail/](https://eur03.safelinks.protection.outlook.com/?url=https%3A%2F%2Fconceptlibrary.saildatabank.com%2Fphenotypes%2FPH1909%2Fversion%2F3996%2Fdetail%2F&data=05%7C02%7CS.E.Jordan%40Swansea.ac.uk%7Ce21d135f41b54815bb0c08ddb48d2fb5%7Cbbcab52e9fbe43d6a2f39f66c43df268%7C0%7C0%7C638865240665538506%7CUnknown%7CTWFpbGZsb3d8eyJFbXB0eU1hcGkiOnRydWUsIlYiOiIwLjAuMDAwMCIsIlAiOiJXaW4zMiIsIkFOIjoiTWFpbCIsIldUIjoyfQ%3D%3D%7C0%7C%7C%7C&sdata=d%2BDGXimfA5R8YQEe8D7u9RhVHhu1S147N5ogXmpJYR8%3D&reserved=0) |
| 44. | Fourth, I'm also concerned about how the data on SEN provision was used. From my perspective, an approach analysing education outcome adjusting for SEN provision seems to be in appropriate given that SEN provision identifies children highly likely to have poor educational outcomes. In other words, it's a statistical approach where part of the independent variable (educational outcome) is part of the dependent variables in the model (SEN provision). | We agree; accordingly, data were analysed with & without SEN provision. (previously tables 6, 9 and S4, now 7, 10 and S8). Overall findings regarding deprivation were similar, but, as reported, congenital anomalies were more strongly associated with attainment when SEN was not accounted.  Most children with and without anomalies do not have SEN provision. As shown in table 8, SEN and anomalies are associated. However, there are other reasons for SEN, such as autism or ADHD, which are not accounted, making SEN a proxy for perceived educational difficulties.  We acknowledge these concerns, and have undertaken additional analyses:  To avoid over-specification in these explanatory models (Fig.1), results were reported with and without SEN provision (tables 7, S8), Down syndrome (S7), or deprivation (S18), separately for boys and girls (S12a,b), and level of SEN provision (S15a-d). Low numbers restricted the number of covariates in some models. We are grateful for the reviewers’ suggestions, which identified further insights.  We assessed the associations between education attainment and the combined effects of SEN and anomalies/ sex/ hospitalisation/ deprivation measures in turn and of anomalies and deprivation measures in turn as interaction terms (Supplementary S2 file).  We have now included a supplementary (S2) xl file detailing sequential development of the explanatory logistic regression models at each attainment level:  Anomalies only, unadjusted  Anomalies, year of birth, sex, preterm birth  Anomalies, year of birth, sex, preterm birth, deprivation  Anomalies, year of birth, sex, preterm birth, deprivation, co-morbidities  Anomalies, year of birth, sex, preterm birth, deprivation, co-morbidities, SEN  Anomalies, year of birth, sex, preterm birth, deprivation, co-morbidities, SEN, interaction terms |
| 45. | Fifth, the terms "educational outcomes", "educational attainment", and "academic achievement" seem to be used interchangeably. Are these referring to the same outcome? I'd welcome a more consistent use of precisely defined outcomes. | Thank you. We have standardised to “educational attainment” as far as possible. Outcomes and attainment are not synonymous, particularly at age 11. ‘Outcome’ is now reserved for referring to the analyses. It also fitted the ‘key words’ more closely. |
| 46. | Sixth, the paper is very / too long, dealing with a wide range of issues. I'd recommend a shorter paper with a narrower scope and fewer and more target objectives, potentially dividing this paper into more than one publication.  What is the key research interest? Is it about the "interaction" with socioeconomic deprivation"? Is it about the narrowing attainment gap over time? Is it about the impact of SEND provision? | We agree that accounting for the complexity of the situation (to the best of our ability) has lengthened the paper. We aimed to explore the contribution of major congenital anomalies to educational outcomes, in relation to other determinants of health and wellbeing. Reviewers’ insightful ideas for increased exploration of the data have, inevitably, lengthened the paper.  Most editors discourage “salami slicing” of data sets, and politicians say they prefer to have everything in a single document. The approach recommended by the reviewers has generated many supplementary analyses to support our key findings.  Unfortunately, the attainment gaps are widening.  Our priority is to identify the children in greatest need, so that services can be targeted. Currently, all school governors in Wales are being consulted on a revised funding formula for schools. The best way to inform this consultation is to offer a unified analysis of the relative contributions of identifiable risk factors. |
| 47. | Seventh, I understand that this is a complete-case analysis with all the associated risk of bias. Why didn't the authors explore other approaches to handle missing data? | Thank you. Our outcome data are restricted to children in mainstream schools in Wales. There were <5% missing cases for the other variables (now clarified in table S2). In the main, prescription data are missing from one geographical area that borders England. Accordingly, imputation from other areas might be inappropriate.  We have added to limitations line 583: Missing data were largely confined to the outcome variable and missing data for other variables were no more than 5% (Dettori et al 2018). Since imputation may underestimate variability whilst never fully compensating for missing data in cohort studies (Li et al 2024), no imputation analyses were undertaken. This complete case analysis is not transferable to children outside mainstream education. |
| 48. | Eighth, certain parts of the Discussion were not always easy to follow. | Thank you. The discussion has been redrafted. We hope that any obfuscations have been addressed. |
| 49. | Ninth, I'd welcome conclusions and implication for practice that are more tightly linked to and guided by the presented results. For example, the last sentence of the Discussion ("A unified, integrated, holistic, knowledgeable, expert service prioritising these most vulnerable children through to GCSEs, adulthood and employment,[46, 135, 137, 138] would pre-empt poverty, isolation and disability interacting to generate a profoundly disadvantaged subgroup.") could have been written without knowing the results of this paper. | Thank you. I should have explained the context of our recommendation. Currently, provision is split between 3 services, each with their own administrative procedures. Neither triaging nor a unified service exist, and, given the evidence presented, should. We have re-written:  Achieving the goals of ‘no child left behind (2020), no community left behind’ will entail identifying and ***triaging*** children at risk from compounded socio-economic and biological disadvantage, ensuring they are not overlooked in service re-organisation (Welsh Parliament 2024), and expediting intervention (Lee et al 2024).  And  Would transcending tripartite provision from education, social and health services, with a unified, holistic, knowledgeable, expert service to target these most vulnerable children through school to employment, (Fleming et al., 2019, Jay and Gilbert, 2021, Little and Saunders, 2015, Morgan, 2024) pre-empt poverty, isolation and disability interacting to generate a profoundly disadvantaged subgroup? |

## References

Damkjaer M, Urhoj SK, Tan J, Briggs G, Loane M, Given JE, Barrachina-Bonet L, Cavero-Carbonell C, Coi A, Neville AJ, Heino A, Kiuru-Kuhlefelt S, Jordan S, Scanlon I, Pierini A, Puccini A, Garne E, Morris JK. Prescription of cardiovascular medication in children with congenital heart defects across six European Regions from 2000 to 2014: data from the EUROlinkCAT population-based cohort study. BMJ Open. 2022 Apr 21;12(4):e057400. doi: 10.1136/bmjopen-2021-057400. PMID: 35450908.

Dettori JR, Norvell DC, Chapman JR. The Sin of Missing Data: Is All Forgiven by Way of Imputation? Global Spine J. 2018 Dec;8(8):892-894. doi: 10.1177/2192568218811922. Epub 2018 Dec 3. PMID: 30560043; PMCID: PMC6293424.

Divin N, Given JE, Tan J, Astolfi G, Ballardini E, Barrachina-Bonet L, Cavero-Carbonell C, Coi A, Garne E, Gissler M, Heino A, Jordan S, Pierini A, Scanlon I, Urhøj SK, Morris JK, Loane M. Antiasthmatic prescriptions in children with and without congenital anomalies: a population-based study. BMJ Open. 2023 Oct 13;13(10):e068885. doi: 10.1136/bmjopen-2022-068885. PMID: 37832979.

Donaldson G 2015 Successful Futures Independent Review of Curriculum and Assessment Arrangements in Wales. WG, <https://www.gov.wales/sites/default/files/publications/2018-03/successful-futures.pdf>

Given J, Morris JK, Garne E, Ballardini E, Barrachina-Bonet L, Cavero-Carbonell C, Gissler M, Gorini F, Heino A, Jordan S, Neville AJ, Pierini A, Scanlon I, Tan J, Urhoj SK, Loane M. Prescriptions for insulin and insulin analogues in children with and without major congenital anomalies: a data linkage cohort study across six European regions. Eur J Pediatr. 2023 Mar 4. doi: 10.1007/s00431-023-04885-6. Epub ahead of print. PMID: 36869270.

Hall KC, Robinson JC, Karimi M, Sung JH. Effect of Maternal Race, Residential Rurality, and Social Vulnerability on Critical Congenital Heart Defect Risk. Pediatr Cardiol. 2024 Apr 9. doi: 10.1007/s00246-024-03472-2. Epub ahead of print. PMID: 38592473.

Jennings AC, Rutherford MJ, Lambert PC. A Spline-Based Approach to Smoothly Constrain Hazard Ratios With a View to Apply Treatment Effect Waning. Stat Med. 2025 Mar 15;44(6):e70035. doi: 10.1002/sim.70035. PMID: 40059376; PMCID: PMC11891414.

Joshi H & Hawkes D 2006 Early and Late Entry to Motherhood in Wales: Evidence of Socio-economic Inequalities in the First Survey of the UK Millennium Cohort REPORT TO THE WELSH ASSEMBLY GOVERNMENT Centre for Longitudinal Research, Institute of Education, University of London. Available: <https://www.gov.wales/sites/default/files/statistics-and-research/2018-12/060524-entry-motherhood-en.pdf> accessed 19.8.25

Kinsner-Ovaskainen, A., Perraud, A., Barisic, I., Bergman, J.E. and Garne, E., EUROCAT Guide 1.5 - Instructions for the registration of congenital anomalies - Updated Version - August 2025, European Commission, Brussels, 2025, JRC143499

<https://eu-rd-platform.jrc.ec.europa.eu/system/files/public/eurocat/EUROCAT_Guide_1.5.pdf>

Li, J., Guo, S., Ma, R. *et al.* Comparison of the effects of imputation methods for missing data in predictive modelling of cohort study datasets. *BMC Med Res Methodol* 24, 41 (2024). https://doi.org/10.1186/s12874-024-02173-x

Miao Q, Dunn S, Wen SW, Lougheed J, Reszel J, Lavin Venegas C, Walker M. Neighbourhood maternal socioeconomic status indicators and risk of congenital heart disease. BMC Pregnancy Childbirth. 2021 Jan 21;21(1):72. doi: 10.1186/s12884-020-03512-8. PMID: 33478420; PMCID: PMC7819193.

MORRIS, J. K., GARNE, E., LOANE, M., BARIŠIĆ, I., DENSEM, J., LATOS-BIELEŃSKA, A., NEVILLE, A., PIERINI, A., RANKIN, J., DE WALLE, H., TAN, J. & CRARIDGE, H. 2021. EUROlinkCAT protocol for a European population-based data linkage study investigating the survival, morbidity and education of children with congenital anomalies. *BMJ Open,* 11**,** e047859.

HS digital 2024 NCARDRS Congenital Anomaly Official Statistics Report, 2021 chapter 4.3 <https://digital.nhs.uk/data-and-information/publications/statistical/ncardrs-congenital-anomaly-statistics-annual-data/ncardrs-congenital-anomaly-statistics-report-2021/deprivation>

NHS Digital NHS outcomes framework indicators - march 2022 release; 2.3.Ii unplanned hospitalisation for Asthma, diabetes and epilepsy in under 19s. 2022. [https://digital.nhs.uk/data-and-information/publications/statistical/nhs-outcomes-framework/march-2022/domain-2---enhancing-quality-of-life-for-people-with-long-term-conditions-nof/2.3.ii-unplanned-hospitalisation-for-asthma-diabetes-and-epilepsy-in-unde](https://digital.nhs.uk/data-and-information/publications/statistical/nhs-outcomes-framework/march-2022/domain-2---enhancing-quality-of-life-for-people-with-long-term-conditions-nof/2.3.ii-unplanned-hospitalisation-for-asthma-diabetes-and-epilepsy-in-under-19s)

Schisterman EF, Cole SR, Platt RW. Overadjustment bias and unnecessary adjustment in epidemiologic studies. Epidemiology. 2009 Jul;20(4):488-95. doi: 10.1097/EDE.0b013e3181a819a1. PMID: 19525685; PMCID: PMC2744485.

Seeger JD, Williams PL, Walker AM. An application of propensity score matching using claims data. Pharmacoepidemiol Drug Saf. 2005 Jul;14(7):465-76. doi: 10.1002/pds.1062. PMID: 15651087.

Thomson K, Moffat M, Arisa O*, et al* Socioeconomic inequalities and adverse pregnancy outcomes in the UK and Republic of Ireland: a systematic review and meta-analysis *BMJ Open* 2021;11:e042753. doi: 10.1136/bmjopen-2020-042753

Townsend P. Deprivation. *Journal of Social Policy*. 1987;16(2):125-146. doi:10.1017/S0047279400020341

Valabhji J, Barron E, Pratt A, Hafezparast N, Dunbar-Rees R, Turner EB, Roberts K, Mathews J, Deegan R, Cornelius V, Pickles J, Wainman G, Bakhai C, Johnston DG, Gregg EW, Khunti K. Prevalence of multiple long-term conditions (multimorbidity) in England: a whole population study of over 60 million people. J R Soc Med. 2024 Mar;117(3):104-117. doi: 10.1177/01410768231206033. Epub 2023 Oct 31. PMID: 37905525; PMCID: PMC11046366.

Welsh Government 2021 The Additional Learning Needs Code and regulations: explanatory memorandum. available: <https://www.gov.wales/the-additional-learning-needs-code-and-regulations>

WHO/CDC/ICBDSR. 2014 Birth defects surveillance: a manual for programme managers. Geneva: World Health Organization; 2014.

Yang J, Carmichael SL, Canfield M, Song J, Shaw GM; National Birth Defects Prevention Study. Socioeconomic status in relation to selected birth defects in a large multicentered US case-control study. Am J Epidemiol. 2008 Jan 15;167(2):145-54. doi: 10.1093/aje/kwm283. Epub 2007 Oct 17. PMID: 17947220.

**Bibliography**

Han Y, Li S, Song Y, Sun J, Yan W, Wang J, Gao X, Li X, Ren C, Zhao Q. Risk factors for infant hearing loss: a meta-analysis. Eur J Pediatr. 2024 May;183(5):2401-2409. doi: 10.1007/s00431-024-05498-3. Epub 2024 Mar 8. PMID: 38456989.

Jain A, Boyd NK, Paulsen KC, Vogel BN, Nguyen L, Santoro JD. Ophthalmologic and neuro-ophthalmologic findings in children with Down syndrome. Am J Med Genet C Semin Med Genet. 2023 Dec;193(4):e32068. doi: 10.1002/ajmg.c.32068. Epub 2023 Oct 4. PMID: 37794641.

Welsh Government 2025 [**Budgeted expenditure on Special Educational Needs (SEN) and Additional Learning Needs (ALN) provision: April 2025 to March 2026**](https://www.gov.wales/budgeted-expenditure-special-educational-needs-sen-and-additional-learning-needs-aln-provision-april-2025-to-march-2026) available <https://www.gov.wales/budgeted-expenditure-special-educational-needs-sen-and-additional-learning-needs-aln-provision-april-2025-to-march-2026-html>
