# Supplementary material for: Associations of major congenital anomalies, long-term health conditions, and socioeconomic deprivation with educational attainment in Wales: A population-based data linkage study
Source: PLoS Med. 2026 Oct 16;23(10):e1005050. doi: 10.1371/journal.pmed.1005050 (PMC13705610; doi:10.1371/journal.pmed.1005050)
Supplement: Supplementary file 6 [file pmed.1005050.s009.docx]

Responses to reviewers plosmed

PMEDICINE-D-26-00859R1 Decision - [EMID:48257a31ed57ece5]

"The children left behind – the cumulative impact of congenital anomalies, long-term conditions and poverty on educational attainment in Wales: a population databank linkage study" (PMEDICINE-D-26-00859R1) to PLOS Medicine.

We ask that you submit your revision by Jul 09 2026 11:59PM. However, if this deadline is not feasible, please contact me by email, and we can discuss a suitable alternative.

Don't hesitate to contact me directly with any questions ([lgaynor@plos.org](mailto:lgaynor@plos.org)).

Best regards,3

Louise

Louise Gaynor-Brook, MBBS PhD

Senior Editor

PLOS Medicine

[lgaynor@plos.org](mailto:lgaynor@plos.org)

-----------------------------------------------------------

Dear Louise,

Thank you for the opportunity to revise our paper. We are grateful for the support and insights of the reviewers.

Our ‘point by point’ responses are below.

Yours,

Sue

## Comments from the academic editor:

| The aim of the study is clear in title but incomplete in abstract and end of introduction. | Thank you. We have endeavoured to clarify by adding the factors accounted to the statement of the aim.  Abstract  We investigated factors affecting educational attainment of children with and without major congenital anomalies in state-funded mainstream schools at ages 11 and 16 (key stages [KS] 2 and 4), accounting for maternal and child deprivation, medicated co-morbidities, hospitalisation, gestation length, child sex, year of birth, and special education needs (SEN) provision.  End of introduction:  We aimed to explore determinants of educational attainment of children with and without major congenital anomalies attending state-funded mainstream schools in Wales at the end of primary and secondary schooling, at ages 11 (KS2) and 16 (KS4) [55,56], accounting for deprivation (FSM eligibility, income, environment, access to services), hospitalisation, medicated co-morbidities (asthma, epilepsy, diabetes) sex, preterm birth, year of birth. and SEN provision. |
| --- | --- |
| Summary in beginning of discussion could be improved. | Thank you. We hope this provides a clear summary of the paper. please let us know if it should be revised.  The children least likely to succeed lived in poverty with major congenital anomalies (Figs 4,6). Educational attainment at age 16 was more strongly associated with deprivation than anomalies or co-morbidities, when hospitalisation, sex, preterm birth, year of birth, and SEN provision were included in analyses. Accounting for SEN provision narrowed, but did not close, the attainment gap between children with and without anomalies (Tables 8, 9, S8). The attainment gap between children with and without SEN is narrowing in England [90], but not in Wales. Meanwhile, the disparity between those eligible and ineligible for FSM is increasing [61, 91]. |

## Comments from the reviewers:

| Comments | Responses |
| --- | --- |
| 1. Reviewer #1: Thank you for addressing my comments. While I do not agree hospitalisation should be a proxy for unmeasured confounder, I understand the rationale. However, such rationale should be clearly, and explicitly stated in the Methods, when you described hospitalisation as a confounder. Please specify what these variable were intended to represent as unmeasured confounders, ideally with a causal diagram to illustrate. | Thank you, we agree that this should be stated in the methods.  We have added line 248:  Days spent in hospital offers a proxy measure for illnesses other than those accounted; it represents a small proportion of school non-attendance.  We have added DAGs (S1, S2 figs) and text on mediators to our supplementary material, S1 appendix ‘Details of Methods’. |
|  |  |
| Reviewer #2: This revised version of the manuscript is greatly improved. A few questions and recommendations remain. | Thank you |
| 2. First, and especially for finalizing the paper for page proofs, this reviewer strongly urges that the authors use only a single decimal whenever a percent is reported (otherwise, with two decimals you are really providing a rate per 1,000, whereas the units for percents are per 100). Similarly, for odds ratios and confidence intervals, two decimal places are sufficient. Precision of estimate is not improved by reporting these statistics to three decimals. | Thank you for the expert advice. We have endeavoured to comply.  Where we quote external sources, we use the figures as given. |
| 3. Second, most of the analyses reported here appear to be for isolated birth defects. But this should be made clearer to the reader throughout. While it may be assumed that educational outcomes are poorer in children with one of these major defects in combination with other defects, that has not been analyzed here (unless I missed this in the methods). | Thank you. We have added to ‘limitations’ (line 672): The heterogeneous categories ‘all anomalies’ and ‘other anomalies’ include children with different degrees of disability and we were unable to explore these.  We explain:  Line 171  [65]. Children with major anomalies outside these categories were analysed as a single group, ‘other anomalies’, together with children with more than one anomaly.  Line 255  Congenital anomalies were analysed together, and, where numbers permitted, as isolated anomalies. |
| 4. Similarly, while there are data to identify which children are in special education placements, even more helpful would be data on specific developmental disabilities or conditions that warranted these placements. There's a substantial overlap between major birth defects and developmental disabilities*, and the analysis would be enriched by incorporating data on disabilities, which are likely available within the SAIL integrated data repository. But that is probably more for a separate analysis - here though the authors could discuss this as a potential limitation and area for further exploration. | We agree that these data would support our analysis. However, many disabilities (e.g. deafness, blindness) are not fully documented in children’s records. We acknowledge that we did not include ADHD and autism in our analyses. A separate analysis (Given et al 2025) indicates that these conditions may be under-reported. An unpublished paper by our group indicates that our electronic healthcare records fail to capture intellectual disability. We have added under limitations: (line 659)  Intellectual disability is poorly recorded in electronic records, and associations with anomalies, and multiple anomalies [156], could not be explored independently of SEN provision. |
| 5. Finally, while its unusual, perhaps its ok to end the section on implications, and the manuscript as a whole, with a question. But the ask implied in the final sentence is quite complex, and to some extent transcends what can be learned from the analyses presented here. | Thank you. The sentence has been reformatted to avoid a question. In our view, the data indicate a problem with current provision of sufficient magnitude to support others’ calls for intervention. |
| All in all, the authors are to be commended for a much improved resubmission. | Thank you for helping us. |
| 6. *Years ago this reviewer summarized the literature on co-occurring birth defects and developmental disabilities: Kirby RS. Co-occurrence of developmental disabilities with birth defects. Ment Retard Dev Disabil Res Rev. 2002;8(3):182-187. doi:10.1002/mrdd.10034 | Thank you. This seminal paper strengthens the referencing. |
| 7. Reviewer #3: Than you for giving me the opportunity to review this paper that looks at the effects of congenital abnormalities on educational attainment. The paper is well written and comprehensive, and the authors have answered the previous round of comments well. The use of the linked data that are available in SAIL allows for this important and novel analysis. This is a important topic and a well conducted study. | Thank you |
| However, I have a number of comments: |  |
| 8. Your title suggests that congenital abnormalities, poverty and long-term conditions are equally important, however, your analysis mainly focussed on congenital abnormalities. Consider changing the title to better reflect this? | We appreciate the reviewer’s suggestion. The title has been in place over several rounds of reviews, and this is the first prompt to change it.  Poverty and long-term conditions featured prominently in our analyses and are key to our findings. On balance, we feel that change at this stage might cause confusion with the existing preprint. |
| 9. You answered the comments about over-adjustment well. However, the predictive nature of your analysis and the need to avoid causal conclusions needs to be strongly highlighted in the discussion. | Thank you. We agree that observational work should not attribute causation.  We have checked the text for causal inference, and added (line 624). As with all observational research, no causation can be inferred. |
| 10. One of the issues that is highlighted by the comments is that interpreting ORs for multiple exposures from one model is problematic. I would consider separate models for each exposure. I think a really good example of where this is interpreting an ORs for poverty form table 10; a big issue is very few variables are confounders of the relationship between deprivation and educational outcomes, most factors offer the downstream of deprivation.   I think this is a really good example of why multiple DAGs would be so useful. | Thank you. Our supplementary material now presents unadjusted analyses and models without specified variables, such as SEN provision, year of birth. The S2 file contains sequential models where groups of variables are added in turn. Line 261: Covariates were added to models in four categories: child traits (year of birth, sex, gestational age), deprivation (FSM, ATS quintile, physical environment quintile, income quintile, mother’s deprivation quintile), medical interventions and co-morbidities (days in hospital, asthma, epilepsy, diabetes, cardiac conditions), and SEN provision (S2 file).  We have added (line 478):  Sequential addition of variables confirmed the impact of SEN on the association between anomalies and attainment (S2 file).  We agree, the interactions between inter-generational poverty, illness and educational attainment are complex, and most covariates are unlikely to be confounders.  Disability drives both attainment and poverty. Where there is a disabled child in a family, that family is more likely to fall into poverty, and the child’s educational attainment is further jeopardised.  We have now explored mediation for deprivation and birth, hospitalisation and SEN provision and added 2 DAGs to the supplementary material (S1 file p.2), whilst considering one reviewer’s advice to shorten the paper. |
| 11. I think the argument of including hospital stay and comorbidities as covariates remains problematic - they occur after congenital abnormalities so cannot be considered as potential confounders, they are likely mediators.   In fact a number of your covariates are not confounders.  SEN also a mediator - change the way you discuss results and ensure you don't use any causal language (e.g. potential confounders). | We agree, observational work cannot define causation, and now clarify, (line 624):  As with all observational research, no causation can be inferred.  We re-checked for causal language, and changed the sub-heading from ‘potential confounders’ to ‘covariates’.  We agree, and have clarified (line 215)  Covariates were added sequentially to4explanatory analysis to assess their influence and explore modifiable risk factors.  Due to concerns regarding shortening of the paper, we have explored mediation in S1 file. Only SEN provision meets the classical criteria for a mediator (Barron & Kenny 1986).  The origin of the comorbidities explored is often genetic or arises in early pregnancy, like anomalies e.g. eosinophilia or auto-immunity. They often arise independently of anomalies.    Hospitalisation occurs for reasons other than anomalies, in all children, and these are not otherwise accounted.  We agree and have added:  Line 270:  Hospitalisation, deprivation at birth and SEN provision were examined for mediation effects [87] (S1 file).  Line 379:  Inclusion of SEN provision in the regression models reduced, but did not eliminate, the associations between anomalies and lower achievement (Table 8, S2 file); SEN provision mediated the effect of anomalies on educational attainment.  The term “confounder” has been removed – thank you for this suggestion. |
| 12. Despite there being no association between IMD and congenital abnormalities, I feel you should still consider this as a mediator as these decisions should be made a priori rather than data driven | Thank you. As suggested we examined maternal deprivation as a potential mediator (S1 file).  We also found no significant association in our earlier work on this database (Jordan et al 2016), so were not expecting an association. The data are in S3 Table. To clarify, we have now cited this paper. Line 598:  The absence of overall association between WIMD at birth and congenital anomalies, as previously reported (Jordan et al 2016) runs counter to reports elsewhere [126-130]; this may be attributed to associations between maternal age and SES in Wales [131].  Adding deprivation to the model (S2 file) made little difference to the association between anomalies and educational attainment. We explore mediation in S1 file. |
| 13. Interactions require very large sample sizes, it is worth highlighting that despite a very large sample size in SAIL the study may still have been underpowered using the EUROCAT categories is fine. | Thank you. We have added (line 479):  Inclusion of interaction terms left findings essentially unchanged; confidence intervals widened slightly, but the limited sample size precluded further interpretation. |
| 14. I think the point about combining abnormalities is reasonable and should be highlighted in the limitations. | We agree and have added (line 619):  The heterogeneous ‘all anomalies’ and ‘other or multiple anomalies’ categories include children with a range of disabilities, and we were unable to explore individuals’ levels of disability. |
| 15. your justification for using the specific childhood conditions is good. However, this could potentially be strengthened by highlighting how these are related to perinatal issues. | Thank you.  We were unable to explore other perinatal issues, as our data on intensive care, breastfeeding and C sections are incomplete. Were we to obtain further funding, these, and other variables, could be explored in sensitivity analyses. We have acknowledged this problem. Line 655: Data on school attendance, social care interventions [149], ethnicity, breastfeeding [150], multiple births, autism, ADHD, mental health, cystic fibrosis, perinatal complications, maternal ill-health, medication use or substance misuse [151] were not included [65]. |
| 16. While you explain that a DAG was not included due to complexity Introduced by interactions, I think simply not including the interaction terms would still be beneficial 17. There is existing evidence of differential consequences across socioeconomic strata of negative perinatal outcomes; referencing this would strengthen your introduction - <https://eur03.safelinks.protection.outlook.com/?url=https%3A%2F%2Fbmjopen.bmj.com%2Fcontent%2Fbmjopen%2F15%2F1%2Fe084147.full.pdf&data=05%7C02%7Cs.e.jordan%40swansea.ac.uk%7C22be00fffc384d225c7f08decd20750e%7Cbbcab52e9fbe43d6a2f39f66c43df268%7C0%7C0%7C639173736454700759%7CUnknown%7CTWFpbGZsb3d8eyJFbXB0eU1hcGkiOnRydWUsIlYiOiIwLjAuMDAwMCIsIlAiOiJXaW4zMiIsIkFOIjoiTWFpbCIsIldUIjoyfQ%3D%3D%7C0%7C%7C%7C&sdata=bNaWxAWmlgAhhDGKqLbKN3KVhKfdQhCGEps0mH%2BhGOY%3D&reserved=0> | On advice from reviewers, we have prepared some tentative DAGs, as supplementary material, S1 fig, S2 fig.  We analysed with and without interaction terms (S2 file).  Thank you for raising this interesting point. We have reviewed our data, but differences appear relatively uniform across the quintiles. The largest difference is in the central quintile. We have added: (line 461)  Differences in attainment rates between children with and without anomalies ranged from 7.4% to 10.2%, with the largest difference in the central quintile (Fig 6) (S11 Table).  Attributing this to the relatively low GINI coefficient for Wales might be too speculative, and we are advised against lengthening the paper.  Thank you for highlighting this paper, which has stimulated a discussion of the discrepancies in findings regarding preterm birth. However, discussion has been curtailed by advice from other reviewers to shorten the text. We have added, line 574:  Associations between preterm birth and educational attainment [112] were not apparent when SEN provision was accounted; preterm birth frequently coincides with anomalies [113] (Tables 5, 10, S8). |
| 18. Your aims can be expanded on more - you've done more than you state In the background you mention that congenital abnormalities effect educational attainment indirectly through child health, is there not also a direct impact? | Thank you. We have expanded our aims:  We aimed to explore the educational attainment of children with and without major congenital anomalies attending state-funded mainstream schools in Wales at the end of primary and secondary schooling, at ages 11 (KS2) and 16 (KS4) [55,56], accounting for socio-economic status (SES) (as FSM eligibility, income, environment, access to services, and maternal deprivation), hospitalisation, medicated co-morbidities (asthma, epilepsy, diabetes) sex, preterm birth, year of birth and SEN provision.  We agree, and line 111 acknowledges that educational attainment may be affected by congenital anomalies. |
| 19. Only including mainstream is a limitation - this likely underestimates the effect of congenital abnormalities and the missing data pattern is missing not at random - ensure this is highlighted stronger 20. You mention that interactions are significant but 2 things - is it multiplicative or additive, ad what direction is the effects (exacerbate or attenuate) | We agree, and acknowledge this limitation; however, the data are not collected. We have included this in our ‘author summary’:  This analysis informs resource allocation in Wales: generalisation of findings to children outside mainstream education or to other countries rests with readers.  The models were additive, but interaction terms were assessed by including product terms in logistic regression models (i.e., representing multiplicative interactions on the odds-ratio scale). As so many interaction terms were examined across the outcomes, we think the detailed interpretation of each significant interaction cannot easily be communicated in the main text, but we agree with the importance of their inclusion. We have clarified that the that the odds ratios and confidence intervals for significant interactions are reported in Supplementary File S2, and added the direction of the effect to aid the reader.  Reviewer 2, above, indicates that our sample may be too small to interpret interaction terms. Very few interaction terms were statistically significant (S2 file).  We have written:  Inclusion of interaction terms left findings essentially unchanged; confidence intervals widened slightly, but the limited sample size precluded further interpretation.  We have now inserted information as to the direction of the associations. Line 481:  Most interaction terms including anomalies were not statistically significant, other than the positive effect of SEN school-support combined with anomalies for 5 GCSEs C–A* with EWMS, and negative effect of statementing combined with anomalies for any 5 GCSEs. The combined effect of SEN with FSM was (positively) significant at all assessments except 5 GCSEs C–A* with EWMS; SEN combined with income was positively significant for some outcomes. In the main, SEN combined with hospitalisation was not significant. Of the WIMD components, only income interacted with SEN, and none interacted with anomalies (S2 file). |
| Minor comments: |  |
| 21. Figure 1 legend is not in the manuscript 22. Line 141 - explain why you chose this end date 23. Line 242/243 - ks2 and ks4 should be the other way around 24. Line 273 - incorrect capitalisation of they 25. Line 482 - a-theoretical, incorrect '-' | We apologise, and thank you for identifying this omission. It has been rectified.  We have added ‘(earliest and latest data available)’ to line 144.  We explain under ‘limitations’ line 627:  Education assessments were disrupted during the COVID-19 pandemic, and, currently, there are no more recent education data available in SAIL. The KS2 teacher assessment has been reviewed, but there are no reasons to question their internal validity [136]; publication of KS2 results ceased in 2018/19 [137].  To make this change would introduce an error. Most children in the cohort at age 16 were in the cohort at age 11, but many children present at age 11 did not reach 16 by 2019 (end of data availability).  Thank you, the punctuation error is corrected.  I have removed the optional hyphen. |
| 26. Line 570-572 - rewrite as a little unclear at the moment 27. Could the descriptive tables be combined? | Thank you. We have rewritten:  We do not know whether this discrepancy is due to better case finding or worse air quality [146] in the more deprived communities [147] [148, 149], or more poverty [60, 142] or greater use of medicines in pregnancy [129, 150].  The existing tables are >1 page long. Combining these would generate tables longer than PLOS’s permitted length of 2 pages. We await editorial advice. |
| 28. Reviewer #4: Thank you for the opportunity to review this manuscript. The authors had conducted an important and highly informative piece of work, which will be of interest to clinicians and policymarkers in general. In reviewing this article, I was able to read through previous reviewers' comments and the authors' responses to these comments as well as the tracked changes made to original manuscript. These are my additional comments: | Thank you. |
| Introduction |  |
| 29. The authors described the prevalence rates of 20% of major anomalies. This figure seemed unexpectedly high and I was unable to identify support for it in the cited references. The prevalence rate of anomalies within the cohort studied were only about 3.5%. Please could the authors check and clarify. | We apologise. The text has been misunderstood. We have deleted the sentence; this complies with advice to shorten the text. We opted not to re-write to avoid any ambiguities.  The prevalence rates of some (approximately 20%) major anomalies, including congenital heart disease (CHD) and craniosynostosis, are increasing [11, 12]. |
| Methods |  |
| 30. - The selection of and inclusion of the medicated co-morbidities (epilepsy, diabetes, asthma) in the analyses should be better justified. I understood that the authors seek to explore the impact of co-morbidities on educational attainment. Nonetheless, as described in the results section line 292-293, that there is no association of congenital anomalies with diabetes. What is then the value of including diabetic prescriptions? | We agree that referencing on this could be strengthened, and have further supported the text regarding long-term conditions and educational outcomes (line 111):  ‘Although educational attainment may be affected by congenital anomalies or medicated long-term conditions (Wisk et al 2017, Koivusilta et al 2022), […]’  In Wales, digital databases and experience indicate that asthma, diabetes and epilepsy account for most childhood ill-health (reference 45).  At KS2, descriptive data indicate more children with anomalies than without have type 1 diabetes (Table 6). We explored this further in adjusted analyses. We are happy to report that children with type 1 diabetes are as successful as their contemporaries.  Ill-health affects school attendance and, according to the literature, may affect educational outcomes.  A systematic review suggests that type 1 diabetes may adversely affect school performance at 16 (Oakley et al 2020, reference 50); more recently, associations between childhood diabetes and school outcomes have been portrayed more negatively (Nouira et al 2024). We did not know before seeing the results that our findings are discordant with those from Sweden and Tunisia, and we report this. Line 567:  Adjusted models (including SEN provision) found no associations between medicated asthma [45-48] or diabetes [49, 50] and educational attainment.  The S2 file reports models with and without the 3 long-term conditions.  The association between diabetes and anomalies is complicated, but outside the scope of our study. We know that children of women with diabetes are more likely to have anomalies <https://www.ncbi.nlm.nih.gov/sites/books/NBK558974/>. They are also more likely to inherit diabetes <https://diabetes.org/about-diabetes/genetics-diabetes>. The extent to which these coincide is unclear. |
| 31. 584- Related to the above point, this particular sentence (line 119-120) in the introduction is confusing and needs rewording: "To include important medicated comorbidities (epilepsy, diabetes, asthma and cardiac conditions), the study was restricted to this portion of the national population." | Thank you. We have modified (line 153):  The study was restricted to this portion of the national population to account for medicated epilepsy, asthma and diabetes.  In practice, this restriction lost ~1% of the population. (S3 table) |
| 32. - Potential confounders (line 179): please specify what perinatal factors were included as covariates. | Thank you, we have specified: preterm birth and year of birth |
| 33. - Line 135-136, Down syndrome is missing from the text | Thank you, the omission has been corrected. |
| Results |  |
| 34. - The sentence 'Children receiving SEN provision were less successful' in line 332 needs expanding on. Apart from being an intervention, SEN provision may be a marker of disease severity. | We agree that SEN provision level may be a marker for severity of educational need, and reflects perceived needs. We have clarified in line 238:  SEN provision recorded in the year of assessment was taken as a marker for the perceived severity of a child’s difficulties  We have expanded line 387 (was 332):  Children receiving SEN provision were less successful, reflecting their additional needs. |
| 35. - FSM eligibility is a measure for deprivation. Whilst I understand the authors' intention to highlight the impact of both SEN and FSM, the paragraph from line 332 would seem better placed after the results describing the educational attainment of children with and without congenital anomalies. | Thank you for this insight. We have now moved the section on SEN & anomalies to follow the description of the cohort. This table (now table 7) illustrates the distribution of SEN provision. Tables S6a, S6b, and S14 give the unadjusted results for FSM and attainment. |
| 36. - Additionally, the results will be more informative if Table 8 is displayed stratifying the cohort into: (i) congenital anomalies with FSM, (ii) congenital anomalies without FSM, (iii) no anomalies with FSM, (iv) no anomalies without FSM. | Thank you. The stratified data are available in S6a and S6b tables, and figures 4 & 5. Table 9 is designed to illustrate the differences between the risk factors: anomalies and FSM. We have clarified, line 403:  Of the children with anomalies and FSM eligibility, 2.60% (5/192) receiving SEN statements, and 34.64% (115/332) without SEN provision attained 5 GCSEs C-A* with EWMS. Children with anomalies ineligible for FSM were two to four times more likely to be successful: 10.45% (37/354) of those receiving statements and 67.45% (1628/2413) of those not receiving SEN provision achieved these GCSEs (Fig. 4, S6a Table). |
| 37. - Similarly Figure 3 should display include the same information for children without anomalies. | Thank you for this suggestion. We have added this as fig 5, and S6b table. |
| Discussion |  |
| 38. - Would children with genetic diagnoses associated congenital anomalies other than chromosomal anomalies be captured within the dataset? It is likely that some of these diagnoses may be associated with intellectual disability, which if not diagnosed, will be misclassified into 'isolated anomalies'. The burden of intellectual disability is then captured in the need for SEN. | Yes, thank you, we have clarified as a note to Table 4:  *Children with non-chromosomal genetic conditions are captured in the dataset and are included in the “ALL ANOMALIES” subgroup. This includes all genetic conditions with an ICD10 code beginning with Q and Di George syndrome (ICD10 code D821). The EUROCAT syndrome guide lists syndromes included:* [*https://eu-rd-platform.jrc.ec.europa.eu/sites/default/files/EUROCAT%20Syndrome%20Guide%20Revision%20Final%20version%20September%202017.pdf*](https://eur03.safelinks.protection.outlook.com/?url=https%3A%2F%2Feu-rd-platform.jrc.ec.europa.eu%2Fsites%2Fdefault%2Ffiles%2FEUROCAT%2520Syndrome%2520Guide%2520Revision%2520Final%2520version%2520September%25202017.pdf&data=05%7C02%7CS.E.Jordan%40Swansea.ac.uk%7Ca9f1f6dce9f14b86d23f08ded29a094a%7Cbbcab52e9fbe43d6a2f39f66c43df268%7C0%7C0%7C639179756485574318%7CUnknown%7CTWFpbGZsb3d8eyJFbXB0eU1hcGkiOnRydWUsIlYiOiIwLjAuMDAwMCIsIlAiOiJXaW4zMiIsIkFOIjoiTWFpbCIsIldUIjoyfQ%3D%3D%7C0%7C%7C%7C&sdata=MI91lU4rBSzPJXAzzd0D5Vr%2B6Zu0nB2iym7TFjBzgfQ%3D&reserved=0)*. It is possible that some syndromes may be undiagnosed and therefore misclassified as ‘isolated’ anomalies; however, EUROCAT includes all anomalies diagnosed and reported in infancy or prenatally, including those commonly associated with structural malformations and/or found in the Q-chapter of ICD10/BPA.* |
| 39. Overall the manuscript will benefit from being shortened and language editing to fix typos and grammatical/ punctuation errors. | We apologise for errors, and will correct any identified. The automated checker does not indicate errors. Addressing the excellent points made by all reviewers have precluded significant shortening.  We await editorial advice regarding moving table 1 and some of the text on methods to supplementary material. |

Any attachments provided with reviews can be seen via the following link:

**Not seen**

--------------------------------------------------------- ---

## General editorial requests

**Please be sure to check the general editorial comments and include these in your point-by-point response.**

(Note: not all will apply to your paper, but please check each item carefully)

|  | Responses |
| --- | --- |
| 40. * We ask every co-author listed on the manuscript to fill in a contributing author statement, making sure to declare all competing interests. If any of the co-authors have not filled in the statement, we will remind them to do so when the paper is revised. If all statements are not completed in a timely fashion this could hold up the re-review process. If new competing interests are declared later in the revision process, this may also hold up the submission. Should there be a problem getting one of your co-authors to fill in a statement we will be in contact. Please do not add or remove authors without first discussing this with the handling editor. You can see our competing interests policy here: <https://eur03.safelinks.protection.outlook.com/?url=http%3A%2F%2Fjournals.plos.org%2Fplosmedicine%2Fs%2Fcompeting-interests&data=05%7C02%7Cs.e.jordan%40swansea.ac.uk%7C22be00fffc384d225c7f08decd20750e%7Cbbcab52e9fbe43d6a2f39f66c43df268%7C0%7C0%7C639173736454764229%7CUnknown%7CTWFpbGZsb3d8eyJFbXB0eU1hcGkiOnRydWUsIlYiOiIwLjAuMDAwMCIsIlAiOiJXaW4zMiIsIkFOIjoiTWFpbCIsIldUIjoyfQ%3D%3D%7C0%7C%7C%7C&sdata=EOvcwOYT4HCsiEz1er%2BTkOtZtu0KL1c9p8kvu9y2ut4%3D&reserved=0>. | We are sorry, but we are unable to locate the form. |
|  |  |
| 41. * Please upload any figures associated with your paper as individual TIF or EPS files with 300dpi resolution at resubmission; please read our figure guidelines for more information on our requirements: <https://eur03.safelinks.protection.outlook.com/?url=http%3A%2F%2Fjournals.plos.org%2Fplosmedicine%2Fs%2Ffigures&data=05%7C02%7Cs.e.jordan%40swansea.ac.uk%7C22be00fffc384d225c7f08decd20750e%7Cbbcab52e9fbe43d6a2f39f66c43df268%7C0%7C0%7C639173736454827594%7CUnknown%7CTWFpbGZsb3d8eyJFbXB0eU1hcGkiOnRydWUsIlYiOiIwLjAuMDAwMCIsIlAiOiJXaW4zMiIsIkFOIjoiTWFpbCIsIldUIjoyfQ%3D%3D%7C0%7C%7C%7C&sdata=xaP%2BK86UtfwqdVxx8jy7ydG%2B26lmXJxk6V%2B%2F4JtDJuU%3D&reserved=0>. While revising your submission, we strongly recommend that you use PLOS's NAAS tool (<https://eur03.safelinks.protection.outlook.com/?url=https%3A%2F%2Fngplosjournals.pagemajik.ai%2Fartanalysis&data=05%7C02%7Cs.e.jordan%40swansea.ac.uk%7C22be00fffc384d225c7f08decd20750e%7Cbbcab52e9fbe43d6a2f39f66c43df268%7C0%7C0%7C639173736454885488%7CUnknown%7CTWFpbGZsb3d8eyJFbXB0eU1hcGkiOnRydWUsIlYiOiIwLjAuMDAwMCIsIlAiOiJXaW4zMiIsIkFOIjoiTWFpbCIsIldUIjoyfQ%3D%3D%7C0%7C%7C%7C&sdata=cJ47HZSY9C6P3ZMYRIpXGxnJr8n8tmuU7mANH%2FnS%2BRs%3D&reserved=0>) to test your figure files. NAAS can convert your figure files to the TIFF file type and meet basic requirements (such as print size, resolution), or provide you with a report on issues that do not meet our requirements and that NAAS cannot fix. | All figures have been tested and put through PLOS’s NAAS tool. As such, all figures should meet the requirements. |
|  |  |
| 42. After uploading your figures to PLOS's NAAS tool - <https://eur03.safelinks.protection.outlook.com/?url=https%3A%2F%2Fngplosjournals.pagemajik.ai%2Fartanalysis&data=05%7C02%7Cs.e.jordan%40swansea.ac.uk%7C22be00fffc384d225c7f08decd20750e%7Cbbcab52e9fbe43d6a2f39f66c43df268%7C0%7C0%7C639173736454939007%7CUnknown%7CTWFpbGZsb3d8eyJFbXB0eU1hcGkiOnRydWUsIlYiOiIwLjAuMDAwMCIsIlAiOiJXaW4zMiIsIkFOIjoiTWFpbCIsIldUIjoyfQ%3D%3D%7C0%7C%7C%7C&sdata=kkG47J%2BpKHJaklAqd%2F07vopZ3z8E2YzjprZIjt8VzUM%3D&reserved=0>, NAAS will process the files provided and display the results in the "Uploaded Files" section of the page as the processing is complete. | All figures have been tested and put through PLOS’s NAAS tool. As such, all figures should meet the requirements. |
| 43. If the uploaded figures meet our requirements (or NAAS is able to fix the files to meet our requirements), the figure will be marked as "fixed" above. If NAAS is unable to fix the files, a red "failed" label will appear above. | All figures have been tested and put through PLOS’s NAAS tool. As such, all figures should meet the requirements. |
| 44. When NAAS has confirmed that the figure files meet our requirements, please download the file via the download option, and include these NAAS processed figure files when submitting your revised manuscript. | All figures have been tested and put through PLOS’s NAAS tool. As such, all figures should meet the requirements. |
| 45. * Please ensure that the paper adheres to the PLOS Data Availability Policy (see <https://eur03.safelinks.protection.outlook.com/?url=http%3A%2F%2Fjournals.plos.org%2Fplosmedicine%2Fs%2Fdata-availability&data=05%7C02%7Cs.e.jordan%40swansea.ac.uk%7C22be00fffc384d225c7f08decd20750e%7Cbbcab52e9fbe43d6a2f39f66c43df268%7C0%7C0%7C639173736454991344%7CUnknown%7CTWFpbGZsb3d8eyJFbXB0eU1hcGkiOnRydWUsIlYiOiIwLjAuMDAwMCIsIlAiOiJXaW4zMiIsIkFOIjoiTWFpbCIsIldUIjoyfQ%3D%3D%7C0%7C%7C%7C&sdata=oNPkm5WLF1%2Ft56oDiGuosRphgFtjB%2FFYpU71H4qQB7s%3D&reserved=0>), which requires that all data underlying the study's findings be provided in a repository or as Supporting Information. For data residing with a third party, authors are required to provide instructions with contact information (web or email address) for obtaining the data. Please note that a study author cannot be the contact person for the data. PLOS journals do not allow statements supported by "data not shown" or "unpublished results." For such statements, authors must provide supporting data or cite public sources that include it. | We have added a Data Availability statement. |
| 46. * We expect all researchers with submissions to PLOS in which author-generated code underpins the findings in the manuscript to make all author-generated code available without restrictions upon publication of the work. In cases where code is central to the manuscript, we require the code to be made available as a condition of publication. Authors are responsible for ensuring that the code is reusable and well documented. Please make any custom code available, either as part of your data deposition or as a supplementary file. Please add a sentence to your data availability statement regarding any code used in the study, e.g. "The code used in the analysis is available from Github [URL] and archived in Zenodo [DOI link]" Please review our guidelines at <https://eur03.safelinks.protection.outlook.com/?url=https%3A%2F%2Fjournals.plos.org%2Fplosmedicine%2Fs%2Fmaterials-software-and-code-sharing&data=05%7C02%7Cs.e.jordan%40swansea.ac.uk%7C22be00fffc384d225c7f08decd20750e%7Cbbcab52e9fbe43d6a2f39f66c43df268%7C0%7C0%7C639173736455047624%7CUnknown%7CTWFpbGZsb3d8eyJFbXB0eU1hcGkiOnRydWUsIlYiOiIwLjAuMDAwMCIsIlAiOiJXaW4zMiIsIkFOIjoiTWFpbCIsIldUIjoyfQ%3D%3D%7C0%7C%7C%7C&sdata=pcFZGrmZdxSyA%2FUbI74b8DEKl5BRJl%2FmFDFsRZgB9WI%3D&reserved=0> and ensure that your code is shared in a way that follows best practice and facilitates reproducibility and reuse. Because Github depositions can be readily changed or deleted, we encourage you to make a permanent DOI'd copy (e.g. in Zenodo) and provide the URL. | The Data Availability statement has been added. |
|  |  |
| 47. * Please ensure that the study is reported according to the RECORD guideline and include the completed checklist as Supporting Information. When completing the checklist, please use section and paragraph numbers, rather than page numbers. Please add the following statement, or similar, to the Methods: "This study is reported as per the REporting of studies Conducted using Observational Routinely-collected Data (RECORD) guideline (S1 Checklist)." | Text has been added and RECORD statement updated. |
| FORMATTING – GENERAL |  |
| 48. * Abstract: Please structure your abstract using the PLOS Medicine headings (Background, Methods and Findings, Conclusions). Please combine the Methods and Findings sections into one section. | The abstract has this required structure and the mentioned PLOS Medicine headings. |
|  |  |
| 49. * At this stage, we ask that you include a short, non-technical Author Summary of your research to make findings accessible to a wide audience that includes both scientists and non-scientists. The Author Summary should immediately follow the Abstract in your revised manuscript. This text is subject to editorial change and should be distinct from the scientific abstract. Ideally each sub-heading should contain 2-3 single sentence, concise bullet points containing the most salient points from your study. In the final bullet point of 'What Do These Findings Mean?', please include the main limitations of the study in non-technical language. Please see our author guidelines for more information: <https://eur03.safelinks.protection.outlook.com/?url=https%3A%2F%2Fjournals.plos.org%2Fplosmedicine%2Fs%2Frevising-your-manuscript%23loc-author-summary&data=05%7C02%7Cs.e.jordan%40swansea.ac.uk%7C22be00fffc384d225c7f08decd20750e%7Cbbcab52e9fbe43d6a2f39f66c43df268%7C0%7C0%7C639173736455106464%7CUnknown%7CTWFpbGZsb3d8eyJFbXB0eU1hcGkiOnRydWUsIlYiOiIwLjAuMDAwMCIsIlAiOiJXaW4zMiIsIkFOIjoiTWFpbCIsIldUIjoyfQ%3D%3D%7C0%7C%7C%7C&sdata=rxd7J%2BUCn34zeL5xxrK%2FQQrBrqs0qcppv3xLyBaVO1w%3D&reserved=0>. | An Author Summary has been included. This consists of a few bullet points for each of the questions/sub-headings as requested and outlined in the author guidelines. |
| 50. * Please express the main results with 95% CIs as well as p values. When reporting p values please report as p<0.001 and where higher as the exact p value p=0.002, for example. Throughout, suggest reporting statistical information as follows to improve clarity for the reader "22% (95% CI [13%,28%]; p</=)". Please be sure to define all numerical values at first use. | We give ORs and 95% CIs as per contemporary recommendations. |
| 51. * Please include page numbers and line numbers in the manuscript file. Use continuous line numbers (do not restart the numbering on each page). | Done |
| 52. * Please cite the reference numbers in square brackets. Citations should precede punctuation. | As stated, reference numbers are formatted in square brackets and precede punctuation where relevant. |
| FIGURES AND TABLES |  |
| 53. * Please provide titles and legends for all figures and tables (including those in Supporting Information files). | Titles and legends are provided for all figures and tables. |
| 54. * Please define all abbreviations used in each figure/table (including those in Supporting Information files). | Abbreviations used in the figures and titles have been defined. |
| 55. * Please consider avoiding the use of red and green in order to make your figure more accessible to those with color blindness. | Green and red colours have not been used in the manuscript to aid with accessibility. |
| SUPPLEMENTARY MATERIAL |  |
| 56. * Please note that supplementary material will be posted as supplied by the authors. Therefore, please amend it according to the relevant comments outlined here. |  |
| 57. * Please cite your Supporting Information as outlined here: <https://eur03.safelinks.protection.outlook.com/?url=https%3A%2F%2Fjournals.plos.org%2Fplosmedicine%2Fs%2Fsupporting-information&data=05%7C02%7Cs.e.jordan%40swansea.ac.uk%7C22be00fffc384d225c7f08decd20750e%7Cbbcab52e9fbe43d6a2f39f66c43df268%7C0%7C0%7C639173736455174509%7CUnknown%7CTWFpbGZsb3d8eyJFbXB0eU1hcGkiOnRydWUsIlYiOiIwLjAuMDAwMCIsIlAiOiJXaW4zMiIsIkFOIjoiTWFpbCIsIldUIjoyfQ%3D%3D%7C0%7C%7C%7C&sdata=KmuZvfdgsocWhKxiKroMvoEytmJEjAV0UMUSQqop44g%3D&reserved=0> | We have complied. |
| REFERENCES |  |
| 58. * PLOS uses the numbered citation (citation-sequence) method and first six authors, et al. | We have complied. |
|  |  |
| 59. * Please ensure that journal name abbreviations match those found in the National Center for Biotechnology Information (NCBI) databases (<https://eur03.safelinks.protection.outlook.com/?url=http%3A%2F%2Fwww.ncbi.nlm.nih.gov%2Fnlmcatalog%2Fjournals&data=05%7C02%7Cs.e.jordan%40swansea.ac.uk%7C22be00fffc384d225c7f08decd20750e%7Cbbcab52e9fbe43d6a2f39f66c43df268%7C0%7C0%7C639173736455239739%7CUnknown%7CTWFpbGZsb3d8eyJFbXB0eU1hcGkiOnRydWUsIlYiOiIwLjAuMDAwMCIsIlAiOiJXaW4zMiIsIkFOIjoiTWFpbCIsIldUIjoyfQ%3D%3D%7C0%7C%7C%7C&sdata=PmlyUma4hs8weJAH9EUJQKgN3%2B20e9gPpIyh9DKB9VQ%3D&reserved=0>), and are appropriately formatted and capitalised. | We have complied. |
| 60. * Where website addresses are cited, please include the complete URL and specify the date of access (e.g. [accessed: 12/06/2023]). | Checked and updated |
| 61. * Please also see <https://eur03.safelinks.protection.outlook.com/?url=https%3A%2F%2Fjournals.plos.org%2Fplosmedicine%2Fs%2Fsubmission-guidelines%23loc-references&data=05%7C02%7Cs.e.jordan%40swansea.ac.uk%7C22be00fffc384d225c7f08decd20750e%7Cbbcab52e9fbe43d6a2f39f66c43df268%7C0%7C0%7C639173736455288668%7CUnknown%7CTWFpbGZsb3d8eyJFbXB0eU1hcGkiOnRydWUsIlYiOiIwLjAuMDAwMCIsIlAiOiJXaW4zMiIsIkFOIjoiTWFpbCIsIldUIjoyfQ%3D%3D%7C0%7C%7C%7C&sdata=zxPQy4MhSj3AaJaA6439u7Gvo890UHMgZkKKVNcGKaQ%3D&reserved=0> for further details on reference formatting. | We have endeavoured to comply. |
| STUDY TYPE-SPECIFIC REQUESTS - OBSERVATIONAL STUDIES |  |
| 62. * Abstract: Please include the study design, population and setting, number of participants, years during which the study took place (enrolment and follow up), length of follow up, and main outcome measures. | This has been done. |
| 63. * Please ensure that the study is reported according to the RECORD guideline (available from <https://eur03.safelinks.protection.outlook.com/?url=https%3A%2F%2Fwww.record-statement.org%2F&data=05%7C02%7Cs.e.jordan%40swansea.ac.uk%7C22be00fffc384d225c7f08decd20750e%7Cbbcab52e9fbe43d6a2f39f66c43df268%7C0%7C0%7C639173736455333976%7CUnknown%7CTWFpbGZsb3d8eyJFbXB0eU1hcGkiOnRydWUsIlYiOiIwLjAuMDAwMCIsIlAiOiJXaW4zMiIsIkFOIjoiTWFpbCIsIldUIjoyfQ%3D%3D%7C0%7C%7C%7C&sdata=Ob12pyR9e1ToZ6lKi0XZYkXDTdD0Ch8oZLRH9emPt50%3D&reserved=0>) and include the completed checklist as Supporting Information. Please add the following statement, or similar, to the Methods: "This study is reported as per the Reporting of Studies Conducted using Observational Routinely-Collected Data (RECORD) guideline (S1 Checklist)." When completing the checklist, please use section and paragraph numbers, rather than page numbers. | Statement is uploaded as ‘other material’. |
|  |  |
| 64. * For all observational studies, in the manuscript text, please indicate: (1) the specific hypotheses you intended to test, (2) the analytical methods by which you planned to test them, (3) the analyses you actually performed, and (4) when reported analyses differ from those that were planned, transparent explanations for differences that affect the reliability of the study's results. If a reported analysis was performed based on an interesting but unanticipated pattern in the data, please be clear that the analysis was data driven. | Done |
| 65. * Please state in the Methods section whether the study had a prospective protocol or analysis plan. If a prospective analysis plan (from your funding proposal, IRB or other ethics committee submission, study protocol, or other planning document written before analyzing the data) was used in designing the study, please include the relevant document(s) with your revised manuscript as a Supporting Information file to be published alongside your study and cite it in the Methods section. A legend for this file should be included at the end of your manuscript. If no such document exists, please make sure that the Methods section transparently describes when analyses were planned, and when/why any data-driven changes to analyses took place. Changes in the analysis, including those made in response to peer review comments, should be identified as such in the Methods section of the paper, with rationale. | The prospective protocol of this study was registered on the HMA-EMA Catalogue of real-world data studies [https://catalogues.ema.europa.eu/node/2803/study-documents](https://eur03.safelinks.protection.outlook.com/?url=https%3A%2F%2Fcatalogues.ema.europa.eu%2Fnode%2F2803%2Fstudy-documents&data=05%7C02%7CS.E.Jordan%40Swansea.ac.uk%7Ca9f1f6dce9f14b86d23f08ded29a094a%7Cbbcab52e9fbe43d6a2f39f66c43df268%7C0%7C0%7C639179756485629118%7CUnknown%7CTWFpbGZsb3d8eyJFbXB0eU1hcGkiOnRydWUsIlYiOiIwLjAuMDAwMCIsIlAiOiJXaW4zMiIsIkFOIjoiTWFpbCIsIldUIjoyfQ%3D%3D%7C0%7C%7C%7C&sdata=8QMXbsSlLVL0FikuZ%2F8fqkrXPx%2F6v08TtZrbsbP%2Bpy0%3D&reserved=0). |
| Staff team |  |
| 66. Thank you for including an Ethics Statement for your study. Please include (if human participants/donors only) - A statement that formal consent was obtained (must state whether verbal/written) OR the reason consent was not obtained (e.g. anonymity). NOTE: If child participants, the statement must declare that formal consent was obtained from the parent/guardian/head of household. | We apologise for the confusion. SAIL is an anonymised data linkage system. We have modified, line 278:  The EUROlinkCAT study was approved by SAIL’s Independent [Governance](https://www.sciencedirect.com/topics/social-sciences/governance) Review Panel (IGRP), which comprises information governance experts and patient and public representatives, on 27/1/2018 and 20/12/2022 (number 0511). This study design used only irrevocably anonymised, routinely collected data, and therefore was deemed to require neither written informed consent nor further ethical approval [66]. |
| 67. We have noticed that you have uploaded Supporting Information files (Fig S1, S2), but you have not included a list of legends. Please add a full list of legends for your Supporting Information files before or after the references list. | We apologise for this omission. A full list has been added the figure legends to the list of supporting information. |

### References

Baron RM, Kenny DA: The moderator–mediator variable distinction in social psychological research: Conceptual, strategic, and statistical considerations. Journal of Personality and Social Psychology 1986, 51(6):1173–1182. 10.1037/0022-3514.51.6.1173

Given J, Paoletti O, Bromley R, Ballardini E, Beau AB, Bartolini C, Caillet A, Coldea A, Damase-Michel C, Evans HT, Jordan S, Leinonen MK, Manfrini M, Martikainen V, Morris JK, Neville AJ, Nordeng H, Puccini A, Coste F, Loane M. The Effect of Different Algorithms on Prevalence of Attention Deficit Hyperactivity Disorder and Autism Spectrum Disorder in Secondary Healthcare Data in Five European Countries: A Contribution from the ConcePTION Project. J Autism Dev Disord. 2025 Aug 22. doi: 10.1007/s10803-025-06997-4. Epub ahead of print. PMID: 40844687.

Honein MA, Kirby RS, Meyer RE, Xing J, Skerrette NI, Yuskiv N, Marengo L, Petrini JR, Davidoff MJ, Mai CT, Druschel CM, Viner-Brown S, Sever LE; National Birth Defects Prevention Network. The association between major birth defects and preterm birth. Matern Child Health J. 2009 Mar;13(2):164-75. doi: 10.1007/s10995-008-0348-y. Epub 2008 May 17. PMID: 18484173; PMCID: PMC11669608.

Koivusilta L, Kaltiala R, Myöhänen A, Hotulainen R, Rimpelä A. A Chronic Disease in Adolescence and Selection to an Educational Path-A Longitudinal Study. Int J Environ Res Public Health. 2022 Nov 3;19(21):14407. doi: 10.3390/ijerph192114407. PMID: 36361283; PMCID: PMC9657637.

McHale P, Fahy K, Pennington A, Schlüter DK, Barr B, Taylor-Robinson D. How do socioeconomic inequalities and preterm birth interact to modify health and education outcomes? A narrative systematic review. BMJ Open. 2025 Jan 25;15(1):e084147. doi: 10.1136/bmjopen-2024-084147. PMID: 39863409; PMCID: PMC11784320.

Nouira S, Ach T, Ammar A, Ach M, Sabbagh G, Ezzi O, Benabdelkrim A. Prevalence and factors associated with school failure in children with type 1 diabetes. Pediatr Endocrinol Diabetes Metab. 2024;30(1):3-7. doi: 10.5114/pedm.2023.133124. PMID: 39026473; PMCID: PMC11037087.

Oakley NJ, Kneale D, Mann M, Hilliar M, Dayan C, Gregory JW, French R. Type 1 diabetes mellitus and educational attainment in childhood: a systematic review. BMJ Open. 2020 Jan 26;10(1):e033215. doi: 10.1136/bmjopen-2019-033215. PMID: 31988228; PMCID: PMC7045136.

Wisk LE, Weitzman ER. Expectancy and Achievement Gaps in Educational Attainment and Subsequent Adverse Health Effects Among Adolescents With and Without Chronic Medical Conditions. J Adolesc Health. 2017 Oct;61(4):461-470. doi: 10.1016/j.jadohealth.2017.04.006. Epub 2017 Jul 19. PMID: 28734632; PMCID: PMC5610930.
