# Supplementary material for: Associations of major congenital anomalies, long-term health conditions, and socioeconomic deprivation with educational attainment in Wales: A population-based data linkage study
Source: PLoS Med. 2026 Oct 16;23(10):e1005050. doi: 10.1371/journal.pmed.1005050 (PMC13705610; doi:10.1371/journal.pmed.1005050)
Supplement: Supplementary file 7 [file pmed.1005050.s010.docx]

Suzanne de Bruijn, PhD

Associate editor, PLOS Medicine

[sbruijn@plos.org](mailto:sbruijn@plos.org)

24.9.26

Dear Suzanne,

Thank you for reviewing our paper so assiduously. We have adopted all recommendations and responded, as tabulated below. Please let me know if any further amendments are needed.

Yours,

Sue

|  | Response |
| --- | --- |
| 1) Thank you for changing your title. I'm afraid that we have strict guidelines on our titles, so you will not be able to include the phrase "the children left behind' in the title. You're welcome to mention this elsewhere in the manuscript with reference to the landmark report, as you have done in the discussion. | Thank you, we quite understand. |
| 2) You provide data in the European DD/MM/YY format in both the abstract and in the paper. For clarity, can you please amend this to spell out the month? | We apologise, and have amended. |
| 3) Line 221: is this statement correct: “has achieved Level 1 (G–A*) or Level 2 (C–A*) in at least 5 GCSE subjects” or should level 1 be (G-D)? | Yes, the statement is correct. Children who achieve level 2 are also deemed to have achieved level 1. A note has been added to Table 2 to clarify:  Children who achieve level 2 are also deemed to have achieved level 1. |
| 4) Table 2: it seems that the qualification “Level 2 (C-A* in 5 GCSEs including EWMS)” is both a low-level and a high-level pass; is this correct? Or is this accidentally included twice? | Yes, this is correct. I hope the note to Table 2 will remove ambiguity.  Children who achieve level 2 are also deemed to have achieved level 1. |
| 5) Line 351: please remove ‘the burden of deprivation’. | The text has been removed. |
| 6) Table 7+8: it does not seem that you use the abbreviations UCL and LCL in these tables. If this is correct, please remove these from the notes, but include “CI” | We apologise for this oversight, and have corrected. |
| 7) Figure 6: please amend to state ‘congenital anomalies’ rather than ‘anomalies’ | Thank you. Fig 6 has been amended and resubmitted. |
| 8) Table 3: Please consider including more explanation in the table on the data you provide: e.g.: include 'n' and '%' in the header of the table, rather than just in the notes. | Thank you, the additional header has been included. |
| 9) Ethics (line 302): who deemed this to require no ethics approval? Was the study assessed by an IRB? Please provide some explanation. | SAIL’s Independent [Governance](https://www.sciencedirect.com/topics/social-sciences/governance) Review Panel (IGRP) makes these decisions, reference 66 has more detail.  We have expanded (line 304):  “The study used only irrevocably anonymised routinely collected data held within the SAIL Databank. Project 0511 was reviewed and approved by the SAIL independent Information Governance Review Panel (IGRP), which assesses whether the project is appropriate, legally compliant, and in the public interest. Separate ethical approval was not required as only anonymised data were used, within a secure trusted research environment.”  Our wording is similar to that used by earlier SAIL papers in PLOS Medicine [1]. |
| 10) you provide a governance review panel number in the acknowledgments, please add this data to the Ethics statement. | Our number (0511) is included in the ethics statement, lines 305 and 306. |
| 11) financial disclosure: there seem to be duplicate statements regarding the role funders played in this study in the metadata. Please amend if necessary. | We apologise for the repetition, and have corrected. |
|  |  |

1. Alsallakh MA, Rodgers SE, Lyons RA, Sheikh A, Davies GA (2021) Association of socioeconomic deprivation with asthma care, outcomes, and deaths in Wales: A 5-year national linked primary and secondary care cohort study. PLOS Medicine 18(2): e1003497. <https://doi.org/10.1371/journal.pmed.1003497>
