# Supplementary material for: Associations of major congenital anomalies, long-term health conditions, and socioeconomic deprivation with educational attainment in Wales: A population-based data linkage study
Source: PLoS Med. 2026 Oct 16;23(10):e1005050. doi: 10.1371/journal.pmed.1005050 (PMC13705610; doi:10.1371/journal.pmed.1005050)
Supplement: Peer review history

# Author comment: Author response to Decision Letter 0

**DOI:** 10.1371/journal.pmed.1005050.r001

13 Mar 2026

Attachment Submitted filename: pmed response.docx

# Editor report: Decision Letter 0

**DOI:** 10.1371/journal.pmed.1005050.r002

**Authors:** Louise Gaynor-Brook

30 Mar 2026

Dear Dr Jordan,

Thank you for submitting your manuscript entitled "Was submitted to Plos Medicine with reference: PMEDICINE-D-25-01170. The children left behind – the cumulative impact of congenital anomalies, long-term conditions and poverty on educational attainment in Wales: a population databank linkage study" for consideration by PLOS Medicine.

With apologies for the delay, your manuscript has now been evaluated by the PLOS Medicine editorial staff and by an Academic Editor, and I am writing to let you know that we would like to send your submission out for external peer review.

However, before we can send your manuscript to reviewers, we need you to complete your submission by providing the metadata that is required for full assessment. To this end, please login to Editorial Manager where you will find the paper in the 'Submissions Needing Revisions' folder on your homepage. Please click 'Revise Submission' from the Action Links and complete all additional questions in the submission questionnaire.

For clinical studies, please upload a copy of your trial study protocol as a supporting information file. The study protocol should be the version submitted for approval to the institutional review board or ethics committee, should include any amendments to the study protocol, as well as the date of their approval by the institutional review or ethics committee. Please also detail any deviations from the study protocol in the Methods section of your manuscript. The editors will consider the protocol and study conduct prior to a final decision for external review.

Please re-submit your manuscript within two working days, i.e. by Apr 01 2026 11:59PM.

Login to Editorial Manager here: https://www.editorialmanager.com/pmedicine

Once your full submission is complete, your paper will undergo a series of checks in preparation for peer review. Once your manuscript has passed all checks it will be sent out for review.

Feel free to email me at lgaynor@plos.org if you have any queries relating to your submission.

Kind regards,

Louise Gaynor-Brook, MBBS PhD

Senior Editor

PLOS Medicine

# Author comment: Author response to Decision Letter 1

**DOI:** 10.1371/journal.pmed.1005050.r003

31 Mar 2026

Attachment Submitted filename: pmed_response_auresp_1.docx

# Peer review: Decision Letter 1

**DOI:** 10.1371/journal.pmed.1005050.r004

**Authors:** Louise Gaynor-Brook

18 Jun 2026

Dear Dr Jordan,

Many thanks for submitting your manuscript "The children left behind – the cumulative impact of congenital anomalies, long-term conditions and poverty on educational attainment in Wales: a population databank linkage study" (PMEDICINE-D-26-00859R1) to PLOS Medicine. The paper has been reviewed by subject experts and a statistician; their comments are included below and can also be accessed here: [LINK]

After discussing the paper with the editorial team and an academic editor with relevant expertise, I'm pleased to invite you to revise the paper in response to the reviewers' comments. We plan to send the revised paper to some or all of the original reviewers, and we cannot provide any guarantees at this stage regarding publication.

In addition to these revisions, you may need to complete some formatting changes, which you will receive in a follow up email. A member of our team will be in touch with a set of requests shortly. If you do not receive a separate email within a few days, please assume that checks have been completed, and no additional changes are required.

When you upload your revision, please include a point-by-point response that addresses all of the reviewer and editorial points, indicating the changes made in the manuscript and either an excerpt of the revised text or the location (eg: page and line number) where each change can be found. Please also be sure to check the general editorial comments at the end of this letter and include these in your point-by-point response. When you resubmit your paper, please include a clean version of the paper as the main article file and a version with changes tracked as a marked-up manuscript. It may also be helpful to check the guidelines for revised papers at http://journals.plos.org/plosmedicine/s/revising-your-manuscript for any that apply to your paper.

We ask that you submit your revision by Jul 09 2026 11:59PM. However, if this deadline is not feasible, please contact me by email, and we can discuss a suitable alternative.

Don't hesitate to contact me directly with any questions (lgaynor@plos.org).

Best regards,

Louise

Louise Gaynor-Brook, MBBS PhD

Senior Editor

PLOS Medicine

lgaynor@plos.org

-----------------------------------------------------------

Comments from the academic editor:

The aim of the study is clear in title, but incomplete in abstract and end of introduction. Summary in beginning of discussion could be improved.

-----------------------------------------------------------

Comments from the reviewers:

Reviewer #1: Thank you for addressing my comments. While I do not agree hospitalisation should be a proxy for unmeasured confounder, I understand the rationale. However, such rationale should be clearly, and explicitly stated in the Methods, when you described hospitalisation as a confounder. Please specify what these variable were intended to represent as unmeasured confounders, ideally with a causal diagram to illustrate.

Reviewer #2: This revised version of the manuscript is greatly improved. A few questions and recommendations remain.

First, and especially for finalizing the paper for page proofs, this reviewer strongly urges that the authors use only a single decimal whenever a percent is reported (otherwise, with two decimals you are really providing a rate per 1,000, whereas the units for percents are per 100). Similarly, for odds ratios and confidence intervals, two decimal places are sufficient. Precision of estimate is not improved by reporting these statistics to three decimals.

Second, most of the analyses reported here appear to be for isolated birth defects. But this should be made clearer to the reader throughout. While it may be assumed that educational outcomes are poorer in children with one of these major defects in combination with other defects, that has not been analyzed here (unless I missed this in the methods). Similarly, while there are data to identify which children are in special education placements, even more helpful would be data on specific developmental disabilities or conditions that warranted these placements. There's a substantial overlap between major birth defects and developmental disabilities*, and the analysis would be enriched by incorporating data on disabilities, which are likely available within the SAIL integrated data repository. But that is probably more for a separate analysis - here though the authors could discuss this as a potential limitation and area for further exploration.

Finally, while its unusual, perhaps its ok to end the section on implications, and the manuscript as a whole, with a question. But the ask implied in the final sentence is quite complex, and to some extent transcends what can be learned from the analyses presented here.

All in all, the authors are to be commended for a much improved resubmission.

*Years ago this reviewer summarized the literature on co-occurring birth defects and developmental disabilities: Kirby RS. Co-occurrence of developmental disabilities with birth defects. Ment Retard Dev Disabil Res Rev. 2002;8(3):182-187. doi:10.1002/mrdd.10034

Reviewer #3: Than you for giving me the opportunity to review this paper that looks at the effects of congenital abnormalities on educational attainment. The paper is well written and comprehensive, and the authors have answered the previous round of comments well.  The use of the linked data that are available in SAIL allows for this important and novel analysis. This is a important topic and a well conducted study. However, I have a number of comments:

Your title suggests that congenital abnormalities, poverty and long-term conditions are equally important, however, your analysis mainly focussed on congenital abnormalities. Consider changing the title to better reflect this?

You answered the comments about over-adjustment well. However, the predictive nature of your analysis and the need to avoid causal conclusions needs to be strongly highlighted in the discussion.

One of the issues that is highlighted by the comments is that interpreting ORs for multiple exposures from one model is problematic. I would consider separate models for each exposure. I think a really good example of where this is interpreting an ORs for poverty form table 10; a big issue is very few variables are confounders of the relationship between deprivation and educational outcomes, most factors offer the downstream of deprivation. I think this is a really good example of why multiple DAGs would be so useful.

I think the argument of including hospital stay and comorbidities as covariates remains problematic - they occur after congenital abnormalities so cannot be considered as potential confounders, they are likely mediators. In fact a number of your covariates are not confounders. SEN also a mediator - change the way you discuss results and ensure you don't use any causal language (e.g. potential confounders).

Despite there being no association between IMD and congenital abnormalities, I feel you should still consider this as a mediator as these decisions should be made a priori rather than data driven

Interactions require very large sample sizes, it is worth highlighting that despite a very large sample size in SAIL the study may still have been underpowered

using the EUROCAT categories is fine.

I think the point about combining abnormalities is reasonable and should be highlighted in the limitations.

your justification for using the specific childhood conditions is good. However, this could potentially be strengthened by highlighting how these are related to perinatal issues.

While you explain that a DAG was not included due to complexity Introduced by interactions, I think simply not including the interaction terms would still be beneficial

There is existing evidence of differential consequences across socioeconomic strata of negative perinatal outcomes; referencing this would strengthen your introduction - https://bmjopen.bmj.com/content/bmjopen/15/1/e084147.full.pdf

Your aims can be expanded on more - you've done more than you state

In the background you mention that congenital abnormalities effect educational attainment indirectly through child health, is there not also a direct impact?

Only including mainstream is a limitation - this likely underestimates the effect of congenital abnormalities and the missing data pattern is missing not at random - ensure this is highlighted stronger

You mention that interactions are significant but 2 things - is it multiplicative or additive, ad what direction is the effects (exacerbate or attenuate)

Minor comments:

Figure 1 legend is not in the manuscript

Line 141 - explain why you chose this end date

Line 242/243 - ks2 and ks4 should be the other way around

Line 273 - incorrect capitalisation of they

Line 482 - a-theoretical, incorrect '-'

Line 570-572 - rewrite as a little unclear at the moment

Could the descriptive tables be combined?

Reviewer #4: Thank you for the opportunity to review this manuscript. The authors had conducted an important and highly informative piece of work, which will be of interest to clinicians and policymarkers in general. In reviewing this article, I was able to read through previous reviewers' comments and the authors' responses to these comments as well as the tracked changes made to original manuscript. These are my additional comments:

Introduction

The authors described the prevalence rates of 20% of major anomalies. This figure seemed unexpectedly high and I was unable to identify support for it in the cited references. The prevalence rate of anomalies within the cohort studied were only about 3.5%. Please could the authors check and clarify.

Methods

- The selection of and inclusion of the medicated co-morbidities (epilepsy, diabetes, asthma) in the analyses should be better justified. I understood that the authors seek to explore the impact of co-morbidities on educational attainment. Nonetheless, as described in the results section line 292-293, that there is no association of congenital anomalies with diabetes. What is then the value of including diabetic prescriptions?

- Related to the above point, this particular sentence (line 119-120) in the introduction is confusing and needs rewording: "To include important medicated comorbidities (epilepsy, diabetes, asthma and cardiac conditions), the study was restricted to this portion of the national population."

- Potential confounders (line 179): please specify what perinatal factors were included as covariates.

- Line 135-136, Down syndrome is missing from the text

Results

- The sentence 'Children receiving SEN provision were less successful' in line 332 needs expanding on. Apart from being an intervention, SEN provision may be a marker of disease severity.

- FSM eligibility is a measure for deprivation. Whilst I understand the authors' intention to highlight the impact of both SEN and FSM, the paragraph from line 332 would seem better placed after the results describing the educational attainment of children with and without congenital anomalies.

- Additionally, the results will be more informative if Table 8 is displayed stratifying the cohort into: (i) congenital anomalies with FSM, (ii) congenital anomalies without FSM, (iii) no anomalies with FSM, (iv) no anomalies without FSM.

- Similarly Figure 3 should display include the same information for children without anomalies.

Discussion

- Would children with genetic diagnoses associated congenital anomalies other than chromosomal anomalies be captured within the dataset? It is likely that some of these diagnoses may be associated with intellectual disability, which if not diagnosed, will be misclassified into 'isolated anomalies'. The burden of intellectual disability is then captured in the need for SEN.

Overall the manuscript will benefit from being shortened and language editing to fix typos and grammatical/ punctuation errors.

Any attachments provided with reviews can be seen via the following link: [LINK]

--------------------------------------------------------- ---

General editorial requests

**Please be sure to check the general editorial comments and include these in your point-by-point response.**

(Note: not all will apply to your paper, but please check each item carefully)

*We ask every co-author listed on the manuscript to fill in a contributing author statement, making sure to declare all competing interests. If any of the co-authors have not filled in the statement, we will remind them to do so when the paper is revised. If all statements are not completed in a timely fashion this could hold up the re-review process. If new competing interests are declared later in the revision process, this may also hold up the submission. Should there be a problem getting one of your co-authors to fill in a statement we will be in contact. Please do not add or remove authors without first discussing this with the handling editor. You can see our competing interests policy here: http://journals.plos.org/plosmedicine/s/competing-interests.

*Please upload any figures associated with your paper as individual TIF or EPS files with 300dpi resolution at resubmission; please read our figure guidelines for more information on our requirements: http://journals.plos.org/plosmedicine/s/figures. While revising your submission, we strongly recommend that you use PLOS's NAAS tool (https://ngplosjournals.pagemajik.ai/artanalysis) to test your figure files. NAAS can convert your figure files to the TIFF file type and meet basic requirements (such as print size, resolution), or provide you with a report on issues that do not meet our requirements and that NAAS cannot fix.

After uploading your figures to PLOS's NAAS tool - https://ngplosjournals.pagemajik.ai/artanalysis, NAAS will process the files provided and display the results in the "Uploaded Files" section of the page as the processing is complete.

If the uploaded figures meet our requirements (or NAAS is able to fix the files to meet our requirements), the figure will be marked as "fixed" above. If NAAS is unable to fix the files, a red "failed" label will appear above.

When NAAS has confirmed that the figure files meet our requirements, please download the file via the download option, and include these NAAS processed figure files when submitting your revised manuscript.

*Please ensure that the paper adheres to the PLOS Data Availability Policy (see http://journals.plos.org/plosmedicine/s/data-availability), which requires that all data underlying the study's findings be provided in a repository or as Supporting Information. For data residing with a third party, authors are required to provide instructions with contact information (web or email address) for obtaining the data. Please note that a study author cannot be the contact person for the data. PLOS journals do not allow statements supported by "data not shown" or "unpublished results." For such statements, authors must provide supporting data or cite public sources that include it.

*We expect all researchers with submissions to PLOS in which author-generated code underpins the findings in the manuscript to make all author-generated code available without restrictions upon publication of the work. In cases where code is central to the manuscript, we require the code to be made available as a condition of publication. Authors are responsible for ensuring that the code is reusable and well documented. Please make any custom code available, either as part of your data deposition or as a supplementary file. Please add a sentence to your data availability statement regarding any code used in the study, e.g. "The code used in the analysis is available from Github [URL] and archived in Zenodo [DOI link]" Please review our guidelines at https://journals.plos.org/plosmedicine/s/materials-software-and-code-sharing and ensure that your code is shared in a way that follows best practice and facilitates reproducibility and reuse. Because Github depositions can be readily changed or deleted, we encourage you to make a permanent DOI'd copy (e.g. in Zenodo) and provide the URL.

*Please ensure that the study is reported according to the RECORD guideline and include the completed checklist as Supporting Information. When completing the checklist, please use section and paragraph numbers, rather than page numbers. Please add the following statement, or similar, to the Methods: "This study is reported as per the REporting of studies Conducted using Observational Routinely-collected Data (RECORD) guideline (S1 Checklist)."

FORMATTING - GENERAL

*Abstract: Please structure your abstract using the PLOS Medicine headings (Background, Methods and Findings, Conclusions). Please combine the Methods and Findings sections into one section.

*At this stage, we ask that you include a short, non-technical Author Summary of your research to make findings accessible to a wide audience that includes both scientists and non-scientists. The Author Summary should immediately follow the Abstract in your revised manuscript. This text is subject to editorial change and should be distinct from the scientific abstract. Ideally each sub-heading should contain 2-3 single sentence, concise bullet points containing the most salient points from your study. In the final bullet point of 'What Do These Findings Mean?', please include the main limitations of the study in non-technical language. Please see our author guidelines for more information: https://journals.plos.org/plosmedicine/s/revising-your-manuscript#loc-author-summary.

*Please express the main results with 95% CIs as well as p values. When reporting p values please report as p<0.001 and where higher as the exact p value p=0.002, for example. Throughout, suggest reporting statistical information as follows to improve clarity for the reader "22% (95% CI [13%,28%]; p</=)". Please be sure to define all numerical values at first use.

*Please include page numbers and line numbers in the manuscript file. Use continuous line numbers (do not restart the numbering on each page).

*Please cite the reference numbers in square brackets. Citations should precede punctuation.

FIGURES AND TABLES

*Please provide titles and legends for all figures and tables (including those in Supporting Information files).

*Please define all abbreviations used in each figure/table (including those in Supporting Information files).

*Please consider avoiding the use of red and green in order to make your figure more accessible to those with color blindness.

SUPPLEMENTARY MATERIAL

*Please note that supplementary material will be posted as supplied by the authors. Therefore, please amend it according to the relevant comments outlined here.

*Please cite your Supporting Information as outlined here: https://journals.plos.org/plosmedicine/s/supporting-information

REFERENCES

*PLOS uses the numbered citation (citation-sequence) method and first six authors, et al.

*Please ensure that journal name abbreviations match those found in the National Center for Biotechnology Information (NCBI) databases (http://www.ncbi.nlm.nih.gov/nlmcatalog/journals), and are appropriately formatted and capitalised.

*Where website addresses are cited, please include the complete URL and specify the date of access (e.g. [accessed: 12/06/2023]).

*Please also see https://journals.plos.org/plosmedicine/s/submission-guidelines#loc-references for further details on reference formatting.

STUDY TYPE-SPECIFIC REQUESTS - OBSERVATIONAL STUDIES

*Abstract: Please include the study design, population and setting, number of participants, years during which the study took place (enrolment and follow up), length of follow up, and main outcome measures.

*Please ensure that the study is reported according to the RECORD guideline (available from https://www.record-statement.org) and include the completed checklist as Supporting Information. Please add the following statement, or similar, to the Methods: "This study is reported as per the Reporting of Studies Conducted using Observational Routinely-Collected Data (RECORD) guideline (S1 Checklist)." When completing the checklist, please use section and paragraph numbers, rather than page numbers.

*For all observational studies, in the manuscript text, please indicate: (1) the specific hypotheses you intended to test, (2) the analytical methods by which you planned to test them, (3) the analyses you actually performed, and (4) when reported analyses differ from those that were planned, transparent explanations for differences that affect the reliability of the study's results. If a reported analysis was performed based on an interesting but unanticipated pattern in the data, please be clear that the analysis was data driven.

*Please state in the Methods section whether the study had a prospective protocol or analysis plan. If a prospective analysis plan (from your funding proposal, IRB or other ethics committee submission, study protocol, or other planning document written before analyzing the data) was used in designing the study, please include the relevant document(s) with your revised manuscript as a Supporting Information file to be published alongside your study and cite it in the Methods section. A legend for this file should be included at the end of your manuscript. If no such document exists, please make sure that the Methods section transparently describes when analyses were planned, and when/why any data-driven changes to analyses took place. Changes in the analysis, including those made in response to peer review comments, should be identified as such in the Methods section of the paper, with rationale.

# Author comment: Author response to Decision Letter 2

**DOI:** 10.1371/journal.pmed.1005050.r005

14 Jul 2026

Attachment Submitted filename: Responses to reviewers plosmed_SJ_HTE.docx

# Peer review: Decision Letter 2

**DOI:** 10.1371/journal.pmed.1005050.r006

**Authors:** Louise Gaynor-Brook

26 Aug 2026

Dear Dr. Jordan,

Thank you very much for re-submitting your manuscript "The children left behind – the cumulative impact of congenital anomalies, long-term conditions and poverty on educational attainment in Wales: a population databank linkage study" (PMEDICINE-D-26-00859R2) for review by PLOS Medicine.

I have discussed the paper with my colleagues and the academic editor and it was also seen again by three reviewers. I am pleased to say that provided the remaining editorial and production issues are dealt with we are planning to accept the paper for publication in the journal.

The remaining issues that need to be addressed are listed at the end of this email. Any accompanying reviewer attachments can be seen via the link below. Please take these into account before resubmitting your manuscript:

[LINK]

***Please note while forming your response, if your article is accepted, you may have the opportunity to make the peer review history publicly available. The record will include editor decision letters (with reviews) and your responses to reviewer comments. If eligible, we will contact you to opt in or out.***

In revising the manuscript for further consideration here, please ensure you address the specific points made by each reviewer and the editors. In your rebuttal letter you should indicate your response to the reviewers' and editors' comments and the changes you have made in the manuscript. Please submit a clean version of the paper as the main article file. A version with changes marked must also be uploaded as a marked up manuscript file.

Please also check the guidelines for revised papers at http://journals.plos.org/plosmedicine/s/revising-your-manuscript for any that apply to your paper. If you haven't already, we ask that you provide a short, non-technical Author Summary of your research to make findings accessible to a wide audience that includes both scientists and non-scientists. The Author Summary should immediately follow the Abstract in your revised manuscript. This text is subject to editorial change and should be distinct from the scientific abstract.

In addition to these revisions, you may need to complete some formatting changes, which you will receive in a follow up email. A member of our team will be in touch with a set of requests shortly. If you do not receive a separate email within a few days, please assume that checks have been completed, and no additional changes are required.

We expect to receive your revised manuscript within 1 week. Please email us (plosmedicine@plos.org) if you have any questions or concerns.

We ask every co-author listed on the manuscript to fill in a contributing author statement. If any of the co-authors have not filled in the statement, we will remind them to do so when the paper is revised. If all statements are not completed in a timely fashion this could hold up the re-review process. Should there be a problem getting one of your co-authors to fill in a statement we will be in contact. YOU MUST NOT ADD OR REMOVE AUTHORS UNLESS YOU HAVE ALERTED THE EDITOR HANDLING THE MANUSCRIPT TO THE CHANGE AND THEY SPECIFICALLY HAVE AGREED TO IT.

Please ensure that the paper adheres to the PLOS Data Availability Policy (see http://journals.plos.org/plosmedicine/s/data-availability), which requires that all data underlying the study's findings be provided in a repository or as Supporting Information. For data residing with a third party, authors are required to provide instructions with contact information for obtaining the data. PLOS journals do not allow statements supported by "data not shown" or "unpublished results." For such statements, authors must provide supporting data or cite public sources that include it.

To enhance the reproducibility of your results, we recommend that you deposit your laboratory protocols in protocols.io, where a protocol can be assigned its own identifier (DOI) such that it can be cited independently in the future. Additionally, PLOS ONE offers an option to publish peer-reviewed clinical study protocols. Read more information on sharing protocols at https://plos.org/protocols?utm_medium=editorial-email&utm_source=authorletters&utm_campaign=protocols

Please review your reference list to ensure that it is complete and correct. If you have cited papers that have been retracted, please include the rationale for doing so in the manuscript text, or remove these references and replace them with relevant current references. Any changes to the reference list should be mentioned in the rebuttal letter that accompanies your revised manuscript.

Please note, when your manuscript is accepted, an uncorrected proof of your manuscript will be published online ahead of the final version, unless you've already opted out via the online submission form. If, for any reason, you do not want an earlier version of your manuscript published online or are unsure if you have already indicated as such, please let the journal staff know immediately at plosmedicine@plos.org.

If you have any questions in the meantime, please contact me or the journal staff on plosmedicine@plos.org.

We look forward to receiving the revised manuscript by Sep 02 2026 11:59PM.

Sincerely,

Louise Gaynor-Brook, MBBS PhD

Senior Editor

PLOS Medicine

plosmedicine.org

------------------------------------------------------------

Requests from Editors:

GENERAL EDITORIAL REQUESTS

* At this stage, we ask that you include a short, non-technical Author Summary of your research to make findings accessible to a wide audience that includes both scientists and non-scientists. The Author Summary should immediately follow the Abstract in your revised manuscript. This text is subject to editorial change and should be distinct from the scientific abstract. Ideally each sub-heading should contain 2-3 single sentence, concise bullet points containing the most salient points from your study. In the final bullet point of ‘What Do These Findings Mean?’ Please include the main limitations of the study in non-technical language.Please see our author guidelines for more information: https://journals.plos.org/plosmedicine/s/revising-your-manuscript#loc-author-summary."

* Please confirm that your title complies with PLOS Medicine's style. Your title must be nondeclarative and not a question. It should begin with main concept if possible. "Effect of" should be used only if causality can be inferred, i.e., for an RCT. Please place the study design ("A randomized controlled trial," "A retrospective study," "A modelling study," etc.) in the subtitle (ie, after a colon). We suggest "Associations of congenital anomalies, long-term health conditions and socioeconomic deprivation with educational attainment among children in Wales: A population-based data linkage study" or similar.

* Please confirm that your abstract complies with our requirements, including format (three sections: Background, Methods and Findings, and Conclusions) and providing all the information relevant to this study type https://journals.plos.org/plosmedicine/s/submission-guidelines#loc-abstract

* Please ensure that the Introduction ends with a clear description of the study question or hypothesis.

* Please ensure that all abbreviations are defined at first use throughout the text.

* Please confirm that all numbers presented in the abstract are present and identical to numbers presented in the main manuscript text.

GENERAL

* Please include a statement regarding use or non-use of AI tools and technologies at the end of the Methods. Please specify the name(s) and version(s) of any tool(s) used and a description of their use, including how the AI-assisted or generated content was evaluated by authors, and a clear statement of which aspects of the study, article contents, data, or supporting files were affected or generated by AI tool usage. Please also include a statement specifying who retains responsibility for the final manuscript. See our guidelines for more information: https://journals.plos.org/plosmedicine/s/ethical-publishing-practice#loc-artificial-intelligence-tools-and-technologies.

"Use people with lower incomes, people/households with incomes below the federal poverty level, people experiencing poverty, instead of poverty-stricken, the poor, or poor people." Please see https://www.nih.gov/nih-style-guide/person-first-destigmatizing-language for more information.

Please refer to 'congenital anomalies' throughout, instead of 'anomalies'

"* Statistical reporting: Please revise throughout the manuscript, including tables and figures.

- Please report statistical information as follows to improve clarity for the reader ""22% (95% CI [13,28]; p</=)"".

- Please separate upper and lower bounds with commas instead of hyphens as the latter can be confused with reporting of negative values.

- Please repeat statistical definitions (HR, CI etc.) for each set of parentheses."

* In the last sentence of the Abstract Methods and Findings section, please describe the main limitation(s) of the study's methodology.

* In the abstract, please include the important dependent variables that are adjusted for in the analyses.

Please review your text for claims of novelty or primacy (e.g. 'for the first time') and remove this language. In addition, please check that any use of statistical terms (such as trend or significant) are supported by the data, and if not please remove them.

* In the author summary, in the final bullet point of 'What Do These Findings Mean?', please include the main limitations of the study in non-technical language.

Please remove subheadings from the discussion.

FUNDING STATEMENT

* The funding statement should include: specific grant numbers, initials of authors who received each award, URLs to sponsors’ websites. Also, please state whether any sponsors or funders (other than the named authors) played any role in study design, data collection and analysis, the decision to publish, or preparation of the manuscript. If they had no role in the research, include this sentence: “The funders had no role in study design, data collection and analysis, decision to publish, or preparation of the manuscript.”

COMPETING INTERESTS STATEMENT

* All authors must declare their relevant competing interests per the PLOS policy, which can be seen here: https://journals.plos.org/plosmedicine/s/competing-interests For authors with ties to industry, please indicate whether any of the interests has a financial stake in the results of the current study.

*Please amend your competing interests statement. Funding information should go to the relevant section and include URLs.

DATA AVAILABILITY

"* PLOS Medicine requires that the de-identified data underlying the specific results in a published article be made available, without restrictions on access, in a public repository or as Supporting Information at the time of article publication, provided it is legal and ethical to do so. Please see the policy at

http://journals.plos.org/plosmedicine/s/data-availability and FAQs at http://journals.plos.org/plosmedicine/s/data-availability#loc-faqs-for-data-policy "

"* PLOS defines the “minimal data set” to consist of the data set used to reach the conclusions drawn in the manuscript with related metadata and methods, and any additional data required to replicate the reported study findings in their entirety. Authors do not need to submit their entire data set, or the raw data collected during an investigation. Please submit the following data:

The values behind the means, standard deviations and other measures reported;

The values used to build graphs;

The points extracted from images for analysis."

* Please include the statement on code availability in the data availability statement. Please see guidance here: https://journals.plos.org/plosmedicine/s/code-sharing-guidance#loc-data-availability-statement-examples

ETHICS AND CONSENT

* Please review your manuscript and edit to ensure compliance with our inclusive language requirements https://journals.plos.org/plosmedicine/s/human-subjects-research#loc-categorization

FIGURES AND TABLES

* Please provide titles and legends for all figures and tables (including those in Supporting Information files). Please define all acronyms used in each figure or table in its corresponding legend.

* Please provide the unadjusted comparisons as well as the adjusted comparisons in all relevant Tables

* Please specify the variables controlled for in all relevant Tables

OBSERVATIONAL, COHORT, CROSS-SECTIONAL, AND CASE CONTROL STUDIES

"* Did your study have a prospective protocol or analysis plan? Please state this (either way) early in the Methods section.

a) If a prospective analysis plan (from your funding proposal, IRB or other ethics committee submission, study protocol, or other planning document written before analyzing the data) was used in designing the study, please include the relevant prospectively written document with your revised manuscript as a Supporting Information file to be published alongside your study, and cite it in the Methods section. A legend for this file should be included at the end of your manuscript.

b) If no such document exists, please make sure that the Methods section transparently describes when analyses were planned, and when/why any data-driven changes to analyses took place.

c) In either case, changes in the analysis-- including those made in response to peer review comments-- should be identified as such in the Methods section of the paper, with rationale."

* Your study is observational and therefore causality cannot be inferred. Please remove language that implies causality and refer to associations instead.

* For all observational studies, in the manuscript text, please indicate: (1) the specific hypotheses you intended to test, (2) the analytical methods by which you planned to test them, (3) the analyses you actually performed, and (4) when reported analyses differ from those that were planned, transparent explanations for differences that affect the reliability of the study's results. If a reported analysis was performed based on an interesting but unanticipated pattern in the data, please be clear that the analysis was data-driven.

Please ensure that the study is reported according to the RECORD guideline (available from https://www.record-statement.org) and include the completed checklist as Supporting Information. Please add the following statement, or similar, to the Methods: "This study is reported as per the Reporting of Studies Conducted using Observational Routinely-Collected Data (RECORD) guideline (S1 Checklist)." When completing the checklist, please use section and paragraph numbers, rather than page numbers.

Please refer to 'congenital anomalies' throughout, instead of 'anomalies'

Please note that code availability refers to author-generated code used in the study / data analysis. We expect all researchers with submissions to PLOS in which author-generated code underpins the findings in the manuscript to make all author-generated code available without restrictions upon publication of the work. In cases where code is central to the manuscript, we may require the code to be made available as a condition of publication. Authors are responsible for ensuring that the code is reusable and well documented. Please see https://journals.plos.org/plosmedicine/s/materials-software-and-code-sharing#loc-sharing-code for more information; please read the Code Availablity and Code Sharing Guidance in addition to the policy details listed.

Author Summary: We suggest revising "...rich and poor children..." to e.g. "...children in the least and most deprived groups..."

Author Summary: Please refer to 'congenital anomalies' throughout, instead of 'anomalies'

Author Summary: Please revise/define "affluent" and "poorest" - we suggest language relating to e.g. least deprived.

Author Summary: Please revise/define "succeed" - please include details of the level of achievement required to make this inference.

Author Summary: Please revise the final bullet point to omit '... rests with readers" to reframe this point as a limitation. Please include this and other main limitations of the study in non-technical language in the final bullet point.

Author Summary: In order to retain the bullet point relating to epilepsy, please substantiate your conclusion with the relevant main findings/data under "What Did the Researchers Do and Find?"

Protocol and analysis plan: Please append the relevant documents for publication alongside your manuscript, if possible/permitted.

Abstract: Please revise "Education outcomes predict life chances" and refer to associations instead.

Line 39 - Please clarify what is meant by "academic standards"

Please include the important dependent variables that are adjusted for in the analyses.

In the last sentence of the Abstract Methods and Findings section, please describe the main limitation(s) of the study's methodology.

To enhance accessibility for our broad readership, please briefly define/clarify eligibility for free school meals.

Line 51 - Please revise "successful", and instead refer to educational attainment

Line 52 - Please clarify "these GCSEs"

Line 55 - Please omit '... rests with readers" to reframe this point as a limitation. Please include this and other main limitations of the study in the final sentence of the Abstract Methods and Findings.

Abstract Conclusions: Please refer to objective measures of educational attainment and deprivation quintiles, instead of more subjective terms such as affluence, poor, success, etc.

Please revise "The children left behind"

As your study is observational, causality cannot be inferred. Please remove references to 'impact', 'effect', 'affected' e.g. lines 381, 384, 431, 432, 485, 498, 572 and so on.

Introduction: Line 97 - Please revise to 'associated with'

Line 104 - Please revise to "evidence suggests..." or similar

Line 125 - Please include 'to the best of our knowledge' or similar

Line 129 - Please specify ages refer to years

Please revise all instances of 'affluent', 'rich', 'poor', etc. and refer to the relevant measure(s)/indicator(s) of deprivation used to determine this. This includes the supplementary files.

Please revise all instances of 'successful' and 'failure', and refer to the relevant measure(s)/indicator(s) of success used to determine this e.g. lines 390, 395, 408, 415, 417 and so on. This includes the supplementary files.

NB) ORs for unadjusted analyses are not presented in S4 Table.

Line 359 - For clarity, please expand in the main manuscript text on what is considered a 'more intensive intervention'

Please provide 95% CIs in the tables e.g. Table S5a, S5b, and so on.

Where ORs/aORs are presented in the main manuscript text and tables, please specify the comparison group.

Line 376 - Please clarify "In all"

Line 390 - please specify the comparison group.

Lines 391, 397 - please clarify what is meant by 'passing'

Please present numerators and denominators for percentages, at least in the Tables [not necessarily each time they're mentioned] e.g. Table 9.

Line 474 - Please clarify "Boys did better than girls at KS2..."

Line 554 and elsewhere - Please revise "children with any major anomaly [28, 97] or CHD [22, 33, 34] had poorer educational attainment..." to refer to associations instead e.g. "any major anomaly [28, 97] or CHD [22, 33, 34] was associated with poorer educational attainment..."

Line 581 - please refer to e.g. reduced chance

Comments from Reviewers:

Reviewer #1: Thank you for providing the additional rationale. I have no further comments.

Reviewer #2: The authors were provided with a prodigious number of comments and suggestions, to which they have responded admirably. While one can quibble about minor points here and there, on balance these have been handled well, and in my opinion the manuscript is ready to proceed toward publication.

Reviewer #3: Thank you for giving me the opportunity to re-review this paper. the authors have answered all the comments satisfactorally

Any attachments provided with reviews can be seen via the following link:

[LINK]

# Author comment: Author response to Decision Letter 3

**DOI:** 10.1371/journal.pmed.1005050.r007

25 Sep 2026

Attachment Submitted filename: responses to reviewers.docx

# Editor report: Decision Letter 3

**DOI:** 10.1371/journal.pmed.1005050.r008

**Authors:** Suzanne De Bruijn

25 Sep 2026

Dear Dr Jordan,

On behalf of my colleagues and the Academic Editor, Lars Persson, I am pleased to inform you that we have agreed to publish your manuscript "Associations of major congenital anomalies, long-term health conditions and socioeconomic deprivation with educational attainment in Wales: a population-based data linkage study" (PMEDICINE-D-26-00859R3) in PLOS Medicine.

Before your manuscript can be formally accepted you will need to complete some formatting changes, which you will receive in a follow up email. Please be aware that it may take several days for you to receive this email; during this time no action is required by you. Once you have received these formatting requests, please note that your manuscript will not be scheduled for publication until you have made the required changes.

In the meantime, please log into Editorial Manager at http://www.editorialmanager.com/pmedicine/, click the "Update My Information" link at the top of the page, and update your user information to ensure an efficient production process.

PRESS

We frequently collaborate with press offices. If your institution or institutions have a press office, please notify them about your upcoming paper at this point, to enable them to help maximise its impact. If the press office is planning to promote your findings, we would be grateful if they could coordinate with medicinepress@plos.org. If you have not yet opted out of the early version process, we ask that you notify us immediately of any press plans so that we may do so on your behalf.

We also ask that you take this opportunity to read our Embargo Policy regarding the discussion, promotion and media coverage of work that is yet to be published by PLOS. As your manuscript is not yet published, it is bound by the conditions of our Embargo Policy. Please be aware that this policy is in place both to ensure that any press coverage of your article is fully substantiated and to provide a direct link between such coverage and the published work. For full details of our Embargo Policy, please visit http://www.plos.org/about/media-inquiries/embargo-policy/.

Thank you again for submitting to PLOS Medicine. We look forward to publishing your paper.

Sincerely,

Suzanne De Bruijn, PhD

Associate Editor

PLOS Medicine
